# Elevated acetyl‐CoA by amino acid recycling fuels microalgal neutral lipid accumulation in exponential growth phase for biofuel production

**DOI:** 10.1111/pbi.12648

**Published:** 2016-11-08

**Authors:** Lina Yao, Hui Shen, Nan Wang, Jaspaul Tatlay, Liang Li, Tin Wee Tan, Yuan Kun Lee

**Affiliations:** ^1^ Department of Microbiology and Immunology Yong Loo Lin School of Medicine National University of Singapore Singapore Singapore; ^2^ Department of Chemistry University of Alberta Edmonton Alberta Canada; ^3^ Department of Biochemistry Yong Loo Lin School of Medicine National University of Singapore Singapore Singapore; ^4^ National Supercomputing Centre (NSCC) Singapore Singapore

**Keywords:** microalga, TAG, growth phase, acetyl‐CoA, BCAA, biofuel

## Abstract

Microalgal neutral lipids [mainly in the form of triacylglycerols (TAGs)], feasible substrates for biofuel, are typically accumulated during the stationary growth phase. To make microalgal biofuels economically competitive with fossil fuels, generating strains that trigger TAG accumulation from the exponential growth phase is a promising biological approach. The regulatory mechanisms to trigger TAG accumulation from the exponential growth phase (TAEP) are important to be uncovered for advancing economic feasibility. Through the inhibition of pyruvate dehydrogenase kinase by sodium dichloroacetate, acetyl‐CoA level increased, resulting in TAEP in microalga *Dunaliella tertiolecta*. We further reported refilling of acetyl‐CoA pool through branched‐chain amino acid catabolism contributed to an overall sixfold TAEP with marginal compromise (4%) on growth in a TAG‐rich *D. tertiolecta* mutant from targeted screening. Herein, a three‐step α loop‐integrated metabolic model is introduced to shed lights on the neutral lipid regulatory mechanism. This article provides novel approaches to compress lipid production phase and heightens lipid productivity and photosynthetic carbon capture via enhancing acetyl‐CoA level, which would optimize renewable microalgal biofuel to fulfil the demanding fuel market.

## Introduction

To replace traditional fossil fuels and develop sustainable energy production, identifying sources of biologically derived fuels is increasingly urgent. Microalgae is recognized as a promising alternative source as they can accumulate neutral lipid, mainly in the form of triacylglycerol (TAG), which can be converted into biodiesels readily (Hossain *et al*., 2008). In recent years, many attempts have been undertaken for the enhancement of TAG overproduction in microalgae (Radakovits *et al*., 2010). These approaches mainly focus on biochemical and genetic engineering of lipid biosynthesis pathways and blocking of competing pathways (such as carbohydrate formation), so as to increase the pool of metabolites available for TAG biosynthesis (Courchesne *et al*., 2009; Sharma *et al*., 2012). However, almost all these approaches led to TAG accumulation in the stationary growth phase at the expense of biomass accumulation (Chiu *et al*., 2009; Wang *et al*., 2009) and overall lipid productivity. Vigorous growth and TAG accumulation appear to be mutually exclusive as TAG is a secondary (storage) metabolite and the pyruvate to acetyl‐CoA (AcCoA) pathway is tightly regulated by the growth‐dependent pyruvate dehydrogenase complex activity (Li *et al*., 2014; Oliver *et al*., 2009).

The oleaginous diatom *Fistulifera solaris* JPCC DA0580 was the first to be reported to have a temporal overlap of TAG accumulation and cell growth during the exponential growth phase (Satoh *et al*., 2013). Such a feature that triggers TAG accumulation while maintaining high growth rate is a critical advantage in the large‐scale cultivation of oleaginous microalgae for TAG production. To further exploit this potential in microalgae, fast‐growing, TAG‐rich, easily cultivated *Dunaliella tertiolecta* was used as the experimental organism (Rismani‐Yazdi *et al*., 2011; Shin *et al*., 2015; Yao *et al*., 2015).

In microalgae, AcCoA, malonyl‐CoA and NADPH are the major substrates in the plastid supporting fatty acid synthesis. Malonyl‐CoA is also generated from carboxylation of AcCoA. Thus, AcCoA is the primary precursor for fatty acid synthesis (Garrett and Grisham, 2013). The AcCoA balance in an algal cell could be described by the following equation:

$$\left[{\text{AcCoA}}_{T}\right]-\left[{\text{AcCoA}}_{B}\right]=\left[{\text{AcCoA}}_{\mathrm{NL}}\right]$$

Total AcCoA ([AcCoA_T_]) and reduced NADH are produced via glycolysis. In the exponential growth phase, NADH is mainly oxidized through respiration to yield ATP, and AcCoA is used predominantly for biomass growth ([AcCoA_B_]), including that for structural lipid (glycerophospholipids) synthesis, while a minor fraction of AcCoA ([AcCoA_NL_]) and reduced NAD(P)H is used for fatty acid synthesis to accumulate neutral lipids (TAG). When microalgal cells enter the stationary growth phase, carbon metabolism for biomass growth diminished, which leads to accumulation of AcCoA and reduced NADH. Thus, in stationary phases or growth hindering stress conditions, a conspicuous fraction of AcCoA and reduced NAD(P)H is channelled to fatty acids biosynthesis, resulting in TAG accumulation in cells (Carpinelli *et al*., 2014). Recent studies also suggested that intracellular membrane remodelling contributed to TAG accumulation during stationary phase or nitrogen starvation (Simionato *et al*., 2013; Urzica *et al*., 2013; Yoon *et al*., 2012). To accelerate TAG accumulation in exponential growth phase (TAEP) while maintaining cell growth, AcCoA ([AcCoA_T_]) level should be elevated over a certain set point that is needed for biomass growth ([AcCoA_B_]).

There are three principal sources of AcCoA during growth phase, namely fatty acid oxidation, glycolysis pathway and amino acid degradation (Garrett and Grisham, 2013). Fatty acid oxidation is the reversal of fatty acid synthesis and does not generate *de novo* AcCoA. Instead, it is thought that AcCoA is largely derived from the glycolytic pathway via pyruvate. Pyruvate is converted to AcCoA by PDHC in mitochondria and chloroplasts, and this step has been suggested as the key rate limiting step (Garrett and Grisham, 2013; Oliver *et al*., 2009; Tovar‐Méndez *et al*., 2003). One approach to increase AcCoA production is to relieve pyruvate dehydrogenase kinase (PDK) control of pyruvate dehydrogenase complex (PDHC) resulting in the activation of PDHC. This would facilitate the bioconversion of pyruvate to AcCoA and enhance the metabolic flux towards both cell growth via the TCA cycle, and fatty acid biosynthesis in the growth phase. The third source of AcCoA, which derived from amino acid degradation, has largely been ignored as a relevant pathway for bioengineering. Despite the fact that it bypasses pyruvate and the highly controlled PDHC/PDK regulatory process, it was considered insufficient for fatty acid biosynthesis (Garrett and Grisham, 2013).

We hypothesized that increase of AcCoA pool by multiple routes could trigger TAEP. In our study, from the activation of pyruvate to AcCoA reaction by addition of sodium dichloroacetate (DCA) to release the PDHC/PDK regulatory process, we achieved TAEP in the wild‐type (WT) *D. tertiolecta*. Besides this conventional *de novo* synthetic pathway, we questioned the contribution of amino acid degradation on TAEP, although it has largely been ignored. Through performing genetic engineering, we generated mutants, which exhibited pronounced TAEP with little compromise on growth rate. By employing transcriptomics and metabolomics, key phenotypic regulatory characteristics of lipogenesis in this microalga were uncovered, implying that a secondary contributor of AcCoA derived from amino acid catabolism, in particular branched‐chain amino acid catabolism, contributed to TAEP. Although no direct transport of AcCoA between subcellular compartments was reported in plant cells, a PDHC bypass pathway from activation of free acetate into AcCoA exists (Li‐Beisson *et al*., 2013; Lin and Oliver, 2008). These two major approaches were proposed in our three‐step α loop model. The results highlight the complex interplay between microalgal cellular proliferation and carbon flux in lipogenesis and suggested that genetic and metabolic manipulations targeted at amino acid catabolism could be used to increase accumulation of fuel‐relevant molecules in microalgae in the exponential growth phase.

## Results

### DCA treatment elevated AcCoA pool

After addition of DCA to the WT *D. tertiolecta*, TAG was found to be accumulated in the exponential growth phase, as shown in Figure S1a, with marginal comprise on growth (Figure S1b). AcCoA, the primary precursor for growth and fatty acid synthesis, was found 1.8‐fold that in the control (Figure S1c).

AcCoA is *de novo* converted from pyruvate, which is tightly regulated by PDHC, which catalyses the oxidative decarboxylation of pyruvate. PDHC could be deactivated by PDK through reversible ATP‐dependent phosphorylation mainly in mitochondria (Kato *et al*., 2007). To deactivate PDK, DCA was added to the algal culture medium. DCA is a by‐product of chlorine disinfection process, which inhibit PDK, through formation of DCA helix bundle in the N‐terminal domain of PDK (Kato *et al*., 2007; Miller and Uden, 1983). Bound DCA promotes local conformational changes that are communicated to both nucleotide‐binding and lipoyl‐binding pockets of PDK, leading to the inactivation of kinase activity (Kato *et al*., 2007). Thus, when DCA was included in the culture medium, PDHC became active as PDK was blocked resulting in an elevation of AcCoA (Figure S1c).

### FACS enriched a pool of mutant strains with higher TAG content

**Figure 1:**
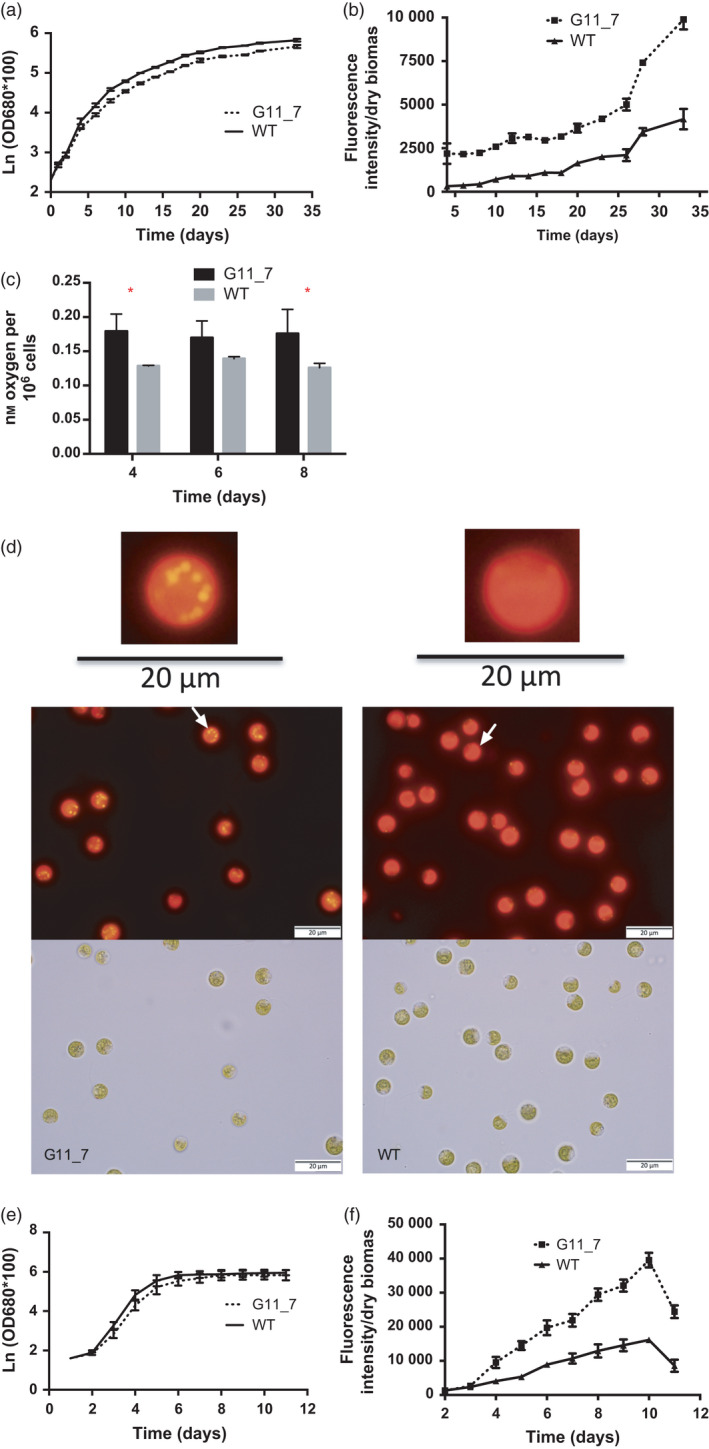
Physiological performance of G11_7 mutant versus WT *Dunaliella tertiolecta*. (a) Growth curve monitored by spectrophotometry under low light. (b) TAG quantitative assay by Nile red staining method under low light. (c) Photosynthetic rate of G11_7 mutant and WT *D. tertiolecta*. (d) Microscopy images (above, Nile red fluorescence; bottom, bright field) of G11_7 mutant (left) and WT (right) under low light (under 100× objective). (e) Growth curve monitored by spectrophotometry under high light. (f) TAG quantitative assay by Nile red staining method under high light. (LL: 30 μmol photons m^−2^ s^−1^, HL: 320 μmol photons m^−2^ s^−1^). Error bars, SEM. Statistical analyses were performed using Student's *t*‐test, * 0.01 ≤ *P* < 0.05; ** 0.001 ≤ *P* < 0.01; *** *P* < 0.001.

We generated TAG‐rich mutant library via genetic engineering and two rounds of fluorescence‐activated cell sorting (FACS) (Terashima *et al*., 2015). All the 27 isolated strains showed reproducible increase in Nile red signal (Figure S2e). Further observation on top six mutants showed consistent higher TAG content with a statistical significant *P* value <0.01 (*t*‐test) comparing to the WT (Figure S2f). Among all the mutants, we selected the stable mutant strain G11_7, which was one of the higher TAG producers at its exponential growth phase for characterization. G11_7 mutant accumulated TAG at its exponential growth phase (Figure 1b) with marginal difference on growth (Figure 1a). On culture day 4, *P* value of TAG accumulation per biomass from the two strains is 0.0013 (**), and the rest of the time points are all less than 0.001 (***), indicating that TAG accumulation per biomass between G11_7 and WT is significantly different throughout all the culture points (Figure 1b). It consistently showed enhanced TAG production (about twofold to sevenfold) compared to WT *D. tertiolecta* (WT). In addition, the mutant had a significant better photosynthetic performance at the same light condition (Figure 1c, 33% higher than WT). It is evidence that the mutant has enhanced energy/carbon capture capacity. Lipid droplets (LPs) were visualized by fluorescent microscopy (Figure 1d) in G11_7 mutant, showing golden‐yellow fluorescence. The TAG accumulation in cultures grown under high‐light condition showed a similar trend as low‐light condition (Figure 1e,f). The fatty acid composition of the mutant and WT is presented in Figure S3a, with a typical profile of unsaturated fatty acids 16 : 1 and 18 : 3(n‐3) being the predominant fatty acids. There is a significant increase in the monounsaturated fatty acid (MUFA) with most others remained similarly (Figure S3b).

### Altered expression level of genes in amino acid catabolism in G11_7 at the exponential growth phase

**Figure 2:**
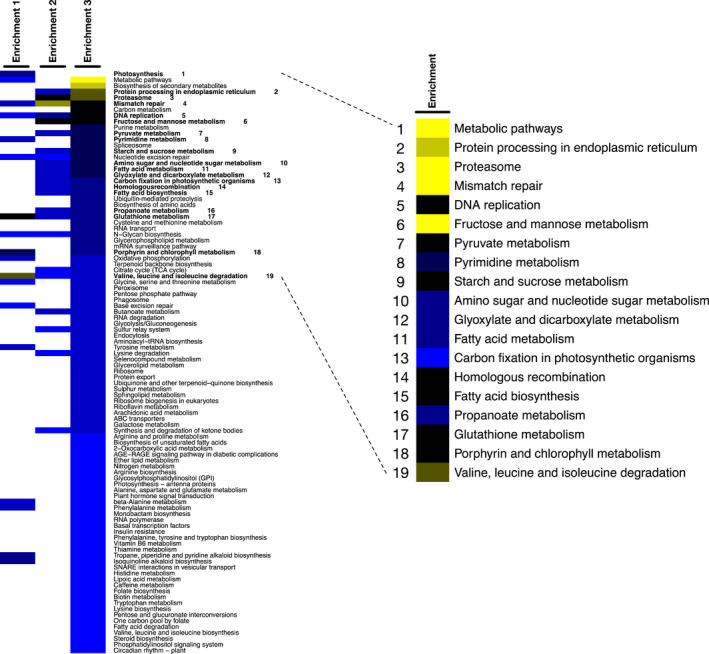
Heat map of the KEGG profiles. Colours represent abundance of KEGG enrichment score (blue to yellow, ascending enrichment score). Rows of the heat map represent pathways from three analyses (Enrichment 1: Partek workflow; Enrichment 2: in‐house workflow), background KEGG gene that derived from *C. reinhardtii* as the reference was set at 3321; Enrichment 3: in‐house workflow, background KEGG gene derived from *D. tertilecta* transcriptome nonredundant database was set at 10,579. Images were made with R (http://www.r-project.org/). Significant KEGG pathways are marked by bold font with number on the left and expansion on the right.

Differential expressed genes are presented in Supplementary Data Set 1. KEGG enrichment scores were calculated and shown in Figure 2 (Chen *et al*., 2015; Kanehisa *et al*., 2016). There are in total 105 KEGG enrichment scores featured in the three predicted analyses, among which 19 KEGG pathways were found significant in at least two enrichment analyses according to scores. The important genes that altered in expression levels in the mutant as compared to the WT are summarized in Supplementary Data Set 1. Valine, leucine and isoleucine (branched‐chain amino acids, BCAA) degradation pathway is the most significantly affected pathway detected in all three analyses. *CuAO* (or *AMX1*, K00276) (copper amine oxidase family), *IVD* (K00253) (isovaleryl‐CoA‐dehydrogenase) and *MCCB* (K01969) (3‐methylcrotonyl‐CoA carboxylase) were the top hits at expression level in both Partek analysis and in‐house workflow.

The complete cDNA sequence of *D. tertiolecta* copper amine oxidase gene (1524‐bp encoding for 507 amino acids), isovaleryl‐CoA dehydrogenase gene (1053‐bp encoding for 350 amino acids) and 3‐methylcrotonyl‐CoA carboxylase beta subunit gene (1725‐bp encoding for 574 amino acids) was obtained using RACE PCR. The candidate *D. tertiolecta* copper amine oxidase gene contained a copper amine oxidase enzyme domain and showed the highest (60%) amino acid homology compared to copper amine oxidase of *Volvox carteri* f. nagariensis, and was designated *DtCuAO* (*AMX1*). The candidate *D. tertiolecta* isovaleryl‐CoA dehydrogenase gene contained an isovaleryl‐CoA dehydrogenase domain and showed the highest (76%) homology in amino acid sequence compared to that of *Chlamydomonas reinhardtii*, and was designated *DtIVD* (*ACAD*). The candidate *D. tertiolecta* 3‐methylcrotonyl‐CoA carboxylase beta subunit gene contained a 3‐methylcrotonyl‐CoA carboxylase beta chain domain and showed the highest (67%) homology in amino acid sequence compared to that of *C. reinhardtii*, and was designated *DtMCCB*. Amino acid sequences of *CuAO*,* IVD* and *MCCB* from other species were obtained by BLAST search in NCBI database with the putative DtCuAO, DtIVD, DtMCCB. The phylogenetic tree constructed by MEGA5 demonstrated that the putative DtCuAO, DtIVD and DtMCCB showed high homology with CuAO, IVD and MCCB, respectively, from other species (Figure S4). Using the predicted sequence of these three genes from the *D. tertiolecta* database, we designed primers and confirmed by high‐fidelity DNA polymerases sequencing. The cDNA sequences of the three genes were also attached as Supplementary Data Set 3a‐c. We arbitrarily tested two genes for their expression levels by quantitative real‐time PCR at different growth phases (Figure S5a) using the *D. tertiolecta* beta‐tubulin gene (DtTUB) as the internal standard for normalization (Lin *et al*., 2013). It showed a similar tendency with that of RNA‐Seq data. Interestingly, the fold changes in the gene expression level and lipid level are correlated, suggesting that they are the key genes regulating the lipid accumulation process. The *DtPDK* gene that subsequently measured in the DCA treatment experiment was discovered by the prediction from the *D. tertiolecta* in‐house database (known as Locus_5000_7Transcript_1/1_Confidence_1.000_Length_2194) and confirmed by experimental sequencing using high‐fidelity DNA polymerases and shown in Supplementary Data Set 3d. Interestingly, we found mRNA expression levels of these three important genes, *DtIVD*,* DtCuAO* and *DtMCCB*, were all up‐regulated in other high‐TAG strains (G11_18, G11_20, G11_25) as shown in Figure S5b.

In the upstream pathways, up‐regulation of *ACCA* (K00626) (acetyl‐CoA C‐acetyltransferase) was also detected, which resulted in the accumulation of AcCoA. Up‐regulation of the downstream *FabD* (K00645) (malonyl‐CoA:acyl‐carrier‐protein transacylase) contributed to accumulation of fatty acids in chloroplasts. The fast fatty acid accumulation might cause a drawn‐down in AcCoA, thus initiating a pull‐down from upstream photosynthetic pathways. The pull‐down may have caused an enhancement of photosynthetic rate (Figure 1c) to support AcCoA *de novo* synthesis, which was supported by the up‐regulation of upstream photosynthesis and glycolysis genes, including *petE* (K02638) (photosynthetic electron transport), *petC* (K02636) (cytochrome b6‐f complex), *LHCA1* (K08907) and *LHCB1* (K08912) (light‐harvesting complex I chlorophyll a/b binding protein 1), *pfkA* (K00850) (6‐phosphofructokinase 1), *PPC* (K01610) (phosphoenolpyruvate carboxykinase (ATP) and *FBP* (K03841) (fructose‐1,6‐bisphosphatase I).

### AcCoA pool was maintained at high level in the mutant

**Figure 3:**
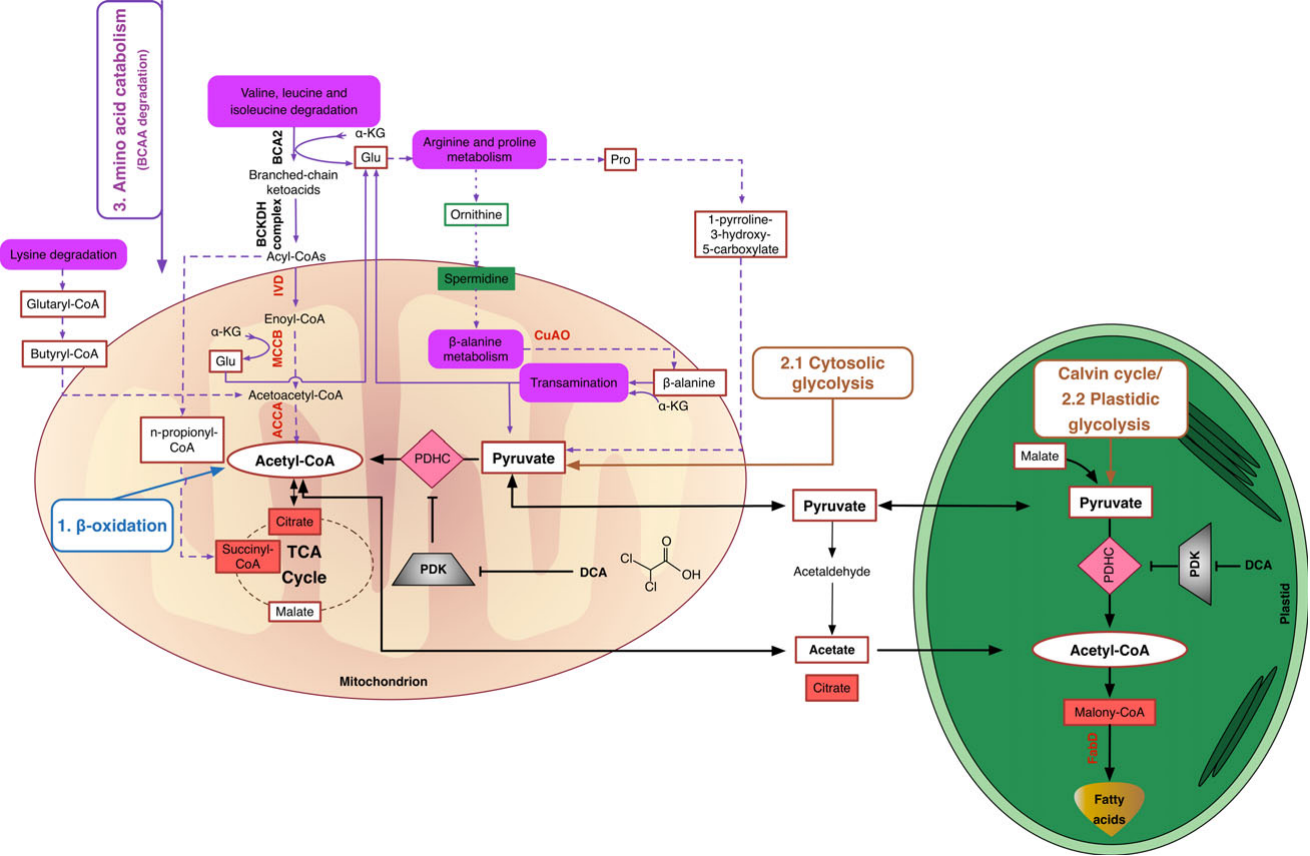
Hypothesized *D. tertiolecta* G11_7 mutant fatty acid metabolic pathways. Enzyme names and intermediates are abbreviated in the figure. Validated overexpressed gene‐coding enzymes are shown in red colour font sitting on the pathway lines. Validated significantly increased intermediates are surrounded by red solid line rectangles, and decreased intermediates are surrounded by green solid line rectangles. The increased/decreased intermediates with a fold change ≥2 or ≤−2 are shown in a filled rectangles with red/green colour. The general three sources of acetyl‐CoA are 1, β‐oxidation, 2, glycolysis and 3: amino acid catabolism. The specific core pathways use solid arrows for one‐step reactions and dash arrows for multiple‐step reactions. The DCA treatment‐related pathways have two critical enzymes, PDH and PDK in pink prismatic and grey trapezoid shapes, respectively. Note: The above showed enzyme and intermediate abbreviations are listed below: BCA2, branched‐chain amino acid aminotransferase; BCKDH, branched‐chain ketoacid dehydrogenase; IVD, isovaleryl‐CoA dehydrogenase; MCCB, 3‐methylcrotonyl‐CoA carboxylase beta unit; ACCA: acetyl‐CoA C‐acetyltransferase; AMX1, copper amine oxidase family protein. PDHC, pyruvate dehydrogenase complex; PDK, pyruvate dehydrogenase kinase; FabD: malonyl‐CoA:acyl‐carrier‐protein transacylase; α‐KG, α‐ketoglutarate; Glu, Glutamate; Pro, Proline.

According to the congruent transcriptome and metabolome analysis (Table S1), the tentative pathways for channelling of metabolites towards fatty acid syntheses are depicted in Figure 3. AcCoA was found 1.3‐fold in G11_7 mutant.

**Figure 4:**
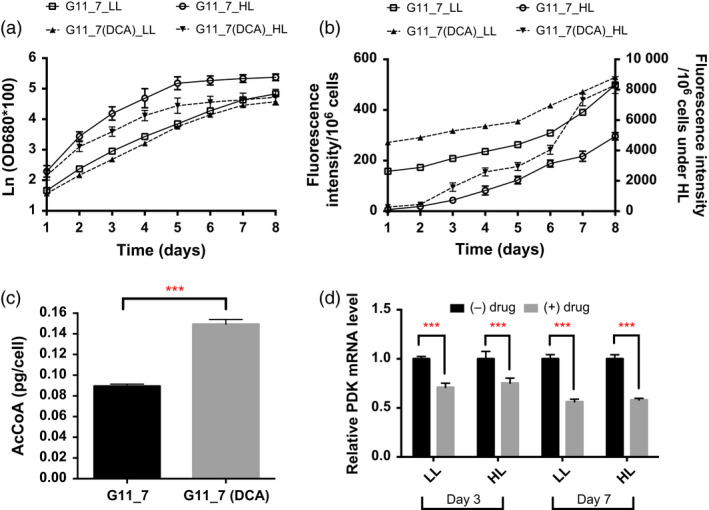
Physiological study and DtPDK mRNA expression levels of G11_7 mutant after DCA treatment. (a) Growth curve monitored by spectrophotometry under low‐light (LL) and high‐light (HL) conditions (LL: 30 μmol photons m^−2^ s^−1^, HL: 320 μmol photons m^−2^ s^−1^). (b) TAG quantitative assay by Nile red staining method under low‐light (LL) and high‐light (HL) conditions (left y axis shows the LL fluorescent performance, right y axis shows the HL fluorescent performance). (c) Acetyl‐CoA level change after addition of DCA to G11_7 under the low‐light condition. (d) Relative DtPDK mRNA expression levels on day 3 and day 7 under different light conditions (LL: 30 μmol photons m^−2^ s^−1^, HL: 320 μmol photons m^−2^ s^−1^). Error bars, SEM. Statistical analyses were performed using Student's *t*‐test, * 0.01 ≤ *P* < 0.05; ** 0.001 ≤ *P* < 0.01; *** *P* < 0.001.

Pyruvate, malate, proline and 1‐pyrroline‐3‐hydroxy‐5‐carboxylate were accumulated in G11_7 mutant, suggesting the relief of PDHC/PDK regulatory process was necessary for efficient bioconversion of the pyruvate‐dependent fatty acid precursors. To achieve this, DCA was further added to the mutant culture medium. TAG accumulation and growth curve in the DCA‐treated G11_7 mutant under both low‐light and high‐light conditions are shown in Figure 4a‐b. There was a significant enhancement of TAG accumulation detected in DCA‐treated mutant cells from exponential growth phase, with an elevated AcCoA level (Figure 4c). Consistently, mRNA expression level of the *DtPDK* was significantly reduced in DCA‐treated groups (Figure 4d). It was also reasoned that under the condition of excess energy supply (in HL), the overflow of AcCoA towards TAG would be more pronounced. Indeed, it was observed that TAG accumulation was further induced when the G11_7 cells (after DCA treatment) entered the stationary phase from day 6 in the HL culture.

### BCAAs fuelled acetyl‐CoA production for TCA metabolism and lipogenesis

In contrast to the WT, the mutant G11_7 has an activated BCAA catabolism via intensive up‐regulation of IVD, MCCB and ACCA, resulting in an enhancement of the AcCoA pool. The increase in glutaryl‐CoA (1.8‐fold) and butyryl‐CoA (1.3‐fold) participating in the lysine degradation pathway also contributed to AcCoA via the up‐regulation of ACCA. The acetyl residue of AcCoA enters the TCA cycle by reaction with oxaloacetate and subsequently incorporated into citrate (2.12‐fold). According to the targeted CoA analyses, n‐propionyl‐CoA, a prerequisite for succinyl‐CoA, had a 1.7‐fold increase in the mutant. Propionyl‐CoA is known to be carboxylated to generate methylmalonyl‐CoA, which is racemized and then isomerized to form succinyl‐CoA (5.9‐fold), a member of the TCA cycle (Ge *et al*., 2014). The flux from both citrates in the TCA cycle and propionyl‐CoA accounts for the massive increase of succinyl‐CoA in the mutant.

Thus, besides priming the TCA cycle for cell growth, the overproduced AcCoA was channelled into the fatty acid reservoir. The enhancement of malonyl‐CoA (2.1‐fold), which plays a key role in the fatty acid biosynthesis and chain elongation, provides the evidence for this.

**Figure 5:**
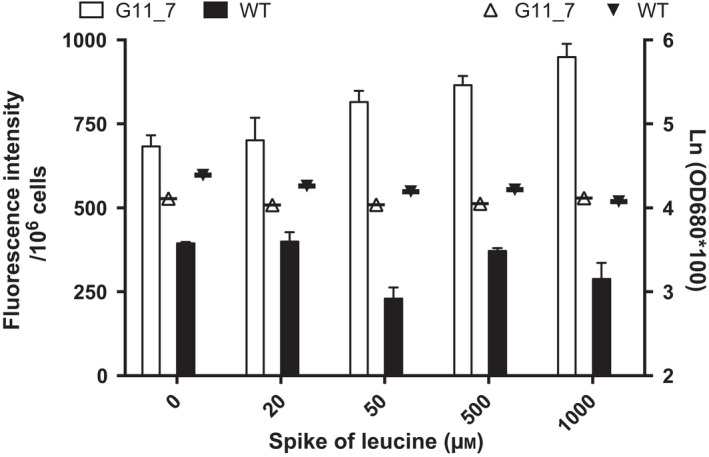
Effect of leucine spike to TAG accumulation and culture growth. TAG contents of G11_7 and WT are presented in bars (primary axis, white bar—G11_7, black bar—WT), and the corresponding culture growth is presented in scatter dot plots (secondary axis, white upward triangle—G11_7, black downward triangle—WT) under different leucine concentration, with all *P* value <0.001.

BCAA catabolic flux was activated by the aforementioned genes, particularly in the leucine degradation pathway. To confirm the contribution of leucine in TAG synthesis in BCAA catabolism, G11_7 mutant and WT were given a spike of different concentrations of leucine in the normal ATCC medium. In contrast to the WT, which did not metabolize leucine as the precursor for cell growth and lipid production, G11_7 mutant strain showed increase TAG production in a dose‐dependent manner (Figure 5). Increase in TAG content with addition of leucine in the medium accounted for as much as 39% of TAG pools.

These data provide experimental evidence for the functional role of BCAA catabolism, reinforcing its importance in regulating lipogenesis in the exponential growth phase. There might be bottlenecks in the synthesis of structural carbohydrates, proteins and lipids for additional cell growth, as no increase in cell growth rate was observed. Instead, pronounced increase of fatty acid was detected providing a hint that fatty acid biosynthesis‐related processes might not be rate limiting during the exponential cell growth phase, and AcCoA generated from the BCAA pathway was transported into chloroplasts for fatty acid accumulation in the mutant.

## Discussion

The primary physiological purpose of amino acids is to serve as the building blocks for protein biosynthesis in eukaryotic cells, and as a consequence, the amount of free amino acids is trivial under most circumstances especially in cultures under high growth rate (Garrett and Grisham, 2013). Amino acids are derived from the TCA cycle, which provides carbon skeletons via 2‐oxoglutarate or oxaloacetate (Kanehisa *et al*., 2016; Lane, 2009; Wagner, 2014). New amino acids can also be formed from transamination by transferring the amino group to a ketoacid (Booth, 2000). In our case, a significant proportion of free amino acids were degraded, driven by transcriptional up‐regulation of *DtIVD*,* DtMCCB* and *DtACCA* encoding for the key enzymes in the BCAA catabolic pathway, leading to AcCoA synthesis. This strategy shows the feasibility of using the aforementioned third source for AcCoA, which was previously ignored.

The functional role of BCAA catabolic process in lipogenesis has been demonstrated in other various organisms. In the diatom, *Phaeodactylum tricornutum*, inhibition of *MCCB* expression by RNA interference disturbed the carbon flux, resulting in decreased TAG accumulation and impaired biomass growth (Ge *et al*., 2014). Green *et al*. (2016) highlighted the contribution of BCAAs to adipocyte metabolism in mouse cell line (3T3‐L1 cells) and demonstrated that amino acids (BCAAs in particular) from both extracellular sources and protein catabolism were highly utilized by differentiated adipocytes. Inhibition of BCAA catabolism negatively influenced 3T3‐L1 adipogenesis. In the study of Peng *et al*. (2015), BCAA catabolic mutants defective in enzymes both upstream and downstream of IVD displayed enhanced senescence in prolonged darkness, showing that function of BCAA catabolism in providing TCA cycle substrates in energy‐limited conditions. It also demonstrated that IVD influences energy homeostasis in multiple ways, providing BCAA catabolic CoA intermediates to the mitochondrial electron transport chain, as well as catabolizing additional substrates such as phytanoyl‐CoA and aromatic amino acids (Araújo *et al*., 2010; Ishizaki *et al*., 2005).

Interestingly, other amino acid catabolic pathways were found in concordant in contributing to the TCA cycle and AcCoA production. Lysine metabolism was previously demonstrated to interact with plant energy metabolism (Angelovici *et al*., 2011). Vorapreeda *et al*. (2012) also reported that leucine and lysine degradation in oleaginous fungi provided the alternative substrate for AcCoA as the precursor for lipid production, by contrast to that in nonoleaginous fungi. This is confirmed by the free amino acid (leucine) uptake study reported here. Free amino acid uptake (transport, assimilation /accumulation) and excretion had been observed in microalgae (Flynn and Butler, 1986; Huo *et al*., 2011). Three exogenous transamination and deamination cycles introduced by Huo *et al*. (2011) also reengineered carbon flux for fuel‐convertible amino acids and enabled protein hydrolysates to be used for fuel production.

**Figure 6:**
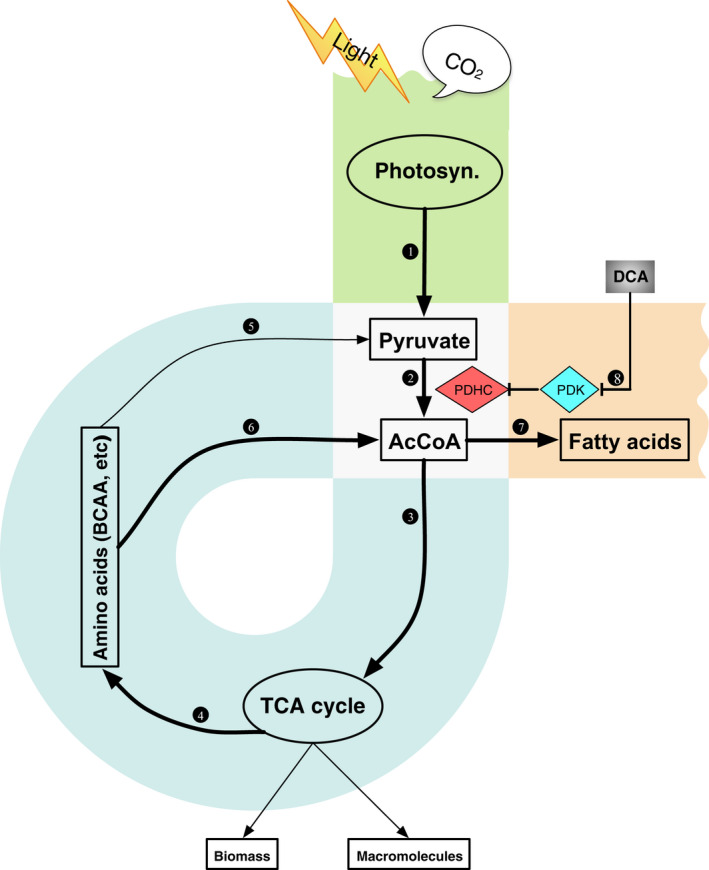
Regulation of metabolic pathways related to energy/carbon capture and conversion in *Dunaliella tertiolecta* mutant G11_7.

In conclusion, we have studied a TAG‐rich mutant strain of *D. tertiolecta* under controlled laboratory conditions to advance our understanding of lipid metabolic pathways in the growth phase. Our study revealed a ‘three‐step α loop’ model to elucidate lipogenesis in the exponential growth phase as shown in Figure 6 and summarized below.

### The vertical path

Under normal conditions, a number of key enzymes in microalgae supply carbon precursors for *de novo* fatty acid synthesis, which include those involved in PDHC, glycolysis and suites of specific transporters. They were found substantially up‐regulated under nitrogen deprivation conditions (Li *et al*., 2014). This pyruvate‐dependent glycolysis pathway is tightly regulated by cell growth via PDHC/PDK cascade (Figure 6, route 1, 2). AcCoA and NADPH produced from this pathway are readily used by the TCA cycle to produce amino acids, macromolecules and energy for biomass growth. During this process, a minor fraction of AcCoA and NADPH is used for fatty acids synthesis (Carpinelli *et al*., 2014).

### The back loop

While in G11_7 mutant, genes involved in the amino acid catabolism in mitochondria were enhanced. The overflow free amino acids and catabolized proteins were channelled into AcCoA. This provides an additional source of AcCoA for fatty acid synthesis (Figure 6, route 5, 6). The recycling of amino acids at a moderate level led to a temporal increase in AcCoA concentration, with little compromise on the biomass growth rate (4%). This elevated AcCoA concentration (30%) exceeds the demand (set point) for biomass growth, which shunts the carbon and energy precursors to the fatty acid synthesis route (Figure 6, route 7). Genes responsible for fatty acid synthesis were constantly overexpressed at the mRNA level, and this equilibrium leads to ultimate generation of TAG in the growth phase.

### The pull‐down

In response to the drawn‐down of [AcCoA] for lipogenesis, a pull‐down of carbon flux from photosynthetic process takes effect, leading to the increase in photosynthetic rate (33%), and overexpression of genes participating in photosynthesis and glycolysis. The inclusion of DCA removed the regulation of PDHC, leading to an increase in AcCoA and accumulation in TAG (42%) (Figure 6, route 8). In consistency with our observation, bioengineering manipulation, such as antisense knock‐down of PDK in the diatom *P. tricornutum*, was reported to promote TAG production by 82% (Ma *et al*., 2014).

The further increase in the TAG accumulation in the stationary growth phase in HL under the treatment of DCA (PDHC activated) could be explained by the following: in the stationary phase, as cells no longer drawn‐down AcCoA for growth, the carbon and energy generated from photosynthesis was channelled to TAG production. Moreover, the cell membrane lipid also serves as the source of BCAA, which is degraded and contributes to the AcCoA reservoir in the stationary growth phase.

The proposed ‘three‐step α loop’ model suggests that the rate of lipogenesis in the growth phase is determined by the balance between the carbon/energy supply, biomass synthesis (growth) and amino acid catabolism. When the carbon metabolism is at high gears, a rapid catabolic rate would lead to accumulation of AcCoA and fatty acid synthesis, without compromise on biomass growth. However, an overrun amino acid catabolism would result in reduced growth rate, lower biomass concentration and ultimately lower TAG productivity of the culture. Collectively, TAEP in microalgae could be stimulated by elevated AcCoA level through multiple approaches. Besides aforementioned two major approaches, a balance for fatty acid oxidation is also of great interest to be investigated. To our knowledge, no genetic manipulation has been achieved in promoting TAG production in the growth phase. Collectively, the deliberated investigation provides targets for metabolic engineering of eukaryotic microalgae for efficient lipid production and may inspire novel biofuel production technology based on growth‐phase lipid‐producing oleaginous microalgae as alternative biofuel feedstocks.

## Experimental procedures

### Strains and culture conditions

The algal culture *Dunaliella tertiolecta* strain UTEX LB‐999 was obtained from the UTEX Culture Collection of Algae (University of Texas at Austin, TX). The microalgal cells were cultivated using ATCC‐1174 DA liquid medium (American Type Culture Collection at Manassas, Virginia) containing 0.5M NaCl in flask with shaking at 100 r.p.m. at 25 °C, under 12‐h light/12‐h dark with light intensity of 30 μmol photons m^−2^ s^−1^. Culture was supplied with 2% CO_2_ every 2 days. For the purpose of clarity, work conducted throughout the article is based on biological and technical triplicates unless otherwise stated.

TAG content was measured using quick Nile red assay (Bertozzini *et al*., 2011; Chen *et al*., 2009, 2011; Yao *et al*., 2015). The growth of microalgal cells was monitored by counting cell number and measuring absorbance at OD680 nm. The specific growth rate (*μ*) was calculated from the equation (Wahidin *et al*., 2013):

$$\mu =\frac{\ln\;(N2-N1)}{t2-t1}$$

where *N*2 and *N*1 are the cell number concentration measured at times *t*2 and *t*1, respectively. The specific growth rate of the mutant was traced and presented in Figure S6.

### Mutant isolation and optimization

*Dunaliella tertiolecta* transformation and random mutagenesis was conducted via electroporation (Shimogawara *et al*., 1998) (double consecutive pulses were delivered at intervals of 10–15 s in a Bio‐Rad Gene Pulser Xcell^™^ Electroporation Systems, with capacitance = 500 μFD, resistance = 400 Ω, voltage = 400 V), and 500 μL of cells was incubated with 50 μL of linearized plasmid (1 μg/10 μL, total amount 1–5 μg) and 5 μL of carrier DNA (fish sperm DNA) in 4‐mm gap cuvette. The plasmid with a cassette conferring resistance to zeocin served to generate random insertional mutagenesis (Yao *et al*., 2015). The Nile red data showed a relatively good correlation with data from total fatty acid methyl esters analysed by gas chromatography mass spectrometry (GC–MS) (*R* ^2^ = 0.97 for G11_7, *R* ^2^ = 0.92 for WT, Figure S7), indicating such an assay could be used as a quick high‐throughput screening method for TAG‐overproducing mutant strains, which was also tested and suggested by Xu *et al*. (2013).

Flow cytometric analyses of *Dunaliella* strains were performed on Nile red‐stained cells on Beckman Coulter CyAN. High‐TAG sorting strategy was applied to enable enrichment of high‐TAG mutant (0.G11) (5th day of cultivation), by two rounds of FACS on pooled mutants stained with Nile red using Beckman Coulter Mo‐Flo Legacy Cell Sorter (Terashima *et al*., 2015). The same strain treated with acetone was used as the background. The distribution of an identically treated culture of WT was used as the control to determine the TAG gates for mutant pool sorting. A high‐TAG gate that captured about 1.91% of WT with maximal Nile red fluorescence signal for a given chlorophyll fluorescence signal was performed (Figure S2a). The same gate captured about 18.99% of the cells in the 0.G11 mutant cells (Figure S2b). All cells that fell into this gate were collected into vials containing 0.5 mL 0.5 M NaCl ATCC medium. The cells were spun down at 750× ***g*** for 5 min, resuspended in fresh 0.5M ATCC medium, and plated onto 0.5 M ATCC 1.5% agar plates and incubated for 2 weeks. The colonies were subsequently transferred to a flask for subculturing. Mutant pool (1.G11) and WT pool on day 5 were collected and used for second round of FACS. The second cell sorting on 1.G11 mutant and WT pools was carried out using the same amount of cells at a high‐TAG gate (Figure S2c) and captured 11.07% of the cells in the mutant pool (Figure S2d). A wider Nile red and chlorophyll signal distribution in the pool of mutants indicates the presence of mutants that accumulate higher and lower amounts of TAG and chlorophyll. All cells that fell into this gate were collected into 96‐well plates containing 0.5 m NaCl ATCC media and incubated under the same culture condition. The cells from each well were then transferred to flasks for further TAG analysis. Kinetic studies of TAG accumulation in mutants and WT *D. tertiolecta* were carried out using Nile red quantitative assay together with GC‐MS technique (Yao *et al*., 2015). On culture day 6, G11_7 mutant and WT were harvested to visualize the LPs using fluorescent microscopy (Olympus BX63, Tokyo, Japan) and measure the photosynthetic rate using an oxygen electrode according to the operating manual (Rank Brothers, Bottisham, Cambridge, UK).

### RNA extraction and cDNA preparation

*Dunaliella tertiolecta* was collected for total RNA extraction using an RNeasy plant mini kit (Qiagen, Valencia, CA), according to the manufacturer's instructions. DNase was added to eliminate genomic DNA contamination. For quantitative real‐time PCR, total RNA was used to synthesize random hexamer‐primed cDNA according to the manufacturer's instructions.

### Next‐generation sequencing analysis

RNA was extracted from G11_7 and WT on culture day 6 in their exponential growth phase (LL). The quality was verified using Agilent RNA 6000 Nano Kit in Agilent 2100 bioanalyzer (Agilent Technologies, Palo Alto, CA) and gel electrophoresis. The samples were linearized with 0.1N NaOH into single‐stranded forms, and they were then neutralized and diluted into 200pM loading concentration with Examp master mix (EPX1 to 3) and loaded into one lane of paired‐end flowcell using the Illumina cBOT machine. The DNA was attached and amplified simultaneously inside each oligo well on the flowcell surfaces, as a proprietary clustering method known as exclusion amplification. This method ensures that only a single DNA template binds and forms a cluster within a single well, reducing the occurrence of polyclonal wells thus increasing the usable reads. The sequencing primer was then attached to the reads, preparing for sequencing run. The flowcell was then loaded into the Illumina HISEQ4000 High Sequencers with the sequencing reagents and run at 2 × 151 cycles, and the second read turnaround was carried out using the sequencers after the first read was completed. The images were captured by the HiSeq Control Software (HCS), and the Real Time Analysis (RTA) software converted the images into Cycle Intensity Files (CIF) and later Basecall (bcl) files. All the bcl files were then transferred to the server for storage and primary analysis. In the primary analysis, the bcl files were converted into fastq files using the bcl2fastq pipeline. After the conversion, the fastq reads were filtered to remove all the reads that did not pass filtering, leaving only useable passed filtered (PF) reads. The usable reads were then analysed and bin to each of the barcode file known as demultiplexing, and those that did not pass the filtering are not demultiplexed. The primary analysis result was then generated as the demultiplexed report and reviewed. The paired‐end raw data (150 bps in length/read) of G11_7 and WT were trimmed of adaptors and examined with pre‐alignment QA/QC (trimmed from both ends based on the parameter setting: Min read length = 25; Quality encoding = Auto detect; End min quality level (Phred)  = 20) (Table S2) in Partek^®^ Flow^®^ software (version 4.0, Partek Inc., St. Louis, MO) with Dt_v10 *D. tertiolecta* transcriptome database (available on author's website: https://github.com/SPURc-Lab) as the reference using reads per kilobase of transcript per million mapped reads (RPKM). Subsequently, the different expressed transcripts were imported into Partek^®^ Genomics Suite^®^ software (version 6.6, Partek Inc.) for gene annotation and KEGG pathway analysis (Yao *et al*., 2015). We also used an in‐house pipeline to analyse the RNA‐Seq data. The transcriptome database was enlarged via Trinity assembler (Grabherr *et al*., 2011) and BLASTX against reference protein sequences from all plants and bacterial from National Center for Biotechnology Information (NCBI; Shin *et al*., 2015). Transcript level comparison was performed using RSEM (Li and Dewey, 2011) with default settings. The count data from RSEM were imported for normalization in the Ebseq pipeline (Leng *et al*., 2013), which was used for differential expression analysis, with the design matrix formulated to fit the experimental conditions. National Supercomputing Centre (NSCC) was used for running the aforementioned software. The sequencing data were deposited into GEO with accession number of GSE82121.

### DCA treatment and amino acid spike

In the DCA‐treated experiment, WT *D. tertiolecta* with spike of 750 μm DCA (Sodium dichloroacetate, 98%, ACROS Organics^™^) and its net control were cultured under low‐light condition as aforementioned. TAG content was measured using quick Nile red assay as aforementioned. *PDK* mRNA levels were measured using quantitative real‐time PCR under different light conditions. AcCoA levels were quantified using Acetyl‐Coenzyme A Assay Kit (Sigma, MAK039). We did the same on G11_7 mutant both under low‐light (30 μmol photons m^−2^ s^−1^) and high‐light conditions (320 μmol photons m^−2^ s^−1^), respectively, with a net G11_7 strain as the control.

In the amino acid spike experiment, different concentrations of leucine (20, 50, 500, 1000 μm) were spiked into the microalgal culture medium of WT and G11_7 mutant *D. tertiolecta*, with its medium as blank control. Their TAG amount was quantified by the quick Nile red assay.

### Cloning and analysis of important genes

The 5′ and 3′ ends of the *D. tertiolecta* templates were cloned using a SMART^™^ RACE cDNA amplification kit (Clontech, Mountain View, CA) based on the total RNA extracted aforementioned. The full lengths of the coding region of putative *DtCuAO*,* DtIVD*,* DtMCCB* (detected in both next‐generation sequencing analyses) genes were amplified from *D. tertiolecta* cDNA by RACE PCR via the predicted region from the *D. tertiolecta* database. All the primers used in this study were listed in Table S3. Phylogenetic tree of protein clusters from various species was constructed by neighbor‐joining (NJ) method using software MEGA 5 [20]. Subcellular localization of the related genes was predicted by SignalP 4.1 (http://www.cbs.dtu.dk/services/SignalP/), ChloroP (http://www.cbs.dtu.dk/services/ChloroP/), MITOPROT (https://ihg.gsf.de/ihg/mitoprot.html), Hectar (http://webtools.sb-roscoff.fr) online.

### Metabolome analysis

Two hundred millilitre algal cells of G11_7 and WT were collected on day 6, respectively, and freeze‐dried for metabolome analysis. Metabolite extraction method was first optimized using a test sample. Four solvent mixtures were tested, and the optimal condition was chosen to ensure the maximum number of peak pairs that could be detected.

Metabolome profiles of the two genotypes of microalgae with biological triplicates were analysed using chemical isotope labelling (CIL) LC‐MS method. The CIL LC‐MS analysis focused on amine/phenol compounds and carboxylic acid submetabolomes extracted from the samples. Three aliquots of each sample were weighed out, and each aliquot was labelled according to protocols reported previously (Guo and Li, 2009, 2010; Peng and Li, 2013). Samples were then mixed appropriately and analysed on the Bruker Impact HD quadrupole time‐of‐flight (Q‐TOF) mass spectrometer (Bruker, Billerica, MA) connected to a Dionex Ultimate 3000 (Dionex, Sunnyvale, CA). The samples were injected onto an Agilent reversed‐phase Eclipse Plus C18 column (2.1 mm × 10 cm, 1.8 μm, 95 Å) for separation. Solvent A was 0.1% (v/v) formic acid/5% (v/v) ACN /H_2_O, and solvent B was 0.1% (v/v) formic acid/ACN. The chromatographic conditions for dansyl labelling were as follows: *t* = 0 min, 20% B; *t* = 3.5 min, 35% B; *t* = 18 min, 65% B; *t* = 21 min, 95% B; *t* = 26 min, 95% B. The gradient for DmPA labelling was *t* = 0 min, 20% B; *t* = 9 min, 50% B; *t* = 22 min, 65% B; *t* = 26 min, 80% B; *t* = 29 min, 98% B; *t* = 40 min, 98% B. Column temperature was set at 30 °C, and a flow rate of 180 μL/min was applied. All MS spectra were obtained in the positive ion mode. The mass range was set as 220–1000 m/z for danzyl labelling and 110–1000 m/z for acid labelling. The MS spectral rate was 1.0 Hz.

For targeted acyl‐CoA analysis, LC‐MS with standard addition method was used to quantify the CoAs in the algae samples (Friis *et al*., 2014). A mixture of fifteen CoA standards was used. 30 μL of each biological triplicate sample was injected onto a Kinetex Coreshell HILIC column (100 mm × 2.1 mm, 1.7 μm) for separation. LC‐MS analysis was carried out using a Dionex Ultimate 3000 (Dionex) connected to a Bruker maXis II Q‐TOF instrument (Bruker). Column temperature was set at 40 °C, and a solvent gradient of 5 min at 500 μL/min was used with an equilibration of 8 min at 600 μL/min. Solvent A was 10 mm ammonium acetate (pH 5.6), and solvent B was 95% ACN/5% 10 mm ammonium acetate (pH 5.6). The gradient was as follows: 0–3.0 min (90% B‐5% B); 3.0–5.5 min (5% B‐5% B). For the Q‐TOF instrument, the mass range was set as 750–1100 m/z and the spectral acquisition rate was 3 Hz. All MS spectra were obtained in the positive ion mode. Peak areas of all the CoAs were extracted using Bruker Target Analysis software for quantification.

Profiling the amine‐ and phenol‐containing metabolites (i.e. the amine/phenol submetabolome) was carried out using differential chemical isotope labelling liquid chromatography mass spectrometry (CIL LC‐MS) (Figure S8). A total of 2246 metabolites (isotope peak pairs) were detected in the 18 LC‐MS runs. Among those metabolites, 276 were considered significant contributors to the differentiation of the two genotypes from Volcano Plot, and the two groups of samples can be well separated through either principal component analysis (PCA) or partial least squares discriminant analysis (PLS‐DA) (Figure S9a‐c). Among those 276 metabolites, 18 were positively identified against dansyl standards library based on both mass and retention time matches; 46 could be putatively identified using MyCompoundID search engine against HMDB based on 0 reaction and Metlin. The detailed amine‐ and phenol‐containing metabolite report is shown in Supplementary Data Set 2a.

In the carboxylic acid submetabolome profiling, 2246 metabolites (isotope peak pairs) were detected in 18 LC‐MS runs. Among those metabolites, 142 were considered significant contributors to the differentiation of the two genotypes (Figure S9d‐f). Separation of the two groups of samples can be very well observed in PCA and PLS‐DA plots. Fold changes for some of the targeted acids are listed in Supplementary Data Set 2b.

Importantly, fifteen targeted acyl‐CoAs with different acyl groups were also performed using LC‐MS, and their fold changes between the two groups, along with CoA concentrations, are shown in Supplementary Data Set 2c. Our emphases were mainly in the fatty acid biosynthesis pathway, protein metabolic pathway, citrate acid cycle (TCA cycle) and their precursors.

## Conflict of interest

The authors declare that they have no conflict of interest.

## Supporting information


**Figure S1**. Physiological study of WT *D. tertiolecta* after DCA treatment. (a) TAG quantitative assay by Nile red staining method under low‐light condition. (b) Growth curve monitored by spectrophotometry under low‐light condition. (c) Acetyl‐CoA level change after addition of DCA to WT. (LL: 30 μmol photons m^−2^ s^−1^). Error bars, SEM. Statistical analyses were performed using Student t test, ***P < 0.001.
**Figure S2**. Fluorescence‐activated cell sorting for mutant and WT *D. tertiolecta*. (a) Fluorescent cell sorting image from first sorted WT cells (R3). (b) Fluorescent cell sorting image from first sorted G11 cells (R3). (c) Fluorescent cell sorting image from second sorted WT cells (R6). (d) Fluorescent cell sorting image from second sorted G11 cells (R6). (e) Fast screening of *D. tertiolecta* colonies from FACS using Nile red staining assay. (f) Detailed screening of *D. tertiolecta* colonies with top TAG accumulation ability in biological triplicate using Nile red staining assay.
**Figure S3**. Fold change of G11_7 and WT fatty acid profile. (a) Fatty acid content is expressed as percentage of total fatty acids of G11_7 and WT (n = 3). (b) *Satd* saturated fatty acids, *Mounsatd* monounsaturated fatty acids, *Pounsatd* polyunsaturated fatty acids, *Unsatd* unsaturated fatty acids, *DUS* the degree of fatty acid unsaturation =  [1.0× (% monoenes) + 2.0× (% dienes) + 3.0× (% trienes) + 4.0× (% tetraenes)]/100. Error bars, SEM. Statistical analyses were performed using Student t test, *P < 0.05, **P < 0.01, ***P < 0.001.
**Figure S4**. Evolutionary relationships of taxa of 3 predicted genes. The evolutionary history was inferred using the Neighbor‐Joining method [1]. (a) *DtCuAO* gene, the optimal tree with the sum of branch length = 32.94110683 is shown. There were a total of 415 positions in the final dataset. (b) *DtIVD* gene, the optimal tree with the sum of branch length = 28.60417585 is shown. There were a total of 294 positions in the final dataset. (c) *DtMCCB* gene, the optimal tree with the sum of branch length = 27.00169906 is shown. There were a total of 156 positions in the final dataset. The percentage of replicate trees in which the associated taxa clustered together in the bootstrap test (1000 replicates) are shown next to the branches [2]. The tree is drawn to scale, with branch lengths in the same units as those of the evolutionary distances used to infer the phylogenetic tree. The evolutionary distances were computed using the Poisson correction method [3] and are in the units of the number of amino acid substitutions per site. The analysis involved 32 amino acid sequences. All positions containing gaps and missing data were eliminated. Evolutionary analyses were conducted in MEGA5 [4].
**Figure S5**. Temporal expression of predicted genes and lipid accumulation fold changes. (a) Fold change in abundance of *DtIVD* and *DtCuAO* transcript (primary axis) during lipid accumulation in G11_7/WT *D. tertiolecta* (secondary axis). (b) Relative *DtIVD*,* DtCuAO*, and *DtMCCB* mRNA transcript abundance (primary axis) during lipid accumulation (secondary axis) in different *D. tertiolecta* strains.
**Figure S6**. Specific growth rate of G11_7 mutant versus WT *Dunaliella tertiolecta* under low light.
**Figure S7**. Linear regression of Nile red assay and GC‐MS measurement. (a) and (b), Horizontal error bars show standard deviation for Nile red assay. Vertical error bars show standard deviation for total fatty acid from GC‐MS measurement. The line shows linear regression between the two methods. Strain genotypes: 1, 2, 3, 4, 5 represent culture day 6, 8, 16, 28, 33 for G11_7 strain in a), respectively; 1, 2, 3, 4, 5 represent culture day 6, 8, 16, 28, 33 for WT strain in b). All experiments were performed in triplicate.
**Figure S8**. Experimental workflow of isotopic labeling LC‐MS for quantifying the changes of metabolites in the WT and G11‐7 mutant *D. tertiolecta*.
**Figure S9**. Volcano Plot, and PCA and PLS‐DA score plot. (a) Volcano plot of amine/phenol analysis. 276 metabolites has Fold Change (mutated/WT) >1.5, *P* < 0.01, with 100 upregulated and 176 downregulated. (b) PCA plot of amine/phenol analysis. (c) PLS‐DA score plot of amine/phenol analysis, R^2^ = 0.997, Q^2^ = 0.96. (d) Volcano Plot of global carboxylic acids profiling. 303 metabolites has Fold Change (mutated/WT) >1.5, *P* < 0.05; 248 up‐regulated and 55 down‐regulated. (e) PCA plot of global carboxylic acids profiling. (f) PLS‐DA score plot of global carboxylic acids profiling.
**Table S1**. Important genes and metabolites affected in the mutant strain G11_7.
**Table S2**. Run summary of G11_7 and WT *D. tertiolecta* on the Illumina HISEQ4000 platform.
**Table S3**. Primer sequence for RACE PCR and real‐time PCR.
**Data Set S1**. Differential expressed transcript list from next‐generation sequencing. (a) Differential expressed genes detected by Partek workflow. (b) Differential expressed genes detected by in‐house workflow.
**Data Set S2**. Specific metabolite list. (a) Profiling the amine‐ and phenol‐containing metabolites. (b) Profiling the Carboxylic Acids. (c) Fold changes of the targeted CoAs.
**Data Set S3**. cDNA sequence of the studied genes and their corresponding sequence of translated amino acid. (a) DtCuAO, (b) DtIVD, (c) DtMCCB, and (d) DtPDK.
