# Supplementary material for: Elevated acetyl‐CoA by amino acid recycling fuels microalgal neutral lipid accumulation in exponential growth phase for biofuel production
Source: Plant Biotechnol J. 2016 Nov 8;15(4):497–509. doi: 10.1111/pbi.12648 (PMC5362678; doi:10.1111/pbi.12648)
Supplement: Supplementary file 1 — Figure S1. Physiological study of WT D. tertiolecta after DCA treatment. (a) TAG quantitative assay by Nile red staining method under low‐light condition. (b) Growth curve monitored by spectrophotometry under low‐light condition. (c) Acetyl‐CoA level change after addition of DCA to WT. (LL: 30 μmol photons m−2 s−1). Error bars, SEM. Statistical analyses were performed using Student t test, ***P < 0.001. Figure S2. Fluorescence‐activated cell sorting for mutant and WT D. tertiolecta. (a) Fluorescent cell sorting image from first sorted WT cells (R3). (b) Fluorescent cell sorting image from first sorted G11 cells (R3). (c) Fluorescent cell sorting image from second sorted WT cells (R6). (d) Fluorescent cell sorting image from second sorted G11 cells (R6). (e) Fast screening of D. tertiolecta colonies from FACS using Nile red staining assay. (f) Detailed screening of D. tertiolecta colonies with top TAG accumulation ability in biological triplicate using Nile red staining assay. Figure S3. Fold change of G11_7 and WT fatty acid profile. (a) Fatty acid content is expressed as percentage of total fatty acids of G11_7 and WT (n = 3). (b) Satd saturated fatty acids, Mounsatd monounsaturated fatty acids, Pounsatd polyunsaturated fatty acids, Unsatd unsaturated fatty acids, DUS the degree of fatty acid unsaturation = [1.0× (% monoenes) + 2.0× (% dienes) + 3.0× (% trienes) + 4.0× (% tetraenes)]/100. Error bars, SEM. Statistical analyses were performed using Student t test, *P < 0.05, **P < 0.01, ***P < 0.001. Figure S4. Evolutionary relationships of taxa of 3 predicted genes. The evolutionary history was inferred using the Neighbor‐Joining method [1]. (a) DtCuAO gene, the optimal tree with the sum of branch length = 32.94110683 is shown. There were a total of 415 positions in the final dataset. (b) DtIVD gene, the optimal tree with the sum of branch length = 28.60417585 is shown. There were a total of 294 positions in the final dataset. (c) DtMCCB gene, the optimal tree with [file PBI-15-497-s001.docx]

# Supplementary Materials

**Supplementary Figures**

a

b

c

**Supplementary Figure 1** Physiological study of WT *D. tertiolecta* after DCA treatment.

(a) TAG quantitative assay by Nile red staining method under low light condition. (b) Growth curve monitored by spectrophotometry under low light condition. (c) Acetyl-CoA level change after addition of DCA to WT. (LL: 30 μmol photons•m^-2^•s^-1^). Error bars, SEM. Statistical analyses were performed using Student t test, ***P < 0.001.

a b

c d

e

f

**Supplementary Figure 2** Fluorescence-activated cell sorting for mutant and WT *D. tertiolecta*.

(a) Fluorescent cell sorting image from first sorted WT cells (R3). (b) Fluorescent cell sorting image from first sorted G11 cells (R3). (c) Fluorescent cell sorting image from second sorted WT cells (R6). (d) Fluorescent cell sorting image from second sorted G11 cells (R6). (e) Fast screening of *D. tertiolecta* colonies from FACS using Nile red staining assay. (f) Detailed screening of *D. tertiolecta* colonies with top TAG accumulation ability in biological triplicate using Nile red staining assay.

a

b

**Supplementary Figure 3** Fold change of G11_7 and WT fatty acid profile.

(a) Fatty acid content is expressed as percentage of total fatty acids of G11_7 and WT (n = 3). (b) *Satd* saturated fatty acids, *Mounsatd* monounsaturated fatty acids, *Pounsatd* polyunsaturated fatty acids, *Unsatd* unsaturated fatty acids, *DUS* the degree of fatty acid unsaturation = [1.0 × (% monoenes) + 2.0 × (% dienes) + 3.0 × (% trienes) + 4.0 × (% tetraenes)]/100.

Error bars, SEM. Statistical analyses were performed using Student t test, *P < 0.05, **P < 0.01, ***P < 0.001.

a
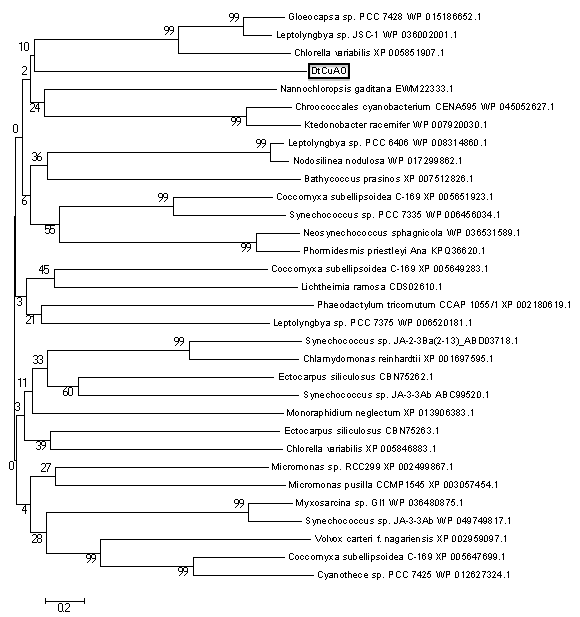


b

c

**Supplementary Figure 4** Evolutionary relationships of taxa of 3 predicted genes.

The evolutionary history was inferred using the Neighbor-Joining method (Saitou and Nei, 1987). (a) *DtCuAO* gene, the optimal tree with the sum of branch length = 32.94110683 is shown. There were a total of 415 positions in the final dataset. (b) *DtIVD* gene, the optimal tree with the sum of branch length = 28.60417585 is shown. There were a total of 294 positions in the final dataset. (c) *DtMCCB* gene, the optimal tree with the sum of branch length = 27.00169906 is shown. There were a total of 156 positions in the final dataset. The percentage of replicate trees in which the associated taxa clustered together in the bootstrap test (1000 replicates) are shown next to the branches (Felsenstein, 1985). The tree is drawn to scale, with branch lengths in the same units as those of the evolutionary distances used to infer the phylogenetic tree. The evolutionary distances were computed using the Poisson correction method (Zuckerkandl and Pauling, 1965) and are in the units of the number of amino acid substitutions per site. The analysis involved 32 amino acid sequences. All positions containing gaps and missing data were eliminated. Evolutionary analyses were conducted in MEGA5 (Tamura et al., 2011).

Disclaimer: Although utmost care has been taken to ensure the correctness of the caption, the caption text is provided "as is" without any warranty of any kind. Authors advise the user to carefully check the caption prior to its use for any purpose and report any errors or problems to the authors immediately (www.megasoftware.net). In no event shall the authors and their employers be liable for any damages, including but not limited to special, consequential, or other damages. Authors specifically disclaim all other warranties expressed or implied, including but not limited to the determination of suitability of this caption text for a specific purpose, use, or application.

a

b

**Supplementary Figure 5** Temporal expression of predicted genes and lipid accumulation fold changes.

(a) Fold change in abundance of *DtIVD* and *DtCuAO* transcript (primary axis) during lipid accumulation in G11_7/WT *D. tertiolecta* (secondary axis). (b) Relative *DtIVD*, *DtCuAO*, and *DtMCCB* mRNA transcript abundance (primary axis) during lipid accumulation (secondary axis) in different *D. tertiolecta* strains.

**Supplementary Figure 6** Specific growth rate of G11_7 mutant versus WT *Dunaliella tertiolecta* under low light.

a

b

**Supplementary Figure 7** Linear regression of Nile red assay and GC-MS measurement.

(a) and (b), Horizontal error bars show standard deviation for Nile red assay. Vertical error bars show standard deviation for total fatty acid from GC-MS measurement. The line shows linear regression between the two methods. Strain genotypes: 1, 2, 3, 4, 5 represent culture day 6, 8, 16, 28, 33 for G11_7 strain in a), respectively; 1, 2, 3, 4, 5 represent culture day 6, 8, 16, 28, 33 for WT strain in b). All experiments were performed in triplicate.


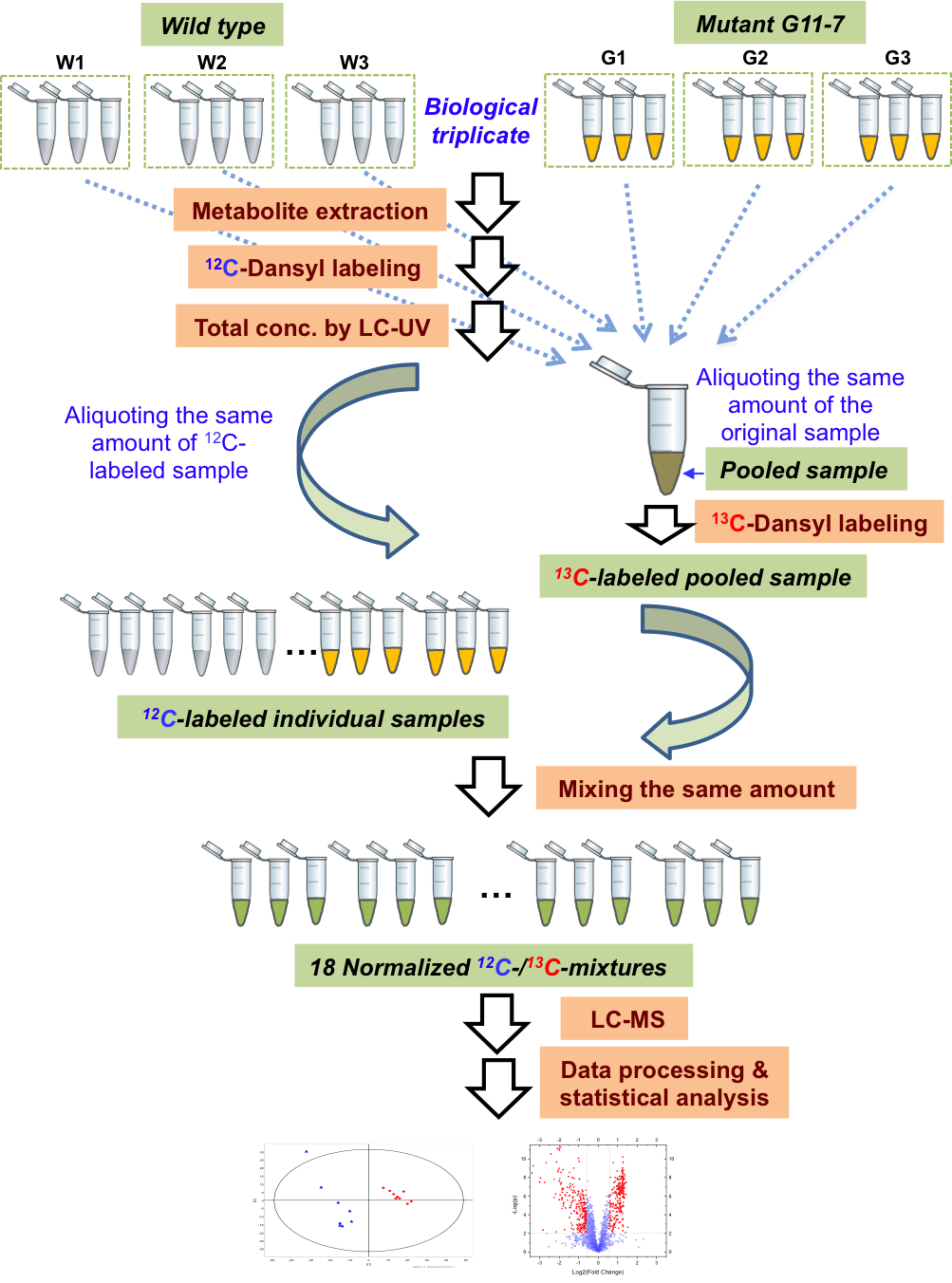


**Supplementary Figure 8** Experimental workflow of isotopic labeling LC-MS for quantifying the changes of metabolites in the WT and G11-7 mutant *D. tertiolecta*.

a

b

c

d

e

f

**Supplementary Figure 9** Volcano Plot, and PCA and PLS-DA score plot.

(a) Volcano plot of amine/phenol analysis. 276 metabolites has Fold Change (mutated/WT) > 1.5, p <0.01, with 100 upregulated and 176 downregulated. (b) PCA plot of amine/phenol analysis. (c) PLS-DA score plot of amine/phenol analysis, R^2^ = 0.997, Q^2^ = 0.96. (d) Volcano Plot of global carboxylic acids profiling. 303 metabolites has Fold Change (mutated/WT) > 1.5, p <0.05; 248 up-regulated and 55 down-regulated. (e) PCA plot of global carboxylic acids profiling. (f) PLS-DA score plot of global carboxylic acids profiling. R^2^ = 0.995, Q^2^ = 0.949.

**Supplementary Tables**

Supplementary Table 1 Important genes and metabolites affected in the mutant strain G11_7.

| **Metabolite Names** | **Fold Change**  **(G11_7/ WT)** | **p-value** | **Participating pathways** | **Gene Names** | **Fold Change**  **(G11_7/ WT)** | **FDR** |
| --- | --- | --- | --- | --- | --- | --- |
| L-Glutamic Acid | 1.59 | 7.18E-07 | Valine, leucine and isoleucine degradation & Transamination | isovaleryl-CoA-dehydrogenase  (IVD) | 4.93  */ 9.37* | 3.3E-02  */ 0* |
| n-Propionyl-CoA | 1.7 | 2.3E-03 |  | 3-methylcrotonyl-CoA carboxylase  (MCCB) | 4.87  */ 6.96* | 2.5E-02  */ -* |
|  |  |  |  | acetyl-CoA C-acetyltransferase  (ACCA) | *111.609* | *0* |
|  |  |  |  | alanine-glyoxylate transaminase / (R)-3-amino-2-methylpropionate-pyruvate transaminase | *0.0115924* | *7.38E-08* |
| Glutaryl-CoA | 1.8 | 5.6E-03 | Lysine degradation | acetyl-CoA C-acetyltransferase  (ACCA) | *111.609* | *0* |
| Butyryl-CoA | 1.3 | 1.4E-02 |  | histone-lysine N-methyltransferase (SETD2) | *0.0560417* | *2.83E-02* |
| Pyruvate | 1.55 | 1.75E-02 | * |  |  |  |
| Acetyl-CoA | 1.3 | 3.1E-05 | * |  |  |  |
|  |  |  |  |  |  |  |
| Citrate | 2.12 | 2.6E-04 | TCA cycle |  |  |  |
| Succinyl-CoA | 5.9 | 4.3E-04 |  |  |  |  |
| Malonyl-CoA | 2.1 | 4.9E-03 | Fatty acid biosynthesis & elongation & Glycerolipid metabolism | malonyl-CoA:acyl-carrier-protein transacylase  (FabD) | *5.67* | *1.0E-04* |
| Myristoyl-CoA (14:0-CoA) | 0.1 | 1.2E-04 |  | glycerol kinase  (GLPK) | *2.35463* | *-* |
| Palmitoleoyl-CoA (16:1-CoA) | 0.3 | 1.0E-03 |  | 2-acylglycerol O-acyltransferase 2  (MOGAT) | *1.5837* | *-* |
| Oleoyl-CoA (18:1-CoA) | 0.5 | 1.3E-02 |  | phospholipid:diacylglycerol acyltransferase | *0.00880818* | *0* |
| Arachidonyl CoA (20:4-CoA) | 0.3 | 4.6E-03 |  |  |  |  |
| L-Proline | 1.52 | 7.05E-05 | Arginine and proline metabolism & β-Alanine & Transamination | copper amine oxidase family protein  (AMX1) | 7.59  */ 10.95* | 1.3E-02  / - |
| 1-Pyrroline-3-hydroxy-5-carboxylate | 1.59 | 7.18E-07 |  |  |  |  |
| Ornithine | -2.26 | 2.88E-06 |  |  |  |  |
| Spermidine | -1.70 | 1.99E-06 |  |  |  |  |
|  |  |  | Photosynthesis | plastocyanin (petE) | *22.4279* | *2.39E-07* |
|  |  |  |  | cytochrome b6-f complex iron-sulfur subunit (petC) | *2.35716* | *1.02E-03* |
|  |  |  |  | ferredoxin (petF) | *1.548* | *-* |
|  |  |  |  | photosystem II 22kDa protein (psbS) | *0.0143979* | *1.75E-09* |
|  |  |  |  | light-harvesting complex I chlorophyll a/b binding protein 1 (LHCA1) | *40.9366* | *1.05E-02* |
|  |  |  |  | light-harvesting complex II chlorophyll a/b binding protein 1 (LHCB1) | *4.4559* | *-* |
|  |  |  | Glycolysis / Gluconeogenesis | 6-phosphofructokinase 1 (pfkA) | *8.3757* | *-* |
|  |  |  |  | phosphoenolpyruvate carboxykinase (ATP) (PPC) | *21.3294* | *1.10E-09* |
|  |  |  |  | fructose-1,6-bisphosphatase I (FBP) | *43.363* | *-* |

The changes in gene expression level found from in-house pipeline are shown in italic with underline. Changes found from Partek pipeline are shown in normal font.

* Pyruvate and acetyl-CoA are core intermediates participating in many pathways.

- No FDR is provided for specific genes since multiple contigs are detected.

Supplementary Table 2 Run summary of G11_7 and WT *D. tertiolecta* on the Illumina HISEQ4000 platform.

| **Sample name** | **Sequencing stats and pre-alignment QA/QC of raw data**  **/pre-alignment QA/QC of post-trimming data** | | | | | **Post-alignment QA/QC after alignment** | |
| --- | --- | --- | --- | --- | --- | --- | --- |
|  | Total reads | Avg. read length | Avg. read quality | % N | % GC | Total reads | % Aligned |
| G11_7-1 | 131,836,866/ **131,836,590** | 150.00/ **149.80** | 38.78/ **38.81** | .03/ **.02** | 55.40/ **55.40** | 65,918,295 | 65.13 |
| G11_7-2 | 175,867,666/ **175,867,448** | 150.00/ **149.80** | 38.82/ **38.85** | .03/ **.02** | 55.16/ **55.16** | 87,933,724 | 63.65 |
| G11_7-3 | 129,743,218/ **129,742,886** | 150.00/ **149.79** | 38.73/ **38.76** | .03/ **.02** | 55.00/ **54.99** | 64,871,443 | 65.75 |
| WT-1 | 135,893,898/ **135,893,720** | 150.00/ **149.82** | 38.92/ **38.95** | .03/ **.02** | 55.35/ **55.34** | 67,946,860 | 63.47 |
| WT-2 | 110,407,944/ **110,407,800** | 150.00/ **149.81** | 38.89/ **38.92** | .03/ **.02** | 54.83/ **54.83** | 55,203,900 | 63.79 |
| WT-3 | 123,035,916/ **123,035,586** | 150.00/ **149.81** | 38.87/ **38.90** | .03/ **.02** | 54.54/ **54.54** | 61,517,793 | 66.31 |

Supplementary Table 3 Primer sequence for RACE PCR and real-time PCR.

| **Primer name** | **Primer Sequence** |  |
| --- | --- | --- |
|
| **RACE or sequence confirmation primers** | |  |
| r_DtCuAO_R1 | TCACATTGTTGCCGCAGACAT | RACE PCR for 5' end |
| r_DtCuAO_R2 | ACACGAAAATCTGCATGACCT | RACE PCR for 5' end (nested) |
| r_DtCuAO_F1 | AATGCGTTCTATGCAAAGGAGCA | RACE PCR for 3' end |
| r_DtCuAO_F2 | TGTCATGCCTGTGGAGACTGT | RACE PCR for 3' end (nested) |
| Dtmccb_F1 | ATGGGTGCGGTGCTTTGGGCT |  |
| Dtmccb_R2 | ACAGGGAACACTGTTAGCAAAG |  |
| rDtACAD_R1 | TCATGCCTAGTTTGTTGAGCT | RACE PCR for 5' end |
| rDtACAD_R2 | ATTGGTGCACCACATTTTGG | RACE PCR for 5' end (nested) |
| rDtACAD_F1 | CCAAAATGTGGTGCACCAAT | RACE PCR for 3' end |
| rDtACAD_F2 | AGCTCAACAAACTAGGCATGA | RACE PCR for 3' end (nested) |
| **qPCR primers** |  |  |
| qDtCuAO_F1 | CATTGGCATACGGATTCTTG |  |
| qDtCuAO_R1 | GTCCGTATTGATCCGGCTAT |  |
| qDtIVD_F1 | TCTGTGCTGTGCTGAAACC |  |
| qDtIVD_R1 | GGCAAGACAGAGCCTGAAA |  |
| qPDK_F | ATGAGCCTCCCAAAGATGAC |  |
| qPDK_R | CGCGTAAGCATATCGACCTA |  |
| DtTUB_F | CAGATGTGGGATGCCAAGAACAT |  |
| DtTUB_R | GTTCAGCATCTGCTC ATCCACCT |  |

**Supplementary Data Sets**

Supplementary Data Set 1 Differential expressed transcript list from next-generation sequencing.

(a) Differential expressed genes detected by Partek workflow.

| **Dt_name** | **Chlre4_ID** | **E_Value** | **proteinName** | **FDR Step Up** | **MultiModel Fold-Change** |
| --- | --- | --- | --- | --- | --- |
| Locus_2504_7Transcript_2/15_Confidence_1.000_Length_148 | Cre33.g782350.t1.2 | 4.00E-13 | DNA/RNA polymerases superfamily protein | 2.75E-04 | -56086.1 |
| Locus_1063_8Transcript_3/4_Confidence_0.636_Length_2786 | Cre33.g782350.t1.2 | 7.00E-107 | DNA/RNA polymerases superfamily protein | 3.32E-04 | -43822.5 |
| Locus_31653_7Transcript_1/1_Confidence_1.000_Length_299 | Cre02.g128758.t1.1 | 1.00E-13 | NA | 1.30E-03 | -23264 |
| Locus_34012_8Transcript_1/1_Confidence_1.000_Length_218 | Cre14.g618250.t1.2 | 6.00E-10 | NA | 9.38E-04 | -10996.9 |
| Locus_34904_9Transcript_1/1_Confidence_1.000_Length_201 | Cre02.g135300.t1.1 | 3.00E-07 | methyl-CPG-binding domain 9 | 2.02E-03 | -8580.39 |
| Locus_11977_5Transcript_1/1_Confidence_1.000_Length_307 | Cre01.g060700.t1.2 | 6.00E-34 | NA | 1.89E-03 | -8148.02 |
| Locus_31476_3Transcript_1/1_Confidence_1.000_Length_315 | Cre12.g528200.t1.2 | 2.00E-07 | DNA replication helicase, putative | 3.83E-03 | -6621.97 |
| Locus_32608_2Transcript_1/1_Confidence_1.000_Length_400 | Cre08.g364000.t1.2 | 6.00E-09 | Transducin family protein / WD-40 repeat family protein | 7.66E-04 | -6614.67 |
| Locus_31495_8Transcript_1/1_Confidence_1.000_Length_162 | Cre09.g390300.t1.2 | 6.00E-07 | Protein kinase superfamily protein with octicosapeptide/Phox/Bem1p domain | 1.53E-02 | -6184.94 |
| Locus_3387_5Transcript_1/1_Confidence_1.000_Length_481 | Cre10.g423050.t1.1 | 4.00E-28 | DNA/RNA polymerases superfamily protein | 6.17E-03 | -5912.42 |
| Locus_53204_9Transcript_1/1_Confidence_1.000_Length_343 | Cre03.g158600.t1.1 | 8.00E-12 | NA | 2.55E-03 | -5674.21 |
| Locus_30464_7Transcript_1/1_Confidence_1.000_Length_178 | Cre12.g496950.t1.2 | 2.00E-09 | WD-40 repeat family protein / notchless protein, putative | 7.68E-03 | -4565.37 |
| Locus_39039_8Transcript_1/1_Confidence_1.000_Length_453 | Cre15.g646200.t1.2 | 2.00E-10 | NA | 4.40E-04 | -4543.68 |
| Locus_30645_7Transcript_1/1_Confidence_1.000_Length_168 | Cre17.g720261.t1.2 | 2.00E-16 | Putative serine esterase family protein | 6.36E-03 | -4192.62 |
| Locus_28826_5Transcript_1/1_Confidence_1.000_Length_356 | Cre06.g274700.t1.2 | 1.00E-14 | OTU-like cysteine protease family protein | 1.59E-03 | -3949.81 |
| Locus_2794_6Transcript_10/20_Confidence_1.000_Length_189 | Cre01.g020850.t1.2 | 6.00E-08 | NA | 5.89E-03 | -3926.04 |
| Locus_41061_8Transcript_1/1_Confidence_1.000_Length_173 | Cre10.g462750.t1.1 | 2.00E-09 | elongator protein 2 | 1.23E-02 | -3822.17 |
| Locus_73847_9Transcript_1/1_Confidence_1.000_Length_218 | Cre09.g398850.t1.1 | 5.00E-08 | tonneau 1b (TON1b) | 9.71E-04 | -3631.7 |
| Locus_9240_3Transcript_1/1_Confidence_1.000_Length_245 | Cre13.g576000.t1.2 | 2.00E-11 | NA | 7.22E-03 | -3138.56 |
| Locus_31869_6Transcript_1/1_Confidence_1.000_Length_225 | Cre02.g146500.t1.2 | 3.00E-08 | yeast YAK1-related gene 1 | 7.14E-03 | -3065.55 |
| Locus_28090_5Transcript_1/1_Confidence_1.000_Length_196 | Cre02.g119651.t1.1 | 1.00E-15 | NA | 1.27E-03 | -2908.28 |
| Locus_51709_10Transcript_1/1_Confidence_1.000_Length_188 | Cre05.g230800.t1.2 | 1.00E-10 | SNF2 domain-containing protein / helicase domain-containing protein / F-box family protein | 1.51E-03 | -2743.9 |
| Locus_11352_5Transcript_1/1_Confidence_1.000_Length_942 | Cre16.g660900.t1.1 | 2.00E-22 | P-loop containing nucleoside triphosphate hydrolases superfamily protein | 1.41E-03 | -2478.45 |
| Locus_11818_3Transcript_1/1_Confidence_1.000_Length_323 | Cre13.g568450.t1.2 | 5.00E-11 | P-loop containing nucleoside triphosphate hydrolases superfamily protein | 2.14E-02 | -2416.82 |
| Locus_17053_5Transcript_1/1_Confidence_1.000_Length_268 | Cre12.g528200.t1.2 | 2.00E-11 | DNA replication helicase, putative | 8.43E-03 | -2103.54 |
| Locus_40520_1Transcript_1/1_Confidence_1.000_Length_202 | Cre15.g642050.t1.1 | 1.00E-08 | NA | 5.79E-04 | -1963.87 |
| Locus_40674_6Transcript_1/1_Confidence_1.000_Length_256 | Cre13.g584450.t1.2 | 8.00E-08 | Leucine-rich repeat transmembrane protein kinase | 4.11E-04 | -1833.69 |
| Locus_27947_1Transcript_3/6_Confidence_0.267_Length_611 | Cre08.g364650.t1.2 | 9.00E-26 | Nucleic acid-binding, OB-fold-like protein | 1.51E-02 | -1805.61 |
| Locus_33763_2Transcript_1/1_Confidence_1.000_Length_428 | Cre06.g290800.t1.2 | 9.00E-45 | S-adenosyl-L-methionine-dependent methyltransferases superfamily protein | 5.08E-05 | -1641.37 |
| Locus_12520_9Transcript_1/1_Confidence_1.000_Length_327 | Cre09.g408450.t1.2 | 2.00E-30 | NA | 9.11E-04 | -1632.01 |
| Locus_208_8Transcript_1/1_Confidence_1.000_Length_527 | Cre05.g243750.t1.2 | 2.00E-07 | NA | 1.10E-03 | -1379.05 |
| Locus_15414_8Transcript_1/1_Confidence_1.000_Length_386 | Cre07.g313500.t1.1 | 8.00E-09 | Nuclear pore complex protein | 6.37E-03 | -1313.86 |
| Locus_9442_1Transcript_1/1_Confidence_1.000_Length_442 | Cre14.g610350.t1.2 | 3.00E-23 | dgd1 suppressor 1 | 2.61E-02 | -1269.34 |
| Locus_39657_9Transcript_1/1_Confidence_1.000_Length_242 | Cre01.g044850.t1.1 | 4.00E-07 | Zinc finger, C3HC4 type (RING finger) family protein | 6.33E-04 | -1164.16 |
| Locus_15804_5Transcript_1/1_Confidence_1.000_Length_275 | Cre13.g602950.t1.2 | 4.00E-07 | NA | 3.85E-04 | -1140.24 |
| Locus_24774_7Transcript_2/2_Confidence_0.889_Length_760 | Cre12.g489600.t1.2 | 3.00E-39 | NA | 1.83E-03 | -1083.69 |
| Locus_3206_5Transcript_2/2_Confidence_0.600_Length_1408 | Cre02.g115450.t1.2 | 5.00E-08 | NA | 4.08E-02 | -1048.54 |
| Locus_1162_3Transcript_6/9_Confidence_0.429_Length_966 | Cre21.g762000.t1.1 | 4.00E-17 | NA | 1.43E-04 | -940.393 |
| Locus_69139_10Transcript_1/1_Confidence_1.000_Length_600 | Cre02.g100800.t1.2 | 1.00E-07 | TBP-associated factor 5 | 1.54E-03 | -881.679 |
| Locus_86325_10Transcript_2/2_Confidence_0.667_Length_411 | Cre10.g457150.t1.1 | 3.00E-16 | NA | 5.60E-03 | -776.754 |
| Locus_15808_1Transcript_1/1_Confidence_1.000_Length_1804 | Cre04.g211950.t1.2 | 9.00E-08 | Exostosin family protein | 8.50E-06 | -538.762 |
| Locus_16315_7Transcript_1/1_Confidence_1.000_Length_1122 | Cre12.g483500.t1.2 | 1.00E-38 | pleckstrin homology (PH) domain-containing protein | 1.12E-02 | -538.434 |
| Locus_6756_9Transcript_1/2_Confidence_0.667_Length_805 | Cre03.g161800.t1.1 | 2.00E-30 | ATPases;nucleotide binding;ATP binding;nucleoside-triphosphatases;transcription factor binding | 7.15E-03 | -452.884 |
| Locus_1290_5Transcript_4/9_Confidence_0.308_Length_1090 | Cre02.g113600.t1.1 | 2.00E-08 | ataurora3 | 2.87E-03 | -26.5001 |
| Locus_6819_1Transcript_1/1_Confidence_1.000_Length_878 | Cre16.g651050.t1.1 | 1.00E-32 | Cytochrome c | 3.58E-02 | -14.3333 |
| Locus_616_3Transcript_5/227_Confidence_1.000_Length_1623 | Cre33.g782350.t1.2 | 6.00E-31 | DNA/RNA polymerases superfamily protein | 4.78E-02 | -8.80338 |
| Locus_12977_8Transcript_1/1_Confidence_1.000_Length_1308 | Cre03.g200250.t1.1 | 1.00E-157 | Phosphoenolpyruvate carboxylase family protein | 2.49E-03 | -7.25401 |
| Locus_3936_6Transcript_2/2_Confidence_0.857_Length_1754 | Cre15.g646150.t1.1 | 4.00E-147 | Tubulin/FtsZ family protein | 2.07E-02 | -4.77275 |
| Locus_1765_7Transcript_1/1_Confidence_1.000_Length_802 | Cre07.g323600.t1.1 | 1.00E-47 | NA | 3.46E-02 | -3.67431 |
| Locus_6299_6Transcript_1/1_Confidence_1.000_Length_1985 | Cre12.g516050.t1.1 | 1.00E-81 | RING/U-box superfamily protein | 3.92E-02 | -3.0671 |
| Locus_934_8Transcript_1/2_Confidence_0.667_Length_628 | Cre16.g667850.t1.1 | 1.00E-62 | DUTP-PYROPHOSPHATASE-LIKE 1 | 2.83E-02 | -3.00664 |
| Locus_319_9Transcript_1/1_Confidence_1.000_Length_4262 | Cre09.g393150.t1.1 | 0 | NA | 3.95E-03 | -2.90694 |
| Locus_1180_3Transcript_1/1_Confidence_1.000_Length_3285 | Cre12.g492950.t1.1 | 0 | ribonucleotide reductase 1 | 2.90E-02 | -2.85704 |
| Locus_26874_5Transcript_1/1_Confidence_1.000_Length_556 | Cre08.g368350.t1.2 | 2.00E-22 | NA | 6.36E-03 | -2.8371 |
| Locus_698_4Transcript_2/2_Confidence_0.667_Length_587 | Cre13.g567700.t1.1 | 1.00E-67 | histone H2A protein 9 | 1.37E-02 | -2.58981 |
| Locus_2591_3Transcript_1/1_Confidence_1.000_Length_768 | Cre13.g598050.t1.2 | 9.00E-23 | NA | 9.35E-03 | -2.47766 |
| Locus_992_5Transcript_1/1_Confidence_1.000_Length_1826 | Cre01.g022000.t1.2 | 2.00E-127 | P-loop containing nucleoside triphosphate hydrolases superfamily protein | 2.90E-02 | -2.34531 |
| Locus_32888_6Transcript_1/1_Confidence_1.000_Length_637 | Cre12.g495350.t1.2 | 7.00E-14 | NA | 3.82E-03 | -2.20545 |
| Locus_29558_2Transcript_1/1_Confidence_1.000_Length_518 | Cre01.g069800.t1.2 | 1.00E-23 | zinc ion binding | 2.46E-02 | -2.005 |
| Locus_5477_1Transcript_2/2_Confidence_0.857_Length_1996 | Cre06.g291850.t1.1 | 5.00E-86 | RNI-like superfamily protein | 3.75E-02 | 2.1912 |
| Locus_29119_8Transcript_1/1_Confidence_1.000_Length_887 | Cre15.g636650.t1.1 | 2.00E-23 | snRNA activating complex family protein | 4.72E-02 | 2.3641 |
| Locus_4628_3Transcript_3/7_Confidence_0.659_Length_1713 | Cre24.g769650.t1.2 | 5.00E-07 | NA | 7.55E-03 | 2.46698 |
| Locus_27798_3Transcript_1/1_Confidence_1.000_Length_484 | Cre03.g151850.t1.1 | 2.00E-43 | Protein of unknown function (DUF1264) | 3.34E-02 | 2.75495 |
| Locus_1523_6Transcript_4/4_Confidence_0.864_Length_3478 | Cre24.g769650.t1.2 | 5.00E-66 | NA | 2.46E-02 | 2.79176 |
| Locus_2411_4Transcript_1/1_Confidence_1.000_Length_1556 | Cre09.g405500.t1.2 | 1.00E-19 | NA | 1.15E-02 | 2.82208 |
| Locus_889_6Transcript_1/2_Confidence_0.667_Length_3064 | Cre18.g748050.t1.2 | 0 | Guanylate-binding family protein | 2.67E-02 | 3.48949 |
| Locus_12331_5Transcript_1/1_Confidence_1.000_Length_531 | Cre16.g664050.t1.1 | 1.00E-56 | GRAM domain family protein | 2.56E-02 | 3.92773 |
| Locus_34086_5Transcript_1/1_Confidence_1.000_Length_1137 | Cre02.g092000.t1.2 | 8.00E-130 | DegP protease 10 | 3.00E-02 | 4.25204 |
| Locus_32741_3Transcript_1/1_Confidence_1.000_Length_1098 | Cre03.g181200.t1.1 | 2.00E-95 | 3-methylcrotonyl-CoA carboxylase | 2.55E-02 | 4.87057 |
| Locus_32331_1Transcript_1/1_Confidence_1.000_Length_161 | Cre06.g296400.t1.1 | 3.00E-23 | isovaleryl-CoA-dehydrogenase | 3.29E-02 | 4.92595 |
| Locus_2820_8Transcript_1/1_Confidence_1.000_Length_2147 | Cre10.g448150.t1.2 | 5.00E-15 | NA | 8.27E-03 | 4.94659 |
| Locus_6559_3Transcript_1/1_Confidence_1.000_Length_1102 | Cre01.g012150.t1.1 | 4.00E-154 | peptidemethionine sulfoxide reductase 3 | 3.10E-02 | 6.14632 |
| Locus_19662_6Transcript_1/1_Confidence_1.000_Length_633 | Cre14.g619839.t1.2 | 4.00E-36 | sporulation 11-2 | 3.72E-02 | 6.39895 |
| Locus_9475_1Transcript_3/3_Confidence_0.750_Length_1264 | Cre03.g211500.t1.2 | 1.00E-40 | NA | 3.27E-02 | 7.24875 |
| Locus_419_5Transcript_2/10_Confidence_0.185_Length_1459 | Cre02.g140300.t1.2 | 2.00E-57 | Copper amine oxidase family protein | 1.23E-02 | 7.48076 |
| Locus_27829_2Transcript_1/1_Confidence_1.000_Length_1424 | Cre02.g140300.t1.2 | 0 | Copper amine oxidase family protein | 1.29E-02 | 7.58593 |
| Locus_14888_10Transcript_5/5_Confidence_0.583_Length_1689 | Cre24.g769600.t1.2 | 5.00E-18 | sodium proton exchanger, putative (NHX7) (SOS1) | 4.53E-03 | 7.59481 |
| Locus_1140_2Transcript_1/2_Confidence_0.444_Length_1856 | Cre12.g537400.t1.2 | 1.00E-150 | ataurora3 | 6.18E-03 | 8.04775 |
| Locus_18683_6Transcript_1/1_Confidence_1.000_Length_612 | Cre08.g383800.t1.2 | 7.00E-42 | NA | 2.23E-02 | 12.5338 |
| Locus_200_3Transcript_3/3_Confidence_0.600_Length_1227 | Cre03.g184100.t1.2 | 4.00E-12 | NA | 3.71E-03 | 14.5362 |
| Locus_8929_2Transcript_1/1_Confidence_1.000_Length_563 | Cre01.g001400.t1.1 | 9.00E-35 | metalloendopeptidases;zinc ion binding | 1.10E-03 | 14.8226 |
| Locus_3409_1Transcript_2/5_Confidence_0.222_Length_847 | Cre03.g184100.t1.2 | 4.00E-08 | NA | 7.56E-04 | 15.7152 |
| Locus_19531_5Transcript_1/1_Confidence_1.000_Length_778 | Cre14.g616050.t1.2 | 1.00E-19 | Pleckstrin homology (PH) domain superfamily protein | 1.08E-03 | 18.034 |
| Locus_1265_6Transcript_11/18_Confidence_0.163_Length_5624 | Cre14.g625600.t1.2 | 2.00E-38 | NA | 6.18E-03 | 18.7468 |
| Locus_19403_7Transcript_1/1_Confidence_1.000_Length_574 | Cre01.g045000.t1.1 | 4.00E-28 | CAP (Cysteine-rich secretory proteins, Antigen 5, and Pathogenesis-related 1 protein) superfamily protein | 9.30E-04 | 25.152 |
| Locus_4500_2Transcript_1/3_Confidence_0.778_Length_3788 | Cre03.g199050.t1.1 | 0 | Protein kinase family protein | 5.55E-03 | 25.8374 |
| Locus_10368_7Transcript_1/1_Confidence_1.000_Length_1058 | Cre08.g375400.t1.2 | 2.00E-18 | KNOTTED-like homeobox of Arabidopsis thaliana 7 | 1.13E-02 | 26.979 |
| Locus_4341_3Transcript_1/1_Confidence_1.000_Length_1858 | Cre06.g282800.t1.1 | 0 | isocitrate lyase | 1.20E-03 | 31.2022 |
| Locus_4829_3Transcript_3/6_Confidence_0.353_Length_1065 | Cre07.g312850.t1.1 | 8.00E-64 | Protein of unknown function (DUF679) | 2.04E-02 | 34.9229 |
| Locus_1804_7Transcript_3/3_Confidence_0.500_Length_782 | Cre08.g360900.t1.1 | 7.00E-48 | Ribosomal protein S19 family protein | 7.67E-03 | 41.9243 |
| Locus_24061_2Transcript_1/1_Confidence_1.000_Length_1050 | Cre03.g175950.t1.2 | 3.00E-18 | NA | 5.60E-03 | 45.2966 |
| Locus_21824_5Transcript_1/1_Confidence_1.000_Length_1966 | Cre01.g066100.t1.2 | 2.00E-178 | hapless 2 | 2.65E-04 | 45.7172 |
| Locus_4526_2Transcript_1/1_Confidence_1.000_Length_2170 | Cre06.g300500.t1.2 | 8.00E-100 | NA | 9.91E-04 | 50.1768 |
| Locus_2589_1Transcript_1/1_Confidence_1.000_Length_1215 | Cre08.g374250.t1.2 | 5.00E-43 | NA | 9.41E-05 | 51.9112 |
| Locus_958_6Transcript_1/1_Confidence_1.000_Length_1071 | Cre08.g374250.t1.2 | 3.00E-09 | NA | 3.26E-03 | 54.7768 |
| Locus_35963_3Transcript_1/1_Confidence_1.000_Length_747 | Cre09.g413750.t1.2 | 2.00E-52 | HOPW1-1-interacting 1 | 3.22E-02 | 60.0543 |
| Locus_14671_6Transcript_1/1_Confidence_1.000_Length_809 | Cre03.g175600.t1.2 | 7.00E-21 | Protein kinase superfamily protein | 3.55E-04 | 60.4262 |
| Locus_30418_5Transcript_1/1_Confidence_1.000_Length_1090 | Cre16.g669800.t1.1 | 1.00E-103 | AGC (cAMP-dependent, cGMP-dependent and protein kinase C) kinase family protein | 1.10E-03 | 65.931 |
| Locus_20421_2Transcript_1/1_Confidence_1.000_Length_354 | Cre06.g280600.t1.1 | 2.00E-13 | gamete expressed protein 1 | 2.81E-02 | 76.2204 |
| Locus_1_6Transcript_2441/2655_Confidence_1.000_Length_1371 | Cre02.g113752.t1.1 | 3.00E-16 | EIN3-binding F box protein 1 | 7.01E-03 | 453.601 |
| Locus_1664_3Transcript_7/13_Confidence_0.603_Length_5672 | Cre12.g493550.t1.1 | 2.00E-19 | Tetratricopeptide repeat (TPR)-like superfamily protein | 2.29E-02 | 487.316 |
| Locus_23751_6Transcript_1/1_Confidence_1.000_Length_630 | Cre02.g146650.t1.2 | 2.00E-26 | tubulin-tyrosine ligases;tubulin-tyrosine ligases | 2.36E-02 | 823.054 |
| Locus_25256_10Transcript_1/1_Confidence_1.000_Length_434 | Cre09.g392450.t1.2 | 6.00E-19 | sec23/sec24 transport family protein | 1.65E-03 | 879.173 |
| Locus_9292_2Transcript_1/1_Confidence_1.000_Length_415 | Cre16.g680850.t1.2 | 5.00E-07 | NA | 1.54E-02 | 1096.12 |
| Locus_14535_3Transcript_1/1_Confidence_1.000_Length_441 | Cre02.g089608.t1.1 | 8.00E-28 | RAD3-like DNA-binding helicase protein | 1.16E-03 | 1194.45 |
| Locus_39490_5Transcript_1/1_Confidence_1.000_Length_270 | Cre09.g405150.t1.2 | 6.00E-09 | Pseudouridine synthase family protein | 1.60E-03 | 1434.91 |
| Locus_7823_8Transcript_1/1_Confidence_1.000_Length_296 | Cre07.g325750.t1.1 | 2.00E-10 | NA | 9.03E-04 | 1588.26 |
| Locus_3943_3Transcript_2/3_Confidence_0.600_Length_1041 | Cre03.g153600.t1.1 | 2.00E-08 | NA | 2.17E-03 | 1724.24 |
| Locus_11153_5Transcript_2/5_Confidence_0.357_Length_656 | Cre03.g184100.t1.2 | 1.00E-08 | NA | 2.75E-02 | 1918.78 |
| Locus_23696_10Transcript_1/1_Confidence_1.000_Length_185 | Cre03.g161800.t1.1 | 6.00E-23 | ATPases;nucleotide binding;ATP binding;nucleoside-triphosphatases;transcription factor binding | 1.08E-03 | 1982.96 |
| Locus_40237_1Transcript_1/1_Confidence_1.000_Length_211 | Cre12.g542450.t1.1 | 2.00E-15 | P-loop containing nucleoside triphosphate hydrolases superfamily protein | 2.42E-04 | 2068.97 |
| Locus_41162_5Transcript_1/1_Confidence_1.000_Length_250 | Cre13.g578100.t1.2 | 3.00E-15 | Protein of unknown function (DUF3414) | 5.59E-03 | 2086.19 |
| Locus_9475_1Transcript_2/3_Confidence_0.750_Length_1290 | Cre03.g211500.t1.2 | 1.00E-14 | NA | 8.55E-04 | 2169.36 |
| Locus_13460_8Transcript_1/1_Confidence_1.000_Length_368 | Cre03.g162400.t1.2 | 2.00E-55 | NA | 7.07E-04 | 2257.44 |
| Locus_72438_10Transcript_1/1_Confidence_1.000_Length_166 | Cre14.g631200.t1.1 | 2.00E-09 | NA | 1.90E-03 | 2338.86 |
| Locus_22837_10Transcript_1/1_Confidence_1.000_Length_470 | Cre07.g312900.t1.2 | 7.00E-15 | HEAT repeat ;HECT-domain (ubiquitin-transferase) | 4.04E-03 | 2392.07 |
| Locus_17016_8Transcript_1/1_Confidence_1.000_Length_246 | Cre13.g602950.t1.2 | 1.00E-16 | NA | 8.42E-03 | 2790.87 |
| Locus_46803_10Transcript_1/1_Confidence_1.000_Length_564 | Cre02.g135300.t1.1 | 1.00E-06 | methyl-CPG-binding domain 9 | 4.62E-03 | 3003.41 |
| Locus_26060_9Transcript_1/1_Confidence_1.000_Length_366 | Cre14.g632950.t1.1 | 6.00E-28 | transcription regulators | 2.51E-03 | 3118.77 |
| Locus_42289_7Transcript_1/1_Confidence_1.000_Length_324 | Cre12.g483500.t1.2 | 5.00E-17 | pleckstrin homology (PH) domain-containing protein | 6.11E-03 | 3189.02 |
| Locus_42041_6Transcript_1/1_Confidence_1.000_Length_264 | Cre03.g211500.t1.2 | 3.00E-11 | NA | 2.52E-04 | 3243.31 |
| Locus_51004_10Transcript_1/1_Confidence_1.000_Length_324 | Cre16.g685900.t1.1 | 5.00E-11 | protein arginine methyltransferase 3 | 2.77E-03 | 4033.92 |
| Locus_24093_6Transcript_1/1_Confidence_1.000_Length_137 | Cre03.g175950.t1.2 | 1.00E-07 | NA | 1.42E-03 | 25380.2 |

(b) Differential expressed genes detected by in-house pipeline.

| **Dt_name** | **PPEE**  **(FDR)** | **Fold Change** | **ncbi_proteinid** | **function_name** | **E_Value** | **kegg_genes** | **kegg_orthology** |
| --- | --- | --- | --- | --- | --- | --- | --- |
| TRINITY_DN33164_c2_g2_i1 | 4.05E-08 | 0.000981959 | XP_002953052.1 | hypothetical protein VOLCADRAFT_82053 [Volvox carteri f.nagariensis] | 8.00E-43 | vcn:VOLCADRAFT_82053 | K01836 |
| TRINITY_DN1794_c0_g1_i4 | 0 | 0.00245181 | XP_002958740.1 | hypothetical protein VOLCADRAFT_100034 [Volvox carteri f.nagariensis] | 1.00E-47 | vcn:VOLCADRAFT_100034 | K02331 |
| TRINITY_DN41654_c0_g1_i3 | 0 | 0.00302066 | XP_002953476.1 | hypothetical protein VOLCADRAFT_109884 [Volvox carteri f.nagariensis] | 1.00E-155 | vcn:VOLCADRAFT_109884 | K10808 |
| TRINITY_DN29115_c0_g1_i5 | 0 | 0.00326218 | XP_002954216.1 | hypothetical protein VOLCADRAFT_82699 [Volvox carteri f. nagariensis] | 4.00E-75 | vcn:VOLCADRAFT_82699 | K05309 |
| TRINITY_DN24789_c1_g2_i1 | 0 | 0.00337751 | XP_002945626.1 | hypothetical protein VOLCADRAFT_102603 [Volvox carteri f.nagariensis] | 2.00E-22 | vcn:VOLCADRAFT_102603 | K04097 |
| TRINITY_DN46460_c1_g1_i1 | 0 | 0.00339126 | XP_002956915.1 | hypothetical protein VOLCADRAFT_30174, partial [Volvox carteri f.nagariensis] | 0 | vcn:VOLCADRAFT_30174 | K12828 |
| TRINITY_DN6552_c0_g2_i1 | 0 | 0.00342542 | XP_001700490.1 | predicted protein [Chlamydomonas reinhardtii] | 4.00E-99 | cre:CHLREDRAFT_194103 | K13125 |
| TRINITY_DN11538_c0_g1_i1 | 0 | 0.00347566 | XP_002949461.1 | hypothetical protein VOLCADRAFT_109721 [Volvox carteri f.nagariensis] | 7.00E-52 | vcn:VOLCADRAFT_109721 | K02137 |
| TRINITY_DN39388_c4_g4_i5 | 0 | 0.00350958 | XP_002955511.1 | hypothetical protein VOLCADRAFT_83295 [Volvox carteri f. nagariensis] | 1.00E-107 | vcn:VOLCADRAFT_83295 | K15103 |
| TRINITY_DN39388_c4_g4_i3 | 0 | 0.00398329 | XP_002955511.1 | hypothetical protein VOLCADRAFT_83295 [Volvox carteri f. nagariensis] | 1.00E-107 | vcn:VOLCADRAFT_83295 | K15103 |
| TRINITY_DN7072_c1_g3_i1 | 0 | 0.00435383 | XP_001701717.1 | SEC62-subunit of ER-translocon [Chlamydomonas reinhardtii] | 6.00E-65 | cre:CHLREDRAFT_186547 | K12275 |
| TRINITY_DN48149_c0_g2_i1 | 0 | 0.00489492 | XP_002950309.1 | hypothetical protein VOLCADRAFT_60238 [Volvox carteri f. nagariensis] | 0 | vcn:VOLCADRAFT_60238 | K05546 |
| TRINITY_DN48005_c0_g1_i12 | 0 | 0.00552395 | XP_011399333.1 | Centromere-associated protein E [Auxenochlorella protothecoides] | 2.00E-91 | apro:F751_0159 | K11498 |
| TRINITY_DN45878_c1_g2_i3 | 1.34E-05 | 0.00576626 | XP_002958086.1 | DEAH-box nuclear pre-mRNA splicing factor [Volvox carteri f.nagariensis] | 1.00E-176 | vcn:VOLCADRAFT_84321 | K12818 |
| TRINITY_DN33365_c0_g2_i1 | 0 | 0.00614731 | XP_005643160.1 | DEAD-domain-containing protein [Coccomyxa subellipsoidea C-169] | 1.00E-120 | csl:COCSUDRAFT_31548 | K17679 |
| TRINITY_DN26941_c0_g2_i1 | 0 | 0.00636186 | XP_001700779.1 | hypothetical protein CHLREDRAFT_113116, partial [Chlamydomonasreinhardtii] | 1.00E-156 | cre:CHLREDRAFT_113116 | K10590 |
| TRINITY_DN39278_c0_g2_i1 | 0 | 0.00691054 | XP_003064258.1 | kinesin-II motor protein, flagellar associated [Micromonas pusillaCCMP1545] | 2.00E-29 | mpp:MICPUCDRAFT_36912 | K10394 |
| TRINITY_DN27356_c0_g1_i1 | 2.69E-05 | 0.0071754 | XP_001689942.1 | deoxypusine synthase 1, partial [Chlamydomonas reinhardtii] | 1.00E-158 | cre:CHLREDRAFT_128965 | K00809 |
| TRINITY_DN44741_c0_g1_i6 | 0 | 0.00742305 | XP_002948501.1 | hypothetical protein VOLCADRAFT_58370, partial [Volvox carteri f.nagariensis] | 8.00E-80 | vcn:VOLCADRAFT_58370 | K01262 |
| TRINITY_DN47612_c4_g11_i1 | 0 | 0.00796965 | XP_001690237.1 | ATP synthase I-like protein [Chlamydomonas reinhardtii] | 6.00E-20 | cre:CHLREDRAFT_146879 | K02116 |
| TRINITY_DN3250_c0_g5_i1 | 0 | 0.00807145 | XP_001702822.1 | protein arginine N-methyltransferase [Chlamydomonas reinhardtii] | 1.00E-141 | cre:CHLREDRAFT_140543 | K11434 |
| TRINITY_DN60574_c0_g2_i2 | 0 | 0.0084105 | XP_002947162.1 | hypothetical protein VOLCADRAFT_103394 [Volvox carteri f.nagariensis] | 0 | vcn:VOLCADRAFT_103394 | K14811 |
| TRINITY_DN32737_c1_g1_i3 | 0 | 0.00861205 | XP_005650476.1 | NAF1-domain-containing protein [Coccomyxa subellipsoidea C-169] | 2.00E-18 | csl:COCSUDRAFT_64906 | K14763 |
| TRINITY_DN32174_c2_g1_i4 | 0 | 0.00862818 | XP_002947557.1 | hypothetical protein VOLCADRAFT_87798, partial [Volvox carteri f.nagariensis] | 9.00E-46 | vcn:VOLCADRAFT_87798 | K07300 |
| TRINITY_DN47233_c0_g3_i2 | 0 | 0.00870572 | XP_001703170.1 | flagellar inner arm dynein 1 heavy chain alpha [Chlamydomonasreinhardtii] | 0 | cre:CHLREDRAFT_60432 | K10408 |
| TRINITY_DN50830_c1_g3_i1 | 0 | 0.00880818 | XP_005646446.1 | Lecithin:cholesterol acyltransferase [Coccomyxa subellipsoidea C-169] | 1.00E-101 | csl:COCSUDRAFT_37169 | K00679 |
| TRINITY_DN60054_c2_g63_i1 | 0 | 0.00900994 | XP_001694498.1 | protoporphyrinogen oxidase [Chlamydomonas reinhardtii] | 9.00E-99 | cre:CHLREDRAFT_191043 | K00231 |
| TRINITY_DN60454_c1_g1_i1 | 0 | 0.00946978 | XP_002955877.1 | hypothetical protein VOLCADRAFT_96758 [Volvox carteri f. nagariensis] | 3.00E-19 | vcn:VOLCADRAFT_96758 | K14301 |
| TRINITY_DN22130_c0_g2_i1 | 0 | 0.00954332 | XP_005651712.1 | Prefoldin-domain-containing protein [Coccomyxa subellipsoideaC-169] | 8.00E-23 | csl:COCSUDRAFT_64101 | K04797 |
| TRINITY_DN6139_c0_g1_i2 | 0 | 0.00985449 | XP_002952566.1 | hypothetical protein VOLCADRAFT_109832 [Volvox carteri f.nagariensis] | 1.00E-141 | vcn:VOLCADRAFT_109832 | K06215 |
| TRINITY_DN50502_c0_g2_i1 | 0 | 0.00994826 | XP_001699854.1 | predicted protein, partial [Chlamydomonas reinhardtii] | 1.00E-105 | cre:CHLREDRAFT_112251 | K14820 |
| TRINITY_DN44777_c1_g4_i2 | 0 | 0.0107104 | XP_002949269.1 | hypothetical protein VOLCADRAFT_89548 [Volvox carteri f. nagariensis] | 2.00E-23 | vcn:VOLCADRAFT_89548 | K18667 |
| TRINITY_DN48415_c2_g3_i1 | 0 | 0.0109812 | XP_001691402.1 | SNF2 superfamily protein [Chlamydomonas reinhardtii] | 5.00E-42 | cre:CHLREDRAFT_170090 | K15710 |
| TRINITY_DN34756_c1_g2_i2 | 7.24E-06 | 0.0112344 | XP_002957215.1 | hypothetical protein VOLCADRAFT_98317 [Volvox carteri f.nagariensis] | 1.00E-45 | vcn:VOLCADRAFT_98317 | K17550 |
| TRINITY_DN31283_c0_g2_i1 | 0.0003513 | 0.0114795 | XP_002947222.1 | flagellar outer dynein arm heavy chain beta [Volvox carteri f.nagariensis] | 0 | vcn:VOLCADRAFT_79478 | K10408 |
| TRINITY_DN21269_c1_g2_i1 | 7.38E-08 | 0.0115924 | XP_001702954.1 | alanine-glyoxylate transaminase [Chlamydomonas reinhardtii] | 1.00E-153 | cre:CHLREDRAFT_133057 | K00827 |
| TRINITY_DN46571_c0_g1_i4 | 3.33E-16 | 0.0122218 | XP_002280613.1 | PREDICTED: cyclin-dependent kinase D-3 [Vitis vinifera] | 3.00E-28 | vvi:100247566 | K02202 |
| TRINITY_DN47233_c0_g3_i9 | 1.27E-11 | 0.0123714 | XP_001703170.1 | flagellar inner arm dynein 1 heavy chain alpha [Chlamydomonasreinhardtii] | 0 | cre:CHLREDRAFT_60432 | K10408 |
| TRINITY_DN23912_c1_g3_i1 | 2.99E-11 | 0.0125507 | XP_005645392.1 | hydroxymethylpyrimidine phosphate synthase [Coccomyxa subellipsoideaC-169] | 0 | csl:COCSUDRAFT_54252 | K03147 |
| TRINITY_DN50708_c0_g2_i1 | 0.000556545 | 0.0132446 | XP_005648825.1 | hypothetical protein COCSUDRAFT_28729 [Coccomyxa subellipsoideaC-169] | 0 | csl:COCSUDRAFT_28729 | K10610 |
| TRINITY_DN45297_c0_g1_i3 | 2.96E-14 | 0.0135064 | NP_213971.1 | cellulose synthase catalytic subunit [Aquifex aeolicus VF5] | 6.00E-29 | aae:aq_1407 | K00694 |
| TRINITY_DN28950_c0_g1_i1 | 3.44E-15 | 0.0139847 | XP_002948738.1 | hypothetical protein VOLCADRAFT_104067 [Volvox carteri f.nagariensis] | 1.00E-115 | vcn:VOLCADRAFT_104067 | K17605 |
| TRINITY_DN46577_c15_g1_i3 | 1.11E-16 | 0.0139925 | XP_007017771.1 | 5'-3' exoribonuclease 3 isoform 1 [Theobroma cacao] | 2.00E-16 | tcc:TCM_034205 | K12619 |
| TRINITY_DN60218_c1_g7_i1 | 1.23E-10 | 0.0139942 | XP_002957079.1 | protein disulfide isomerase 1 [Volvox carteri f. nagariensis] | 1.00E-147 | vcn:VOLCADRAFT_77404 | K09580 |
| TRINITY_DN23067_c1_g3_i1 | 7.77E-16 | 0.0141756 | XP_002951538.1 | hypothetical protein VOLCADRAFT_75062 [Volvox carteri f.nagariensis] | 2.00E-78 | vcn:VOLCADRAFT_75062 | K08516 |
| TRINITY_DN46825_c0_g2_i3 | 1.75E-09 | 0.0143979 | XP_002955730.1 | hypothetical protein VOLCADRAFT_83375 [Volvox carteri f.nagariensis] | 4.00E-35 | vcn:VOLCADRAFT_83375 | K03542 |
| TRINITY_DN40813_c0_g16_i1 | 6.33E-15 | 0.0150993 | XP_001692956.1 | pre-mRNA-splicing ATP-dependent RNA helicase [Chlamydomonasreinhardtii] | 0 | cre:CHLREDRAFT_136917 | K12858 |
| TRINITY_DN26025_c0_g2_i2 | 3.86E-10 | 0.0158347 | XP_002949252.1 | hypothetical protein VOLCADRAFT_104190 [Volvox carteri f.nagariensis] | 1.00E-22 | vcn:VOLCADRAFT_104190 | K11877 |
| TRINITY_DN47195_c0_g2_i2 | 9.84E-06 | 0.0159835 | XP_001703346.1 | centriole proteome protein, partial [Chlamydomonas reinhardtii] | 1.00E-161 | cre:CHLREDRAFT_9715 | K16533 |
| TRINITY_DN46499_c7_g3_i3 | 9.20E-08 | 0.0160174 | XP_005644118.1 | hypothetical protein COCSUDRAFT_58323 [Coccomyxa subellipsoideaC-169] | 1.00E-15 | csl:COCSUDRAFT_58323 | K08741 |
| TRINITY_DN29115_c0_g1_i4 | 1.22E-09 | 0.016087 | XP_002954216.1 | hypothetical protein VOLCADRAFT_82699 [Volvox carteri f. nagariensis] | 6.00E-40 | vcn:VOLCADRAFT_82699 | K05309 |
| TRINITY_DN32033_c0_g1_i4 | 9.42E-09 | 0.0161513 | XP_001695308.1 | protein required for templated centriole assembly [Chlamydomonasreinhardtii] | 8.00E-38 | cre:CHLREDRAFT_130542 | K16755 |
| TRINITY_DN35017_c0_g2_i1 | 3.68E-12 | 0.0162139 | XP_010236060.1 | PREDICTED: E3 ubiquitin-protein ligase COP1 [Brachypodium distachyon] | 3.00E-50 | bdi:100827481 | K10143 |
| TRINITY_DN42743_c0_g1_i2 | 2.74E-11 | 0.0163708 | XP_001691473.1 | seryl-tRNA(Sec) kinase [Chlamydomonas reinhardtii] | 1.00E-26 | cre:CHLREDRAFT_206160 | K10837 |
| TRINITY_DN45134_c0_g2_i1 | 1.95E-12 | 0.0170383 | XP_002948132.1 | hypothetical protein VOLCADRAFT_116706 [Volvox carteri f.nagariensis] | 9.00E-88 | vcn:VOLCADRAFT_116706 | K14408 |
| TRINITY_DN18272_c1_g1_i1 | 6.30E-09 | 0.0170862 | XP_002955255.1 | ribosomal protein S6 component of cytosolic 80S ribosome and 40Ssmall subunit [Volvox carteri f. nagariensis] | 2.00E-93 | vcn:VOLCADRAFT_127296 | K02991 |
| TRINITY_DN39203_c1_g1_i3 | 0.000392598 | 0.0177918 | XP_002949613.1 | hypothetical protein VOLCADRAFT_120772 [Volvox carteri f.nagariensis] | 1.00E-34 | vcn:VOLCADRAFT_120772 | K14165 |
| TRINITY_DN24514_c2_g1_i3 | 0 | 0.017833 | XP_001693396.1 | predicted protein, partial [Chlamydomonas reinhardtii] | 4.00E-42 | cre:CHLREDRAFT_98954 | K03470 |
| TRINITY_DN38304_c0_g2_i10 | 0.00216358 | 0.018097 | XP_005646084.1 | kinesin-domain-containing protein [Coccomyxa subellipsoidea C-169] | 3.00E-21 | csl:COCSUDRAFT_37341 | K10400 |
| TRINITY_DN42922_c0_g1_i2 | 3.75E-07 | 0.0183745 | XP_002955721.1 | hypothetical protein VOLCADRAFT_42256, partial [Volvox carteri f.nagariensis] | 3.00E-92 | vcn:VOLCADRAFT_42256 | K00166 |
| TRINITY_DN60774_c2_g2_i1 | 1.99E-05 | 0.0186845 | XP_001699805.1 | Mg2+ transporter protein, CorA-like protein [Chlamydomonasreinhardtii] | 1.00E-11 | cre:CHLREDRAFT_186922 | K16075 |
| TRINITY_DN40158_c0_g2_i1 | 7.52E-10 | 0.0188532 | XP_005848716.1 | hypothetical protein CHLNCDRAFT_144377 [Chlorella variabilis] | 2.00E-42 | cvr:CHLNCDRAFT_144377 | K11803 |
| TRINITY_DN11112_c0_g1_i1 | 1.46E-05 | 0.0193782 | XP_001702014.1 | predicted protein [Chlamydomonas reinhardtii] | 6.00E-70 | cre:CHLREDRAFT_155147 | K11864 |
| TRINITY_DN47299_c0_g1_i7 | 5.20E-09 | 0.0195295 | XP_002509213.1 | dynein heavy chain [Micromonas sp. RCC299] | 1.00E-132 | mis:MICPUN_96693 | K10408 |
| TRINITY_DN16698_c0_g1_i1 | 0.000257175 | 0.0198805 | XP_002953046.1 | hypothetical protein VOLCADRAFT_105760 [Volvox carteri f.nagariensis] | 1.00E-59 | vcn:VOLCADRAFT_105760 | K01259 |
| TRINITY_DN19162_c1_g2_i1 | 1.58E-08 | 0.0199416 | XP_001701661.1 | RNA pseudouridine synthase, partial [Chlamydomonas reinhardtii] | 9.00E-62 | cre:CHLREDRAFT_96859 | K15452 |
| TRINITY_DN47303_c0_g12_i5 | 5.94E-07 | 0.0203685 | XP_001702409.1 | ribulose-1,5-bisphosphate carboxylase/oxygenase small subunit 1,chloroplast precursor [Chlamydomonas reinhardtii] | 9.00E-55 | cre:CHLREDRAFT_82986 | K01602 |
| TRINITY_DN7940_c0_g1_i2 | 1.00E-07 | 0.0206659 | XP_001700042.1 | 26S proteasome regulatory subunit [Chlamydomonas reinhardtii] | 1.00E-117 | cre:CHLREDRAFT_23975 | K03036 |
| TRINITY_DN44174_c0_g3_i1 | 0.00048873 | 0.0209908 | XP_002952437.1 | hypothetical protein VOLCADRAFT_81829 [Volvox carteri f.nagariensis] | 1.00E-36 | vcn:VOLCADRAFT_81829 | K00670 |
| TRINITY_DN18045_c11_g5_i1 | 0.000893457 | 0.0212958 | XP_001695353.1 | chloropyll a-b binding protein of LHCII type I, chloroplastprecursor [Chlamydomonas reinhardtii] | 2.00E-24 | cre:CHLREDRAFT_184490 | K08912 |
| TRINITY_DN55239_c0_g3_i1 | 0.000988956 | 0.0213481 | XP_002953562.1 | hypothetical protein VOLCADRAFT_82311 [Volvox carteri f. nagariensis] | 1.00E-154 | vcn:VOLCADRAFT_82311 | K01963 |
| TRINITY_DN46548_c0_g1_i3 | 6.16E-07 | 0.0214309 | XP_002954046.1 | hypothetical protein VOLCADRAFT_121252, partial [Volvox carteri f.nagariensis] | 4.00E-47 | vcn:VOLCADRAFT_121252 | K14572 |
| TRINITY_DN46505_c1_g3_i2 | 6.20E-07 | 0.0214337 | XP_005843025.1 | hypothetical protein CHLNCDRAFT_59390 [Chlorella variabilis] | 4.00E-46 | cvr:CHLNCDRAFT_59390 | K06569 |
| TRINITY_DN47630_c0_g1_i13 | 3.76E-06 | 0.0217698 | XP_001703346.1 | centriole proteome protein, partial [Chlamydomonas reinhardtii] | 1.00E-109 | cre:CHLREDRAFT_9715 | K16533 |
| TRINITY_DN45443_c1_g1_i12 | 0.00290507 | 0.0219194 | XP_002947572.1 | hypothetical protein VOLCADRAFT_56931, partial [Volvox carteri f.nagariensis] | 1.00E-139 | vcn:VOLCADRAFT_56931 | K14536 |
| TRINITY_DN47840_c0_g1_i12 | 0.00835451 | 0.0225823 | XP_001702488.1 | chromosome condensation complex protein [Chlamydomonas reinhardtii] | 4.00E-14 | cre:CHLREDRAFT_186204 | K06677 |
| TRINITY_DN42922_c0_g1_i13 | 0.00279259 | 0.0234825 | XP_005848768.1 | hypothetical protein CHLNCDRAFT_22004 [Chlorella variabilis] | 1.00E-75 | cvr:CHLNCDRAFT_22004 | K00166 |
| TRINITY_DN46182_c10_g1_i1 | 0.00052021 | 0.0248588 | XP_001703024.1 | carboxylic ester hydrolase/lipase, partial [Chlamydomonasreinhardtii] | 8.00E-47 | cre:CHLREDRAFT_108522 | K01052 |
| TRINITY_DN4865_c0_g1_i1 | 0.00071461 | 0.0250482 | XP_005851445.1 | hypothetical protein CHLNCDRAFT_137782 [Chlorella variabilis] | 4.00E-58 | cvr:CHLNCDRAFT_137782 | K14759 |
| TRINITY_DN31333_c0_g7_i1 | 0.00493799 | 0.0251868 | XP_001702374.1 | Snf1-like protein kinase [Chlamydomonas reinhardtii] | 1.00E-156 | cre:CHLREDRAFT_185806 | K14498 |
| TRINITY_DN50977_c0_g1_i1 | 0.00281616 | 0.0255256 | XP_002502349.1 | predicted protein [Micromonas sp. RCC299] | 2.00E-37 | mis:MICPUN_58268 | K16196 |
| TRINITY_DN32186_c0_g1_i3 | 0.000401377 | 0.0256615 | XP_001699515.1 | heme oxygenase [Chlamydomonas reinhardtii] | 3.00E-12 | cre:CHLREDRAFT_152591 | K00510 |
| TRINITY_DN45615_c5_g7_i10 | 0.010527 | 0.0269159 | XP_002958779.1 | actin-binding protein gelsolin [Volvox carteri f. nagariensis] | 4.00E-14 | vcn:VOLCADRAFT_108308 | K05768 |
| TRINITY_DN20227_c0_g1_i1 | 0.00288393 | 0.0279086 | XP_005849080.1 | hypothetical protein CHLNCDRAFT_143575 [Chlorella variabilis] | 2.00E-81 | cvr:CHLNCDRAFT_143575 | K14829 |
| TRINITY_DN33622_c1_g1_i1 | 0.011366 | 0.0281292 | XP_002954696.1 | hypothetical protein VOLCADRAFT_118821 [Volvox carteri f.nagariensis] | 1.00E-57 | vcn:VOLCADRAFT_118821 | K12199 |
| TRINITY_DN47517_c0_g2_i5 | 0.00116112 | 0.0283467 | XP_002954979.1 | dynein heavy chain 9 [Volvox carteri f. nagariensis] | 0 | vcn:VOLCADRAFT_65425 | K10408 |
| TRINITY_DN38551_c0_g2_i1 | 0.0108733 | 0.0302058 | XP_001702714.1 | predicted protein [Chlamydomonas reinhardtii] | 8.00E-40 | cre:CHLREDRAFT_165870 | K00555 |
| TRINITY_DN45047_c1_g1_i13 | 0.0147288 | 0.0329518 | XP_010266929.1 | PREDICTED: probable DNA helicase MCM9 [Nelumbo nucifera] | 2.00E-93 | nnu:104604326 | K10738 |
| TRINITY_DN32441_c0_g3_i1 | 0.0222886 | 0.0340803 | XP_002949800.1 | hypothetical protein VOLCADRAFT_104457 [Volvox carteri f.nagariensis] | 1.00E-136 | vcn:VOLCADRAFT_104457 | K00645 |
| TRINITY_DN31523_c0_g1_i4 | 0.0101042 | 0.0351885 | XP_001693301.1 | Qc-SNARE protein, Bet1/mBET1 family [Chlamydomonas reinhardtii] | 1.00E-10 | cre:CHLREDRAFT_183904 | K08504 |
| TRINITY_DN28506_c0_g1_i3 | 0.019729 | 0.0372498 | XP_001689413.1 | lipoate protein ligase [Chlamydomonas reinhardtii] | 2.00E-53 | cre:CHLREDRAFT_195940 | K03801 |
| TRINITY_DN47434_c0_g2_i11 | 0.00941594 | 0.0390256 | XP_002945657.1 | microtubule-associated protein MAP65 [Volvox carteri f. nagariensis] | 2.00E-67 | vcn:VOLCADRAFT_120144 | K16732 |
| TRINITY_DN42551_c0_g1_i15 | 0.0132593 | 0.0403647 | XP_001701581.1 | predicted protein [Chlamydomonas reinhardtii] | 3.00E-11 | cre:CHLREDRAFT_194530 | K07252 |
| TRINITY_DN43599_c2_g1_i7 | 0.00930199 | 0.042776 | XP_002953149.1 | NimA-related protein kinase 4 [Volvox carteri f. nagariensis] | 7.00E-24 | vcn:VOLCADRAFT_82126 | K08857 |
| TRINITY_DN44176_c0_g1_i13 | 0.00385571 | 0.04347 | XP_001691362.1 | DnaJ-like protein, partial [Chlamydomonas reinhardtii] | 4.00E-13 | cre:CHLREDRAFT_10705 | K09531 |
| TRINITY_DN46641_c0_g1_i1 | 0.0406617 | 0.0445291 | XP_005847716.1 | hypothetical protein CHLNCDRAFT_133757 [Chlorella variabilis] | 2.00E-18 | cvr:CHLNCDRAFT_133757 | K10706 |
| TRINITY_DN36296_c5_g1_i7 | 0.013703 | 0.0473599 | XP_001693368.1 | hypothetical protein CHLREDRAFT_190601 [Chlamydomonas reinhardtii] | 6.00E-54 | cre:CHLREDRAFT_190601 | K17525 |
| TRINITY_DN45443_c1_g1_i6 | 0.00751811 | 0.048095 | XP_002947572.1 | hypothetical protein VOLCADRAFT_56931, partial [Volvox carteri f.nagariensis] | 1.00E-161 | vcn:VOLCADRAFT_56931 | K14536 |
| TRINITY_DN28382_c0_g2_i1 | 0.00849422 | 0.0521404 | XP_005851043.1 | hypothetical protein CHLNCDRAFT_29960 [Chlorella variabilis] | 5.00E-13 | cvr:CHLNCDRAFT_29960 | K00700 |
| TRINITY_DN40582_c0_g1_i4 | 0.00332213 | 0.0535071 | XP_005849153.1 | hypothetical protein CHLNCDRAFT_143780 [Chlorella variabilis] | 1.00E-38 | cvr:CHLNCDRAFT_143780 | K06677 |
| TRINITY_DN11434_c1_g6_i1 | 0.0283354 | 0.0560417 | XP_001694743.1 | histone methyltransferase [Chlamydomonas reinhardtii] | 1.00E-137 | cre:CHLREDRAFT_191388 | K11423 |
| TRINITY_DN47938_c1_g3_i31 | 0.00147568 | 0.059218 | XP_001694871.1 | sodium/phosphate symporter [Chlamydomonas reinhardtii] | 1.00E-64 | cre:CHLREDRAFT_196237 | K14640 |
| TRINITY_DN47883_c1_g1_i3 | 0 | 0.0595334 | XP_001691461.1 | dynein heavy chain [Chlamydomonas reinhardtii] | 0 | cre:CHLREDRAFT_206178 | K10408 |
| TRINITY_DN18002_c0_g1_i2 | 0.0424049 | 0.0607291 | XP_002953524.1 | B type mitotic cyclin [Volvox carteri f. nagariensis] | 1.00E-47 | vcn:VOLCADRAFT_127276 | K05868 |
| TRINITY_DN45963_c0_g2_i9 | 0.0413329 | 0.0638689 | XP_005843025.1 | hypothetical protein CHLNCDRAFT_59390 [Chlorella variabilis] | 1.00E-66 | cvr:CHLNCDRAFT_59390 | K06569 |
| TRINITY_DN43331_c1_g2_i4 | 0.000436457 | 0.064853 | XP_005851810.1 | hypothetical protein CHLNCDRAFT_33556 [Chlorella variabilis] | 8.00E-42 | cvr:CHLNCDRAFT_33556 | K13177 |
| TRINITY_DN43599_c2_g1_i12 | 0.00581789 | 0.0653644 | XP_002953149.1 | NimA-related protein kinase 4 [Volvox carteri f. nagariensis] | 1.00E-23 | vcn:VOLCADRAFT_82126 | K08857 |
| TRINITY_DN47299_c0_g1_i13 | 0.00182303 | 0.0665667 | XP_002509213.1 | dynein heavy chain [Micromonas sp. RCC299] | 1.00E-131 | mis:MICPUN_96693 | K10408 |
| TRINITY_DN40625_c0_g4_i2 | 0.0411515 | 0.06718 | XP_001702339.1 | 3',5'-cyclic-nucleotide phosphodiesterase [Chlamydomonas reinhardtii] | 2.00E-73 | cre:CHLREDRAFT_194640 | K13293 |
| TRINITY_DN47417_c0_g1_i32 | 0.0331617 | 0.0672573 | XP_002956896.1 | hypothetical protein VOLCADRAFT_97964 [Volvox carteri f. nagariensis] | 7.00E-19 | vcn:VOLCADRAFT_97964 | K06236 |
| TRINITY_DN44741_c0_g1_i7 | 0.00387916 | 0.0801647 | XP_002948501.1 | hypothetical protein VOLCADRAFT_58370, partial [Volvox carteri f.nagariensis] | 2.00E-85 | vcn:VOLCADRAFT_58370 | K01262 |
| TRINITY_DN36193_c0_g1_i5 | 0.021266 | 0.0850933 | XP_001701510.1 | kinesin-ii motor protein [Chlamydomonas reinhardtii] | 1.00E-80 | cre:CHLREDRAFT_185750 | K10394 |
| TRINITY_DN36849_c0_g2_i3 | 0.00675561 | 0.088196 | XP_011396503.1 | Chromatin assembly factor 1 subunit B [Auxenochlorellaprotothecoides] | 4.00E-71 | apro:F751_2888 | K10751 |
| TRINITY_DN34784_c1_g1_i1 | 0 | 0.0959621 | XP_011396375.1 | Deoxyuridine 5'-triphosphate nucleotidohydrolase [Auxenochlorellaprotothecoides] | 6.00E-49 | apro:F751_4384 | K01520 |
| TRINITY_DN39981_c1_g1_i4 | 0.000229155 | 0.103425 | XP_010691223.1 | PREDICTED: probable histone H2A variant 3 [Beta vulgaris subsp.vulgaris] | 1.00E-48 | bvg:104904618 | K11251 |
| TRINITY_DN46311_c13_g2_i7 | 0.0179374 | 0.107216 | XP_001689583.1 | predicted protein [Chlamydomonas reinhardtii] | 2.00E-56 | cre:CHLREDRAFT_171763 | K11858 |
| TRINITY_DN43141_c0_g1_i4 | 3.08E-10 | 0.109638 | XP_001693122.1 | centriole proteome protein, partial [Chlamydomonas reinhardtii] | 3.00E-53 | cre:CHLREDRAFT_13542 | K16757 |
| TRINITY_DN14349_c0_g1_i2 | 0.0100482 | 0.122515 | XP_001416103.1 | predicted protein [Ostreococcus lucimarinus CCE9901] | 3.00E-33 | olu:OSTLU_29930 | K01736 |
| TRINITY_DN43469_c0_g2_i2 | 0.000907313 | 0.122829 | XP_002958391.1 | hypothetical protein VOLCADRAFT_69374 [Volvox carteri f. nagariensis] | 2.00E-87 | vcn:VOLCADRAFT_69374 | K19787 |
| TRINITY_DN57900_c1_g1_i1 | 0 | 0.127161 | XP_002953231.1 | hypothetical protein VOLCADRAFT_93958 [Volvox carteri f.nagariensis] | 5.00E-61 | vcn:VOLCADRAFT_93958 | K06185 |
| TRINITY_DN47840_c0_g1_i6 | 0.020175 | 0.135031 | XP_005648295.1 | ARM repeat-containing protein [Coccomyxa subellipsoidea C-169] | 1.00E-58 | csl:COCSUDRAFT_63275 | K06677 |
| TRINITY_DN32850_c1_g1_i1 | 2.66E-11 | 0.139194 | XP_001699527.1 | flagellar associated protein [Chlamydomonas reinhardtii] | 1.00E-46 | cre:CHLREDRAFT_132213 | K06185 |
| TRINITY_DN45047_c1_g1_i19 | 0.0215527 | 0.142967 | XP_010266929.1 | PREDICTED: probable DNA helicase MCM9 [Nelumbo nucifera] | 3.00E-93 | nnu:104604326 | K10738 |
| TRINITY_DN47548_c3_g1_i2 | 0.00351717 | 0.148218 | XP_005644118.1 | hypothetical protein COCSUDRAFT_58323 [Coccomyxa subellipsoideaC-169] | 2.00E-60 | csl:COCSUDRAFT_58323 | K08741 |
| TRINITY_DN42219_c1_g1_i8 | 3.78E-05 | 0.157124 | XP_001689575.1 | separase, cell cycle protease [Chlamydomonas reinhardtii] | 6.00E-59 | cre:CHLREDRAFT_12539 | K02365 |
| TRINITY_DN42922_c0_g1_i15 | 0.0019242 | 0.171769 | XP_002955721.1 | hypothetical protein VOLCADRAFT_42256, partial [Volvox carteri f.nagariensis] | 9.00E-58 | vcn:VOLCADRAFT_42256 | K00166 |
| TRINITY_DN44685_c1_g1_i1 | 3.77E-09 | 0.183375 | XP_005845167.1 | hypothetical protein CHLNCDRAFT_58681 [Chlorella variabilis] | 3.00E-34 | cvr:CHLNCDRAFT_58681 | K15434 |
| TRINITY_DN47853_c0_g1_i1 | 1.48E-06 | 0.189468 | XP_001695440.1 | predicted protein [Chlamydomonas reinhardtii] | 1.00E-117 | cre:CHLREDRAFT_174686 | K16743 |
| TRINITY_DN2967_c0_g1_i1 | 5.48E-11 | 0.192754 | XP_005647298.1 | DnaJ-domain-containing protein [Coccomyxa subellipsoidea C-169] | 9.00E-33 | csl:COCSUDRAFT_63891 | K09537 |
| TRINITY_DN2499_c1_g1_i1 | 0.00528909 | 0.193454 | XP_002952497.1 | plastid division protein FtsZ2 [Volvox carteri f. nagariensis] | 1.00E-118 | vcn:VOLCADRAFT_81851 | K03531 |
| TRINITY_DN45047_c1_g1_i1 | 0.0408748 | 0.197867 | XP_011626766.1 | PREDICTED: probable DNA helicase MCM9 [Amborella trichopoda] | 1.00E-99 | atr:18423306 | K10738 |
| TRINITY_DN46363_c1_g2_i8 | 0.00356452 | 0.200423 | XP_001701617.1 | kinesin-like protein [Chlamydomonas reinhardtii] | 1.00E-165 | cre:CHLREDRAFT_186414 | K10397 |
| TRINITY_DN8688_c0_g2_i1 | 0.0395167 | 0.200882 | XP_002956068.1 | hypothetical protein VOLCADRAFT_76956 [Volvox carteri f.nagariensis] | 3.00E-24 | vcn:VOLCADRAFT_76956 | K00975 |
| TRINITY_DN35564_c2_g3_i4 | 0 | 0.209707 | XP_001694924.1 | predicted protein [Chlamydomonas reinhardtii] | 7.00E-34 | cre:CHLREDRAFT_158173 | K08073 |
| TRINITY_DN47517_c0_g2_i6 | 0.00462776 | 0.213197 | XP_002954979.1 | dynein heavy chain 9 [Volvox carteri f. nagariensis] | 0 | vcn:VOLCADRAFT_65425 | K10408 |
| TRINITY_DN45273_c1_g3_i4 | 0.0019359 | 0.218544 | XP_001689967.1 | NADP malic enzyme [Chlamydomonas reinhardtii] | 5.00E-77 | cre:CHLREDRAFT_196351 | K00029 |
| TRINITY_DN39075_c0_g2_i1 | 1.67E-13 | 0.220701 | XP_005852018.1 | hypothetical protein CHLNCDRAFT_132961 [Chlorella variabilis] | 1.00E-25 | cvr:CHLNCDRAFT_132961 | K10744 |
| TRINITY_DN38807_c3_g1_i2 | 4.50E-05 | 0.221823 | XP_001689583.1 | predicted protein [Chlamydomonas reinhardtii] | 4.00E-19 | cre:CHLREDRAFT_171763 | K11858 |
| TRINITY_DN32582_c0_g1_i1 | 0.000356326 | 0.229324 | XP_002948700.1 | hypothetical protein VOLCADRAFT_89087 [Volvox carteri f.nagariensis] | 5.00E-15 | vcn:VOLCADRAFT_89087 | K17428 |
| TRINITY_DN32093_c0_g3_i2 | 2.55E-05 | 0.237288 | XP_001698587.1 | predicted protein [Chlamydomonas reinhardtii] | 1.00E-61 | cre:CHLREDRAFT_187742 | K16745 |
| TRINITY_DN47399_c6_g5_i16 | 0.00553317 | 0.245529 | XP_001689583.1 | predicted protein [Chlamydomonas reinhardtii] | 5.00E-50 | cre:CHLREDRAFT_171763 | K11858 |
| TRINITY_DN46499_c7_g3_i2 | 0.0239255 | 0.246802 | XP_005848992.1 | hypothetical protein CHLNCDRAFT_144556 [Chlorella variabilis] | 7.00E-60 | cvr:CHLNCDRAFT_144556 | K08741 |
| TRINITY_DN46850_c4_g2_i4 | 1.68E-06 | 0.248645 | XP_005852064.1 | hypothetical protein CHLNCDRAFT_133064 [Chlorella variabilis] | 1.00E-103 | cvr:CHLNCDRAFT_133064 | K02836 |
| TRINITY_DN41654_c0_g1_i4 | 1.37E-14 | 0.255278 | XP_002953476.1 | hypothetical protein VOLCADRAFT_109884 [Volvox carteri f.nagariensis] | 1.00E-156 | vcn:VOLCADRAFT_109884 | K10808 |
| TRINITY_DN45314_c0_g3_i2 | 1.88E-06 | 0.259058 | XP_005651734.1 | FAD/NAD(P)-binding domain-containing protein [Coccomyxasubellipsoidea C-169] | 4.00E-48 | csl:COCSUDRAFT_64116 | K00486 |
| TRINITY_DN9283_c0_g1_i1 | 0.000759289 | 0.26012 | XP_005851323.1 | hypothetical protein CHLNCDRAFT_138182 [Chlorella variabilis] | 1.00E-14 | cvr:CHLNCDRAFT_138182 | K07047 |
| TRINITY_DN47399_c6_g5_i5 | 0.00214901 | 0.274955 | XP_001689583.1 | predicted protein [Chlamydomonas reinhardtii] | 4.00E-35 | cre:CHLREDRAFT_171763 | K11858 |
| TRINITY_DN45047_c1_g1_i21 | 4.51E-07 | 0.277832 | XP_010039824.1 | PREDICTED: probable DNA helicase MCM9 isoform X1 [Eucalyptus grandis] | 2.00E-99 | egr:104428551 | K10738 |
| TRINITY_DN47016_c7_g4_i4 | 3.19E-06 | 0.281823 | XP_002945867.1 | hypothetical protein VOLCADRAFT_120224 [Volvox carteri f.nagariensis] | 1.00E-167 | vcn:VOLCADRAFT_120224 | K14835 |
| TRINITY_DN42551_c0_g1_i2 | 2.34E-11 | 0.28636 | XP_001701581.1 | predicted protein [Chlamydomonas reinhardtii] | 2.00E-29 | cre:CHLREDRAFT_194530 | K07252 |
| TRINITY_DN37349_c2_g3_i8 | 0.0217381 | 0.300281 | XP_001693499.1 | predicted protein [Chlamydomonas reinhardtii] | 4.00E-35 | cre:CHLREDRAFT_182706 | K14401 |
| TRINITY_DN53037_c0_g1_i1 | 1.55E-08 | 0.315382 | XP_001698157.1 | replication protein A, 70 kDa DNA-binding subunit [Chlamydomonasreinhardtii] | 7.00E-85 | cre:CHLREDRAFT_205634 | K07466 |
| TRINITY_DN1264_c1_g4_i1 | 0.0457089 | 0.316456 | XP_001691107.1 | phosphatidate cytidylyltransferase [Chlamydomonas reinhardtii] | 8.00E-77 | cre:CHLREDRAFT_188754 | K00981 |
| TRINITY_DN48035_c0_g1_i1 | 0 | 0.327265 | XP_001694585.1 | multicopper ferroxidase [Chlamydomonas reinhardtii] | 0 | cre:CHLREDRAFT_184156 | K14735 |
| TRINITY_DN10107_c0_g1_i1 | 1.81E-05 | 0.329501 | XP_005644563.1 | NSG5 protein [Coccomyxa subellipsoidea C-169] | 0 | csl:COCSUDRAFT_54521 | K10807 |
| TRINITY_DN36416_c0_g1_i2 | 0 | 0.342089 | XP_001700649.1 | predicted protein [Chlamydomonas reinhardtii] | 2.00E-51 | cre:CHLREDRAFT_139957 | K06950 |
| TRINITY_DN46397_c0_g1_i9 | 4.10E-06 | 0.34607 | XP_011397088.1 | putative ATP-dependent RNA helicase DDX11 [Auxenochlorellaprotothecoides] | 7.00E-55 | apro:F751_5043 | K11273 |
| TRINITY_DN52955_c0_g1_i1 | 5.14E-13 | 0.35673 | XP_001697031.1 | chloroplast septum site-determining protein [Chlamydomonasreinhardtii] | 1.00E-112 | cre:CHLREDRAFT_184923 | K03609 |
| TRINITY_DN48223_c0_g2_i1 | 1.74E-11 | 0.358816 | XP_011402404.1 | Cob(I)yrinic acid a,c-diamide adenosyltransferase, mitochondrial[Auxenochlorella protothecoides] | 9.00E-42 | apro:F751_5789 | K00798 |
| TRINITY_DN43836_c1_g2_i3 | 1.79E-08 | 0.361518 | XP_002952850.1 | hypothetical protein VOLCADRAFT_105671 [Volvox carteri f.nagariensis] | 8.00E-22 | vcn:VOLCADRAFT_105671 | K13165 |
| TRINITY_DN40707_c0_g1_i3 | 0.00347937 | 0.362308 | XP_005849455.1 | hypothetical protein CHLNCDRAFT_142719 [Chlorella variabilis] | 1.00E-29 | cvr:CHLNCDRAFT_142719 | K18806 |
| TRINITY_DN39438_c1_g10_i5 | 9.75E-06 | 0.36533 | XP_001754507.1 | predicted protein [Physcomitrella patens] | 3.00E-77 | ppp:PHYPADRAFT_115789 | K11971 |
| TRINITY_DN8657_c0_g2_i1 | 0.00816102 | 0.365573 | XP_002953705.1 | hypothetical protein VOLCADRAFT_106052 [Volvox carteri f.nagariensis] | 1.00E-100 | vcn:VOLCADRAFT_106052 | K06674 |
| TRINITY_DN22922_c0_g1_i1 | 0.00161556 | 0.369264 | XP_001699275.1 | acyl-carrier protein [Chlamydomonas reinhardtii] | 2.00E-31 | cre:CHLREDRAFT_132151 | K03955 |
| TRINITY_DN30615_c0_g4_i1 | 0 | 0.372797 | XP_001690530.1 | hypothetical protein CHLREDRAFT_182890 [Chlamydomonas reinhardtii] | 8.00E-73 | cre:CHLREDRAFT_182890 | K19362 |
| TRINITY_DN57745_c0_g2_i1 | 2.54E-06 | 0.384585 | XP_001702420.1 | plastid division protein [Chlamydomonas reinhardtii] | 1.00E-115 | cre:CHLREDRAFT_140409 | K03531 |
| TRINITY_DN38123_c2_g2_i3 | 0.000148813 | 0.384869 | XP_002955064.1 | hypothetical protein VOLCADRAFT_85581 [Volvox carteri f. nagariensis] | 4.00E-64 | vcn:VOLCADRAFT_85581 | K02896 |
| TRINITY_DN45034_c8_g5_i2 | 0.000304243 | 0.395933 | XP_001702910.1 | predicted protein, partial [Chlamydomonas reinhardtii] | 5.00E-53 | cre:CHLREDRAFT_180040 | K19382 |
| TRINITY_DN39784_c2_g1_i3 | 2.94E-05 | 0.399234 | XP_011401883.1 | TIMELESS-interacting protein [Auxenochlorella protothecoides] | 6.00E-18 | apro:F751_4309 | K10904 |
| TRINITY_DN13351_c0_g1_i2 | 0 | 0.401678 | XP_002955914.1 | hypothetical protein VOLCADRAFT_106985 [Volvox carteri f.nagariensis] | 3.00E-99 | vcn:VOLCADRAFT_106985 | K15285 |
| TRINITY_DN46590_c0_g3_i3 | 0.00788584 | 0.4044 | XP_001695146.1 | ribosomal protein L39, component of cytosolic 80S ribosome and 60Slarge subunit [Chlamydomonas reinhardtii] | 2.00E-18 | cre:CHLREDRAFT_130723 | K02924 |
| TRINITY_DN45286_c2_g3_i3 | 3.78E-07 | 0.40738 | XP_001698866.1 | hypothetical protein CHLREDRAFT_151985, partial [Chlamydomonasreinhardtii] | 1.00E-38 | cre:CHLREDRAFT_151985 | K11271 |
| TRINITY_DN46623_c0_g3_i7 | 0.00178626 | 0.412712 | XP_002958631.1 | Kif5 kinesin, partial [Volvox carteri f. nagariensis] | 3.00E-80 | vcn:VOLCADRAFT_31481 | K10396 |
| TRINITY_DN21444_c0_g3_i2 | 2.20E-13 | 0.415071 | XP_002946055.1 | hypothetical protein VOLCADRAFT_78675 [Volvox carteri f. nagariensis] | 1.00E-118 | vcn:VOLCADRAFT_78675 | K11843 |
| TRINITY_DN57644_c0_g1_i1 | 0.00243481 | 0.416942 | XP_002947909.1 | hypothetical protein VOLCADRAFT_79941 [Volvox carteri f. nagariensis] | 1.00E-110 | vcn:VOLCADRAFT_79941 | K04802 |
| TRINITY_DN42179_c1_g1_i1 | 1.33E-09 | 0.424885 | XP_011399055.1 | FACT complex subunit SSRP1 [Auxenochlorella protothecoides] | 6.00E-17 | apro:F751_4652 | K09272 |
| TRINITY_DN38412_c0_g1_i1 | 8.82E-08 | 0.432016 | XP_002949309.1 | hypothetical protein VOLCADRAFT_117121 [Volvox carteri f.nagariensis] | 7.00E-41 | vcn:VOLCADRAFT_117121 | K07056 |
| TRINITY_DN37056_c0_g2_i2 | 0.00200119 | 0.432925 | XP_006342391.1 | PREDICTED: ATP-dependent DNA helicase Q-like 3 [Solanum tuberosum] | 4.00E-30 | sot:102581697 | K10901 |
| TRINITY_DN39938_c0_g1_i1 | 3.72E-06 | 0.435125 | XP_005845158.1 | hypothetical protein CHLNCDRAFT_137326 [Chlorella variabilis] | 4.00E-61 | cvr:CHLNCDRAFT_137326 | K03155 |
| TRINITY_DN55612_c0_g1_i1 | 0 | 0.435804 | XP_002954301.1 | hypothetical protein VOLCADRAFT_106317 [Volvox carteri f.nagariensis] | 6.00E-77 | vcn:VOLCADRAFT_106317 | K01307 |
| TRINITY_DN57981_c1_g1_i1 | 3.17E-12 | 0.436129 | XP_003058814.1 | predicted protein [Micromonas pusilla CCMP1545] | 2.00E-35 | mpp:MICPUCDRAFT_57959 | K10739 |
| TRINITY_DN38364_c1_g4_i4 | 6.64E-06 | 0.436977 | XP_002950951.1 | hypothetical protein VOLCADRAFT_91458 [Volvox carteri f. nagariensis] | 1.00E-135 | vcn:VOLCADRAFT_91458 | K00666 |
| TRINITY_DN6115_c0_g1_i1 | 4.37E-05 | 0.444823 | XP_001695365.1 | histone gene transcript 5' hairpin-binding protein, partial[Chlamydomonas reinhardtii] | 8.00E-20 | cre:CHLREDRAFT_95266 | K18710 |
| TRINITY_DN55296_c0_g1_i1 | 2.54E-08 | 0.44843 | XP_002955219.1 | hypothetical protein VOLCADRAFT_76573 [Volvox carteri f. nagariensis] | 1.00E-168 | vcn:VOLCADRAFT_76573 | K00051 |
| TRINITY_DN448_c0_g2_i1 | 0.00441258 | 0.450335 | XP_001696501.1 | cell division cycle protein 45, partial [Chlamydomonas reinhardtii] | 2.00E-84 | cre:CHLREDRAFT_142125 | K06628 |
| TRINITY_DN36626_c0_g1_i1 | 0 | 0.454008 | XP_001695895.1 | ortholog of meckel syndrome 1 [Chlamydomonas reinhardtii] | 9.00E-78 | cre:CHLREDRAFT_130473 | K16744 |
| TRINITY_DN36043_c0_g1_i2 | 0.0011856 | 0.457917 | XP_002951502.1 | hypothetical protein VOLCADRAFT_92063 [Volvox carteri f.nagariensis] | 1.00E-52 | vcn:VOLCADRAFT_92063 | K00799 |
| TRINITY_DN30053_c4_g1_i1 | 0.0348228 | 0.462176 | XP_005845513.1 | hypothetical protein CHLNCDRAFT_58502 [Chlorella variabilis] | 7.00E-27 | cvr:CHLNCDRAFT_58502 | K11291 |
| TRINITY_DN3242_c0_g2_i1 | 2.53E-05 | 0.463507 | XP_001695339.1 | predicted protein, partial [Chlamydomonas reinhardtii] | 8.00E-36 | cre:CHLREDRAFT_104097 | K16466 |
| TRINITY_DN1264_c1_g5_i1 | 2.55E-07 | 0.470412 | XP_001691107.1 | phosphatidate cytidylyltransferase [Chlamydomonas reinhardtii] | 8.00E-77 | cre:CHLREDRAFT_188754 | K00981 |
| TRINITY_DN41280_c1_g3_i2 | 8.68E-06 | 0.470585 | XP_002950776.1 | hypothetical protein VOLCADRAFT_120919 [Volvox carteri f.nagariensis] | 1.00E-75 | vcn:VOLCADRAFT_120919 | K13519 |
| TRINITY_DN35564_c2_g3_i3 | 0.0398615 | 0.471493 | XP_001694924.1 | predicted protein [Chlamydomonas reinhardtii] | 5.00E-34 | cre:CHLREDRAFT_158173 | K08073 |
| TRINITY_DN44391_c1_g1_i1 | 1.10E-06 | 0.47193 | XP_001700953.1 | primase subunit of DNA polymerase alpha [Chlamydomonas reinhardtii] | 1.00E-143 | cre:CHLREDRAFT_54359 | K02684 |
| TRINITY_DN47390_c0_g3_i7 | 0.0014306 | 0.472747 | XP_003059396.1 | predicted protein, partial [Micromonas pusilla CCMP1545] | 9.00E-30 | mpp:MICPUCDRAFT_69392 | K08737 |
| TRINITY_DN26846_c0_g2_i2 | 4.31E-09 | 0.473105 | XP_005848182.1 | hypothetical protein CHLNCDRAFT_22624 [Chlorella variabilis] | 1.00E-85 | cvr:CHLNCDRAFT_22624 | K02732 |
| TRINITY_DN13192_c1_g2_i1 | 7.93E-12 | 0.47361 | XP_002948852.1 | hypothetical protein VOLCADRAFT_116951, partial [Volvox carteri f.nagariensis] | 0 | vcn:VOLCADRAFT_116951 | K19348 |
| TRINITY_DN45164_c0_g2_i3 | 0.0215333 | 0.476945 | XP_005850035.1 | hypothetical protein CHLNCDRAFT_142022 [Chlorella variabilis] | 1.00E-70 | cvr:CHLNCDRAFT_142022 | K12310 |
| TRINITY_DN34009_c0_g1_i1 | 6.28E-05 | 0.479209 | XP_005845240.1 | hypothetical protein CHLNCDRAFT_137518 [Chlorella variabilis] | 5.00E-24 | cvr:CHLNCDRAFT_137518 | K14294 |
| TRINITY_DN43141_c0_g1_i8 | 0.0291942 | 0.47949 | XP_002955641.1 | centriole protein [Volvox carteri f. nagariensis] | 6.00E-96 | vcn:VOLCADRAFT_96623 | K16757 |
| TRINITY_DN47633_c0_g4_i2 | 0.000198429 | 0.481728 | XP_002949672.1 | hypothetical protein VOLCADRAFT_90164 [Volvox carteri f. nagariensis] | 0 | vcn:VOLCADRAFT_90164 | K19352 |
| TRINITY_DN18128_c1_g2_i2 | 0.0435403 | 0.482728 | XP_002955833.1 | hypothetical protein VOLCADRAFT_66339 [Volvox carteri f. nagariensis] | 2.00E-61 | vcn:VOLCADRAFT_66339 | K15281 |
| TRINITY_DN17437_c1_g2_i1 | 0.000473276 | 0.484471 | XP_011401518.1 | Nudix hydrolase 1, partial [Auxenochlorella protothecoides] | 1.00E-24 | apro:F751_5727 | K03574 |
| TRINITY_DN44324_c0_g1_i5 | 0 | 0.487532 | XP_002953871.1 | hypothetical protein VOLCADRAFT_64063 [Volvox carteri f. nagariensis] | 1.00E-107 | vcn:VOLCADRAFT_64063 | K02685 |
| TRINITY_DN45286_c0_g1_i2 | 0.000241952 | 0.487723 | XP_001698866.1 | hypothetical protein CHLREDRAFT_151985, partial [Chlamydomonasreinhardtii] | 2.00E-27 | cre:CHLREDRAFT_151985 | K11271 |
| TRINITY_DN17437_c1_g2_i2 | 0.00125772 | 0.491809 | XP_011401518.1 | Nudix hydrolase 1, partial [Auxenochlorella protothecoides] | 1.00E-30 | apro:F751_5727 | K03574 |
| TRINITY_DN21184_c0_g3_i1 | 5.36E-11 | 0.492031 | XP_002952572.1 | hypothetical protein VOLCADRAFT_93192 [Volvox carteri f. nagariensis] | 4.00E-60 | vcn:VOLCADRAFT_93192 | K11303 |
| TRINITY_DN36663_c1_g2_i1 | 0 | 0.496616 | XP_002957249.1 | hypothetical protein VOLCADRAFT_77509 [Volvox carteri f.nagariensis] | 1.00E-79 | vcn:VOLCADRAFT_77509 | K16745 |
| TRINITY_DN45034_c8_g5_i3 | 0 | 0.497125 | XP_001702910.1 | predicted protein, partial [Chlamydomonas reinhardtii] | 8.00E-56 | cre:CHLREDRAFT_180040 | K19382 |
| TRINITY_DN12156_c0_g1_i1 | 0 | 0.497347 | XP_011399055.1 | FACT complex subunit SSRP1 [Auxenochlorella protothecoides] | 3.00E-16 | apro:F751_4652 | K09272 |
| TRINITY_DN27663_c0_g2_i2 | 0.00372265 | 0.498123 | XP_005849233.1 | hypothetical protein CHLNCDRAFT_51197 [Chlorella variabilis] | 2.00E-15 | cvr:CHLNCDRAFT_51197 | K06085 |
| TRINITY_DN45438_c3_g1_i1 | 2.79E-08 | 0.500061 | XP_001701896.1 | DNA repair glycosylase, partial [Chlamydomonas reinhardtii] | 8.00E-15 | cre:CHLREDRAFT_141355 | K03575 |
| TRINITY_DN39382_c0_g4_i2 | 0.000275729 | 0.503587 | XP_002956198.1 | hypothetical protein VOLCADRAFT_77041 [Volvox carteri f.nagariensis] | 4.00E-91 | vcn:VOLCADRAFT_77041 | K01915 |
| TRINITY_DN24734_c1_g1_i1 | 9.46E-05 | 0.508672 | XP_005847079.1 | hypothetical protein CHLNCDRAFT_134765 [Chlorella variabilis] | 5.00E-22 | cvr:CHLNCDRAFT_134765 | K10751 |
| TRINITY_DN41057_c0_g1_i1 | 0.0453131 | 0.509993 | XP_002957192.1 | hypothetical protein VOLCADRAFT_98273 [Volvox carteri f. nagariensis] | 1.00E-100 | vcn:VOLCADRAFT_98273 | K14521 |
| TRINITY_DN30507_c0_g1_i1 | 0 | 0.510897 | XP_005846032.1 | hypothetical protein CHLNCDRAFT_25365, partial [Chlorellavariabilis] | 3.00E-54 | cvr:CHLNCDRAFT_25365 | K11422 |
| TRINITY_DN18898_c1_g1_i1 | 2.22E-16 | 0.51157 | XP_001699653.1 | centriole proteome protein, partial [Chlamydomonas reinhardtii] | 1.00E-158 | cre:CHLREDRAFT_112249 | K16482 |
| TRINITY_DN45831_c0_g2_i3 | 9.81E-06 | 0.511749 | XP_009351018.1 | PREDICTED: ATP-dependent DNA helicase PIF1-like [Pyrus xbretschneideri] | 4.00E-61 | pxb:103942556 | K15255 |
| TRINITY_DN14925_c1_g2_i1 | 2.50E-05 | 0.511764 | XP_005847690.1 | hypothetical protein CHLNCDRAFT_134115 [Chlorella variabilis] | 2.00E-90 | cvr:CHLNCDRAFT_134115 | K15340 |
| TRINITY_DN24789_c1_g1_i1 | 0.0140724 | 0.514295 | XP_002945626.1 | hypothetical protein VOLCADRAFT_102603 [Volvox carteri f.nagariensis] | 7.00E-40 | vcn:VOLCADRAFT_102603 | K04097 |
| TRINITY_DN58240_c1_g1_i1 | 3.24E-05 | 0.514785 | XP_002958416.1 | hypothetical protein VOLCADRAFT_108124 [Volvox carteri f.nagariensis] | 5.00E-24 | vcn:VOLCADRAFT_108124 | K17969 |
| TRINITY_DN34041_c0_g2_i3 | 0.000125201 | 0.514799 | XP_001695715.1 | tRNA-2'-O-ribose methyltransferase [Chlamydomonas reinhardtii] | 3.00E-96 | cre:CHLREDRAFT_119276 | K14864 |
| TRINITY_DN17380_c4_g1_i1 | 0.0487862 | 0.514805 | XP_015055629.1 | PREDICTED: chlorophyll a-b binding protein 8, chloroplastic [Solanumpennellii] | 3.00E-55 | spen:107002211 | K08909 |
| TRINITY_DN47351_c18_g2_i1 | 0.00300462 | 0.517159 | XP_005646130.1 | molybdenum cofactor biosynthesis prote [Coccomyxa subellipsoideaC-169] | 9.00E-84 | csl:COCSUDRAFT_24726 | K03639 |
| TRINITY_DN57892_c2_g1_i1 | 1.30E-12 | 0.518244 | XP_001701896.1 | DNA repair glycosylase, partial [Chlamydomonas reinhardtii] | 3.00E-85 | cre:CHLREDRAFT_141355 | K03575 |
| TRINITY_DN42114_c1_g2_i1 | 0.00316509 | 0.518508 | XP_011395839.1 | Leucyl-cystinyl aminopeptidase [Auxenochlorella protothecoides] | 4.00E-27 | apro:F751_0330 | K08776 |
| TRINITY_DN34640_c0_g2_i1 | 4.51E-07 | 0.518891 | XP_005650630.1 | S-adenosyl-L-methionine-dependent methyltransferase [Coccomyxasubellipsoidea C-169] | 1.00E-86 | csl:COCSUDRAFT_32480 | K18477 |
| TRINITY_DN50725_c0_g1_i1 | 4.61E-08 | 0.52212 | XP_002946883.1 | hypothetical protein VOLCADRAFT_116136 [Volvox carteri f.nagariensis] | 1.00E-140 | vcn:VOLCADRAFT_116136 | K02324 |
| TRINITY_DN57795_c1_g2_i1 | 0.000309956 | 0.522431 | XP_001690540.1 | hypothetical protein CHLREDRAFT_182903 [Chlamydomonas reinhardtii] | 3.00E-18 | cre:CHLREDRAFT_182903 | K18171 |
| TRINITY_DN47706_c9_g1_i1 | 0.0142649 | 0.527528 | XP_002948658.1 | WD40 protein [Volvox carteri f. nagariensis] | 5.00E-97 | vcn:VOLCADRAFT_109001 | K14963 |
| TRINITY_DN3566_c0_g1_i1 | 1.06E-10 | 0.529824 | XP_001689574.1 | DNA replication complex GINS protein [Chlamydomonas reinhardtii] | 1.00E-45 | cre:CHLREDRAFT_146514 | K10733 |
| TRINITY_DN39049_c0_g2_i1 | 1.82E-06 | 0.530366 | XP_001699109.1 | hypothetical molybdopterin biosynthesis protein, MoaA family,partial [Chlamydomonas reinhardtii] | 6.00E-44 | cre:CHLREDRAFT_106419 | K03639 |
| TRINITY_DN11538_c0_g3_i1 | 7.10E-07 | 0.531407 | XP_002949461.1 | hypothetical protein VOLCADRAFT_109721 [Volvox carteri f.nagariensis] | 2.00E-52 | vcn:VOLCADRAFT_109721 | K02137 |
| TRINITY_DN44169_c1_g1_i1 | 2.87E-08 | 0.532223 | XP_001692026.1 | SWI/SNF chromatin remodeling complex component [Chlamydomonasreinhardtii] | 1.00E-146 | cre:CHLREDRAFT_145450 | K10877 |
| TRINITY_DN18783_c2_g1_i1 | 6.11E-15 | 0.538696 | XP_002952572.1 | hypothetical protein VOLCADRAFT_93192 [Volvox carteri f. nagariensis] | 1.00E-12 | vcn:VOLCADRAFT_93192 | K11303 |
| TRINITY_DN57913_c1_g2_i1 | 7.49E-05 | 0.540063 | XP_011396218.1 | Malate dehydrogenase, glyoxysomal [Auxenochlorella protothecoides] | 8.00E-12 | apro:F751_1044 | K00026 |
| TRINITY_DN14173_c1_g1_i1 | 0.0147522 | 0.543055 | XP_005650459.1 | small nuclear ribonucleo protein E [Coccomyxa subellipsoidea C-169] | 2.00E-33 | csl:COCSUDRAFT_35455 | K11097 |
| TRINITY_DN43530_c1_g1_i2 | 3.83E-07 | 0.547212 | XP_002948740.1 | hypothetical protein VOLCADRAFT_104070 [Volvox carteri f.nagariensis] | 1.00E-54 | vcn:VOLCADRAFT_104070 | K00685 |
| TRINITY_DN42443_c1_g1_i2 | 0.000118172 | 0.553474 | XP_005649211.1 | DNA repair helicase [Coccomyxa subellipsoidea C-169] | 2.00E-55 | csl:COCSUDRAFT_14063 | K11273 |
| TRINITY_DN34962_c0_g2_i1 | 5.90E-08 | 0.556775 | XP_002958390.1 | hypothetical protein VOLCADRAFT_99672 [Volvox carteri f. nagariensis] | 4.00E-26 | vcn:VOLCADRAFT_99672 | K10877 |
| TRINITY_DN3302_c0_g2_i1 | 0 | 0.557433 | XP_002947321.1 | hypothetical protein VOLCADRAFT_120451 [Volvox carteri f.nagariensis] | 1.00E-107 | vcn:VOLCADRAFT_120451 | K00472 |
| TRINITY_DN23657_c2_g9_i1 | 0.00177096 | 0.557573 | XP_002961812.1 | hypothetical protein SELMODRAFT_165253 [Selaginella moellendorffii] | 1.00E-43 | smo:SELMODRAFT_165253 | K12178 |
| TRINITY_DN34332_c0_g1_i1 | 1.17E-07 | 0.557708 | XP_002950020.1 | hypothetical protein VOLCADRAFT_80889 [Volvox carteri f.nagariensis] | 3.00E-25 | vcn:VOLCADRAFT_80889 | K03349 |
| TRINITY_DN467_c1_g1_i1 | 0 | 0.558074 | XP_002948753.1 | hypothetical protein VOLCADRAFT_58667 [Volvox carteri f. nagariensis] | 1.00E-168 | vcn:VOLCADRAFT_58667 | K08830 |
| TRINITY_DN37609_c0_g1_i1 | 8.88E-16 | 0.559933 | XP_002954155.1 | hypothetical protein VOLCADRAFT_106247 [Volvox carteri f.nagariensis] | 1.00E-74 | vcn:VOLCADRAFT_106247 | K11407 |
| TRINITY_DN3249_c0_g1_i2 | 0.00873943 | 0.561089 | XP_010031236.1 | PREDICTED: arginyl-tRNA--protein transferase 1-like [Eucalyptusgrandis] | 2.00E-23 | egr:104421097 | K00685 |
| TRINITY_DN36070_c1_g1_i1 | 0.0303831 | 0.563378 | XP_002873092.1 | zinc finger family protein [Arabidopsis lyrata subsp. lyrata] | 2.00E-12 | aly:ARALYDRAFT_487121 | K15691 |
| TRINITY_DN28462_c0_g2_i3 | 1.52E-08 | 0.564898 | XP_001697086.1 | hypothetical protein CHLREDRAFT_176336 [Chlamydomonas reinhardtii] | 1.00E-180 | cre:CHLREDRAFT_176336 | K16487 |
| TRINITY_DN47635_c9_g7_i1 | 0.00245725 | 0.566068 | YP_445018.1 | ribosomal RNA small subunit methyltransferase B [Salinibacter ruberDSM 13855] | 4.00E-29 | sru:SRU_0884 | K03500 |
| TRINITY_DN52959_c0_g2_i1 | 0.00683211 | 0.569662 | XP_001695959.1 | adenosine 5'-phosphosulfate kinase [Chlamydomonas reinhardtii] | 1.00E-80 | cre:CHLREDRAFT_184419 | K00860 |
| TRINITY_DN41473_c2_g3_i1 | 0.0308913 | 0.569992 | XP_002950688.1 | hypothetical protein VOLCADRAFT_104814 [Volvox carteri f.nagariensis] | 6.00E-40 | vcn:VOLCADRAFT_104814 | K15033 |
| TRINITY_DN29270_c0_g1_i3 | 0.000311849 | 0.57187 | XP_002953001.1 | hypothetical protein VOLCADRAFT_118277 [Volvox carteri f.nagariensis] | 2.00E-64 | vcn:VOLCADRAFT_118277 | K13348 |
| TRINITY_DN55572_c2_g1_i1 | 1.19E-13 | 0.572486 | XP_001703250.1 | DNA polymerase epsilon catalytic subunit A/1 [Chlamydomonasreinhardtii] | 2.00E-54 | cre:CHLREDRAFT_9481 | K02324 |
| TRINITY_DN36626_c0_g2_i1 | 0.037797 | 0.573098 | XP_002952858.1 | hypothetical protein VOLCADRAFT_105683 [Volvox carteri f.nagariensis] | 2.00E-41 | vcn:VOLCADRAFT_105683 | K16744 |
| TRINITY_DN58142_c1_g1_i1 | 0.00315717 | 0.5738 | XP_005643385.1 | anti-silence-domain-containing protein [Coccomyxa subellipsoideaC-169] | 1.00E-53 | csl:COCSUDRAFT_20133 | K10753 |
| TRINITY_DN44810_c0_g1_i5 | 3.98E-06 | 0.574027 | XP_001703420.1 | calmodulin [Chlamydomonas reinhardtii] | 7.00E-70 | cre:CHLREDRAFT_188144 | K02183 |
| TRINITY_DN45585_c1_g1_i1 | 0.000135211 | 0.577133 | XP_005650052.1 | Metallo-dependent phosphatase [Coccomyxa subellipsoidea C-169] | 1.00E-100 | csl:COCSUDRAFT_35839 | K04460 |
| TRINITY_DN40110_c0_g1_i2 | 1.51E-08 | 0.578989 | XP_003061818.1 | predicted protein [Micromonas pusilla CCMP1545] | 5.00E-66 | mpp:MICPUCDRAFT_20393 | K08869 |
| TRINITY_DN5961_c0_g1_i1 | 0.0166957 | 0.582216 | XP_002953223.1 | hypothetical protein VOLCADRAFT_105835 [Volvox carteri f.nagariensis] | 1.00E-99 | vcn:VOLCADRAFT_105835 | K08737 |
| TRINITY_DN23248_c0_g2_i1 | 0.0330205 | 0.583725 | XP_002957568.1 | hypothetical protein VOLCADRAFT_107737 [Volvox carteri f.nagariensis] | 0 | vcn:VOLCADRAFT_107737 | K02320 |
| TRINITY_DN47085_c3_g1_i1 | 0 | 0.587438 | XP_005850716.1 | hypothetical protein CHLNCDRAFT_140821 [Chlorella variabilis] | 5.00E-34 | cvr:CHLNCDRAFT_140821 | K18158 |
| TRINITY_DN43665_c0_g2_i1 | 5.00E-10 | 0.587608 | XP_002950575.1 | hypothetical protein VOLCADRAFT_81183 [Volvox carteri f. nagariensis] | 1.00E-123 | vcn:VOLCADRAFT_81183 | K02328 |
| TRINITY_DN52993_c0_g1_i1 | 5.21E-05 | 0.589634 | XP_002953223.1 | hypothetical protein VOLCADRAFT_105835 [Volvox carteri f.nagariensis] | 1.00E-158 | vcn:VOLCADRAFT_105835 | K08737 |
| TRINITY_DN47973_c29_g8_i1 | 0.00028179 | 0.589653 | XP_002946957.1 | epsilon tubulin [Volvox carteri f. nagariensis] | 0 | vcn:VOLCADRAFT_56250 | K10391 |
| TRINITY_DN40923_c0_g1_i4 | 6.41E-07 | 0.589861 | XP_002946615.1 | choline, partial [Volvox carteri f. nagariensis] | 7.00E-66 | vcn:VOLCADRAFT_43837 | K00894 |
| TRINITY_DN50508_c0_g1_i1 | 1.95E-06 | 0.59041 | XP_001696579.1 | glutathione reductase [Chlamydomonas reinhardtii] | 0 | cre:CHLREDRAFT_134165 | K00383 |
| TRINITY_DN52907_c0_g1_i1 | 4.33E-07 | 0.590769 | XP_011399722.1 | ATP-dependent DNA helicase Q-like 1 [Auxenochlorella protothecoides] | 1.00E-116 | apro:F751_0706 | K10901 |
| TRINITY_DN6063_c0_g1_i1 | 0.0254489 | 0.591705 | XP_002956921.1 | hypothetical protein VOLCADRAFT_107445 [Volvox carteri f.nagariensis] | 1.00E-72 | vcn:VOLCADRAFT_107445 | K14411 |
| TRINITY_DN46787_c0_g3_i1 | 0.0324376 | 0.591784 | XP_001697423.1 | katanin p80 subunit [Chlamydomonas reinhardtii] | 7.00E-94 | cre:CHLREDRAFT_80954 | K18643 |
| TRINITY_DN48318_c0_g1_i1 | 6.96E-08 | 0.592811 | XP_001695560.1 | hypothetical protein CHLREDRAFT_138215 [Chlamydomonas reinhardtii] | 1.00E-46 | cre:CHLREDRAFT_138215 | K02183 |
| TRINITY_DN23757_c1_g1_i1 | 8.89E-05 | 0.595211 | XP_002948518.1 | hypothetical protein VOLCADRAFT_88956 [Volvox carteri f. nagariensis] | 2.00E-17 | vcn:VOLCADRAFT_88956 | K16470 |
| TRINITY_DN36645_c1_g1_i1 | 0 | 0.596512 | XP_002945688.1 | hypothetical protein VOLCADRAFT_85913 [Volvox carteri f.nagariensis] | 1.00E-35 | vcn:VOLCADRAFT_85913 | K19384 |
| TRINITY_DN58008_c0_g1_i1 | 0.0121824 | 0.598182 | XP_002946329.1 | hypothetical protein VOLCADRAFT_79001 [Volvox carteri f. nagariensis] | 1.00E-123 | vcn:VOLCADRAFT_79001 | K15103 |
| TRINITY_DN58033_c0_g1_i1 | 0.00211066 | 0.599699 | XP_001689909.1 | DNA polymerase delta subunit one [Chlamydomonas reinhardtii] | 0 | cre:CHLREDRAFT_189721 | K02327 |
| TRINITY_DN2507_c0_g1_i1 | 0 | 0.600437 | XP_011083237.1 | PREDICTED: putative 3,4-dihydroxy-2-butanone kinase [Sesamum indicum] | 1.00E-129 | sind:105165796 | K00863 |
| TRINITY_DN46131_c15_g10_i1 | 0 | 0.602391 | XP_001760684.1 | gamma tubulin ring complex protein 3 [Physcomitrella patens] | 1.00E-133 | ppp:PHYPADRAFT_162526 | K16570 |
| TRINITY_DN9619_c0_g1_i1 | 3.48E-05 | 0.602486 | XP_005645155.1 | parvulin-type peptidyl-prolyl cis-trans isomerase [Coccomyxasubellipsoidea C-169] | 1.00E-82 | csl:COCSUDRAFT_54370 | K09578 |
| TRINITY_DN34854_c0_g2_i1 | 1.15E-11 | 0.604723 | XP_005645737.1 | hypothetical protein COCSUDRAFT_57105 [Coccomyxa subellipsoideaC-169] | 1.00E-155 | csl:COCSUDRAFT_57105 | K10597 |
| TRINITY_DN16886_c0_g1_i1 | 0 | 0.60994 | XP_002954857.1 | hypothetical protein VOLCADRAFT_65267 [Volvox carteri f. nagariensis] | 1.00E-103 | vcn:VOLCADRAFT_65267 | K18121 |
| TRINITY_DN5917_c0_g1_i1 | 1.68E-11 | 0.611008 | XP_005850664.1 | hypothetical protein CHLNCDRAFT_140697 [Chlorella variabilis] | 4.00E-38 | cvr:CHLNCDRAFT_140697 | K00899 |
| TRINITY_DN47432_c1_g1_i1 | 4.60E-07 | 0.611556 | XP_002956704.1 | hypothetical protein VOLCADRAFT_77213 [Volvox carteri f.nagariensis] | 2.00E-53 | vcn:VOLCADRAFT_77213 | K00587 |
| TRINITY_DN31535_c1_g1_i3 | 0.0108933 | 0.613279 | XP_002949769.1 | hypothetical protein VOLCADRAFT_90131 [Volvox carteri f. nagariensis] | 4.00E-55 | vcn:VOLCADRAFT_90131 | K04708 |
| TRINITY_DN50853_c0_g1_i1 | 0.000475409 | 0.614219 | XP_002957431.1 | DNA replication factor C complex subunit 2 [Volvox carteri f.nagariensis] | 1.00E-139 | vcn:VOLCADRAFT_84109 | K10755 |
| TRINITY_DN39193_c4_g2_i1 | 0 | 0.615039 | XP_002949136.1 | hypothetical protein VOLCADRAFT_104207 [Volvox carteri f.nagariensis] | 1.00E-116 | vcn:VOLCADRAFT_104207 | K03103 |
| TRINITY_DN13192_c1_g1_i1 | 0.0097656 | 0.615284 | XP_002948852.1 | hypothetical protein VOLCADRAFT_116951, partial [Volvox carteri f.nagariensis] | 1.00E-36 | vcn:VOLCADRAFT_116951 | K19348 |
| TRINITY_DN20086_c0_g1_i1 | 5.66E-06 | 0.615653 | XP_001696648.1 | predicted protein [Chlamydomonas reinhardtii] | 3.00E-37 | cre:CHLREDRAFT_97782 | K10734 |
| TRINITY_DN47160_c1_g2_i2 | 7.41E-06 | 0.616505 | XP_001697418.1 | predicted protein [Chlamydomonas reinhardtii] | 7.00E-38 | cre:CHLREDRAFT_150286 | K10732 |
| TRINITY_DN44169_c1_g1_i3 | 0.0291502 | 0.617026 | XP_002958390.1 | hypothetical protein VOLCADRAFT_99672 [Volvox carteri f. nagariensis] | 2.00E-84 | vcn:VOLCADRAFT_99672 | K10877 |
| TRINITY_DN39193_c4_g1_i1 | 0 | 0.619889 | XP_002949136.1 | hypothetical protein VOLCADRAFT_104207 [Volvox carteri f.nagariensis] | 1.00E-115 | vcn:VOLCADRAFT_104207 | K03103 |
| TRINITY_DN40280_c0_g1_i4 | 0.000492673 | 0.620514 | XP_002948836.1 | hypothetical protein VOLCADRAFT_120666 [Volvox carteri f.nagariensis] | 7.00E-11 | vcn:VOLCADRAFT_120666 | K12900 |
| TRINITY_DN48275_c0_g1_i1 | 0.00765249 | 0.621314 | XP_002947763.1 | hypothetical protein VOLCADRAFT_79791 [Volvox carteri f. nagariensis] | 0 | vcn:VOLCADRAFT_79791 | K03165 |
| TRINITY_DN22991_c1_g1_i1 | 3.24E-12 | 0.624733 | XP_002945891.1 | hypothetical protein VOLCADRAFT_72532 [Volvox carteri f.nagariensis] | 2.00E-52 | vcn:VOLCADRAFT_72532 | K12890 |
| TRINITY_DN27195_c1_g1_i2 | 4.83E-05 | 0.62662 | XP_001695879.1 | DNA replication factor C complex subunit 3 [Chlamydomonasreinhardtii] | 1.00E-141 | cre:CHLREDRAFT_137896 | K10756 |
| TRINITY_DN45805_c3_g3_i1 | 0.00107738 | 0.629166 | XP_005646342.1 | putative DnaJ protein [Coccomyxa subellipsoidea C-169] | 4.00E-61 | csl:COCSUDRAFT_47940 | K09529 |
| TRINITY_DN9028_c0_g2_i1 | 1.39E-08 | 0.629192 | XP_001697282.1 | fructose-6-P aldolase [Chlamydomonas reinhardtii] | 2.00E-55 | cre:CHLREDRAFT_176076 | K00616 |
| TRINITY_DN501_c0_g1_i1 | 0.00268228 | 0.631345 | NP_001237510.1 | uncharacterized protein LOC100499891 [Glycine max] | 8.00E-67 | gmx:100499891 | K06689 |
| TRINITY_DN48635_c2_g1_i1 | 0.000187606 | 0.633527 | XP_005846084.1 | hypothetical protein CHLNCDRAFT_135940 [Chlorella variabilis] | 7.00E-44 | cvr:CHLNCDRAFT_135940 | K10728 |
| TRINITY_DN3626_c0_g2_i1 | 5.36E-09 | 0.634715 | XP_002946471.1 | hypothetical protein VOLCADRAFT_120319 [Volvox carteri f.nagariensis] | 3.00E-28 | vcn:VOLCADRAFT_120319 | K08288 |
| TRINITY_DN37055_c1_g1_i4 | 1.72E-06 | 0.634948 | XP_005650265.1 | hypothetical protein COCSUDRAFT_64802 [Coccomyxa subellipsoideaC-169] | 1.00E-53 | csl:COCSUDRAFT_64802 | K12837 |
| TRINITY_DN6155_c0_g1_i1 | 4.10E-05 | 0.637087 | XP_001697709.1 | hypothetical protein CHLREDRAFT_131472 [Chlamydomonas reinhardtii] | 3.00E-69 | cre:CHLREDRAFT_131472 | K10689 |
| TRINITY_DN22854_c1_g1_i1 | 1.50E-06 | 0.637997 | XP_005646040.1 | DNA mismatch repair protein [Coccomyxa subellipsoidea C-169] | 0 | csl:COCSUDRAFT_37328 | K08735 |
| TRINITY_DN3242_c0_g1_i1 | 0.0347972 | 0.64026 | XP_001695339.1 | predicted protein, partial [Chlamydomonas reinhardtii] | 8.00E-36 | cre:CHLREDRAFT_104097 | K16466 |
| TRINITY_DN53162_c1_g1_i1 | 1.04E-06 | 0.64335 | XP_002955674.1 | RabC/Rab18 [Volvox carteri f. nagariensis] | 1.00E-63 | vcn:VOLCADRAFT_66052 | K07910 |
| TRINITY_DN59941_c0_g1_i1 | 0.00144915 | 0.643927 | XP_001698706.1 | UDP-glucose 4-epimerase [Chlamydomonas reinhardtii] | 3.00E-67 | cre:CHLREDRAFT_139260 | K01784 |
| TRINITY_DN22394_c0_g1_i2 | 0.00469453 | 0.64609 | XP_005851493.1 | hypothetical protein CHLNCDRAFT_137877 [Chlorella variabilis] | 3.00E-11 | cvr:CHLNCDRAFT_137877 | K15075 |
| TRINITY_DN53242_c0_g1_i1 | 4.89E-10 | 0.646338 | XP_002953136.1 | hypothetical protein VOLCADRAFT_105823 [Volvox carteri f.nagariensis] | 1.00E-136 | vcn:VOLCADRAFT_105823 | K11718 |
| TRINITY_DN45811_c2_g2_i3 | 5.67E-07 | 0.64777 | XP_002949344.1 | hypothetical protein VOLCADRAFT_89714 [Volvox carteri f. nagariensis] | 2.00E-37 | vcn:VOLCADRAFT_89714 | K20003 |
| TRINITY_DN45801_c2_g1_i2 | 0.0459919 | 0.648222 | XP_002958969.1 | C type cyclin [Volvox carteri f. nagariensis] | 5.00E-42 | vcn:VOLCADRAFT_127505 | K15161 |
| TRINITY_DN26605_c0_g2_i1 | 2.18E-10 | 0.648917 | XP_005647214.1 | P-loop containing nucleoside triphosphate hydrolase protein[Coccomyxa subellipsoidea C-169] | 8.00E-43 | csl:COCSUDRAFT_42319 | K05643 |
| TRINITY_DN46262_c0_g1_i1 | 5.52E-05 | 0.64899 | XP_005845994.1 | hypothetical protein CHLNCDRAFT_136052 [Chlorella variabilis] | 1.00E-58 | cvr:CHLNCDRAFT_136052 | K07583 |
| TRINITY_DN47867_c15_g2_i1 | 1.62E-08 | 0.650068 | XP_005846304.1 | hypothetical protein CHLNCDRAFT_135675 [Chlorella variabilis] | 1.00E-22 | cvr:CHLNCDRAFT_135675 | K03861 |
| TRINITY_DN42288_c0_g2_i2 | 0.000421171 | 0.650367 | XP_002954087.1 | hypothetical protein VOLCADRAFT_94950 [Volvox carteri f. nagariensis] | 4.00E-89 | vcn:VOLCADRAFT_94950 | K15032 |
| TRINITY_DN12400_c2_g1_i1 | 3.95E-09 | 0.65055 | XP_001689926.1 | predicted protein [Chlamydomonas reinhardtii] | 4.00E-66 | cre:CHLREDRAFT_189746 | K05955 |
| TRINITY_DN803_c0_g2_i1 | 0 | 0.651409 | XP_002953450.1 | nuclease, Rad2 family [Volvox carteri f. nagariensis] | 1.00E-129 | vcn:VOLCADRAFT_75802 | K04799 |
| TRINITY_DN2832_c0_g1_i1 | 0 | 0.65343 | XP_005844095.1 | hypothetical protein CHLNCDRAFT_37095 [Chlorella variabilis] | 1.00E-89 | cvr:CHLNCDRAFT_37095 | K01011 |
| TRINITY_DN48265_c0_g1_i1 | 0.0493727 | 0.654582 | XP_002947552.1 | hypothetical protein VOLCADRAFT_103490 [Volvox carteri f.nagariensis] | 2.00E-48 | vcn:VOLCADRAFT_103490 | K01802 |
| TRINITY_DN28748_c0_g1_i1 | 5.58E-07 | 0.65727 | XP_002957593.1 | isocitrate dehydrogenase, NADP-dependent, mitochondrial [Volvoxcarteri f. nagariensis] | 0 | vcn:VOLCADRAFT_77629 | K00031 |
| TRINITY_DN38439_c4_g1_i1 | 3.35E-07 | 0.658611 | XP_001703298.1 | sulfhydryl oxidase [Chlamydomonas reinhardtii] | 1.00E-43 | cre:CHLREDRAFT_143915 | K17783 |
| TRINITY_DN23058_c6_g1_i4 | 0 | 0.659069 | XP_005843025.1 | hypothetical protein CHLNCDRAFT_59390 [Chlorella variabilis] | 1.00E-76 | cvr:CHLNCDRAFT_59390 | K06569 |
| TRINITY_DN55189_c0_g1_i2 | 0.00374251 | 0.659964 | XP_002956041.1 | hypothetical protein VOLCADRAFT_109993 [Volvox carteri f.nagariensis] | 2.00E-44 | vcn:VOLCADRAFT_109993 | K13249 |
| TRINITY_DN55237_c0_g1_i1 | 9.16E-05 | 0.662338 | XP_005649689.1 | ARF-like small GTPase [Coccomyxa subellipsoidea C-169] | 2.00E-85 | csl:COCSUDRAFT_22483 | K07942 |
| TRINITY_DN37898_c0_g1_i6 | 0.00110865 | 0.667687 | XP_002956213.1 | hypothetical protein VOLCADRAFT_107104 [Volvox carteri f.nagariensis] | 2.00E-35 | vcn:VOLCADRAFT_107104 | K08902 |
| TRINITY_DN43166_c2_g1_i3 | 0.0316886 | 0.669288 | XP_001703127.1 | hypothetical protein CHLREDRAFT_123463, partial [Chlamydomonasreinhardtii] | 1.00E-103 | cre:CHLREDRAFT_123463 | K14664 |
| TRINITY_DN48087_c0_g1_i1 | 0 | 0.670087 | XP_002946415.1 | chaperonin complex component [Volvox carteri f. nagariensis] | 0 | vcn:VOLCADRAFT_108884 | K09493 |
| TRINITY_DN44628_c0_g1_i1 | 0 | 0.670384 | XP_002953831.1 | hypothetical protein VOLCADRAFT_94568 [Volvox carteri f. nagariensis] | 1.00E-50 | vcn:VOLCADRAFT_94568 | K10290 |
| TRINITY_DN52834_c2_g1_i1 | 0 | 0.67233 | XP_002956003.1 | chaperonin complex component [Volvox carteri f. nagariensis] | 0 | vcn:VOLCADRAFT_66389 | K09500 |
| TRINITY_DN36133_c1_g1_i3 | 0.00606314 | 0.67411 | XP_002956099.1 | type VIII myosin heavy chain MyoC [Volvox carteri f. nagariensis] | 3.00E-13 | vcn:VOLCADRAFT_107085 | K10357 |
| TRINITY_DN18063_c0_g2_i1 | 0.000590764 | 0.674983 | XP_011397788.1 | 37S ribosomal protein MRP17, mitochondrial [Auxenochlorellaprotothecoides] | 5.00E-22 | apro:F751_1779 | K02990 |
| TRINITY_DN48147_c1_g1_i1 | 1.44E-08 | 0.675652 | XP_001692428.1 | chloroplast thylakoid processing peptidase [Chlamydomonasreinhardtii] | 2.00E-68 | cre:CHLREDRAFT_183472 | K03100 |
| TRINITY_DN50906_c2_g1_i1 | 0.00150006 | 0.676185 | XP_001693725.1 | cytidine deaminase [Chlamydomonas reinhardtii] | 2.00E-63 | cre:CHLREDRAFT_127415 | K01489 |
| TRINITY_DN30905_c0_g3_i1 | 0 | 0.676618 | XP_001691292.1 | gamma tubulin [Chlamydomonas reinhardtii] | 0 | cre:CHLREDRAFT_188933 | K10389 |
| TRINITY_DN7569_c0_g1_i1 | 1.65E-05 | 0.677368 | XP_001421533.1 | predicted protein, partial [Ostreococcus lucimarinus CCE9901] | 9.00E-61 | olu:OSTLU_4487 | K02604 |
| TRINITY_DN40615_c1_g1_i2 | 0.000362117 | 0.67859 | XP_005844865.1 | hypothetical protein CHLNCDRAFT_138369 [Chlorella variabilis] | 5.00E-57 | cvr:CHLNCDRAFT_138369 | K06130 |
| TRINITY_DN38437_c1_g1_i1 | 0.0495693 | 0.678653 | XP_001690398.1 | hypothetical protein CHLREDRAFT_188432 [Chlamydomonas reinhardtii] | 4.00E-39 | cre:CHLREDRAFT_188432 | K17807 |
| TRINITY_DN3469_c3_g1_i1 | 0.00019778 | 0.678956 | XP_001698646.1 | peptidyl-prolyl cis-trans isomerase, FKBP-type [Chlamydomonasreinhardtii] | 5.00E-51 | cre:CHLREDRAFT_156074 | K01802 |
| TRINITY_DN9067_c0_g1_i1 | 1.61E-10 | 0.680029 | XP_001696917.1 | DNA replication factor C complex subunit 1, partial [Chlamydomonasreinhardtii] | 0 | cre:CHLREDRAFT_150793 | K10754 |
| TRINITY_DN55239_c0_g2_i1 | 0.00538628 | 0.680204 | XP_002953562.1 | hypothetical protein VOLCADRAFT_82311 [Volvox carteri f. nagariensis] | 1.00E-154 | vcn:VOLCADRAFT_82311 | K01963 |
| TRINITY_DN37045_c0_g1_i1 | 0.00420218 | 0.681675 | XP_002952936.1 | hypothetical protein VOLCADRAFT_118225, partial [Volvox carteri f.nagariensis] | 4.00E-40 | vcn:VOLCADRAFT_118225 | K19329 |
| TRINITY_DN46365_c0_g1_i2 | 0.0303818 | 0.681877 | XP_002953915.1 | proline dehydrogenase/oxidase [Volvox carteri f. nagariensis] | 1.00E-99 | vcn:VOLCADRAFT_64330 | K00318 |
| TRINITY_DN32216_c0_g1_i1 | 0 | 0.683325 | XP_002948056.1 | hypothetical protein VOLCADRAFT_79933 [Volvox carteri f. nagariensis] | 1.00E-169 | vcn:VOLCADRAFT_79933 | K01647 |
| TRINITY_DN15818_c0_g2_i1 | 0.00501079 | 0.683741 | XP_001691678.1 | 26S proteasome regulatory subunit [Chlamydomonas reinhardtii] | 0 | cre:CHLREDRAFT_24096 | K03064 |
| TRINITY_DN60218_c1_g8_i1 | 0.00134391 | 0.68377 | XP_002957079.1 | protein disulfide isomerase 1 [Volvox carteri f. nagariensis] | 1.00E-147 | vcn:VOLCADRAFT_77404 | K09580 |
| TRINITY_DN55771_c0_g1_i1 | 0.0105733 | 0.685946 | XP_003596650.1 | eukaryotic translation initiation factor SUI1 [Medicago truncatula] | 2.00E-37 | mtr:MTR_2g083180 | K03113 |
| TRINITY_DN6223_c0_g1_i1 | 1.94E-07 | 0.685986 | XP_001698588.1 | RabGAP/TBC protein [Chlamydomonas reinhardtii] | 7.00E-68 | cre:CHLREDRAFT_1807 | K19953 |
| TRINITY_DN46967_c6_g4_i1 | 3.14E-08 | 0.686464 | XP_005646503.1 | Rft-1-domain-containing protein [Coccomyxa subellipsoidea C-169] | 1.00E-75 | csl:COCSUDRAFT_48002 | K06316 |
| TRINITY_DN22738_c0_g2_i2 | 5.73E-06 | 0.686493 | XP_001691275.1 | polyadenylated-RNA export factor [Chlamydomonas reinhardtii] | 2.00E-50 | cre:CHLREDRAFT_144990 | K18723 |
| TRINITY_DN5850_c0_g2_i1 | 0 | 0.686975 | XP_005651321.1 | hypothetical protein COCSUDRAFT_64653 [Coccomyxa subellipsoideaC-169] | 8.00E-45 | csl:COCSUDRAFT_64653 | K12837 |
| TRINITY_DN57993_c0_g1_i1 | 2.33E-13 | 0.68771 | XP_002955468.1 | hypothetical protein VOLCADRAFT_76727 [Volvox carteri f. nagariensis] | 1.00E-148 | vcn:VOLCADRAFT_76727 | K09272 |
| TRINITY_DN3790_c0_g1_i1 | 3.17E-09 | 0.689061 | XP_002955581.1 | hypothetical protein VOLCADRAFT_96486 [Volvox carteri f.nagariensis] | 2.00E-96 | vcn:VOLCADRAFT_96486 | K17262 |
| TRINITY_DN58038_c2_g1_i1 | 0.000943993 | 0.691171 | XP_005846874.1 | hypothetical protein CHLNCDRAFT_134704 [Chlorella variabilis] | 4.00E-28 | cvr:CHLNCDRAFT_134704 | K10684 |
| TRINITY_DN2067_c0_g1_i1 | 0.00011612 | 0.691551 | XP_002948735.1 | 26S proteasome regulatory complex [Volvox carteri f. nagariensis] | 1.00E-111 | vcn:VOLCADRAFT_80386 | K03037 |
| TRINITY_DN47206_c7_g3_i1 | 0.00262927 | 0.6919 | XP_001700473.1 | SR protein factor [Chlamydomonas reinhardtii] | 1.00E-14 | cre:CHLREDRAFT_195844 | K12896 |
| TRINITY_DN4735_c0_g2_i1 | 0.00190409 | 0.692805 | XP_005646437.1 | PIN domain-like protein [Coccomyxa subellipsoidea C-169] | 3.00E-63 | csl:COCSUDRAFT_17205 | K10746 |
| TRINITY_DN55547_c0_g1_i1 | 1.47E-14 | 0.693912 | XP_001692857.1 | DNA-directed RNA polymerase II, 19 kDa polypeptide [Chlamydomonasreinhardtii] | 6.00E-71 | cre:CHLREDRAFT_79435 | K03022 |
| TRINITY_DN6115_c0_g2_i1 | 0.0421327 | 0.69522 | XP_011400756.1 | Oocyte-specific histone RNA stem-loop-binding protein 2[Auxenochlorella protothecoides] | 2.00E-19 | apro:F751_2760 | K18710 |
| TRINITY_DN60218_c1_g2_i1 | 0.0050994 | 0.695514 | XP_002957079.1 | protein disulfide isomerase 1 [Volvox carteri f. nagariensis] | 1.00E-147 | vcn:VOLCADRAFT_77404 | K09580 |
| TRINITY_DN44764_c0_g3_i1 | 5.22E-08 | 0.697435 | XP_001690217.1 | predicted protein, partial [Chlamydomonas reinhardtii] | 1.00E-128 | cre:CHLREDRAFT_116578 | K11583 |
| TRINITY_DN48172_c0_g1_i1 | 0.0336737 | 0.698474 | XP_002948782.1 | hypothetical protein VOLCADRAFT_103997 [Volvox carteri f.nagariensis] | 9.00E-41 | vcn:VOLCADRAFT_103997 | K12667 |
| TRINITY_DN45588_c1_g1_i1 | 3.11E-11 | 0.698633 | XP_002951152.1 | hypothetical protein VOLCADRAFT_91684 [Volvox carteri f. nagariensis] | 7.00E-41 | vcn:VOLCADRAFT_91684 | K16489 |
| TRINITY_DN42476_c0_g2_i1 | 0.0073938 | 0.700155 | XP_002958660.1 | hypothetical protein VOLCADRAFT_84560 [Volvox carteri f. nagariensis] | 0 | vcn:VOLCADRAFT_84560 | K01937 |
| TRINITY_DN50928_c0_g1_i1 | 2.53E-08 | 0.700536 | XP_002947711.1 | hypothetical protein VOLCADRAFT_79768 [Volvox carteri f. nagariensis] | 1.00E-78 | vcn:VOLCADRAFT_79768 | K12179 |
| TRINITY_DN37488_c1_g2_i1 | 7.43E-05 | 0.700826 | XP_002957453.1 | hypothetical protein VOLCADRAFT_98502 [Volvox carteri f.nagariensis] | 4.00E-53 | vcn:VOLCADRAFT_98502 | K07441 |
| TRINITY_DN50709_c0_g1_i1 | 0.00070258 | 0.701437 | XP_002952671.1 | hypothetical protein VOLCADRAFT_81959 [Volvox carteri f. nagariensis] | 1.00E-153 | vcn:VOLCADRAFT_81959 | K08266 |
| TRINITY_DN29702_c4_g1_i1 | 2.10E-05 | 0.704977 | XP_001702577.1 | inorganic pyrophosphatase [Chlamydomonas reinhardtii] | 1.00E-112 | cre:CHLREDRAFT_133620 | K01507 |
| TRINITY_DN60186_c2_g1_i1 | 2.23E-14 | 0.705511 | XP_001695736.1 | hypothetical protein CHLREDRAFT_192018 [Chlamydomonas reinhardtii] | 2.00E-64 | cre:CHLREDRAFT_192018 | K11324 |
| TRINITY_DN48156_c0_g1_i1 | 6.81E-07 | 0.705867 | XP_005645265.1 | hypothetical protein COCSUDRAFT_18195 [Coccomyxa subellipsoideaC-169] | 1.00E-167 | csl:COCSUDRAFT_18195 | K14455 |
| TRINITY_DN8861_c0_g1_i1 | 0 | 0.706355 | XP_002947190.1 | chaperonin complex component [Volvox carteri f. nagariensis] | 0 | vcn:VOLCADRAFT_79453 | K09496 |
| TRINITY_DN42993_c7_g8_i1 | 5.50E-07 | 0.708781 | XP_005851130.1 | hypothetical protein CHLNCDRAFT_137718 [Chlorella variabilis] | 1.00E-139 | cvr:CHLNCDRAFT_137718 | K01738 |
| TRINITY_DN52883_c0_g1_i1 | 0 | 0.709063 | XP_001697528.1 | T-complex protein, epsilon subunit [Chlamydomonas reinhardtii] | 0 | cre:CHLREDRAFT_131113 | K09497 |
| TRINITY_DN50442_c0_g1_i1 | 0 | 0.710504 | XP_001701443.1 | small nuclear ribonucleoprotein Sm D1, partial [Chlamydomonasreinhardtii] | 4.00E-37 | cre:CHLREDRAFT_122530 | K11087 |
| TRINITY_DN5946_c0_g1_i1 | 7.54E-05 | 0.710865 | XP_001697322.1 | peptidyl-prolyl cis-trans isomerase, cyclophilin-type, partial[Chlamydomonas reinhardtii] | 1.00E-159 | cre:CHLREDRAFT_48580 | K12735 |
| TRINITY_DN50528_c0_g1_i1 | 0 | 0.711822 | XP_002952664.1 | chaperonin complex component [Volvox carteri f. nagariensis] | 0 | vcn:VOLCADRAFT_62848 | K09495 |
| TRINITY_DN25254_c0_g3_i1 | 0.00109699 | 0.711891 | XP_001700009.1 | small ARF-related GTPase [Chlamydomonas reinhardtii] | 6.00E-74 | cre:CHLREDRAFT_195528 | K07950 |
| TRINITY_DN43054_c0_g4_i1 | 0.0108198 | 0.712408 | XP_005851632.1 | hypothetical protein CHLNCDRAFT_138191 [Chlorella variabilis] | 3.00E-66 | cvr:CHLNCDRAFT_138191 | K18999 |
| TRINITY_DN48255_c0_g1_i1 | 1.33E-07 | 0.712702 | XP_001699958.1 | cytokinesis-related protein [Chlamydomonas reinhardtii] | 3.00E-96 | cre:CHLREDRAFT_133976 | K06685 |
| TRINITY_DN13163_c0_g1_i1 | 4.10E-05 | 0.713157 | XP_001700885.1 | Sigma2-Adaptin [Chlamydomonas reinhardtii] | 2.00E-66 | cre:CHLREDRAFT_195448 | K11827 |
| TRINITY_DN60057_c1_g1_i1 | 4.52E-08 | 0.713829 | XP_001696661.1 | S-Adenosylmethionine synthetase [Chlamydomonas reinhardtii] | 0 | cre:CHLREDRAFT_182408 | K00789 |
| TRINITY_DN57629_c0_g1_i1 | 5.63E-06 | 0.714012 | XP_002946857.1 | hypothetical protein VOLCADRAFT_103091 [Volvox carteri f.nagariensis] | 4.00E-25 | vcn:VOLCADRAFT_103091 | K18732 |
| TRINITY_DN50688_c0_g1_i1 | 4.07E-05 | 0.714068 | XP_002958461.1 | hypothetical protein VOLCADRAFT_69480, partial [Volvox carteri f.nagariensis] | 1.00E-106 | vcn:VOLCADRAFT_69480 | K01623 |
| TRINITY_DN38524_c0_g1_i1 | 5.41E-05 | 0.714131 | XP_005850221.1 | hypothetical protein CHLNCDRAFT_34551 [Chlorella variabilis] | 1.00E-179 | cvr:CHLNCDRAFT_34551 | K15683 |
| TRINITY_DN48218_c0_g1_i1 | 1.34E-11 | 0.714269 | XP_005846407.1 | hypothetical protein CHLNCDRAFT_58222 [Chlorella variabilis] | 0 | cvr:CHLNCDRAFT_58222 | K15979 |
| TRINITY_DN16603_c0_g1_i2 | 0.0368278 | 0.714391 | XP_002952902.1 | mitochondrial ribosomal protein S9 [Volvox carteri f. nagariensis] | 1.00E-26 | vcn:VOLCADRAFT_62957 | K02996 |
| TRINITY_DN21833_c0_g1_i1 | 0.0056465 | 0.714512 | XP_005849206.1 | hypothetical protein CHLNCDRAFT_143925 [Chlorella variabilis] | 1.00E-14 | cvr:CHLNCDRAFT_143925 | K03537 |
| TRINITY_DN44984_c1_g1_i1 | 0.0269477 | 0.71619 | XP_002950355.1 | hypothetical protein VOLCADRAFT_90898 [Volvox carteri f. nagariensis] | 1.00E-75 | vcn:VOLCADRAFT_90898 | K18932 |
| TRINITY_DN46642_c8_g13_i1 | 0.0013654 | 0.717984 | XP_001699712.1 | homoserine dehydrogenase [Chlamydomonas reinhardtii] | 1.00E-94 | cre:CHLREDRAFT_196320 | K12524 |
| TRINITY_DN14092_c0_g1_i1 | 0.0411376 | 0.718229 | XP_001699424.1 | protein phosphatase 2A catalytic subunit [Chlamydomonas reinhardtii] | 1.00E-150 | cre:CHLREDRAFT_193562 | K04382 |
| TRINITY_DN42329_c0_g5_i1 | 0.000111986 | 0.719169 | XP_005846273.1 | hypothetical protein CHLNCDRAFT_135599 [Chlorella variabilis] | 5.00E-61 | cvr:CHLNCDRAFT_135599 | K17686 |
| TRINITY_DN24571_c2_g3_i1 | 3.35E-13 | 0.720124 | XP_005651498.1 | ctr family copper transporter [Coccomyxa subellipsoidea C-169] | 9.00E-13 | csl:COCSUDRAFT_32313 | K14686 |
| TRINITY_DN12777_c0_g1_i1 | 0.00977134 | 0.720927 | XP_002955877.1 | hypothetical protein VOLCADRAFT_96758 [Volvox carteri f. nagariensis] | 5.00E-87 | vcn:VOLCADRAFT_96758 | K14301 |
| TRINITY_DN48253_c1_g1_i1 | 2.07E-11 | 0.720966 | XP_001701027.1 | ER DnaJ-like protein 1 [Chlamydomonas reinhardtii] | 1.00E-114 | cre:CHLREDRAFT_134606 | K09517 |
| TRINITY_DN47917_c0_g7_i2 | 0.00287682 | 0.722115 | XP_001689871.1 | glyceraldehyde-3-phosphate dehydrogenase [Chlamydomonasreinhardtii] | 3.00E-37 | cre:CHLREDRAFT_129019 | K05298 |
| TRINITY_DN46311_c14_g1_i1 | 0.0153223 | 0.722549 | XP_001689583.1 | predicted protein [Chlamydomonas reinhardtii] | 2.00E-34 | cre:CHLREDRAFT_171763 | K11858 |
| TRINITY_DN55704_c1_g2_i1 | 0.0295524 | 0.723611 | XP_001694001.1 | glutaredoxin, CPYC type [Chlamydomonas reinhardtii] | 6.00E-21 | cre:CHLREDRAFT_195611 | K03676 |
| TRINITY_DN48386_c0_g1_i1 | 0 | 0.723889 | XP_002948722.1 | eukaryotic translation initiation factor 6 [Volvox carteri f.nagariensis] | 1.00E-112 | vcn:VOLCADRAFT_80373 | K03264 |
| TRINITY_DN47831_c1_g1_i1 | 5.45E-05 | 0.725005 | XP_002947916.1 | hypothetical protein VOLCADRAFT_79954 [Volvox carteri f. nagariensis] | 5.00E-83 | vcn:VOLCADRAFT_79954 | K02257 |
| TRINITY_DN6258_c1_g1_i1 | 1.52E-06 | 0.72686 | XP_002954098.1 | hypothetical protein VOLCADRAFT_106272 [Volvox carteri f.nagariensis] | 1.00E-102 | vcn:VOLCADRAFT_106272 | K00852 |
| TRINITY_DN27163_c3_g1_i2 | 5.33E-07 | 0.728245 | XP_001695300.1 | aspartyl-tRNA synthetase [Chlamydomonas reinhardtii] | 0 | cre:CHLREDRAFT_191617 | K01876 |
| TRINITY_DN43175_c1_g1_i1 | 0.00197237 | 0.728434 | XP_001695465.1 | Rh protein [Chlamydomonas reinhardtii] | 1.00E-128 | cre:CHLREDRAFT_24240 | K06580 |
| TRINITY_DN32582_c0_g2_i3 | 0.000387474 | 0.729618 | XP_002948700.1 | hypothetical protein VOLCADRAFT_89087 [Volvox carteri f.nagariensis] | 2.00E-26 | vcn:VOLCADRAFT_89087 | K17428 |
| TRINITY_DN46603_c1_g7_i1 | 0.0219649 | 0.729957 | NP_215622.1 | 3 beta-hydroxysteroid dehydrogenase/delta 5 [Mycobacterium tuberculosis] | 3.00E-68 | mtu:Rv1106c | K16045 |
| TRINITY_DN34423_c4_g4_i1 | 1.02E-08 | 0.730732 | XP_001696992.1 | RNA binding motif protein [Chlamydomonas reinhardtii] | 2.00E-44 | cre:CHLREDRAFT_192599 | K12876 |
| TRINITY_DN46610_c1_g1_i1 | 0.0425201 | 0.731544 | XP_005848807.1 | hypothetical protein CHLNCDRAFT_144082 [Chlorella variabilis] | 1.00E-113 | cvr:CHLNCDRAFT_144082 | K09313 |
| TRINITY_DN12319_c1_g1_i1 | 5.48E-10 | 0.732967 | XP_002946387.1 | hypothetical protein VOLCADRAFT_108879 [Volvox carteri f.nagariensis] | 3.00E-61 | vcn:VOLCADRAFT_108879 | K00794 |
| TRINITY_DN9866_c4_g1_i1 | 2.78E-15 | 0.73309 | XP_005846820.1 | hypothetical protein CHLNCDRAFT_24556 [Chlorella variabilis] | 1.00E-95 | cvr:CHLNCDRAFT_24556 | K11407 |
| TRINITY_DN55805_c0_g1_i1 | 6.18E-10 | 0.734122 | XP_002950603.1 | hypothetical protein VOLCADRAFT_104739 [Volvox carteri f.nagariensis] | 6.00E-80 | vcn:VOLCADRAFT_104739 | K19372 |
| TRINITY_DN22548_c0_g2_i2 | 9.77E-05 | 0.734442 | XP_002953998.1 | hypothetical protein VOLCADRAFT_76054 [Volvox carteri f.nagariensis] | 3.00E-51 | vcn:VOLCADRAFT_76054 | K07305 |
| TRINITY_DN3602_c0_g1_i1 | 0.0143501 | 0.734897 | XP_001695405.1 | ethanolamine kinase, partial [Chlamydomonas reinhardtii] | 4.00E-18 | cre:CHLREDRAFT_118793 | K00894 |
| TRINITY_DN52921_c0_g1_i1 | 6.82E-05 | 0.735211 | XP_001698004.1 | UDP-glucose dehydrogenase [Chlamydomonas reinhardtii] | 0 | cre:CHLREDRAFT_185081 | K00012 |
| TRINITY_DN55663_c0_g1_i1 | 0.0107321 | 0.735242 | NP_194914.1 | putative protein phosphatase 2C 60 [Arabidopsis thaliana] | 5.00E-39 | ath:AT4G31860 | K17499 |
| TRINITY_DN52904_c0_g1_i1 | 2.15E-08 | 0.735381 | XP_001693266.1 | eukaryotic initiation factor [Chlamydomonas reinhardtii] | 1.00E-141 | cre:CHLREDRAFT_79571 | K03246 |
| TRINITY_DN60491_c0_g1_i1 | 0.00917977 | 0.736218 | XP_001690982.1 | pre-mRNA splicing factor, partial [Chlamydomonas reinhardtii] | 1.00E-78 | cre:CHLREDRAFT_135470 | K12849 |
| TRINITY_DN14326_c0_g3_i1 | 3.18E-05 | 0.736612 | XP_002947800.1 | hypothetical protein VOLCADRAFT_103581 [Volvox carteri f.nagariensis] | 1.00E-63 | vcn:VOLCADRAFT_103581 | K11094 |
| TRINITY_DN52980_c0_g1_i1 | 0.000175998 | 0.73672 | XP_005644126.1 | LEDI-3 protein [Coccomyxa subellipsoidea C-169] | 4.00E-68 | csl:COCSUDRAFT_31027 | K03809 |
| TRINITY_DN28232_c1_g2_i1 | 0 | 0.736907 | XP_003074444.1 | calmodulin mutant SYNCAM9 (ISS) [Ostreococcus tauri] | 4.00E-21 | ota:Ot01g03840 | K02183 |
| TRINITY_DN42564_c1_g2_i3 | 1.34E-05 | 0.737189 | XP_001696276.1 | hypothetical protein CHLREDRAFT_112697, partial [Chlamydomonasreinhardtii] | 3.00E-89 | cre:CHLREDRAFT_112697 | K13412 |
| TRINITY_DN47892_c11_g1_i2 | 0.0303856 | 0.737726 | XP_001702393.1 | flagellar associated protein, partial [Chlamydomonas reinhardtii] | 0 | cre:CHLREDRAFT_194683 | K15426 |
| TRINITY_DN55482_c0_g1_i1 | 0 | 0.739144 | XP_001696757.1 | T-complex protein 1, beta subunit [Chlamydomonas reinhardtii] | 0 | cre:CHLREDRAFT_184701 | K09494 |
| TRINITY_DN35005_c1_g1_i1 | 0.017758 | 0.73968 | XP_002949533.1 | hypothetical protein VOLCADRAFT_104322 [Volvox carteri f.nagariensis] | 2.00E-28 | vcn:VOLCADRAFT_104322 | K03946 |
| TRINITY_DN44697_c0_g3_i1 | 0.0301448 | 0.739787 | XP_002952378.1 | hypothetical protein VOLCADRAFT_105503 [Volvox carteri f.nagariensis] | 1.00E-57 | vcn:VOLCADRAFT_105503 | K14307 |
| TRINITY_DN34672_c0_g1_i2 | 1.19E-07 | 0.740525 | XP_001693297.1 | predicted protein [Chlamydomonas reinhardtii] | 2.00E-52 | cre:CHLREDRAFT_190484 | K14315 |
| TRINITY_DN50740_c0_g2_i1 | 0.0149264 | 0.742684 | XP_002955952.1 | hypothetical protein VOLCADRAFT_107008 [Volvox carteri f.nagariensis] | 1.00E-123 | vcn:VOLCADRAFT_107008 | K19269 |
| TRINITY_DN13044_c0_g2_i1 | 0.0046341 | 0.742846 | XP_002948090.1 | hypothetical protein VOLCADRAFT_79982 [Volvox carteri f. nagariensis] | 1.00E-46 | vcn:VOLCADRAFT_79982 | K19706 |
| TRINITY_DN48296_c0_g1_i1 | 4.74E-05 | 0.744788 | XP_001690376.1 | mitochondrial substrate carrier [Chlamydomonas reinhardtii] | 2.00E-96 | cre:CHLREDRAFT_169342 | K14684 |
| TRINITY_DN55761_c2_g1_i1 | 2.29E-09 | 0.744992 | XP_001699077.1 | superoxide dismutase [Mn] [Chlamydomonas reinhardtii] | 8.00E-81 | cre:CHLREDRAFT_193511 | K04564 |
| TRINITY_DN46188_c1_g1_i1 | 1.22E-07 | 0.74551 | XP_002956779.1 | hypothetical protein VOLCADRAFT_110025 [Volvox carteri f.nagariensis] | 2.00E-81 | vcn:VOLCADRAFT_110025 | K01519 |
| TRINITY_DN38644_c0_g1_i1 | 0.00288717 | 0.745691 | XP_002950203.1 | hypothetical protein VOLCADRAFT_104598 [Volvox carteri f.nagariensis] | 1.00E-48 | vcn:VOLCADRAFT_104598 | K12417 |
| TRINITY_DN47343_c0_g1_i2 | 1.41E-05 | 0.746697 | XP_002508596.1 | ytidine and deoxycytidylated deaminase zinc-binding protein[Micromonas sp. RCC299] | 7.00E-46 | mis:MICPUN_112737 | K01493 |
| TRINITY_DN5742_c0_g1_i1 | 2.15E-13 | 0.747309 | XP_001700556.1 | serine/threonine-protein phosphatase PP2A-3 catalytic subunit[Chlamydomonas reinhardtii] | 1.00E-160 | cre:CHLREDRAFT_132486 | K15423 |
| TRINITY_DN3575_c0_g1_i1 | 0.0479486 | 0.747311 | XP_002948082.1 | hypothetical protein VOLCADRAFT_120546 [Volvox carteri f.nagariensis] | 1.00E-178 | vcn:VOLCADRAFT_120546 | K01962 |
| TRINITY_DN45769_c6_g3_i2 | 5.50E-08 | 0.748168 | XP_009419590.1 | PREDICTED: glutamate receptor 3.5-like [Musa acuminata subsp.malaccensis] | 1.00E-13 | mus:103999537 | K05387 |
| TRINITY_DN6230_c1_g1_i1 | 9.09E-09 | 0.749602 | XP_011397225.1 | putative U6 snRNA-associated Sm-like protein LSm4 [Auxenochlorellaprotothecoides] | 3.00E-37 | apro:F751_3055 | K12623 |
| TRINITY_DN8774_c0_g2_i1 | 0.0301746 | 0.749993 | XP_001772308.1 | predicted protein, partial [Physcomitrella patens] | 2.00E-27 | ppp:PHYPADRAFT_18452 | K15153 |
| TRINITY_DN8608_c0_g1_i1 | 1.23E-06 | 0.750346 | XP_002951825.1 | hypothetical protein VOLCADRAFT_105238 [Volvox carteri f.nagariensis] | 5.00E-22 | vcn:VOLCADRAFT_105238 | K09549 |
| TRINITY_DN187_c0_g2_i1 | 0.0115023 | 0.750401 | XP_001698874.1 | hypothetical protein CHLREDRAFT_159339 [Chlamydomonas reinhardtii] | 6.00E-24 | cre:CHLREDRAFT_159339 | K18172 |
| TRINITY_DN57748_c0_g1_i1 | 0.0140006 | 0.750457 | XP_002956096.1 | hypothetical protein VOLCADRAFT_97089 [Volvox carteri f.nagariensis] | 2.00E-14 | vcn:VOLCADRAFT_97089 | K07342 |
| TRINITY_DN57617_c2_g1_i1 | 0.02068 | 0.751176 | XP_011462945.1 | PREDICTED: protein decapping 5 [Fragaria vesca subsp. vesca] | 4.00E-23 | fve:101308565 | K18749 |
| TRINITY_DN47945_c2_g3_i1 | 0 | 0.751639 | XP_002955338.1 | mitogen-activated protein kinase 8 [Volvox carteri f. nagariensis] | 1.00E-159 | vcn:VOLCADRAFT_76648 | K14512 |
| TRINITY_DN24484_c1_g1_i1 | 8.91E-05 | 0.751685 | XP_005645557.1 | thioredoxin-domain-containing protein [Coccomyxa subellipsoideaC-169] | 3.00E-56 | csl:COCSUDRAFT_54093 | K09584 |
| TRINITY_DN48413_c0_g1_i1 | 8.92E-06 | 0.752491 | XP_005845875.1 | hypothetical protein CHLNCDRAFT_59731 [Chlorella variabilis] | 2.00E-38 | cvr:CHLNCDRAFT_59731 | K03358 |
| TRINITY_DN45937_c2_g2_i1 | 0.000136959 | 0.753021 | XP_002945691.1 | hypothetical protein VOLCADRAFT_78672 [Volvox carteri f. nagariensis] | 1.00E-125 | vcn:VOLCADRAFT_78672 | K06013 |
| TRINITY_DN52952_c0_g1_i1 | 0.000813977 | 0.75335 | XP_001768763.1 | predicted protein [Physcomitrella patens] | 5.00E-60 | ppp:PHYPADRAFT_232560 | K15407 |
| TRINITY_DN50578_c0_g2_i1 | 0 | 0.7538 | XP_002955536.1 | hypothetical protein VOLCADRAFT_109972 [Volvox carteri f.nagariensis] | 1.00E-153 | vcn:VOLCADRAFT_109972 | K10355 |
| TRINITY_DN41519_c0_g1_i2 | 0.00654615 | 0.754166 | XP_001698472.1 | predicted protein, partial [Chlamydomonas reinhardtii] | 0 | cre:CHLREDRAFT_134676 | K20032 |
| TRINITY_DN48117_c0_g1_i1 | 7.29E-14 | 0.754216 | XP_001702638.1 | 20S proteasome alpha subunit C [Chlamydomonas reinhardtii] | 1.00E-104 | cre:CHLREDRAFT_133457 | K02728 |
| TRINITY_DN37280_c1_g2_i1 | 0.000589185 | 0.754462 | XP_002950550.1 | hypothetical protein VOLCADRAFT_91063 [Volvox carteri f.nagariensis] | 1.00E-59 | vcn:VOLCADRAFT_91063 | K12834 |
| TRINITY_DN55701_c2_g1_i1 | 1.76E-09 | 0.755314 | XP_001693876.1 | predicted 5-formyltetrahydrofolate cycloligase, partial[Chlamydomonas reinhardtii] | 1.00E-109 | cre:CHLREDRAFT_127515 | K01934 |
| TRINITY_DN47330_c1_g2_i2 | 0.0283824 | 0.755491 | XP_001700282.1 | predicted protein [Chlamydomonas reinhardtii] | 8.00E-87 | cre:CHLREDRAFT_152884 | K13985 |
| TRINITY_DN57847_c0_g1_i1 | 4.47E-05 | 0.755495 | XP_001691599.1 | predicted protein [Chlamydomonas reinhardtii] | 0 | cre:CHLREDRAFT_170459 | K17491 |
| TRINITY_DN3641_c0_g2_i1 | 6.74E-06 | 0.755902 | XP_002947107.1 | hypothetical protein VOLCADRAFT_103327 [Volvox carteri f.nagariensis] | 1.00E-173 | vcn:VOLCADRAFT_103327 | K10393 |
| TRINITY_DN35250_c3_g2_i1 | 0.00122005 | 0.756655 | XP_002953833.1 | small Arf-related GTPase [Volvox carteri f. nagariensis] | 3.00E-79 | vcn:VOLCADRAFT_64069 | K07943 |
| TRINITY_DN57664_c0_g1_i1 | 1.44E-10 | 0.756787 | XP_001690306.1 | signal peptidase, 22 kDa subunit [Chlamydomonas reinhardtii] | 2.00E-47 | cre:CHLREDRAFT_190234 | K12948 |
| TRINITY_DN36401_c0_g1_i2 | 0.00111606 | 0.757531 | XP_005649547.1 | hypothetical protein COCSUDRAFT_46554 [Coccomyxa subellipsoideaC-169] | 2.00E-55 | csl:COCSUDRAFT_46554 | K03125 |
| TRINITY_DN21355_c0_g1_i2 | 1.57E-05 | 0.75767 | XP_001691830.1 | hypothetical protein CHLREDRAFT_115524, partial [Chlamydomonasreinhardtii] | 1.00E-93 | cre:CHLREDRAFT_115524 | K15698 |
| TRINITY_DN57738_c1_g1_i1 | 1.76E-07 | 0.758377 | XP_002956171.1 | hypothetical protein VOLCADRAFT_83518 [Volvox carteri f.nagariensis] | 8.00E-74 | vcn:VOLCADRAFT_83518 | K10573 |
| TRINITY_DN19130_c1_g2_i1 | 6.98E-09 | 0.758931 | XP_001692856.1 | CDP-Ethanolamine:DAG ethanolamine phosphotransferase [Chlamydomonasreinhardtii] | 1.00E-77 | cre:CHLREDRAFT_79396 | K00993 |
| TRINITY_DN60098_c0_g1_i1 | 0 | 0.759432 | XP_002955253.1 | chaperonin complex component [Volvox carteri f. nagariensis] | 0 | vcn:VOLCADRAFT_65637 | K09498 |
| TRINITY_DN44381_c0_g1_i3 | 8.88E-05 | 0.759823 | XP_005851159.1 | hypothetical protein CHLNCDRAFT_29501 [Chlorella variabilis] | 2.00E-83 | cvr:CHLNCDRAFT_29501 | K12571 |
| TRINITY_DN42690_c0_g1_i1 | 4.45E-05 | 0.760355 | XP_001702707.1 | tRNA uridine 5-carboxymethylaminomethyl modification enzyme, partial[Chlamydomonas reinhardtii] | 0 | cre:CHLREDRAFT_38547 | K03495 |
| TRINITY_DN4709_c0_g1_i1 | 0.0473484 | 0.760448 | XP_005649768.1 | acetyl-CoA biotin carboxyl carrier [Coccomyxa subellipsoidea C-169] | 2.00E-34 | csl:COCSUDRAFT_65159 | K02160 |
| TRINITY_DN46047_c0_g2_i1 | 0.0403631 | 0.760706 | XP_002954137.1 | rRNA processing protein Rrp5/programmed cell death protein 11 [Volvoxcarteri f. nagariensis] | 6.00E-82 | vcn:VOLCADRAFT_94877 | K14792 |
| TRINITY_DN10407_c0_g1_i1 | 7.53E-10 | 0.762786 | XP_008811208.1 | PREDICTED: actin-related protein 4 [Phoenix dactylifera] | 1.00E-114 | pda:103722428 | K11340 |
| TRINITY_DN35763_c1_g1_i2 | 9.61E-11 | 0.763861 | XP_002954156.1 | hypothetical protein VOLCADRAFT_82641 [Volvox carteri f.nagariensis] | 2.00E-98 | vcn:VOLCADRAFT_82641 | K00472 |
| TRINITY_DN55463_c0_g1_i1 | 3.34E-06 | 0.764689 | XP_002951029.1 | eukaryotic translation initiation factor 2B gamma [Volvox carteri f.nagariensis] | 1.00E-134 | vcn:VOLCADRAFT_120926 | K03241 |
| TRINITY_DN58065_c0_g1_i1 | 1.76E-06 | 0.764723 | XP_002952992.1 | cellular apoptosis susceptibility/chromosome segregation 1-likeprotein [Volvox carteri f. nagariensis] | 0 | vcn:VOLCADRAFT_82084 | K18423 |
| TRINITY_DN46805_c4_g10_i1 | 0.0185091 | 0.76528 | XP_002952264.1 | hypothetical protein VOLCADRAFT_81770 [Volvox carteri f.nagariensis] | 1.00E-55 | vcn:VOLCADRAFT_81770 | K12180 |
| TRINITY_DN8727_c0_g1_i1 | 3.04E-07 | 0.765533 | XP_002948091.1 | hypothetical protein VOLCADRAFT_73556 [Volvox carteri f. nagariensis] | 2.00E-97 | vcn:VOLCADRAFT_73556 | K03679 |
| TRINITY_DN9643_c0_g4_i1 | 0 | 0.765899 | XP_002955019.1 | hypothetical protein VOLCADRAFT_106614 [Volvox carteri f.nagariensis] | 8.00E-42 | vcn:VOLCADRAFT_106614 | K12603 |
| TRINITY_DN46868_c0_g2_i1 | 0.000636438 | 0.765957 | XP_005645218.1 | polymeras-like protein II polypeptide D [Coccomyxa subellipsoideaC-169] | 5.00E-13 | csl:COCSUDRAFT_54196 | K03012 |
| TRINITY_DN29702_c4_g2_i1 | 0.000111812 | 0.766328 | XP_002954091.1 | hypothetical protein VOLCADRAFT_82661 [Volvox carteri f.nagariensis] | 2.00E-99 | vcn:VOLCADRAFT_82661 | K01507 |
| TRINITY_DN15337_c0_g1_i2 | 0.000363463 | 0.766401 | XP_002950017.1 | hypothetical protein VOLCADRAFT_59795 [Volvox carteri f.nagariensis] | 2.00E-66 | vcn:VOLCADRAFT_59795 | K14397 |
| TRINITY_DN3364_c0_g1_i2 | 0.0220664 | 0.766843 | XP_005648152.1 | TPR-like protein [Coccomyxa subellipsoidea C-169] | 5.00E-54 | csl:COCSUDRAFT_15316 | K03353 |
| TRINITY_DN55583_c3_g1_i1 | 0 | 0.768825 | XP_001698736.1 | mitochondrial F1F0 ATP synthase, delta subunit [Chlamydomonasreinhardtii] | 5.00E-48 | cre:CHLREDRAFT_185200 | K02134 |
| TRINITY_DN57950_c0_g2_i1 | 2.98E-05 | 0.769318 | XP_006288716.1 | hypothetical protein CARUB_v10002027mg [Capsella rubella] | 3.00E-16 | crb:CARUB_v10002027mg | K11095 |
| TRINITY_DN50604_c0_g1_i1 | 5.82E-05 | 0.769871 | XP_001699044.1 | dolichyl-diphosphooligosaccharide-protein glycosyltransferase[Chlamydomonas reinhardtii] | 1.00E-120 | cre:CHLREDRAFT_121156 | K12670 |
| TRINITY_DN44151_c0_g1_i2 | 0.0128568 | 0.769954 | XP_001699267.1 | heavy metal transporting ATPase [Chlamydomonas reinhardtii] | 0 | cre:CHLREDRAFT_206047 | K17686 |
| TRINITY_DN44001_c2_g3_i1 | 0.00329073 | 0.770411 | XP_005642617.1 | hypothetical protein COCSUDRAFT_26470 [Coccomyxa subellipsoideaC-169] | 0 | csl:COCSUDRAFT_26470 | K11262 |
| TRINITY_DN58055_c0_g1_i1 | 0.0194916 | 0.77043 | XP_002953232.1 | hypothetical protein VOLCADRAFT_118302, partial [Volvox carteri f.nagariensis] | 2.00E-47 | vcn:VOLCADRAFT_118302 | K13335 |
| TRINITY_DN57826_c2_g1_i1 | 1.05E-11 | 0.770821 | XP_005847779.1 | hypothetical protein CHLNCDRAFT_133913 [Chlorella variabilis] | 1.00E-125 | cvr:CHLNCDRAFT_133913 | K00765 |
| TRINITY_DN60217_c0_g1_i1 | 0.0428022 | 0.77098 | XP_002955859.1 | fatty acid desaturase, delta-12 [Volvox carteri f. nagariensis] | 1.00E-150 | vcn:VOLCADRAFT_66373 | K10256 |
| TRINITY_DN60277_c1_g1_i1 | 3.49E-07 | 0.771156 | XP_002951811.1 | iron-sulfur cluster assembly protein [Volvox carteri f. nagariensis] | 1.00E-106 | vcn:VOLCADRAFT_75135 | K09013 |
| TRINITY_DN47064_c4_g1_i3 | 0.00167375 | 0.77121 | XP_002951805.1 | hypothetical protein VOLCADRAFT_117940 [Volvox carteri f.nagariensis] | 1.00E-105 | vcn:VOLCADRAFT_117940 | K14050 |
| TRINITY_DN47255_c19_g16_i1 | 0.00713082 | 0.771272 | XP_001690981.1 | phosphoribosylaminoimidazole carboxylase, eukaryotic-type[Chlamydomonas reinhardtii] | 0 | cre:CHLREDRAFT_196726 | K11808 |
| TRINITY_DN29774_c0_g1_i4 | 1.39E-06 | 0.772033 | XP_001698334.1 | molybdenum cofactor biosynthesis protein [Chlamydomonas reinhardtii] | 1.00E-149 | cre:CHLREDRAFT_113296 | K15376 |
| TRINITY_DN55310_c0_g1_i1 | 2.88E-14 | 0.772425 | XP_002956005.1 | hypothetical protein VOLCADRAFT_76912 [Volvox carteri f.nagariensis] | 2.00E-67 | vcn:VOLCADRAFT_76912 | K07575 |
| TRINITY_DN47674_c7_g1_i1 | 0.0328259 | 0.7757 | XP_002958345.1 | hypothetical protein VOLCADRAFT_99613 [Volvox carteri f. nagariensis] | 6.00E-27 | vcn:VOLCADRAFT_99613 | K08857 |
| TRINITY_DN22531_c0_g2_i1 | 0.0352436 | 0.777204 | XP_005851362.1 | hypothetical protein CHLNCDRAFT_137564 [Chlorella variabilis] | 3.00E-53 | cvr:CHLNCDRAFT_137564 | K10703 |
| TRINITY_DN48586_c1_g1_i1 | 0.00146031 | 0.777403 | XP_001698652.1 | hypothetical protein CHLREDRAFT_185179 [Chlamydomonas reinhardtii] | 1.00E-19 | cre:CHLREDRAFT_185179 | K11878 |
| TRINITY_DN16660_c3_g1_i1 | 0 | 0.778797 | XP_001697869.1 | histone H3 variant [Chlamydomonas reinhardtii] | 7.00E-50 | cre:CHLREDRAFT_185125 | K11253 |
| TRINITY_DN46459_c2_g3_i1 | 0.0119102 | 0.779425 | XP_005851298.1 | hypothetical protein CHLNCDRAFT_33921 [Chlorella variabilis] | 1.00E-47 | cvr:CHLNCDRAFT_33921 | K03016 |
| TRINITY_DN27099_c4_g1_i1 | 2.17E-05 | 0.779891 | XP_005851544.1 | hypothetical protein CHLNCDRAFT_56758 [Chlorella variabilis] | 3.00E-45 | cvr:CHLNCDRAFT_56758 | K01522 |
| TRINITY_DN9076_c0_g3_i1 | 1.45E-07 | 0.780176 | XP_005650118.1 | ubiquinone biosynthesis protein COQ9 [Coccomyxa subellipsoidea C-169] | 2.00E-39 | csl:COCSUDRAFT_32690 | K18587 |
| TRINITY_DN23048_c2_g1_i1 | 0.00532514 | 0.780289 | XP_002505939.1 | predicted protein [Micromonas sp. RCC299] | 2.00E-76 | mis:MICPUN_98314 | K17261 |
| TRINITY_DN48054_c0_g1_i1 | 1.41E-05 | 0.780573 | XP_002955536.1 | hypothetical protein VOLCADRAFT_109972 [Volvox carteri f.nagariensis] | 0 | vcn:VOLCADRAFT_109972 | K10355 |
| TRINITY_DN60052_c0_g1_i1 | 0 | 0.780666 | XP_001701305.1 | 20S proteasome alpha subunit B [Chlamydomonas reinhardtii] | 1.00E-108 | cre:CHLREDRAFT_194387 | K02726 |
| TRINITY_DN60535_c1_g1_i1 | 3.10E-06 | 0.78088 | XP_001695659.1 | U6 small ribonucleoprotein F [Chlamydomonas reinhardtii] | 6.00E-33 | cre:CHLREDRAFT_130885 | K12625 |
| TRINITY_DN3512_c1_g1_i1 | 5.88E-07 | 0.78119 | XP_001701241.1 | phosphoserine phosphatase [Chlamydomonas reinhardtii] | 9.00E-80 | cre:CHLREDRAFT_194322 | K01079 |
| TRINITY_DN48069_c3_g1_i1 | 4.84E-05 | 0.781671 | XP_001700921.1 | translocon-associated protein beta [Chlamydomonas reinhardtii] | 1.00E-27 | cre:CHLREDRAFT_182433 | K13250 |
| TRINITY_DN55543_c0_g1_i1 | 0.00146714 | 0.78204 | XP_005644985.1 | hypothetical protein COCSUDRAFT_43885 [Coccomyxa subellipsoideaC-169] | 2.00E-42 | csl:COCSUDRAFT_43885 | K13024 |
| TRINITY_DN50602_c1_g1_i1 | 0.00566702 | 0.78209 | XP_001692033.1 | mitochondrial inner membrane translocase [Chlamydomonasreinhardtii] | 2.00E-26 | cre:CHLREDRAFT_196559 | K17780 |
| TRINITY_DN47756_c0_g2_i1 | 0.000191446 | 0.78244 | XP_001703013.1 | predicted protein [Chlamydomonas reinhardtii] | 3.00E-44 | cre:CHLREDRAFT_123186 | K06287 |
| TRINITY_DN20047_c0_g1_i1 | 0.0119272 | 0.783596 | XP_002953517.1 | hypothetical protein VOLCADRAFT_63817 [Volvox carteri f. nagariensis] | 1.00E-155 | vcn:VOLCADRAFT_63817 | K11717 |
| TRINITY_DN47957_c27_g32_i1 | 1.10E-12 | 0.784071 | XP_001701515.1 | peroxisomal targeting signal 2 receptor [Chlamydomonas reinhardtii] | 1.00E-138 | cre:CHLREDRAFT_132770 | K13341 |
| TRINITY_DN19077_c0_g6_i1 | 6.01E-06 | 0.784261 | XP_005849895.1 | hypothetical protein CHLNCDRAFT_34758 [Chlorella variabilis] | 6.00E-70 | cvr:CHLNCDRAFT_34758 | K15731 |
| TRINITY_DN43314_c5_g1_i1 | 0.0140257 | 0.784875 | XP_002957217.1 | alpha tubulin [Volvox carteri f. nagariensis] | 0 | vcn:VOLCADRAFT_77526 | K07374 |
| TRINITY_DN22026_c0_g1_i1 | 3.66E-15 | 0.784995 | XP_001699757.1 | cell division cycle protein 14, partial [Chlamydomonas reinhardtii] | 1.00E-130 | cre:CHLREDRAFT_112184 | K06639 |
| TRINITY_DN12975_c0_g1_i1 | 0.00773505 | 0.785576 | XP_001696196.1 | hypothetical protein CHLREDRAFT_167044 [Chlamydomonas reinhardtii] | 1.00E-118 | cre:CHLREDRAFT_167044 | K14641 |
| TRINITY_DN10703_c0_g1_i1 | 4.24E-05 | 0.786558 | XP_005648190.1 | glyoxalase I [Coccomyxa subellipsoidea C-169] | 1.00E-100 | csl:COCSUDRAFT_23589 | K01759 |
| TRINITY_DN8739_c0_g2_i1 | 0.00709145 | 0.786884 | XP_001702729.1 | ubiquitin-related modifier [Chlamydomonas reinhardtii] | 1.00E-31 | cre:CHLREDRAFT_111518 | K12161 |
| TRINITY_DN10927_c0_g2_i1 | 9.05E-07 | 0.787193 | XP_002952633.1 | hypothetical protein VOLCADRAFT_81927 [Volvox carteri f. nagariensis] | 1.00E-143 | vcn:VOLCADRAFT_81927 | K09613 |
| TRINITY_DN25034_c0_g1_i1 | 6.66E-05 | 0.787957 | XP_001692245.1 | hypothetical protein CHLREDRAFT_145893, partial [Chlamydomonasreinhardtii] | 8.00E-94 | cre:CHLREDRAFT_145893 | K15451 |
| TRINITY_DN42043_c1_g1_i1 | 0 | 0.787972 | XP_005643284.1 | hypothetical protein COCSUDRAFT_38543 [Coccomyxa subellipsoideaC-169] | 0 | csl:COCSUDRAFT_38543 | K12483 |
| TRINITY_DN50427_c0_g1_i1 | 7.41E-12 | 0.787977 | XP_001699641.1 | mitochondrial F1F0 ATP synthase, alpha subunit [Chlamydomonasreinhardtii] | 0 | cre:CHLREDRAFT_76602 | K02132 |
| TRINITY_DN48009_c3_g2_i1 | 2.59E-09 | 0.788081 | XP_001695876.1 | hypothetical protein CHLREDRAFT_137847, partial [Chlamydomonasreinhardtii] | 8.00E-68 | cre:CHLREDRAFT_137847 | K11092 |
| TRINITY_DN60017_c1_g1_i1 | 3.35E-08 | 0.78831 | XP_001690422.1 | signal peptide peptidase, eukaryotic-type [Chlamydomonas reinhardtii] | 1.00E-107 | cre:CHLREDRAFT_206029 | K09595 |
| TRINITY_DN6366_c0_g1_i1 | 0.000213266 | 0.788526 | XP_002946971.1 | hypothetical protein VOLCADRAFT_72975 [Volvox carteri f. nagariensis] | 0 | vcn:VOLCADRAFT_72975 | K10956 |
| TRINITY_DN57594_c3_g1_i1 | 0 | 0.788888 | XP_005650255.1 | threonyl-tRNA synthetase [Coccomyxa subellipsoidea C-169] | 6.00E-90 | csl:COCSUDRAFT_64793 | K01868 |
| TRINITY_DN17490_c0_g1_i1 | 3.51E-05 | 0.789443 | XP_011099215.1 | PREDICTED: upstream activation factor subunit spp27-like isoform X1[Sesamum indicum] | 7.00E-43 | sind:105177679 | K15223 |
| TRINITY_DN53181_c1_g1_i1 | 2.03E-06 | 0.789684 | XP_002499541.1 | predicted protein [Micromonas sp. RCC299] | 5.00E-56 | mis:MICPUN_78442 | K00943 |
| TRINITY_DN8759_c0_g1_i1 | 0.000252352 | 0.789785 | XP_001698706.1 | UDP-glucose 4-epimerase [Chlamydomonas reinhardtii] | 1.00E-154 | cre:CHLREDRAFT_139260 | K01784 |
| TRINITY_DN52992_c0_g1_i1 | 0.00110201 | 0.790738 | XP_002955670.1 | hypothetical protein VOLCADRAFT_76813 [Volvox carteri f.nagariensis] | 2.00E-25 | vcn:VOLCADRAFT_76813 | K08762 |
| TRINITY_DN2142_c0_g1_i1 | 3.79E-08 | 0.790949 | XP_001698146.1 | dolichol-phosphate glucosyltransferase [Chlamydomonas reinhardtii] | 1.00E-101 | cre:CHLREDRAFT_18353 | K00729 |
| TRINITY_DN46954_c0_g2_i1 | 6.51E-07 | 0.791124 | XP_001689570.1 | predicted protein, partial [Chlamydomonas reinhardtii] | 4.00E-58 | cre:CHLREDRAFT_146505 | K18342 |
| TRINITY_DN6444_c1_g1_i1 | 0.0101061 | 0.791995 | XP_002949598.1 | hypothetical protein VOLCADRAFT_32707, partial [Volvox carteri f.nagariensis] | 5.00E-70 | vcn:VOLCADRAFT_32707 | K09140 |
| TRINITY_DN12019_c1_g3_i1 | 0.00759851 | 0.792245 | XP_001697125.1 | mitochondrial ribosomal protein L11 [Chlamydomonas reinhardtii] | 2.00E-44 | cre:CHLREDRAFT_184822 | K02867 |
| TRINITY_DN60123_c0_g1_i1 | 1.21E-06 | 0.792413 | XP_001691632.1 | beta subunit of mitochondrial ATP synthase [Chlamydomonasreinhardtii] | 0 | cre:CHLREDRAFT_78348 | K02133 |
| TRINITY_DN33473_c0_g1_i1 | 4.43E-08 | 0.793632 | XP_002981558.1 | hypothetical protein SELMODRAFT_114698 [Selaginella moellendorffii] | 1.00E-121 | smo:SELMODRAFT_114698 | K11450 |
| TRINITY_DN50614_c0_g1_i1 | 3.27E-13 | 0.793728 | XP_001695594.1 | exportin [Chlamydomonas reinhardtii] | 0 | cre:CHLREDRAFT_138199 | K14290 |
| TRINITY_DN42655_c0_g1_i2 | 8.83E-11 | 0.794216 | XP_002951773.1 | hypothetical protein VOLCADRAFT_44148, partial [Volvox carteri f.nagariensis] | 5.00E-69 | vcn:VOLCADRAFT_44148 | K13354 |
| TRINITY_DN51002_c0_g1_i1 | 2.42E-11 | 0.794455 | XP_002946518.1 | molecular chaperone [Volvox carteri f. nagariensis] | 2.00E-87 | vcn:VOLCADRAFT_72637 | K09528 |
| TRINITY_DN47028_c0_g2_i1 | 0.000340847 | 0.79455 | XP_002946560.1 | hypothetical protein VOLCADRAFT_102982 [Volvox carteri f.nagariensis] | 1.00E-26 | vcn:VOLCADRAFT_102982 | K14557 |
| TRINITY_DN1565_c0_g1_i1 | 2.07E-07 | 0.794568 | XP_001691863.1 | small rab-related GTPase [Chlamydomonas reinhardtii] | 2.00E-99 | cre:CHLREDRAFT_195519 | K07904 |
| TRINITY_DN55810_c1_g1_i1 | 2.25E-05 | 0.796749 | XP_005645006.1 | monodehydroascorbate reductase [Coccomyxa subellipsoidea C-169] | 1.00E-135 | csl:COCSUDRAFT_33977 | K08232 |
| TRINITY_DN46064_c6_g1_i1 | 4.20E-05 | 0.796886 | XP_001702356.1 | trehalose 6-phosphate phosphatase [Chlamydomonas reinhardtii] | 1.00E-102 | cre:CHLREDRAFT_60124 | K01087 |
| TRINITY_DN23274_c0_g1_i1 | 0.00156209 | 0.797483 | XP_005848297.1 | hypothetical protein CHLNCDRAFT_144931 [Chlorella variabilis] | 7.00E-97 | cvr:CHLNCDRAFT_144931 | K04487 |
| TRINITY_DN60249_c0_g1_i1 | 3.15E-07 | 0.797579 | XP_002956910.1 | hypothetical protein VOLCADRAFT_107427 [Volvox carteri f.nagariensis] | 1.00E-108 | vcn:VOLCADRAFT_107427 | K01814 |
| TRINITY_DN19261_c2_g1_i1 | 3.77E-08 | 0.797626 | XP_001777604.1 | predicted protein [Physcomitrella patens] | 1.00E-41 | ppp:PHYPADRAFT_145059 | K03434 |
| TRINITY_DN9462_c0_g1_i1 | 0.00223838 | 0.79811 | XP_002954868.1 | hypothetical protein VOLCADRAFT_106582 [Volvox carteri f.nagariensis] | 0 | vcn:VOLCADRAFT_106582 | K00383 |
| TRINITY_DN29381_c1_g2_i1 | 0.000611711 | 0.798264 | XP_001703262.1 | hypothetical protein CHLREDRAFT_168911 [Chlamydomonas reinhardtii] | 4.00E-54 | cre:CHLREDRAFT_168911 | K17776 |
| TRINITY_DN6512_c2_g1_i1 | 2.64E-05 | 0.79991 | XP_002945626.1 | hypothetical protein VOLCADRAFT_102603 [Volvox carteri f.nagariensis] | 2.00E-20 | vcn:VOLCADRAFT_102603 | K04097 |
| TRINITY_DN50763_c0_g1_i1 | 1.26E-07 | 0.801054 | XP_002953451.1 | hypothetical protein VOLCADRAFT_43315, partial [Volvox carteri f.nagariensis] | 1.00E-167 | vcn:VOLCADRAFT_43315 | K01866 |
| TRINITY_DN8745_c0_g1_i1 | 0.000484016 | 0.801806 | XP_002951627.1 | hypothetical protein VOLCADRAFT_61491 [Volvox carteri f. nagariensis] | 0 | vcn:VOLCADRAFT_61491 | K12736 |
| TRINITY_DN57741_c0_g1_i1 | 0 | 0.803232 | XP_001690775.1 | sterol-C24-methyltransferase [Chlamydomonas reinhardtii] | 1.00E-137 | cre:CHLREDRAFT_161904 | K08242 |
| TRINITY_DN37241_c3_g1_i1 | 0.00735712 | 0.803233 | XP_005847263.1 | hypothetical protein CHLNCDRAFT_9858, partial [Chlorella variabilis] | 6.00E-96 | cvr:CHLNCDRAFT_9858 | K06125 |
| TRINITY_DN32898_c0_g1_i1 | 2.64E-05 | 0.803638 | XP_002953466.1 | malate dehydrogenase [Volvox carteri f. nagariensis] | 1.00E-139 | vcn:VOLCADRAFT_75816 | K00026 |
| TRINITY_DN46187_c0_g4_i1 | 5.30E-05 | 0.8044 | XP_005848586.1 | hypothetical protein CHLNCDRAFT_57693 [Chlorella variabilis] | 1.00E-151 | cvr:CHLNCDRAFT_57693 | K10589 |
| TRINITY_DN53176_c0_g1_i1 | 0.0357052 | 0.805078 | XP_001703505.1 | amino acid permease [Chlamydomonas reinhardtii] | 1.00E-112 | cre:CHLREDRAFT_196429 | K15015 |
| TRINITY_DN52844_c0_g1_i1 | 1.14E-11 | 0.805933 | XP_001692927.1 | glutamine synthetase [Chlamydomonas reinhardtii] | 1.00E-159 | cre:CHLREDRAFT_136895 | K01915 |
| TRINITY_DN23984_c2_g1_i1 | 0.0193218 | 0.807121 | XP_005647417.1 | hypothetical protein COCSUDRAFT_66433 [Coccomyxa subellipsoideaC-169] | 3.00E-46 | csl:COCSUDRAFT_66433 | K12741 |
| TRINITY_DN55440_c0_g1_i1 | 2.11E-15 | 0.807509 | XP_002947006.1 | small nuclear ribonucleoprotein [Volvox carteri f. nagariensis] | 7.00E-43 | vcn:VOLCADRAFT_79410 | K11088 |
| TRINITY_DN433_c1_g1_i1 | 4.02E-09 | 0.807828 | XP_005643738.1 | DUF382-domain-containing protein [Coccomyxa subellipsoidea C-169] | 1.00E-150 | csl:COCSUDRAFT_49041 | K12829 |
| TRINITY_DN21875_c0_g1_i1 | 6.17E-09 | 0.807869 | XP_002946277.1 | hypothetical protein VOLCADRAFT_86387 [Volvox carteri f. nagariensis] | 0 | vcn:VOLCADRAFT_86387 | K18995 |
| TRINITY_DN55573_c0_g1_i1 | 0.000452515 | 0.808995 | XP_006469891.1 | PREDICTED: ADP,ATP carrier protein 3, mitochondrial [Citrus sinensis] | 1.00E-105 | cit:102607117 | K05863 |
| TRINITY_DN52962_c1_g1_i1 | 9.70E-07 | 0.809143 | XP_001699500.1 | transmembrane ATPase [Chlamydomonas reinhardtii] | 2.00E-19 | cre:CHLREDRAFT_59366 | K02152 |
| TRINITY_DN40295_c0_g2_i2 | 0.00130847 | 0.810087 | XP_002954154.1 | hypothetical protein VOLCADRAFT_118613 [Volvox carteri f.nagariensis] | 0 | vcn:VOLCADRAFT_118613 | K14788 |
| TRINITY_DN47187_c4_g1_i2 | 2.00E-06 | 0.810108 | XP_001699221.1 | hypothetical protein CHLREDRAFT_152465 [Chlamydomonas reinhardtii] | 1.00E-166 | cre:CHLREDRAFT_152465 | K15305 |
| TRINITY_DN48287_c0_g1_i1 | 1.04E-05 | 0.810724 | XP_001690813.1 | predicted protein [Chlamydomonas reinhardtii] | 0 | cre:CHLREDRAFT_223 | K14777 |
| TRINITY_DN6213_c0_g1_i1 | 0.00472925 | 0.811081 | XP_002952360.1 | hypothetical protein VOLCADRAFT_81828 [Volvox carteri f. nagariensis] | 7.00E-87 | vcn:VOLCADRAFT_81828 | K15429 |
| TRINITY_DN8832_c0_g2_i1 | 0 | 0.811343 | XP_001703190.1 | hypothetical protein CHLREDRAFT_180347, partial [Chlamydomonasreinhardtii] | 1.00E-151 | cre:CHLREDRAFT_180347 | K15501 |
| TRINITY_DN55525_c0_g1_i1 | 0 | 0.81139 | XP_001696637.1 | glycine cleavage system, H-protein [Chlamydomonas reinhardtii] | 2.00E-47 | cre:CHLREDRAFT_196067 | K02437 |
| TRINITY_DN43272_c8_g3_i1 | 3.20E-06 | 0.811609 | XP_002947558.1 | hypothetical protein VOLCADRAFT_57026 [Volvox carteri f.nagariensis] | 4.00E-76 | vcn:VOLCADRAFT_57026 | K02738 |
| TRINITY_DN57763_c0_g1_i1 | 0.0144244 | 0.81166 | XP_002958600.1 | hypothetical protein VOLCADRAFT_108195 [Volvox carteri f.nagariensis] | 1.00E-99 | vcn:VOLCADRAFT_108195 | K02736 |
| TRINITY_DN53393_c1_g1_i1 | 0.000102275 | 0.811798 | XP_001690529.1 | hypothetical protein CHLREDRAFT_55169 [Chlamydomonas reinhardtii] | 6.00E-83 | cre:CHLREDRAFT_55169 | K09602 |
| TRINITY_DN481_c0_g1_i1 | 0.012209 | 0.811799 | XP_001701596.1 | mitochondrial ribosomal protein L7/L12 [Chlamydomonas reinhardtii] | 1.00E-26 | cre:CHLREDRAFT_194556 | K02935 |
| TRINITY_DN57627_c2_g1_i1 | 0.000495106 | 0.811877 | XP_002947136.1 | dihydrodipicolinate synthase [Volvox carteri f. nagariensis] | 1.00E-123 | vcn:VOLCADRAFT_56764 | K01714 |
| TRINITY_DN55997_c2_g1_i1 | 0.0285607 | 0.812846 | XP_001697169.1 | mitochondrial ribosomal protein L23 [Chlamydomonas reinhardtii] | 7.00E-27 | cre:CHLREDRAFT_150589 | K02892 |
| TRINITY_DN3580_c0_g2_i1 | 4.14E-09 | 0.813452 | XP_001696623.1 | serine/threonine phosphatase, family 2C [Chlamydomonas reinhardtii] | 2.00E-83 | cre:CHLREDRAFT_142396 | K17508 |
| TRINITY_DN45110_c2_g3_i2 | 0.00621506 | 0.813686 | XP_005649191.1 | zf-DHHC-domain-containing protein [Coccomyxa subellipsoidea C-169] | 7.00E-32 | csl:COCSUDRAFT_53025 | K18932 |
| TRINITY_DN50617_c0_g1_i1 | 0.0110348 | 0.81499 | XP_001696429.1 | vacuolar H+ ATPase V1 sector, subunit C [Chlamydomonas reinhardtii] | 1.00E-118 | cre:CHLREDRAFT_187322 | K02148 |
| TRINITY_DN4651_c0_g2_i1 | 2.41E-12 | 0.81505 | XP_002945797.1 | hypothetical protein VOLCADRAFT_78796 [Volvox carteri f.nagariensis] | 1.00E-22 | vcn:VOLCADRAFT_78796 | K00417 |
| TRINITY_DN7940_c0_g1_i1 | 0.00358012 | 0.815377 | XP_001700042.1 | 26S proteasome regulatory subunit [Chlamydomonas reinhardtii] | 1.00E-119 | cre:CHLREDRAFT_23975 | K03036 |
| TRINITY_DN55240_c1_g1_i1 | 0 | 0.815377 | XP_001691193.1 | gamma-cop [Chlamydomonas reinhardtii] | 0 | cre:CHLREDRAFT_78205 | K17267 |
| TRINITY_DN55240_c1_g2_i1 | 0 | 0.815396 | XP_001691193.1 | gamma-cop [Chlamydomonas reinhardtii] | 0 | cre:CHLREDRAFT_78205 | K17267 |
| TRINITY_DN33746_c2_g1_i1 | 0 | 0.815635 | XP_011626632.1 | PREDICTED: polyubiquitin [Amborella trichopoda] | 5.00E-38 | atr:105421327 | K08770 |
| TRINITY_DN57784_c1_g1_i1 | 0.000633305 | 0.815874 | XP_001691775.1 | predicted protein [Chlamydomonas reinhardtii] | 2.00E-22 | cre:CHLREDRAFT_170481 | K07253 |
| TRINITY_DN44777_c1_g3_i1 | 0.0083298 | 0.81597 | XP_002949269.1 | hypothetical protein VOLCADRAFT_89548 [Volvox carteri f. nagariensis] | 1.00E-45 | vcn:VOLCADRAFT_89548 | K18667 |
| TRINITY_DN55443_c1_g1_i1 | 0 | 0.816278 | XP_001700829.1 | phosphoribosylformylglycinamidine cyclo-ligase [Chlamydomonasreinhardtii] | 1.00E-133 | cre:CHLREDRAFT_54374 | K01933 |
| TRINITY_DN17210_c2_g2_i1 | 6.08E-05 | 0.816374 | XP_002955914.1 | hypothetical protein VOLCADRAFT_106985 [Volvox carteri f.nagariensis] | 4.00E-87 | vcn:VOLCADRAFT_106985 | K15285 |
| TRINITY_DN53193_c1_g1_i1 | 0.00616435 | 0.816596 | XP_002956472.1 | hypothetical protein VOLCADRAFT_110015 [Volvox carteri f.nagariensis] | 2.00E-77 | vcn:VOLCADRAFT_110015 | K02734 |
| TRINITY_DN47850_c0_g1_i2 | 0.000183193 | 0.817316 | XP_001698684.1 | hypothetical protein CHLREDRAFT_151721 [Chlamydomonas reinhardtii] | 1.00E-132 | cre:CHLREDRAFT_151721 | K05275 |
| TRINITY_DN48428_c0_g1_i1 | 0.000752611 | 0.817599 | XP_002948131.1 | hypothetical protein VOLCADRAFT_103761 [Volvox carteri f.nagariensis] | 5.00E-75 | vcn:VOLCADRAFT_103761 | K17496 |
| TRINITY_DN53482_c0_g1_i1 | 6.34E-06 | 0.81794 | XP_011398157.1 | Elongation factor 2 [Auxenochlorella protothecoides] | 2.00E-25 | apro:F751_3945 | K03234 |
| TRINITY_DN33746_c2_g16_i1 | 3.73E-05 | 0.818983 | XP_011626632.1 | PREDICTED: polyubiquitin [Amborella trichopoda] | 7.00E-36 | atr:105421327 | K08770 |
| TRINITY_DN2117_c0_g2_i1 | 0.000485 | 0.820319 | XP_002949634.1 | mitochondrial ribosomal protein S7, partial [Volvox carteri f.nagariensis] | 6.00E-14 | vcn:VOLCADRAFT_59573 | K02992 |
| TRINITY_DN43783_c2_g1_i1 | 0.000494126 | 0.821036 | XP_005650609.1 | Actin/actin-like protein [Coccomyxa subellipsoidea C-169] | 9.00E-61 | csl:COCSUDRAFT_46476 | K11662 |
| TRINITY_DN57861_c0_g1_i1 | 0.0141567 | 0.82161 | XP_005648666.1 | hypothetical protein COCSUDRAFT_65754 [Coccomyxa subellipsoideaC-169] | 0 | csl:COCSUDRAFT_65754 | K12860 |
| TRINITY_DN48181_c1_g1_i1 | 0.000919788 | 0.822508 | XP_001695456.1 | coiled-coil domain 6-like protein [Chlamydomonas reinhardtii] | 9.00E-53 | cre:CHLREDRAFT_191672 | K09288 |
| TRINITY_DN17367_c0_g1_i1 | 0.0436739 | 0.825394 | XP_011396222.1 | Signal recognition particle 9 kDa protein [Auxenochlorellaprotothecoides] | 1.00E-19 | apro:F751_1048 | K03109 |
| TRINITY_DN60410_c0_g1_i1 | 1.25E-09 | 0.825543 | XP_002958781.1 | chaperonin complex component [Volvox carteri f. nagariensis] | 0 | vcn:VOLCADRAFT_108310 | K09499 |
| TRINITY_DN33746_c2_g9_i1 | 2.46E-06 | 0.826327 | XP_011626632.1 | PREDICTED: polyubiquitin [Amborella trichopoda] | 1.00E-51 | atr:105421327 | K08770 |
| TRINITY_DN48196_c0_g1_i1 | 0 | 0.827602 | XP_001702008.1 | protein of endonuclease / exonuclease / phosphatase family[Chlamydomonas reinhardtii] | 0 | cre:CHLREDRAFT_155138 | K12603 |
| TRINITY_DN57678_c0_g1_i1 | 1.09E-05 | 0.828269 | XP_001693168.1 | farnesyl diphosphate synthase [Chlamydomonas reinhardtii] | 1.00E-147 | cre:CHLREDRAFT_137019 | K00787 |
| TRINITY_DN48180_c0_g1_i1 | 1.67E-06 | 0.828998 | XP_001696864.1 | L-aspartate oxidase [Chlamydomonas reinhardtii] | 0 | cre:CHLREDRAFT_58579 | K00278 |
| TRINITY_DN50566_c0_g1_i1 | 5.56E-05 | 0.829361 | XP_002950454.1 | hypothetical protein VOLCADRAFT_109764 [Volvox carteri f.nagariensis] | 1.00E-78 | vcn:VOLCADRAFT_109764 | K15030 |
| TRINITY_DN48233_c1_g1_i1 | 0.0011529 | 0.830289 | XP_001696254.1 | hypothetical protein CHLREDRAFT_187099 [Chlamydomonas reinhardtii] | 1.00E-96 | cre:CHLREDRAFT_187099 | K02735 |
| TRINITY_DN33746_c2_g2_i1 | 5.55E-12 | 0.830438 | XP_011626632.1 | PREDICTED: polyubiquitin [Amborella trichopoda] | 1.00E-31 | atr:105421327 | K08770 |
| TRINITY_DN58079_c1_g1_i1 | 0.000140305 | 0.830912 | XP_001699273.1 | OST3/OST6 family protein [Chlamydomonas reinhardtii] | 1.00E-58 | cre:CHLREDRAFT_196082 | K12669 |
| TRINITY_DN48142_c3_g1_i1 | 0.000207154 | 0.83177 | XP_002950288.1 | hypothetical protein VOLCADRAFT_81030 [Volvox carteri f. nagariensis] | 1.00E-134 | vcn:VOLCADRAFT_81030 | K00856 |
| TRINITY_DN18185_c0_g1_i1 | 0.00446315 | 0.832384 | XP_002953782.1 | hypothetical protein VOLCADRAFT_64030 [Volvox carteri f. nagariensis] | 1.00E-176 | vcn:VOLCADRAFT_64030 | K00620 |
| TRINITY_DN55373_c0_g1_i1 | 0 | 0.832771 | XP_002947964.1 | hypothetical protein VOLCADRAFT_103748 [Volvox carteri f.nagariensis] | 1.00E-147 | vcn:VOLCADRAFT_103748 | K12666 |
| TRINITY_DN44085_c0_g2_i2 | 0.0340499 | 0.83288 | XP_005644612.1 | hypothetical protein COCSUDRAFT_48589 [Coccomyxa subellipsoideaC-169] | 2.00E-68 | csl:COCSUDRAFT_48589 | K03348 |
| TRINITY_DN60028_c1_g1_i1 | 0.00115099 | 0.833553 | XP_001691250.1 | 20S proteasome alpha subunit F [Chlamydomonas reinhardtii] | 1.00E-105 | cre:CHLREDRAFT_115213 | K02725 |
| TRINITY_DN55351_c0_g1_i1 | 3.94E-08 | 0.833887 | XP_001690795.1 | glycerol-3-phosphate permease-like protein [Chlamydomonasreinhardtii] | 0 | cre:CHLREDRAFT_144519 | K13783 |
| TRINITY_DN16613_c0_g1_i1 | 0 | 0.834417 | XP_002951779.1 | hypothetical protein VOLCADRAFT_120990 [Volvox carteri f.nagariensis] | 0 | vcn:VOLCADRAFT_120990 | K03798 |
| TRINITY_DN31824_c0_g1_i2 | 0.0103403 | 0.834454 | XP_001698900.1 | hypothetical protein CHLREDRAFT_159323, partial [Chlamydomonasreinhardtii] | 1.00E-26 | cre:CHLREDRAFT_159323 | K03145 |
| TRINITY_DN795_c1_g2_i1 | 1.55E-05 | 0.834726 | XP_001703219.1 | cullin [Chlamydomonas reinhardtii] | 0 | cre:CHLREDRAFT_133199 | K10609 |
| TRINITY_DN40123_c2_g1_i3 | 0.0471439 | 0.835608 | XP_011400305.1 | Alpha-L-arabinofuranosidase 1 [Auxenochlorella protothecoides] | 6.00E-77 | apro:F751_4608 | K01209 |
| TRINITY_DN48248_c0_g1_i1 | 3.06E-05 | 0.836187 | XP_001691181.1 | small rab-related GTPase [Chlamydomonas reinhardtii] | 2.00E-92 | cre:CHLREDRAFT_195521 | K07897 |
| TRINITY_DN41556_c6_g1_i1 | 0 | 0.836793 | XP_002953271.1 | NADH:ubiquinone oxidoreductase 30 kDa subunit [Volvox carteri f.nagariensis] | 2.00E-85 | vcn:VOLCADRAFT_75676 | K03936 |
| TRINITY_DN6067_c0_g1_i1 | 0.0171692 | 0.8377 | XP_002955960.1 | hypothetical protein VOLCADRAFT_66444 [Volvox carteri f. nagariensis] | 0 | vcn:VOLCADRAFT_66444 | K01883 |
| TRINITY_DN24271_c0_g1_i2 | 0.00406351 | 0.837767 | XP_002446053.1 | hypothetical protein SORBIDRAFT_06g001050 [Sorghum bicolor] | 1.00E-139 | sbi:SORBI_06g001050 | K05544 |
| TRINITY_DN36584_c0_g2_i2 | 0.000154884 | 0.837816 | XP_001703337.1 | predicted protein, partial [Chlamydomonas reinhardtii] | 2.00E-89 | cre:CHLREDRAFT_99525 | K10395 |
| TRINITY_DN21789_c3_g1_i1 | 6.51E-06 | 0.838049 | XP_001693453.1 | NADH:ubiquinone oxidoreductase 18 kDa subunit [Chlamydomonasreinhardtii] | 5.00E-37 | cre:CHLREDRAFT_127639 | K03949 |
| TRINITY_DN58135_c1_g1_i1 | 0.00112365 | 0.838194 | XP_002955555.1 | 26S proteasome regulatory complex [Volvox carteri f. nagariensis] | 1.00E-116 | vcn:VOLCADRAFT_109976 | K03038 |
| TRINITY_DN12602_c0_g3_i1 | 1.90E-06 | 0.839634 | XP_001694607.1 | phytol kinase-related protein [Chlamydomonas reinhardtii] | 9.00E-68 | cre:CHLREDRAFT_173650 | K18678 |
| TRINITY_DN8539_c0_g1_i1 | 1.14E-13 | 0.840709 | XP_001696039.1 | AIR synthase-related protein [Chlamydomonas reinhardtii] | 0 | cre:CHLREDRAFT_131059 | K01952 |
| TRINITY_DN50683_c0_g1_i1 | 0.0152692 | 0.840957 | XP_001702817.1 | eukaryotic initiation factor [Chlamydomonas reinhardtii] | 1.00E-124 | cre:CHLREDRAFT_108477 | K03680 |
| TRINITY_DN2468_c0_g1_i1 | 3.01E-05 | 0.84152 | XP_001691870.1 | eukaryotic initiation factor [Chlamydomonas reinhardtii] | 0 | cre:CHLREDRAFT_128636 | K03253 |
| TRINITY_DN2668_c1_g1_i1 | 4.45E-07 | 0.843362 | XP_002948217.1 | hypothetical protein VOLCADRAFT_80147 [Volvox carteri f. nagariensis] | 1.00E-102 | vcn:VOLCADRAFT_80147 | K15356 |
| TRINITY_DN19402_c1_g1_i1 | 4.96E-05 | 0.845684 | XP_002950745.1 | hypothetical protein VOLCADRAFT_109095 [Volvox carteri f.nagariensis] | 7.00E-59 | vcn:VOLCADRAFT_109095 | K12403 |
| TRINITY_DN48264_c1_g1_i1 | 0.0455909 | 0.846188 | XP_001692523.1 | subunit f DNA-directed RNA polymerase [Chlamydomonas reinhardtii] | 4.00E-14 | cre:CHLREDRAFT_78868 | K03009 |
| TRINITY_DN33746_c2_g7_i1 | 0 | 0.846369 | XP_011626632.1 | PREDICTED: polyubiquitin [Amborella trichopoda] | 8.00E-56 | atr:105421327 | K08770 |
| TRINITY_DN60090_c1_g1_i1 | 0.0249974 | 0.84683 | XP_002957505.1 | DNA binding helix-turn helix protein [Volvox carteri f.nagariensis] | 5.00E-38 | vcn:VOLCADRAFT_110064 | K03627 |
| TRINITY_DN48844_c1_g1_i1 | 0.00938058 | 0.848746 | XP_002945886.1 | hypothetical protein VOLCADRAFT_72524 [Volvox carteri f. nagariensis] | 2.00E-52 | vcn:VOLCADRAFT_72524 | K02878 |
| TRINITY_DN60268_c0_g1_i1 | 0.0305455 | 0.84877 | XP_002951280.1 | hypothetical protein VOLCADRAFT_105037 [Volvox carteri f.nagariensis] | 1.00E-114 | vcn:VOLCADRAFT_105037 | K15356 |
| TRINITY_DN266_c0_g1_i1 | 7.68E-05 | 0.849264 | XP_001696916.1 | SM/Sec1-family protein [Chlamydomonas reinhardtii] | 0 | cre:CHLREDRAFT_131436 | K19998 |
| TRINITY_DN60325_c1_g1_i1 | 3.96E-05 | 0.85019 | XP_002952668.1 | hypothetical protein VOLCADRAFT_62890 [Volvox carteri f. nagariensis] | 1.00E-104 | vcn:VOLCADRAFT_62890 | K04083 |
| TRINITY_DN51033_c3_g1_i1 | 0.0158119 | 0.851166 | XP_002950669.1 | hypothetical protein VOLCADRAFT_101939 [Volvox carteri f.nagariensis] | 5.00E-65 | vcn:VOLCADRAFT_101939 | K12734 |
| TRINITY_DN18294_c0_g1_i1 | 0.00389117 | 0.853826 | XP_002947954.1 | eukaryotic translation initiation factor 4 [Volvox carteri f.nagariensis] | 1.00E-148 | vcn:VOLCADRAFT_103732 | K03260 |
| TRINITY_DN18265_c0_g6_i1 | 0 | 0.855345 | XP_001690213.1 | small rab-related GTPase [Chlamydomonas reinhardtii] | 4.00E-95 | cre:CHLREDRAFT_195522 | K07893 |
| TRINITY_DN33746_c2_g14_i2 | 0 | 0.855645 | XP_011626632.1 | PREDICTED: polyubiquitin [Amborella trichopoda] | 8.00E-48 | atr:105421327 | K08770 |
| TRINITY_DN60187_c1_g1_i1 | 0.0230824 | 0.856044 | XP_001697380.1 | plastid ribosomal protein L4 [Chlamydomonas reinhardtii] | 8.00E-74 | cre:CHLREDRAFT_185040 | K02926 |
| TRINITY_DN22247_c0_g2_i1 | 0.0113691 | 0.857287 | XP_002956722.1 | hypothetical protein VOLCADRAFT_67317 [Volvox carteri f. nagariensis] | 1.00E-135 | vcn:VOLCADRAFT_67317 | K06228 |
| TRINITY_DN57620_c1_g1_i1 | 1.53E-07 | 0.857566 | XP_002946855.1 | vacuolar H+ ATPase V0 sector, subunit D [Volvox carteri f.nagariensis] | 1.00E-178 | vcn:VOLCADRAFT_72840 | K02146 |
| TRINITY_DN55227_c0_g2_i1 | 0.00666774 | 0.858825 | XP_001689587.1 | 20S proteasome alpha subunit D [Chlamydomonas reinhardtii] | 1.00E-105 | cre:CHLREDRAFT_183620 | K02731 |
| TRINITY_DN50671_c1_g1_i1 | 0.0391215 | 0.860225 | XP_002945811.1 | hypothetical protein VOLCADRAFT_102779 [Volvox carteri f.nagariensis] | 1.00E-130 | vcn:VOLCADRAFT_102779 | K05359 |
| TRINITY_DN50437_c1_g1_i1 | 0.00152685 | 0.860425 | XP_005845039.1 | hypothetical protein CHLNCDRAFT_26131 [Chlorella variabilis] | 1.00E-124 | cvr:CHLNCDRAFT_26131 | K05928 |
| TRINITY_DN4746_c0_g2_i1 | 0.00475651 | 0.860774 | XP_001696258.1 | subunit of the signal recognition particle [Chlamydomonasreinhardtii] | 1.00E-102 | cre:CHLREDRAFT_7632 | K03107 |
| TRINITY_DN6178_c0_g1_i1 | 0.0407658 | 0.861751 | XP_001692687.1 | peptidase subunit of mitochondrial ATP-dependent protease hslUV[Chlamydomonas reinhardtii] | 4.00E-68 | cre:CHLREDRAFT_56993 | K01419 |
| TRINITY_DN60361_c0_g1_i1 | 0.00484404 | 0.863267 | XP_001692161.1 | intraflagellar transport protein IFT52 [Chlamydomonas reinhardtii] | 1.00E-144 | cre:CHLREDRAFT_24116 | K19681 |
| TRINITY_DN22950_c2_g2_i1 | 4.65E-05 | 0.863774 | XP_001696613.1 | 14-3-3 protein [Chlamydomonas reinhardtii] | 5.00E-99 | cre:CHLREDRAFT_187228 | K06630 |
| TRINITY_DN8860_c1_g1_i1 | 6.13E-08 | 0.864489 | XP_001778619.1 | predicted protein [Physcomitrella patens] | 3.00E-84 | ppp:PHYPADRAFT_170356 | K12572 |
| TRINITY_DN47796_c10_g1_i1 | 8.12E-05 | 0.866591 | XP_002953621.1 | hypothetical protein VOLCADRAFT_82410 [Volvox carteri f. nagariensis] | 0 | vcn:VOLCADRAFT_82410 | K00121 |
| TRINITY_DN57770_c0_g1_i1 | 3.45E-05 | 0.866793 | XP_002947297.1 | 4-hydroxy-3-methylbut-2-enyl diphosphate reductase chloroplastprecursor [Volvox carteri f. nagariensis] | 1.00E-174 | vcn:VOLCADRAFT_79586 | K03527 |
| TRINITY_DN5128_c0_g1_i1 | 0.000756269 | 0.869429 | XP_002948379.1 | hypothetical protein VOLCADRAFT_88659, partial [Volvox carteri f.nagariensis] | 1.00E-154 | vcn:VOLCADRAFT_88659 | K14289 |
| TRINITY_DN42259_c0_g2_i8 | 0.00356612 | 0.870229 | XP_002952192.1 | Ca2+/H+ antiporter, cation antiporter, membrane protein [Volvoxcarteri f. nagariensis] | 8.00E-31 | vcn:VOLCADRAFT_62308 | K07300 |
| TRINITY_DN8538_c0_g2_i1 | 0 | 0.872548 | XP_001702575.1 | 40 kDa translocon at mitochondrial outer envelope membrane[Chlamydomonas reinhardtii] | 1.00E-124 | cre:CHLREDRAFT_195513 | K11518 |
| TRINITY_DN60118_c0_g1_i1 | 0 | 0.872923 | XP_001695070.1 | small rab-related GTPase [Chlamydomonas reinhardtii] | 1.00E-103 | cre:CHLREDRAFT_148836 | K07877 |
| TRINITY_DN50461_c0_g1_i1 | 8.67E-06 | 0.874361 | XP_005851025.1 | hypothetical protein CHLNCDRAFT_56983 [Chlorella variabilis] | 1.00E-112 | cvr:CHLNCDRAFT_56983 | K02737 |
| TRINITY_DN60578_c1_g1_i1 | 0.0225156 | 0.87505 | XP_002954651.1 | programmed cell death protein 2, C terminal [Volvox carteri f.nagariensis] | 5.00E-78 | vcn:VOLCADRAFT_118784 | K14801 |
| TRINITY_DN55813_c0_g1_i1 | 0.00242781 | 0.876672 | XP_002949303.1 | hypothetical protein VOLCADRAFT_89620 [Volvox carteri f.nagariensis] | 4.00E-74 | vcn:VOLCADRAFT_89620 | K14769 |
| TRINITY_DN22950_c2_g1_i2 | 0.0033226 | 0.876758 | XP_001702812.1 | 14-3-3 protein [Chlamydomonas reinhardtii] | 1.00E-122 | cre:CHLREDRAFT_185967 | K06630 |
| TRINITY_DN437_c0_g1_i1 | 1.78E-05 | 0.876894 | XP_002952903.1 | hypothetical protein VOLCADRAFT_93610 [Volvox carteri f. nagariensis] | 1.00E-140 | vcn:VOLCADRAFT_93610 | K11826 |
| TRINITY_DN6876_c0_g2_i1 | 0.000291754 | 0.876976 | XP_002951751.1 | hypothetical protein VOLCADRAFT_105177 [Volvox carteri f.nagariensis] | 2.00E-26 | vcn:VOLCADRAFT_105177 | K03596 |
| TRINITY_DN24171_c0_g1_i1 | 0.0114662 | 0.878624 | XP_005844804.1 | hypothetical protein CHLNCDRAFT_138705 [Chlorella variabilis] | 1.00E-177 | cvr:CHLNCDRAFT_138705 | K11886 |
| TRINITY_DN60436_c2_g1_i1 | 0.0216633 | 0.878687 | XP_001698797.1 | NADH:ubiquinone oxidoreductase 14 kDa subunit [Chlamydomonasreinhardtii] | 1.00E-35 | cre:CHLREDRAFT_164424 | K03965 |
| TRINITY_DN29411_c0_g2_i1 | 3.55E-08 | 0.879915 | XP_001697830.1 | ATPase, phospholipid transporter [Chlamydomonas reinhardtii] | 0 | cre:CHLREDRAFT_193025 | K01530 |
| TRINITY_DN8735_c0_g1_i2 | 0.015024 | 0.885034 | XP_002955185.1 | hypothetical protein VOLCADRAFT_83102 [Volvox carteri f.nagariensis] | 4.00E-80 | vcn:VOLCADRAFT_83102 | K20028 |
| TRINITY_DN8748_c0_g1_i1 | 1.70E-05 | 0.888166 | XP_001691372.1 | beta-amylase [Chlamydomonas reinhardtii] | 0 | cre:CHLREDRAFT_183141 | K01177 |
| TRINITY_DN7015_c0_g1_i1 | 0.0359744 | 0.890581 | XP_002952493.1 | 26S proteasome regulatory complex [Volvox carteri f. nagariensis] | 1.00E-124 | vcn:VOLCADRAFT_109166 | K03039 |
| TRINITY_DN11130_c0_g1_i1 | 0.000410798 | 0.892157 | XP_002956918.1 | hypothetical protein VOLCADRAFT_77298 [Volvox carteri f. nagariensis] | 1.00E-160 | vcn:VOLCADRAFT_77298 | K17086 |
| TRINITY_DN7748_c0_g2_i1 | 0.0106519 | 0.892481 | XP_001696815.1 | subunit of retromer complex [Chlamydomonas reinhardtii] | 1.00E-122 | cre:CHLREDRAFT_158645 | K18466 |
| TRINITY_DN53277_c0_g1_i1 | 0.0123134 | 0.892582 | XP_002953705.1 | hypothetical protein VOLCADRAFT_106052 [Volvox carteri f.nagariensis] | 9.00E-22 | vcn:VOLCADRAFT_106052 | K06674 |
| TRINITY_DN46976_c0_g1_i1 | 0.000112625 | 0.892582 | XP_001690544.1 | EMP/nonaspanin domain family protein [Chlamydomonas reinhardtii] | 0 | cre:CHLREDRAFT_127873 | K17086 |
| TRINITY_DN46976_c0_g2_i1 | 0.000112625 | 0.895108 | XP_001690544.1 | EMP/nonaspanin domain family protein [Chlamydomonas reinhardtii] | 0 | cre:CHLREDRAFT_127873 | K17086 |
| TRINITY_DN22869_c1_g1_i1 | 0.0333849 | 0.895568 | XP_001696415.1 | NADP-dependent malic enzyme [Chlamydomonas reinhardtii] | 0 | cre:CHLREDRAFT_126820 | K00029 |
| TRINITY_DN57845_c0_g1_i1 | 0.0235209 | 1.14189 | XP_001692030.1 | seryl-trna synthetase [Chlamydomonas reinhardtii] | 0 | cre:CHLREDRAFT_206186 | K01875 |
| TRINITY_DN46578_c0_g2_i1 | 0.000145251 | 1.14918 | XP_002955621.1 | hypothetical protein VOLCADRAFT_66123 [Volvox carteri f. nagariensis] | 9.00E-87 | vcn:VOLCADRAFT_66123 | K15889 |
| TRINITY_DN57962_c0_g1_i1 | 1.93E-09 | 1.14918 | XP_001701673.1 | dynamin-related GTPase [Chlamydomonas reinhardtii] | 0 | cre:CHLREDRAFT_195428 | K01528 |
| TRINITY_DN57962_c0_g2_i1 | 1.93E-09 | 1.17816 | XP_001701673.1 | dynamin-related GTPase [Chlamydomonas reinhardtii] | 0 | cre:CHLREDRAFT_195428 | K01528 |
| TRINITY_DN12860_c0_g2_i1 | 0.00953128 | 1.19289 | XP_002950380.1 | hypothetical protein VOLCADRAFT_104643 [Volvox carteri f.nagariensis] | 1.00E-68 | vcn:VOLCADRAFT_104643 | K14823 |
| TRINITY_DN17214_c0_g1_i2 | 0.000748061 | 1.19435 | XP_010048411.1 | PREDICTED: serine/threonine-protein kinase/endoribonucleaseIRE1a-like [Eucalyptus grandis] | 1.00E-12 | egr:104437206 | K08852 |
| TRINITY_DN44644_c3_g1_i1 | 0.0200676 | 1.20127 | XP_002955156.1 | nitrate reductase [Volvox carteri f. nagariensis] | 0 | vcn:VOLCADRAFT_76569 | K10534 |
| TRINITY_DN48395_c1_g1_i1 | 0.0228548 | 1.20508 | XP_001699718.1 | plastid ribosomal protein L17 [Chlamydomonas reinhardtii] | 1.00E-45 | cre:CHLREDRAFT_195620 | K02879 |
| TRINITY_DN55411_c0_g2_i1 | 2.79E-08 | 1.21856 | XP_002947880.1 | hypothetical protein VOLCADRAFT_120525 [Volvox carteri f.nagariensis] | 1.00E-134 | vcn:VOLCADRAFT_120525 | K02357 |
| TRINITY_DN52789_c1_g2_i1 | 5.33E-15 | 1.21975 | XP_002947450.1 | hypothetical protein VOLCADRAFT_79605 [Volvox carteri f. nagariensis] | 0 | vcn:VOLCADRAFT_79605 | K03301 |
| TRINITY_DN48270_c0_g1_i1 | 0.014798 | 1.24206 | XP_002948124.1 | hypothetical protein VOLCADRAFT_73602 [Volvox carteri f. nagariensis] | 1.00E-104 | vcn:VOLCADRAFT_73602 | K00365 |
| TRINITY_DN29757_c0_g1_i1 | 1.92E-06 | 1.24428 | XP_001697511.1 | chromodomain-helicase-DNA-binding protein, partial [Chlamydomonasreinhardtii] | 1.00E-117 | cre:CHLREDRAFT_150334 | K14437 |
| TRINITY_DN32850_c1_g2_i1 | 0 | 1.2502 | XP_002953231.1 | hypothetical protein VOLCADRAFT_93958 [Volvox carteri f. nagariensis] | 0 | vcn:VOLCADRAFT_93958 | K06185 |
| TRINITY_DN25057_c0_g1_i1 | 8.68E-08 | 1.25384 | XP_011395697.1 | ABC transporter E family member 2 [Auxenochlorella protothecoides] | 0 | apro:F751_5540 | K06174 |
| TRINITY_DN22493_c2_g2_i1 | 0.00164532 | 1.26053 | XP_001697667.1 | prefoldin-related KE2-like protein [Chlamydomonas reinhardtii] | 9.00E-12 | cre:CHLREDRAFT_151087 | K09548 |
| TRINITY_DN47819_c0_g3_i2 | 1.11E-16 | 1.26285 | XP_002954454.1 | hypothetical protein VOLCADRAFT_95258 [Volvox carteri f. nagariensis] | 3.00E-46 | vcn:VOLCADRAFT_95258 | K14304 |
| TRINITY_DN60184_c0_g1_i1 | 5.57E-09 | 1.27107 | XP_001696402.1 | isopropylmalate dehydratase, small subunit [Chlamydomonasreinhardtii] | 5.00E-84 | cre:CHLREDRAFT_126865 | K01704 |
| TRINITY_DN18315_c0_g2_i3 | 0.0135343 | 1.27194 | XP_001696152.1 | hypothetical protein CHLREDRAFT_119554, partial [Chlamydomonasreinhardtii] | 0 | cre:CHLREDRAFT_119554 | K01872 |
| TRINITY_DN55464_c0_g1_i1 | 0.0354526 | 1.27273 | XP_002952366.1 | hypothetical protein VOLCADRAFT_105494 [Volvox carteri f.nagariensis] | 1.00E-139 | vcn:VOLCADRAFT_105494 | K18534 |
| TRINITY_DN6175_c2_g1_i1 | 6.38E-05 | 1.27983 | XP_001700103.1 | GTP binding protein TypA [Chlamydomonas reinhardtii] | 0 | cre:CHLREDRAFT_139610 | K06207 |
| TRINITY_DN2497_c0_g1_i1 | 0 | 1.28266 | XP_001691597.1 | plastid acyl-ACP desaturase [Chlamydomonas reinhardtii] | 1.00E-159 | cre:CHLREDRAFT_205753 | K03921 |
| TRINITY_DN40622_c0_g1_i1 | 0.00349399 | 1.28897 | XP_002957141.1 | hypothetical protein VOLCADRAFT_67840 [Volvox carteri f.nagariensis] | 1.00E-25 | vcn:VOLCADRAFT_67840 | K09561 |
| TRINITY_DN41375_c0_g1_i1 | 0.000286096 | 1.29989 | XP_002950180.1 | hypothetical protein VOLCADRAFT_60049, partial [Volvox carteri f.nagariensis] | 1.00E-99 | vcn:VOLCADRAFT_60049 | K14859 |
| TRINITY_DN31025_c0_g1_i1 | 1.31E-07 | 1.30249 | XP_002950949.1 | hypothetical protein VOLCADRAFT_91452 [Volvox carteri f. nagariensis] | 6.00E-96 | vcn:VOLCADRAFT_91452 | K17583 |
| TRINITY_DN15684_c0_g1_i1 | 8.68E-05 | 1.30437 | XP_001696830.1 | DnaJ-like protein [Chlamydomonas reinhardtii] | 5.00E-60 | cre:CHLREDRAFT_175485 | K09518 |
| TRINITY_DN55355_c0_g2_i1 | 6.44E-15 | 1.31474 | XP_001699403.1 | ser/thr kinase [Chlamydomonas reinhardtii] | 1.00E-114 | cre:CHLREDRAFT_132038 | K14498 |
| TRINITY_DN30091_c0_g1_i2 | 0.0442738 | 1.32409 | XP_002947037.1 | component of cytosolic 80S ribosome and 40S small subunit [Volvoxcarteri f. nagariensis] | 4.00E-79 | vcn:VOLCADRAFT_73024 | K02998 |
| TRINITY_DN43286_c1_g1_i4 | 1.20E-05 | 1.33822 | XP_002947804.1 | hypothetical protein VOLCADRAFT_41600, partial [Volvox carteri f.nagariensis] | 1.00E-151 | vcn:VOLCADRAFT_41600 | K01285 |
| TRINITY_DN12939_c0_g1_i3 | 0.0209765 | 1.35009 | XP_002958598.1 | hypothetical protein VOLCADRAFT_99865 [Volvox carteri f. nagariensis] | 0 | vcn:VOLCADRAFT_99865 | K00703 |
| TRINITY_DN504_c0_g1_i1 | 0.00604903 | 1.35069 | XP_005843025.1 | hypothetical protein CHLNCDRAFT_59390 [Chlorella variabilis] | 5.00E-91 | cvr:CHLNCDRAFT_59390 | K06569 |
| TRINITY_DN50527_c0_g1_i1 | 0.00361813 | 1.35365 | XP_001697664.1 | tRNA pseudouridine synthase [Chlamydomonas reinhardtii] | 3.00E-82 | cre:CHLREDRAFT_151084 | K06173 |
| TRINITY_DN41128_c0_g1_i1 | 3.38E-13 | 1.35497 | XP_002952713.1 | hypothetical protein VOLCADRAFT_93375 [Volvox carteri f. nagariensis] | 1.00E-180 | vcn:VOLCADRAFT_93375 | K18010 |
| TRINITY_DN45901_c1_g1_i1 | 1.92E-05 | 1.3612 | XP_002958679.1 | hypothetical protein VOLCADRAFT_99968 [Volvox carteri f. nagariensis] | 1.00E-55 | vcn:VOLCADRAFT_99968 | K15287 |
| TRINITY_DN47680_c2_g1_i3 | 2.03E-07 | 1.36214 | XP_001697738.1 | methionine aminopeptidase [Chlamydomonas reinhardtii] | 1.00E-101 | cre:CHLREDRAFT_131477 | K01265 |
| TRINITY_DN18832_c0_g1_i1 | 3.81E-07 | 1.36408 | XP_002955341.1 | programmed cell death protein 6 interacting protein X [Volvox carterif. nagariensis] | 0 | vcn:VOLCADRAFT_96198 | K12200 |
| TRINITY_DN44646_c5_g2_i4 | 0.0262825 | 1.3676 | XP_001697738.1 | methionine aminopeptidase [Chlamydomonas reinhardtii] | 4.00E-99 | cre:CHLREDRAFT_131477 | K01265 |
| TRINITY_DN41964_c4_g1_i2 | 0.000558829 | 1.37672 | XP_002947264.1 | hypothetical protein VOLCADRAFT_87448 [Volvox carteri f.nagariensis] | 1.00E-37 | vcn:VOLCADRAFT_87448 | K03859 |
| TRINITY_DN46301_c0_g3_i3 | 0.0269794 | 1.38422 | XP_001689707.1 | bardet-biedl syndrome 7 protein [Chlamydomonas reinhardtii] | 1.00E-178 | cre:CHLREDRAFT_190054 | K16749 |
| TRINITY_DN26909_c2_g1_i1 | 0.000381161 | 1.3855 | XP_002954744.1 | hypothetical protein VOLCADRAFT_65163 [Volvox carteri f. nagariensis] | 1.00E-106 | vcn:VOLCADRAFT_65163 | K01809 |
| TRINITY_DN45437_c1_g5_i1 | 0 | 1.38794 | XP_001695628.1 | hypothetical protein CHLREDRAFT_119255, partial [Chlamydomonasreinhardtii] | 1.00E-132 | cre:CHLREDRAFT_119255 | K10406 |
| TRINITY_DN57783_c0_g1_i1 | 2.95E-13 | 1.40451 | XP_002952894.1 | Argonaute-like protein [Volvox carteri f. nagariensis] | 0 | vcn:VOLCADRAFT_105714 | K11593 |
| TRINITY_DN13945_c0_g2_i1 | 4.79E-05 | 1.41198 | XP_002947059.1 | aurora like protein kinase [Volvox carteri f. nagariensis] | 1.00E-114 | vcn:VOLCADRAFT_127400 | K08850 |
| TRINITY_DN50606_c0_g1_i1 | 0 | 1.41491 | XP_002956562.1 | hypothetical protein VOLCADRAFT_107303 [Volvox carteri f.nagariensis] | 7.00E-33 | vcn:VOLCADRAFT_107303 | K15171 |
| TRINITY_DN19372_c1_g1_i1 | 7.42E-08 | 1.41967 | XP_010526263.1 | PREDICTED: serine/arginine-rich splicing factor RSZ22-like[Tarenaya hassleriana] | 3.00E-18 | thj:104803867 | K12896 |
| TRINITY_DN9067_c0_g2_i1 | 0.00557983 | 1.41981 | XP_001696917.1 | DNA replication factor C complex subunit 1, partial [Chlamydomonasreinhardtii] | 0 | cre:CHLREDRAFT_150793 | K10754 |
| TRINITY_DN50544_c1_g4_i1 | 1.62E-08 | 1.42022 | XP_001695734.1 | CDF transporter, membrane protein [Chlamydomonas reinhardtii] | 6.00E-47 | cre:CHLREDRAFT_205987 | K14689 |
| TRINITY_DN57801_c1_g1_i1 | 8.56E-14 | 1.42049 | XP_002953447.1 | hypothetical protein VOLCADRAFT_82287 [Volvox carteri f. nagariensis] | 1.00E-159 | vcn:VOLCADRAFT_82287 | K00145 |
| TRINITY_DN46600_c0_g1_i3 | 0.00268798 | 1.42809 | XP_005843936.1 | hypothetical protein CHLNCDRAFT_37165 [Chlorella variabilis] | 1.00E-101 | cvr:CHLNCDRAFT_37165 | K01008 |
| TRINITY_DN48126_c2_g1_i1 | 1.00E-07 | 1.42984 | XP_002955216.1 | hypothetical protein VOLCADRAFT_83067 [Volvox carteri f. nagariensis] | 0 | vcn:VOLCADRAFT_83067 | K00366 |
| TRINITY_DN39611_c0_g1_i2 | 3.73E-06 | 1.44347 | XP_001697602.1 | 5-Formyltetrahydrofolate cycloligase [Chlamydomonas reinhardtii] | 6.00E-20 | cre:CHLREDRAFT_196694 | K01934 |
| TRINITY_DN2134_c1_g2_i1 | 4.55E-06 | 1.44387 | XP_001697436.1 | predicted protein [Chlamydomonas reinhardtii] | 1.00E-50 | cre:CHLREDRAFT_81024 | K12833 |
| TRINITY_DN8500_c0_g1_i1 | 4.44E-16 | 1.44957 | XP_002952346.1 | hypothetical protein VOLCADRAFT_62507 [Volvox carteri f. nagariensis] | 1.00E-128 | vcn:VOLCADRAFT_62507 | K03921 |
| TRINITY_DN60181_c0_g1_i1 | 0.000486059 | 1.44968 | XP_001695163.1 | dihydrolipoyl dehydrogenase [Chlamydomonas reinhardtii] | 0 | cre:CHLREDRAFT_57890 | K00382 |
| TRINITY_DN39434_c2_g6_i2 | 2.41E-06 | 1.45199 | XP_011397498.1 | DNA repair protein RAD51-like protein 3 [Auxenochlorellaprotothecoides] | 8.00E-42 | apro:F751_2328 | K10870 |
| TRINITY_DN47891_c23_g16_i1 | 0.0406327 | 1.45553 | XP_001698270.1 | nucleoredoxin, partial [Chlamydomonas reinhardtii] | 7.00E-38 | cre:CHLREDRAFT_177214 | K17609 |
| TRINITY_DN11696_c2_g1_i1 | 0.0149472 | 1.45702 | XP_002946111.1 | component of cytosolic 80S ribosome and 60S large subunit [Volvoxcarteri f. nagariensis] | 8.00E-61 | vcn:VOLCADRAFT_72356 | K02889 |
| TRINITY_DN48556_c1_g1_i1 | 0 | 1.46005 | XP_005651138.1 | aldolase [Coccomyxa subellipsoidea C-169] | 3.00E-67 | csl:COCSUDRAFT_64567 | K00616 |
| TRINITY_DN8765_c2_g1_i2 | 0.0467641 | 1.46351 | XP_002947882.1 | hypothetical protein VOLCADRAFT_103657 [Volvox carteri f.nagariensis] | 3.00E-26 | vcn:VOLCADRAFT_103657 | K13168 |
| TRINITY_DN14025_c1_g1_i1 | 0.00362383 | 1.46517 | XP_002953444.1 | hypothetical protein VOLCADRAFT_63715 [Volvox carteri f.nagariensis] | 5.00E-35 | vcn:VOLCADRAFT_63715 | K14689 |
| TRINITY_DN58061_c0_g1_i1 | 6.33E-06 | 1.4684 | XP_001697575.1 | RNA terminal 3' phosphate cyclase [Chlamydomonas reinhardtii] | 1.00E-112 | cre:CHLREDRAFT_138936 | K11108 |
| TRINITY_DN11590_c1_g1_i2 | 0 | 1.46878 | XP_002949928.1 | hypothetical protein VOLCADRAFT_80842 [Volvox carteri f.nagariensis] | 8.00E-65 | vcn:VOLCADRAFT_80842 | K00670 |
| TRINITY_DN3136_c0_g2_i1 | 0 | 1.47177 | XP_005847260.1 | hypothetical protein CHLNCDRAFT_57944 [Chlorella variabilis] | 1.00E-101 | cvr:CHLNCDRAFT_57944 | K18624 |
| TRINITY_DN6049_c0_g2_i1 | 0 | 1.4718 | XP_001692598.1 | predicted protein [Chlamydomonas reinhardtii] | 0 | cre:CHLREDRAFT_136266 | K01873 |
| TRINITY_DN53345_c0_g2_i1 | 1.64E-07 | 1.47222 | XP_001692808.1 | apoferredoxin [Chlamydomonas reinhardtii] | 4.00E-13 | cre:CHLREDRAFT_147787 | K02639 |
| TRINITY_DN46577_c15_g1_i1 | 0.00377419 | 1.47499 | XP_007017771.1 | 5'-3' exoribonuclease 3 isoform 1 [Theobroma cacao] | 2.00E-16 | tcc:TCM_034205 | K12619 |
| TRINITY_DN11552_c0_g1_i1 | 0.000382689 | 1.47581 | XP_002946216.1 | hypothetical protein VOLCADRAFT_78866 [Volvox carteri f. nagariensis] | 1.00E-138 | vcn:VOLCADRAFT_78866 | K13506 |
| TRINITY_DN36144_c0_g1_i2 | 0.000744223 | 1.48963 | XP_001697505.1 | ubiquitin-protein ligase [Chlamydomonas reinhardtii] | 1.00E-175 | cre:CHLREDRAFT_34875 | K10589 |
| TRINITY_DN42849_c1_g2_i2 | 0.003014 | 1.49005 | XP_002948873.1 | hypothetical protein VOLCADRAFT_58539, partial [Volvox carteri f.nagariensis] | 7.00E-18 | vcn:VOLCADRAFT_58539 | K16274 |
| TRINITY_DN46536_c1_g1_i2 | 1.37E-08 | 1.49328 | XP_005643258.1 | DUF163-domain-containing protein, partial [Coccomyxa subellipsoideaC-169] | 2.00E-46 | csl:COCSUDRAFT_20430 | K00783 |
| TRINITY_DN37055_c1_g1_i1 | 0.0385381 | 1.4934 | XP_002948948.1 | hypothetical protein VOLCADRAFT_89331 [Volvox carteri f. nagariensis] | 8.00E-36 | vcn:VOLCADRAFT_89331 | K12837 |
| TRINITY_DN24728_c2_g3_i1 | 0.0395511 | 1.49352 | XP_001698477.1 | hypothetical protein CHLREDRAFT_142912, partial [Chlamydomonasreinhardtii] | 3.00E-72 | cre:CHLREDRAFT_142912 | K10392 |
| TRINITY_DN4837_c0_g1_i2 | 1.19E-06 | 1.4938 | XP_002951219.1 | hypothetical protein VOLCADRAFT_117772 [Volvox carteri f.nagariensis] | 1.00E-37 | vcn:VOLCADRAFT_117772 | K14947 |
| TRINITY_DN11285_c0_g2_i1 | 2.60E-08 | 1.49623 | XP_002947773.1 | hypothetical protein VOLCADRAFT_116527 [Volvox carteri f.nagariensis] | 5.00E-26 | vcn:VOLCADRAFT_116527 | K09562 |
| TRINITY_DN57938_c2_g1_i1 | 0.0288076 | 1.49814 | XP_005843993.1 | hypothetical protein CHLNCDRAFT_56341 [Chlorella variabilis] | 0 | cvr:CHLNCDRAFT_56341 | K01873 |
| TRINITY_DN5978_c0_g2_i1 | 1.25E-07 | 1.49902 | XP_005647602.1 | glucose-6-phosphate dehydrogenase 1 [Coccomyxa subellipsoidea C-169] | 2.00E-62 | csl:COCSUDRAFT_66151 | K00036 |
| TRINITY_DN44070_c1_g2_i1 | 0.000199575 | 1.50193 | XP_005846746.1 | hypothetical protein CHLNCDRAFT_31596 [Chlorella variabilis] | 1.00E-175 | cvr:CHLNCDRAFT_31596 | K10878 |
| TRINITY_DN2263_c0_g1_i1 | 2.69E-06 | 1.51424 | XP_005642793.1 | hypothetical protein COCSUDRAFT_45509 [Coccomyxa subellipsoideaC-169] | 1.00E-32 | csl:COCSUDRAFT_45509 | K14457 |
| TRINITY_DN50430_c0_g1_i1 | 5.54E-11 | 1.51483 | XP_005852175.1 | hypothetical protein CHLNCDRAFT_59537 [Chlorella variabilis] | 1.00E-133 | cvr:CHLNCDRAFT_59537 | K00208 |
| TRINITY_DN47033_c0_g2_i2 | 1.85E-10 | 1.51725 | XP_001691331.1 | riboflavin kinase [Chlamydomonas reinhardtii] | 1.00E-102 | cre:CHLREDRAFT_128353 | K00861 |
| TRINITY_DN60774_c2_g9_i1 | 0.00708833 | 1.52207 | XP_001699805.1 | Mg2+ transporter protein, CorA-like protein [Chlamydomonasreinhardtii] | 1.00E-11 | cre:CHLREDRAFT_186922 | K16075 |
| TRINITY_DN28079_c1_g4_i1 | 0.000100193 | 1.52219 | XP_006366830.1 | PREDICTED: serine carboxypeptidase-like 20 [Solanum tuberosum] | 1.00E-122 | sot:102596886 | K16296 |
| TRINITY_DN53580_c2_g1_i1 | 0 | 1.52604 | XP_001692808.1 | apoferredoxin [Chlamydomonas reinhardtii] | 2.00E-21 | cre:CHLREDRAFT_147787 | K02639 |
| TRINITY_DN47485_c10_g1_i1 | 0 | 1.53265 | XP_002953163.1 | hypothetical protein VOLCADRAFT_118300, partial [Volvox carteri f.nagariensis] | 2.00E-99 | vcn:VOLCADRAFT_118300 | K14779 |
| TRINITY_DN28478_c0_g2_i1 | 0 | 1.53464 | XP_001702590.1 | NADH:ubiquinone oxidoreductase 51 kDa subunit [Chlamydomonasreinhardtii] | 0 | cre:CHLREDRAFT_186342 | K03942 |
| TRINITY_DN19372_c1_g1_i3 | 2.24E-05 | 1.53515 | XP_010526263.1 | PREDICTED: serine/arginine-rich splicing factor RSZ22-like[Tarenaya hassleriana] | 4.00E-18 | thj:104803867 | K12896 |
| TRINITY_DN4478_c0_g1_i1 | 0 | 1.53754 | XP_002945622.1 | hypothetical protein VOLCADRAFT_85780 [Volvox carteri f. nagariensis] | 0 | vcn:VOLCADRAFT_85780 | K08653 |
| TRINITY_DN60238_c1_g1_i1 | 1.33E-10 | 1.53795 | XP_001695742.1 | cystathionine gamma-synthase [Chlamydomonas reinhardtii] | 1.00E-166 | cre:CHLREDRAFT_24268 | K01739 |
| TRINITY_DN45314_c0_g3_i1 | 0.0225252 | 1.53951 | XP_005651734.1 | FAD/NAD(P)-binding domain-containing protein [Coccomyxasubellipsoidea C-169] | 7.00E-44 | csl:COCSUDRAFT_64116 | K00486 |
| TRINITY_DN44825_c12_g14_i4 | 0.00166072 | 1.54176 | XP_005644542.1 | glycoside hydrolase [Coccomyxa subellipsoidea C-169] | 6.00E-21 | csl:COCSUDRAFT_44382 | K19355 |
| TRINITY_DN46625_c5_g1_i1 | 0 | 1.54618 | XP_002958505.1 | hypothetical protein VOLCADRAFT_108163 [Volvox carteri f.nagariensis] | 1.00E-177 | vcn:VOLCADRAFT_108163 | K11308 |
| TRINITY_DN60100_c0_g3_i1 | 1.73E-09 | 1.54831 | XP_002954903.1 | hypothetical protein VOLCADRAFT_82992 [Volvox carteri f. nagariensis] | 1.00E-156 | vcn:VOLCADRAFT_82992 | K01889 |
| TRINITY_DN60140_c0_g1_i1 | 1.13E-12 | 1.55239 | XP_001695780.1 | 26S proteasome regulatory subunit [Chlamydomonas reinhardtii] | 0 | cre:CHLREDRAFT_137945 | K03028 |
| TRINITY_DN50708_c0_g1_i1 | 5.00E-10 | 1.55328 | XP_005648825.1 | hypothetical protein COCSUDRAFT_28729 [Coccomyxa subellipsoideaC-169] | 0 | csl:COCSUDRAFT_28729 | K10610 |
| TRINITY_DN11288_c0_g1_i1 | 7.31E-09 | 1.55668 | XP_012478324.1 | PREDICTED: calcium-dependent protein kinase 17-like [Gossypiumraimondii] | 2.00E-88 | gra:105793949 | K13412 |
| TRINITY_DN47399_c6_g1_i4 | 0.00153071 | 1.55719 | XP_001689583.1 | predicted protein [Chlamydomonas reinhardtii] | 3.00E-56 | cre:CHLREDRAFT_171763 | K11858 |
| TRINITY_DN55313_c0_g1_i1 | 0 | 1.5614 | XP_001695651.1 | hypothetical protein CHLREDRAFT_119290 [Chlamydomonas reinhardtii] | 0 | cre:CHLREDRAFT_119290 | K01887 |
| TRINITY_DN15453_c1_g1_i1 | 0.031007 | 1.56454 | XP_011397955.1 | Lysine-specific histone demethylase 1A [Auxenochlorellaprotothecoides] | 4.00E-12 | apro:F751_1946 | K11450 |
| TRINITY_DN46311_c13_g2_i16 | 7.53E-05 | 1.56748 | XP_001689583.1 | predicted protein [Chlamydomonas reinhardtii] | 3.00E-55 | cre:CHLREDRAFT_171763 | K11858 |
| TRINITY_DN41036_c0_g2_i3 | 0.00716875 | 1.57381 | XP_002947178.1 | hypothetical protein VOLCADRAFT_56687 [Volvox carteri f. nagariensis] | 1.00E-21 | vcn:VOLCADRAFT_56687 | K00799 |
| TRINITY_DN2488_c5_g1_i1 | 9.69E-13 | 1.57666 | XP_001692808.1 | apoferredoxin [Chlamydomonas reinhardtii] | 4.00E-37 | cre:CHLREDRAFT_147787 | K02639 |
| TRINITY_DN12252_c0_g1_i1 | 0 | 1.57813 | XP_001695916.1 | GDP-D-mannose pyrophosphorylase [Chlamydomonas reinhardtii] | 1.00E-176 | cre:CHLREDRAFT_130417 | K00966 |
| TRINITY_DN55273_c1_g1_i1 | 1.28E-07 | 1.57815 | XP_002956811.1 | hypothetical protein VOLCADRAFT_83790 [Volvox carteri f. nagariensis] | 1.00E-179 | vcn:VOLCADRAFT_83790 | K09458 |
| TRINITY_DN47380_c3_g1_i1 | 0 | 1.57838 | XP_001701501.1 | predicted protein [Chlamydomonas reinhardtii] | 1.00E-106 | cre:CHLREDRAFT_59969 | K13237 |
| TRINITY_DN57712_c1_g1_i1 | 1.61E-10 | 1.58923 | XP_002949374.1 | hypothetical protein VOLCADRAFT_89775 [Volvox carteri f. nagariensis] | 2.00E-63 | vcn:VOLCADRAFT_89775 | K19400 |
| TRINITY_DN43106_c8_g2_i1 | 0.0028225 | 1.59407 | XP_002954505.1 | hypothetical protein VOLCADRAFT_95405 [Volvox carteri f.nagariensis] | 8.00E-21 | vcn:VOLCADRAFT_95405 | K00464 |
| TRINITY_DN13526_c0_g1_i2 | 5.59E-11 | 1.60474 | XP_005645299.1 | hypothetical protein COCSUDRAFT_25064 [Coccomyxa subellipsoideaC-169] | 8.00E-37 | csl:COCSUDRAFT_25064 | K07238 |
| TRINITY_DN47016_c7_g4_i10 | 4.33E-15 | 1.60981 | XP_002945867.1 | hypothetical protein VOLCADRAFT_120224 [Volvox carteri f.nagariensis] | 1.00E-167 | vcn:VOLCADRAFT_120224 | K14835 |
| TRINITY_DN39058_c0_g1_i1 | 0 | 1.61694 | XP_005648575.1 | hypothetical protein COCSUDRAFT_65707 [Coccomyxa subellipsoideaC-169] | 3.00E-87 | csl:COCSUDRAFT_65707 | K10586 |
| TRINITY_DN53150_c0_g1_i1 | 0 | 1.62043 | XP_001695043.1 | chloroplast outer envelope protein [Chlamydomonas reinhardtii] | 0 | cre:CHLREDRAFT_195498 | K07277 |
| TRINITY_DN46637_c4_g4_i2 | 8.46E-13 | 1.63688 | XP_002950024.1 | hypothetical protein VOLCADRAFT_117441 [Volvox carteri f.nagariensis] | 1.00E-18 | vcn:VOLCADRAFT_117441 | K00858 |
| TRINITY_DN50432_c1_g1_i1 | 1.60E-09 | 1.64148 | XP_001703494.1 | UDP-D-glucuronic acid decarboxylase [Chlamydomonas reinhardtii] | 1.00E-153 | cre:CHLREDRAFT_135101 | K08678 |
| TRINITY_DN44117_c1_g1_i7 | 4.00E-14 | 1.64967 | XP_002957234.1 | hypothetical protein VOLCADRAFT_77474, partial [Volvox carteri f.nagariensis] | 1.00E-42 | vcn:VOLCADRAFT_77474 | K18163 |
| TRINITY_DN42665_c1_g1_i3 | 0 | 1.6571 | XP_005650241.1 | hydroxymethylglutaryl-CoA synthase [Coccomyxa subellipsoidea C-169] | 6.00E-86 | csl:COCSUDRAFT_27385 | K01641 |
| TRINITY_DN46311_c13_g2_i17 | 1.08E-06 | 1.66346 | XP_002955394.1 | guanylyl and adenylyl cyclase family member [Volvox carteri f.nagariensis] | 1.00E-30 | vcn:VOLCADRAFT_106794 | K11858 |
| TRINITY_DN47779_c14_g4_i1 | 1.54E-05 | 1.66448 | XP_002951809.1 | hypothetical protein VOLCADRAFT_81595 [Volvox carteri f. nagariensis] | 0 | vcn:VOLCADRAFT_81595 | K14537 |
| TRINITY_DN23056_c1_g1_i1 | 0 | 1.66463 | XP_002954352.1 | hypothetical protein VOLCADRAFT_76255 [Volvox carteri f. nagariensis] | 1.00E-145 | vcn:VOLCADRAFT_76255 | K16292 |
| TRINITY_DN47253_c0_g2_i16 | 6.95E-05 | 1.66547 | XP_001702980.1 | ribosomal protein S11, component of cytosolic 80S ribosome and 40Ssmall subunit [Chlamydomonas reinhardtii] | 1.00E-69 | cre:CHLREDRAFT_180130 | K02949 |
| TRINITY_DN6471_c1_g1_i1 | 4.60E-05 | 1.66578 | XP_005642793.1 | hypothetical protein COCSUDRAFT_45509 [Coccomyxa subellipsoideaC-169] | 8.00E-40 | csl:COCSUDRAFT_45509 | K14457 |
| TRINITY_DN196_c1_g1_i1 | 6.99E-15 | 1.67349 | XP_002950232.1 | hypothetical protein VOLCADRAFT_109070 [Volvox carteri f.nagariensis] | 1.00E-35 | vcn:VOLCADRAFT_109070 | K12624 |
| TRINITY_DN39102_c1_g1_i1 | 6.60E-06 | 1.68413 | XP_005644898.1 | target of rapamycin kinase [Coccomyxa subellipsoidea C-169] | 0 | csl:COCSUDRAFT_30586 | K07203 |
| TRINITY_DN57810_c1_g1_i1 | 0 | 1.68666 | XP_002946328.1 | hypothetical protein VOLCADRAFT_102923 [Volvox carteri f.nagariensis] | 5.00E-39 | vcn:VOLCADRAFT_102923 | K19720 |
| TRINITY_DN9037_c1_g1_i1 | 2.25E-05 | 1.69044 | XP_001697267.1 | half-size ABC transporter, membrane protein, partial [Chlamydomonasreinhardtii] | 0 | cre:CHLREDRAFT_105113 | K05663 |
| TRINITY_DN45628_c0_g1_i5 | 3.46E-05 | 1.69231 | XP_001698065.1 | receptor of activated protein kinase C 1 [Chlamydomonas reinhardtii] | 1.00E-164 | cre:CHLREDRAFT_105734 | K14753 |
| TRINITY_DN34136_c6_g1_i1 | 0 | 1.69487 | XP_001697084.1 | adenylylphosphosulfate reductase [Chlamydomonas reinhardtii] | 1.00E-165 | cre:CHLREDRAFT_131444 | K05907 |
| TRINITY_DN46475_c1_g1_i2 | 1.33E-05 | 1.7044 | XP_005649356.1 | radical SAM enzyme [Coccomyxa subellipsoidea C-169] | 1.00E-17 | csl:COCSUDRAFT_36095 | K02495 |
| TRINITY_DN43331_c1_g2_i7 | 0.000170733 | 1.70583 | XP_001691858.1 | hypothetical protein CHLREDRAFT_136100 [Chlamydomonas reinhardtii] | 1.00E-175 | cre:CHLREDRAFT_136100 | K13177 |
| TRINITY_DN43393_c4_g1_i5 | 3.67E-10 | 1.70748 | XP_001701499.1 | DNA repair glycosylase, partial [Chlamydomonas reinhardtii] | 4.00E-86 | cre:CHLREDRAFT_153844 | K10773 |
| TRINITY_DN27323_c1_g1_i1 | 0 | 1.70842 | XP_002951881.1 | hypothetical protein VOLCADRAFT_105295 [Volvox carteri f.nagariensis] | 1.00E-168 | vcn:VOLCADRAFT_105295 | K00963 |
| TRINITY_DN45615_c6_g3_i8 | 6.83E-08 | 1.70853 | XP_011399296.1 | Nudix hydrolase 23, chloroplastic [Auxenochlorella protothecoides] | 1.00E-41 | apro:F751_4893 | K18453 |
| TRINITY_DN37407_c0_g1_i2 | 0.00169256 | 1.71452 | XP_002948620.1 | hypothetical protein VOLCADRAFT_103955 [Volvox carteri f.nagariensis] | 2.00E-97 | vcn:VOLCADRAFT_103955 | K01456 |
| TRINITY_DN46931_c0_g1_i1 | 0.00483206 | 1.71836 | XP_002956610.1 | hypothetical protein VOLCADRAFT_67166, partial [Volvox carteri f.nagariensis] | 3.00E-94 | vcn:VOLCADRAFT_67166 | K11835 |
| TRINITY_DN47759_c0_g1_i4 | 4.41E-05 | 1.71858 | XP_005645275.1 | chloroplast fructose-1,6-bisphosphatase II [Coccomyxasubellipsoidea C-169] | 1.00E-50 | csl:COCSUDRAFT_57295 | K03841 |
| TRINITY_DN60247_c0_g4_i1 | 0.00169054 | 1.72149 | XP_001699617.1 | elongation factor EF-Tu-like protein [Chlamydomonas reinhardtii] | 0 | cre:CHLREDRAFT_24423 | K12852 |
| TRINITY_DN24676_c0_g2_i1 | 1.32E-05 | 1.72344 | XP_001694660.1 | dynein heavy chain 2 [Chlamydomonas reinhardtii] | 0 | cre:CHLREDRAFT_130324 | K10408 |
| TRINITY_DN41435_c0_g2_i2 | 5.83E-11 | 1.72505 | XP_001701816.1 | threonine deaminase [Chlamydomonas reinhardtii] | 0 | cre:CHLREDRAFT_196595 | K01754 |
| TRINITY_DN30745_c2_g1_i1 | 0 | 1.73397 | XP_002955080.1 | hypothetical protein VOLCADRAFT_33691, partial [Volvox carteri f.nagariensis] | 6.00E-78 | vcn:VOLCADRAFT_33691 | K05941 |
| TRINITY_DN44677_c2_g2_i1 | 0 | 1.74739 | XP_002949401.1 | hypothetical protein VOLCADRAFT_89829 [Volvox carteri f. nagariensis] | 1.00E-100 | vcn:VOLCADRAFT_89829 | K03023 |
| TRINITY_DN45661_c6_g3_i3 | 3.66E-08 | 1.75837 | XP_001689583.1 | predicted protein [Chlamydomonas reinhardtii] | 1.00E-60 | cre:CHLREDRAFT_171763 | K11858 |
| TRINITY_DN26804_c1_g1_i1 | 1.80E-05 | 1.75916 | XP_001769381.1 | predicted protein [Physcomitrella patens] | 7.00E-93 | ppp:PHYPADRAFT_134776 | K01369 |
| TRINITY_DN48855_c0_g1_i1 | 0.0213949 | 1.76198 | XP_001702843.1 | spermidine synthase [Chlamydomonas reinhardtii] | 1.00E-114 | cre:CHLREDRAFT_206050 | K00797 |
| TRINITY_DN50469_c1_g1_i1 | 0 | 1.76205 | XP_005844242.1 | hypothetical protein CHLNCDRAFT_139272 [Chlorella variabilis] | 3.00E-48 | cvr:CHLNCDRAFT_139272 | K11835 |
| TRINITY_DN42480_c3_g3_i1 | 0.0448546 | 1.76409 | XP_002947795.1 | hypothetical protein VOLCADRAFT_103576 [Volvox carteri f.nagariensis] | 2.00E-25 | vcn:VOLCADRAFT_103576 | K14404 |
| TRINITY_DN38941_c0_g1_i2 | 2.58E-08 | 1.76806 | XP_001694423.1 | DNA polymerase zeta, partial [Chlamydomonas reinhardtii] | 1.00E-155 | cre:CHLREDRAFT_117843 | K02350 |
| TRINITY_DN47290_c1_g2_i3 | 5.83E-05 | 1.77387 | XP_002955427.1 | hypothetical protein VOLCADRAFT_109969 [Volvox carteri f.nagariensis] | 1.00E-86 | vcn:VOLCADRAFT_109969 | K01803 |
| TRINITY_DN8950_c0_g3_i1 | 0.000149614 | 1.77473 | XP_005848921.1 | hypothetical protein CHLNCDRAFT_144365 [Chlorella variabilis] | 4.00E-41 | cvr:CHLNCDRAFT_144365 | K15340 |
| TRINITY_DN48837_c2_g1_i1 | 0.00613811 | 1.78782 | XP_002955656.1 | hypothetical protein VOLCADRAFT_96541 [Volvox carteri f.nagariensis] | 1.00E-27 | vcn:VOLCADRAFT_96541 | K12244 |
| TRINITY_DN53345_c0_g1_i1 | 0.000154126 | 1.80226 | XP_001692808.1 | apoferredoxin [Chlamydomonas reinhardtii] | 2.00E-13 | cre:CHLREDRAFT_147787 | K02639 |
| TRINITY_DN12735_c2_g1_i1 | 0 | 1.80601 | XP_002954591.1 | NADH:ubiquinone oxidoreductase 39 kDa subunit [Volvox carteri f.nagariensis] | 1.00E-125 | vcn:VOLCADRAFT_106450 | K03953 |
| TRINITY_DN5213_c0_g1_i1 | 8.37E-12 | 1.80976 | XP_002954629.1 | flagellar alpha dynein [Volvox carteri f. nagariensis] | 0 | vcn:VOLCADRAFT_76397 | K10408 |
| TRINITY_DN12101_c0_g1_i1 | 2.98E-09 | 1.82158 | XP_005844749.1 | malate dehydrogenase, cytoplasmic [Chlorella variabilis] | 1.00E-114 | cvr:CHLNCDRAFT_32420 | K00025 |
| TRINITY_DN42771_c0_g2_i1 | 1.55E-15 | 1.82195 | XP_008454767.1 | PREDICTED: annexin D5-like [Cucumis melo] | 2.00E-23 | cmo:103495087 | K17095 |
| TRINITY_DN30464_c0_g1_i3 | 4.26E-08 | 1.82501 | XP_001691098.1 | predicted protein [Chlamydomonas reinhardtii] | 6.00E-64 | cre:CHLREDRAFT_94247 | K01193 |
| TRINITY_DN47086_c3_g24_i1 | 0 | 1.82954 | XP_001693810.1 | histone methyltransferase, partial [Chlamydomonas reinhardtii] | 2.00E-12 | cre:CHLREDRAFT_143611 | K11426 |
| TRINITY_DN39611_c0_g1_i4 | 6.22E-10 | 1.83304 | XP_001697602.1 | 5-Formyltetrahydrofolate cycloligase [Chlamydomonas reinhardtii] | 3.00E-55 | cre:CHLREDRAFT_196694 | K01934 |
| TRINITY_DN48084_c0_g1_i1 | 1.79E-07 | 1.83974 | XP_002954700.1 | hypothetical protein VOLCADRAFT_64976 [Volvox carteri f. nagariensis] | 0 | vcn:VOLCADRAFT_64976 | K17681 |
| TRINITY_DN17396_c0_g2_i1 | 0 | 1.8443 | XP_002954675.1 | hypothetical protein VOLCADRAFT_82885 [Volvox carteri f. nagariensis] | 1.00E-127 | vcn:VOLCADRAFT_82885 | K00654 |
| TRINITY_DN60338_c0_g1_i1 | 2.08E-09 | 1.85251 | XP_002955656.1 | hypothetical protein VOLCADRAFT_96541 [Volvox carteri f.nagariensis] | 1.00E-14 | vcn:VOLCADRAFT_96541 | K12244 |
| TRINITY_DN2439_c2_g2_i1 | 0.00479111 | 1.86378 | XP_001702980.1 | ribosomal protein S11, component of cytosolic 80S ribosome and 40Ssmall subunit [Chlamydomonas reinhardtii] | 2.00E-69 | cre:CHLREDRAFT_180130 | K02949 |
| TRINITY_DN33641_c0_g1_i1 | 0.00641126 | 1.86679 | XP_001694767.1 | methyltransferase [Chlamydomonas reinhardtii] | 5.00E-34 | cre:CHLREDRAFT_130314 | K03501 |
| TRINITY_DN19618_c0_g1_i1 | 1.54E-09 | 1.8671 | XP_002950112.1 | hypothetical protein VOLCADRAFT_60063 [Volvox carteri f. nagariensis] | 0 | vcn:VOLCADRAFT_60063 | K14776 |
| TRINITY_DN47831_c8_g7_i6 | 0.0165316 | 1.87078 | XP_001697462.1 | chorismate mutase [Chlamydomonas reinhardtii] | 2.00E-82 | cre:CHLREDRAFT_196317 | K01850 |
| TRINITY_DN34339_c1_g2_i1 | 0.0214149 | 1.87086 | XP_003549964.1 | PREDICTED: lon protease homolog 2, peroxisomal-like [Glycine max] | 4.00E-74 | gmx:100789982 | K01338 |
| TRINITY_DN6240_c1_g1_i1 | 0.000447003 | 1.90018 | XP_001696681.1 | uroporphyrinogen-iii synthase [Chlamydomonas reinhardtii] | 2.00E-83 | cre:CHLREDRAFT_195943 | K01719 |
| TRINITY_DN44646_c5_g2_i1 | 0.0168663 | 1.91677 | XP_001697738.1 | methionine aminopeptidase [Chlamydomonas reinhardtii] | 2.00E-60 | cre:CHLREDRAFT_131477 | K01265 |
| TRINITY_DN47016_c7_g4_i3 | 0 | 1.93202 | XP_002945867.1 | hypothetical protein VOLCADRAFT_120224 [Volvox carteri f.nagariensis] | 1.00E-167 | vcn:VOLCADRAFT_120224 | K14835 |
| TRINITY_DN27323_c1_g2_i1 | 4.94E-05 | 1.94865 | XP_002951881.1 | hypothetical protein VOLCADRAFT_105295 [Volvox carteri f.nagariensis] | 1.00E-91 | vcn:VOLCADRAFT_105295 | K00963 |
| TRINITY_DN48187_c1_g1_i1 | 0 | 1.96793 | XP_005847622.1 | hypothetical protein CHLNCDRAFT_23208 [Chlorella variabilis] | 1.00E-113 | cvr:CHLNCDRAFT_23208 | K14376 |
| TRINITY_DN47186_c0_g4_i5 | 4.43E-13 | 1.97949 | XP_001699781.1 | eukaryotic initiation factor [Chlamydomonas reinhardtii] | 2.00E-52 | cre:CHLREDRAFT_141865 | K03236 |
| TRINITY_DN60076_c0_g1_i1 | 0 | 1.97949 | XP_001692963.1 | CDP-Ethanolamine synthase [Chlamydomonas reinhardtii] | 1.00E-173 | cre:CHLREDRAFT_136865 | K00967 |
| TRINITY_DN60076_c0_g2_i1 | 0 | 1.98951 | XP_001692963.1 | CDP-Ethanolamine synthase [Chlamydomonas reinhardtii] | 1.00E-173 | cre:CHLREDRAFT_136865 | K00967 |
| TRINITY_DN47623_c5_g1_i1 | 1.74E-06 | 1.99149 | XP_002947410.1 | hypothetical protein VOLCADRAFT_79713 [Volvox carteri f. nagariensis] | 0 | vcn:VOLCADRAFT_79713 | K01938 |
| TRINITY_DN39260_c4_g1_i1 | 0.0154042 | 2.00404 | XP_001692205.1 | hypothetical protein CHLREDRAFT_128764, partial [Chlamydomonasreinhardtii] | 1.00E-142 | cre:CHLREDRAFT_128764 | K00864 |
| TRINITY_DN4879_c0_g1_i1 | 0 | 2.00456 | XP_001759999.1 | predicted protein, partial [Physcomitrella patens] | 1.00E-14 | ppp:PHYPADRAFT_19499 | K03127 |
| TRINITY_DN41923_c1_g1_i1 | 0.00124136 | 2.03216 | XP_002949357.1 | hypothetical protein VOLCADRAFT_59362 [Volvox carteri f. nagariensis] | 4.00E-58 | vcn:VOLCADRAFT_59362 | K13131 |
| TRINITY_DN39611_c0_g1_i6 | 1.32E-05 | 2.03252 | XP_005643121.1 | 5-Formyltetrahydrofolate cycloligase [Coccomyxa subellipsoidea C-169] | 2.00E-19 | csl:COCSUDRAFT_31705 | K01934 |
| TRINITY_DN47022_c4_g5_i1 | 4.88E-12 | 2.03525 | XP_002948125.1 | hypothetical protein VOLCADRAFT_116697 [Volvox carteri f.nagariensis] | 1.00E-91 | vcn:VOLCADRAFT_116697 | K13281 |
| TRINITY_DN40206_c0_g3_i2 | 1.34E-05 | 2.0463 | XP_001695465.1 | Rh protein [Chlamydomonas reinhardtii] | 2.00E-20 | cre:CHLREDRAFT_24240 | K06580 |
| TRINITY_DN38948_c2_g3_i2 | 0.00365651 | 2.05144 | XP_004972771.1 | PREDICTED: choline-phosphate cytidylyltransferase 2-like [Setariaitalica] | 4.00E-36 | sita:101753030 | K00968 |
| TRINITY_DN43175_c1_g1_i2 | 0.0475239 | 2.07218 | XP_001695465.1 | Rh protein [Chlamydomonas reinhardtii] | 1.00E-105 | cre:CHLREDRAFT_24240 | K06580 |
| TRINITY_DN53048_c0_g2_i1 | 0 | 2.07363 | XP_005847083.1 | hypothetical protein CHLNCDRAFT_24199 [Chlorella variabilis] | 0 | cvr:CHLNCDRAFT_24199 | K01881 |
| TRINITY_DN44070_c1_g1_i1 | 3.29E-05 | 2.07399 | XP_005647003.1 | topoisomerase [Coccomyxa subellipsoidea C-169] | 3.00E-60 | csl:COCSUDRAFT_42760 | K10878 |
| TRINITY_DN46030_c0_g2_i1 | 0.000937409 | 2.10503 | XP_001702496.1 | predicted protein [Chlamydomonas reinhardtii] | 3.00E-86 | cre:CHLREDRAFT_165793 | K00858 |
| TRINITY_DN27378_c1_g2_i3 | 0.000571836 | 2.10553 | XP_002946288.1 | Bardet-Biedl syndrome 5 [Volvox carteri f. nagariensis] | 3.00E-69 | vcn:VOLCADRAFT_78967 | K16748 |
| TRINITY_DN46172_c1_g1_i3 | 0.00211184 | 2.10769 | XP_002954837.1 | hypothetical protein VOLCADRAFT_82983 [Volvox carteri f. nagariensis] | 3.00E-23 | vcn:VOLCADRAFT_82983 | K04505 |
| TRINITY_DN17364_c1_g1_i1 | 0 | 2.11216 | XP_002950098.1 | hypothetical protein VOLCADRAFT_104604 [Volvox carteri f.nagariensis] | 1.00E-160 | vcn:VOLCADRAFT_104604 | K12842 |
| TRINITY_DN9040_c0_g1_i1 | 0 | 2.13529 | XP_002950024.1 | hypothetical protein VOLCADRAFT_117441 [Volvox carteri f.nagariensis] | 1.00E-121 | vcn:VOLCADRAFT_117441 | K00858 |
| TRINITY_DN45757_c7_g1_i2 | 2.96E-05 | 2.15473 | XP_005647335.1 | putative GDP-L-fucose synthetase [Coccomyxa subellipsoidea C-169] | 3.00E-79 | csl:COCSUDRAFT_24021 | K02377 |
| TRINITY_DN44677_c2_g1_i1 | 6.24E-06 | 2.18294 | XP_001695819.1 | hypothetical protein CHLREDRAFT_174488 [Chlamydomonas reinhardtii] | 3.00E-21 | cre:CHLREDRAFT_174488 | K03023 |
| TRINITY_DN6472_c1_g1_i1 | 0 | 2.18631 | XP_005643723.1 | hypothetical protein COCSUDRAFT_19880 [Coccomyxa subellipsoideaC-169] | 1.00E-140 | csl:COCSUDRAFT_19880 | K07561 |
| TRINITY_DN44825_c12_g14_i6 | 0.0026564 | 2.22002 | XP_005644542.1 | glycoside hydrolase [Coccomyxa subellipsoidea C-169] | 5.00E-21 | csl:COCSUDRAFT_44382 | K19355 |
| TRINITY_DN46016_c1_g2_i1 | 0.00500315 | 2.229 | XP_002955511.1 | hypothetical protein VOLCADRAFT_83295 [Volvox carteri f.nagariensis] | 1.00E-47 | vcn:VOLCADRAFT_83295 | K15103 |
| TRINITY_DN45801_c2_g1_i5 | 5.10E-05 | 2.27387 | XP_002958969.1 | C type cyclin [Volvox carteri f. nagariensis] | 1.00E-41 | vcn:VOLCADRAFT_127505 | K15161 |
| TRINITY_DN45505_c0_g2_i4 | 1.05E-06 | 2.35716 | XP_002954671.1 | hypothetical protein VOLCADRAFT_106463 [Volvox carteri f.nagariensis] | 2.00E-46 | vcn:VOLCADRAFT_106463 | K15255 |
| TRINITY_DN45345_c0_g1_i3 | 0.00102287 | 2.36349 | XP_002947706.1 | Rieske iron-sulfur subunit of the cytochrome b6f complex,chloroplast precursor [Volvox carteri f. nagariensis] | 9.00E-64 | vcn:VOLCADRAFT_109628 | K02636 |
| TRINITY_DN45387_c1_g1_i2 | 1.88E-13 | 2.36564 | XP_002951134.1 | hypothetical protein VOLCADRAFT_104984 [Volvox carteri f.nagariensis] | 1.00E-51 | vcn:VOLCADRAFT_104984 | K01094 |
| TRINITY_DN46536_c1_g5_i2 | 0.00801295 | 2.3665 | XP_001694243.1 | phosphate/phosphoenolpyruvate translocator [Chlamydomonasreinhardtii] | 1.00E-104 | cre:CHLREDRAFT_191135 | K15283 |
| TRINITY_DN38314_c0_g2_i3 | 1.52E-08 | 2.39311 | XP_005651667.1 | peptidase C54 [Coccomyxa subellipsoidea C-169] | 5.00E-29 | csl:COCSUDRAFT_55147 | K08342 |
| TRINITY_DN46311_c13_g2_i13 | 0.00290477 | 2.42234 | XP_001689583.1 | predicted protein [Chlamydomonas reinhardtii] | 3.00E-61 | cre:CHLREDRAFT_171763 | K11858 |
| TRINITY_DN47033_c0_g2_i1 | 0.00117683 | 2.44372 | XP_002947022.1 | hypothetical protein VOLCADRAFT_103251 [Volvox carteri f.nagariensis] | 6.00E-16 | vcn:VOLCADRAFT_103251 | K00861 |
| TRINITY_DN46730_c1_g1_i1 | 2.88E-06 | 2.44905 | XP_002957258.1 | hypothetical protein VOLCADRAFT_107595 [Volvox carteri f.nagariensis] | 7.00E-25 | vcn:VOLCADRAFT_107595 | K11147 |
| TRINITY_DN29101_c2_g1_i3 | 0.00640054 | 2.45289 | XP_002954657.1 | hypothetical protein VOLCADRAFT_82855 [Volvox carteri f. nagariensis] | 2.00E-50 | vcn:VOLCADRAFT_82855 | K19090 |
| TRINITY_DN43278_c0_g1_i3 | 0.000294976 | 2.49217 | XP_001693043.1 | diacylglycerol kinase [Chlamydomonas reinhardtii] | 2.00E-94 | cre:CHLREDRAFT_147241 | K04718 |
| TRINITY_DN40908_c0_g1_i2 | 9.32E-05 | 2.5071 | XP_002948733.1 | hypothetical protein VOLCADRAFT_58732, partial [Volvox carteri f.nagariensis] | 9.00E-44 | vcn:VOLCADRAFT_58732 | K00703 |
| TRINITY_DN39611_c0_g1_i3 | 0 | 2.51248 | XP_001697602.1 | 5-Formyltetrahydrofolate cycloligase [Chlamydomonas reinhardtii] | 6.00E-15 | cre:CHLREDRAFT_196694 | K01934 |
| TRINITY_DN47871_c0_g2_i5 | 1.04E-06 | 2.51297 | XP_001698471.1 | hypothetical protein CHLREDRAFT_142899 [Chlamydomonas reinhardtii] | 1.00E-67 | cre:CHLREDRAFT_142899 | K15271 |
| TRINITY_DN44352_c0_g1_i4 | 0.0498283 | 2.59557 | NP_001234990.1 | peroxisomal betaine-aldehyde dehydrogenase [Glycine max] | 1.00E-33 | gmx:100170755 | K00130 |
| TRINITY_DN38354_c0_g2_i1 | 0.0102338 | 2.64034 | XP_001693304.1 | 3'-5' exoribonuclease, partial [Chlamydomonas reinhardtii] | 3.00E-31 | cre:CHLREDRAFT_147450 | K18758 |
| TRINITY_DN42771_c0_g2_i3 | 5.08E-06 | 2.64209 | XP_008454767.1 | PREDICTED: annexin D5-like [Cucumis melo] | 7.00E-16 | cmo:103495087 | K17095 |
| TRINITY_DN42890_c0_g1_i1 | 3.60E-05 | 2.64871 | XP_002949213.1 | phosphofructokinase family protein [Volvox carteri f. nagariensis] | 1.00E-137 | vcn:VOLCADRAFT_74177 | K00850 |
| TRINITY_DN2478_c0_g3_i1 | 9.31E-09 | 2.66058 | XP_002501575.1 | predicted protein [Micromonas sp. RCC299] | 1.00E-144 | mis:MICPUN_57477 | K16747 |
| TRINITY_DN4865_c0_g1_i2 | 0.00553923 | 2.70904 | XP_005851445.1 | hypothetical protein CHLNCDRAFT_137782 [Chlorella variabilis] | 7.00E-58 | cvr:CHLNCDRAFT_137782 | K14759 |
| TRINITY_DN24446_c0_g1_i1 | 0.0455514 | 2.71191 | XP_002950249.1 | hypothetical protein VOLCADRAFT_117497, partial [Volvox carteri f.nagariensis] | 6.00E-35 | vcn:VOLCADRAFT_117497 | K00972 |
| TRINITY_DN35070_c7_g3_i3 | 0 | 2.71777 | XP_005642758.1 | OTU-domain-containing protein [Coccomyxa subellipsoidea C-169] | 1.00E-19 | csl:COCSUDRAFT_20805 | K13719 |
| TRINITY_DN39260_c4_g1_i6 | 0.0358587 | 2.73143 | XP_001692205.1 | hypothetical protein CHLREDRAFT_128764, partial [Chlamydomonasreinhardtii] | 1.00E-142 | cre:CHLREDRAFT_128764 | K00864 |
| TRINITY_DN3595_c0_g2_i1 | 0.00120137 | 2.7491 | XP_002957229.1 | hypothetical protein VOLCADRAFT_77459 [Volvox carteri f.nagariensis] | 4.00E-39 | vcn:VOLCADRAFT_77459 | K01194 |
| TRINITY_DN30586_c1_g1_i1 | 0 | 2.76765 | XP_005650141.1 | splicing factor, CC1-like protein [Coccomyxa subellipsoidea C-169] | 4.00E-44 | csl:COCSUDRAFT_28136 | K13091 |
| TRINITY_DN38354_c0_g1_i1 | 0 | 2.82453 | XP_001693304.1 | 3'-5' exoribonuclease, partial [Chlamydomonas reinhardtii] | 1.00E-162 | cre:CHLREDRAFT_147450 | K18758 |
| TRINITY_DN44984_c1_g1_i2 | 0.00646898 | 2.85607 | XP_002950355.1 | hypothetical protein VOLCADRAFT_90898 [Volvox carteri f. nagariensis] | 2.00E-68 | vcn:VOLCADRAFT_90898 | K18932 |
| TRINITY_DN43393_c4_g1_i7 | 9.05E-05 | 2.86259 | XP_001701499.1 | DNA repair glycosylase, partial [Chlamydomonas reinhardtii] | 7.00E-52 | cre:CHLREDRAFT_153844 | K10773 |
| TRINITY_DN39106_c2_g2_i3 | 0.000576279 | 2.88485 | XP_005846057.1 | hypothetical protein CHLNCDRAFT_136223 [Chlorella variabilis] | 1.00E-16 | cvr:CHLNCDRAFT_136223 | K11807 |
| TRINITY_DN22734_c1_g2_i7 | 0 | 2.93278 | XP_002948667.1 | hypothetical protein VOLCADRAFT_89014 [Volvox carteri f.nagariensis] | 1.00E-42 | vcn:VOLCADRAFT_89014 | K13071 |
| TRINITY_DN46625_c5_g4_i1 | 2.58E-06 | 2.95254 | XP_002958505.1 | hypothetical protein VOLCADRAFT_108163 [Volvox carteri f.nagariensis] | 8.00E-77 | vcn:VOLCADRAFT_108163 | K11308 |
| TRINITY_DN44917_c0_g1_i2 | 0.000547504 | 2.97453 | XP_005644681.1 | DNA/RNA polymerase [Coccomyxa subellipsoidea C-169] | 6.00E-16 | csl:COCSUDRAFT_57864 | K03509 |
| TRINITY_DN8010_c0_g2_i1 | 1.81E-06 | 2.98277 | XP_005646784.1 | ENTH-domain-containing protein [Coccomyxa subellipsoidea C-169] | 3.00E-49 | csl:COCSUDRAFT_42598 | K12471 |
| TRINITY_DN46136_c0_g1_i15 | 0.0315613 | 3.05979 | XP_002951155.1 | hypothetical protein VOLCADRAFT_74917 [Volvox carteri f. nagariensis] | 2.00E-41 | vcn:VOLCADRAFT_74917 | K03235 |
| TRINITY_DN42890_c0_g1_i12 | 0.00265561 | 3.07699 | XP_002949213.1 | phosphofructokinase family protein [Volvox carteri f. nagariensis] | 6.00E-42 | vcn:VOLCADRAFT_74177 | K00850 |
| TRINITY_DN40405_c0_g2_i1 | 9.60E-05 | 3.23672 | XP_002954657.1 | hypothetical protein VOLCADRAFT_82855 [Volvox carteri f. nagariensis] | 1.00E-28 | vcn:VOLCADRAFT_82855 | K19090 |
| TRINITY_DN21444_c0_g3_i1 | 0.000634312 | 3.25134 | XP_002946055.1 | hypothetical protein VOLCADRAFT_78675 [Volvox carteri f. nagariensis] | 1.00E-118 | vcn:VOLCADRAFT_78675 | K11843 |
| TRINITY_DN32044_c1_g2_i1 | 0.000101325 | 3.2713 | XP_001690837.1 | Qa-SNARE protein, Tlg2/Syntaxin16-family [Chlamydomonasreinhardtii] | 5.00E-37 | cre:CHLREDRAFT_195401 | K08489 |
| TRINITY_DN42028_c1_g2_i1 | 0.00844457 | 3.31139 | XP_002945837.1 | hypothetical protein VOLCADRAFT_55451, partial [Volvox carteri f.nagariensis] | 8.00E-57 | vcn:VOLCADRAFT_55451 | K15463 |
| TRINITY_DN45901_c1_g2_i1 | 4.03E-05 | 3.35752 | XP_002958679.1 | hypothetical protein VOLCADRAFT_99968 [Volvox carteri f.nagariensis] | 7.00E-41 | vcn:VOLCADRAFT_99968 | K15287 |
| TRINITY_DN47548_c3_g1_i7 | 0.0121838 | 3.37711 | XP_005644118.1 | hypothetical protein COCSUDRAFT_58323 [Coccomyxa subellipsoideaC-169] | 9.00E-82 | csl:COCSUDRAFT_58323 | K08741 |
| TRINITY_DN34567_c0_g1_i4 | 0.000240048 | 3.40224 | XP_002947957.1 | hypothetical protein VOLCADRAFT_88396 [Volvox carteri f. nagariensis] | 1.00E-25 | vcn:VOLCADRAFT_88396 | K19720 |
| TRINITY_DN44807_c2_g4_i4 | 0.00126952 | 3.44119 | XP_002949271.1 | hypothetical protein VOLCADRAFT_104214 [Volvox carteri f.nagariensis] | 6.00E-15 | vcn:VOLCADRAFT_104214 | K14709 |
| TRINITY_DN43141_c0_g1_i5 | 0.0467656 | 3.50029 | XP_001693122.1 | centriole proteome protein, partial [Chlamydomonas reinhardtii] | 3.00E-45 | cre:CHLREDRAFT_13542 | K16757 |
| TRINITY_DN45615_c5_g7_i5 | 0.000169161 | 3.63332 | XP_002958779.1 | actin-binding protein gelsolin [Volvox carteri f. nagariensis] | 4.00E-14 | vcn:VOLCADRAFT_108308 | K05768 |
| TRINITY_DN47915_c1_g1_i1 | 3.33E-16 | 3.63357 | XP_001783110.1 | predicted protein, partial [Physcomitrella patens] | 1.00E-34 | ppp:PHYPADRAFT_31023 | K01510 |
| TRINITY_DN30586_c1_g1_i2 | 2.22E-16 | 3.74756 | XP_005650141.1 | splicing factor, CC1-like protein [Coccomyxa subellipsoidea C-169] | 4.00E-44 | csl:COCSUDRAFT_28136 | K13091 |
| TRINITY_DN43041_c0_g4_i2 | 0.00145399 | 3.76572 | XP_011398114.1 | Methylcrotonoyl-CoA carboxylase beta chain, mitochondrial[Auxenochlorella protothecoides] | 3.00E-70 | apro:F751_6178 | K01969 |
| TRINITY_DN22758_c0_g2_i1 | 0 | 3.77775 | XP_005650658.1 | DAO-domain-containing protein [Coccomyxa subellipsoidea C-169] | 1.00E-156 | csl:COCSUDRAFT_27656 | K00111 |
| TRINITY_DN38376_c0_g1_i4 | 0.044661 | 3.82647 | XP_001764186.1 | predicted protein, partial [Physcomitrella patens] | 2.00E-25 | ppp:PHYPADRAFT_42418 | K11793 |
| TRINITY_DN31991_c0_g2_i1 | 2.69E-05 | 3.88016 | XP_002950030.1 | hypothetical protein VOLCADRAFT_74462 [Volvox carteri f.nagariensis] | 4.00E-21 | vcn:VOLCADRAFT_74462 | K19682 |
| TRINITY_DN40429_c0_g2_i1 | 0.0143779 | 3.94102 | XP_005643929.1 | P-loop containing nucleoside triphosphate hydrolase protein[Coccomyxa subellipsoidea C-169] | 2.00E-16 | csl:COCSUDRAFT_38242 | K05674 |
| TRINITY_DN43771_c7_g2_i2 | 2.07E-05 | 4.01509 | XP_002947447.1 | hypothetical protein VOLCADRAFT_120448 [Volvox carteri f.nagariensis] | 2.00E-29 | vcn:VOLCADRAFT_120448 | K10863 |
| TRINITY_DN47380_c3_g1_i3 | 1.11E-16 | 4.05056 | XP_001701501.1 | predicted protein [Chlamydomonas reinhardtii] | 7.00E-75 | cre:CHLREDRAFT_59969 | K13237 |
| TRINITY_DN43141_c0_g1_i6 | 3.97E-06 | 4.21439 | XP_001693122.1 | centriole proteome protein, partial [Chlamydomonas reinhardtii] | 3.00E-53 | cre:CHLREDRAFT_13542 | K16757 |
| TRINITY_DN47022_c4_g5_i4 | 5.37E-06 | 4.26501 | XP_002948125.1 | hypothetical protein VOLCADRAFT_116697 [Volvox carteri f.nagariensis] | 1.00E-43 | vcn:VOLCADRAFT_116697 | K13281 |
| TRINITY_DN36144_c0_g1_i1 | 0.0401708 | 4.29025 | XP_002958626.1 | hypothetical protein VOLCADRAFT_99935 [Volvox carteri f. nagariensis] | 1.00E-22 | vcn:VOLCADRAFT_99935 | K10589 |
| TRINITY_DN28478_c0_g4_i1 | 1.73E-06 | 4.36451 | XP_001702590.1 | NADH:ubiquinone oxidoreductase 51 kDa subunit [Chlamydomonasreinhardtii] | 1.00E-150 | cre:CHLREDRAFT_186342 | K03942 |
| TRINITY_DN43828_c3_g1_i6 | 2.77E-07 | 4.37816 | XP_005849966.1 | hypothetical protein CHLNCDRAFT_17375, partial [Chlorellavariabilis] | 3.00E-33 | cvr:CHLNCDRAFT_17375 | K17879 |
| TRINITY_DN17396_c0_g3_i1 | 8.90E-09 | 4.39273 | XP_002954675.1 | hypothetical protein VOLCADRAFT_82885 [Volvox carteri f. nagariensis] | 1.00E-127 | vcn:VOLCADRAFT_82885 | K00654 |
| TRINITY_DN45047_c1_g1_i15 | 0.00702165 | 4.52187 | XP_010266929.1 | PREDICTED: probable DNA helicase MCM9 [Nelumbo nucifera] | 4.00E-93 | nnu:104604326 | K10738 |
| TRINITY_DN43558_c2_g1_i3 | 0.000659195 | 4.84047 | XP_002957319.1 | tRNA pseudouridine synthase, partial [Volvox carteri f. nagariensis] | 6.00E-27 | vcn:VOLCADRAFT_44340 | K06173 |
| TRINITY_DN47582_c0_g13_i1 | 1.45E-06 | 4.86015 | XP_001703188.1 | ribosomal protein L36, component of cytosolic 80S ribosome and 60Slarge subunit [Chlamydomonas reinhardtii] | 4.00E-36 | cre:CHLREDRAFT_195597 | K02920 |
| TRINITY_DN44373_c2_g2_i2 | 0 | 4.91167 | XP_002958724.1 | hypothetical protein VOLCADRAFT_109463 [Volvox carteri f.nagariensis] | 1.00E-117 | vcn:VOLCADRAFT_109463 | K07304 |
| TRINITY_DN57697_c2_g2_i1 | 0.0406125 | 5.03562 | XP_002957643.1 | hypothetical protein VOLCADRAFT_121646 [Volvox carteri f.nagariensis] | 7.00E-18 | vcn:VOLCADRAFT_121646 | K04450 |
| TRINITY_DN1264_c1_g2_i1 | 2.11E-12 | 5.05711 | XP_001691107.1 | phosphatidate cytidylyltransferase [Chlamydomonas reinhardtii] | 8.00E-77 | cre:CHLREDRAFT_188754 | K00981 |
| TRINITY_DN46901_c0_g1_i1 | 0 | 5.06585 | XP_002959097.1 | copper amine oxidase [Volvox carteri f. nagariensis] | 0 | vcn:VOLCADRAFT_84774 | K00276 |
| TRINITY_DN36337_c1_g2_i1 | 0.0116825 | 5.13918 | XP_001691346.1 | epsilon-adaptin [Chlamydomonas reinhardtii] | 2.00E-45 | cre:CHLREDRAFT_195459 | K12400 |
| TRINITY_DN46010_c1_g2_i1 | 0.00180779 | 5.36137 | XP_001697173.1 | 26S proteasome regulatory subunit [Chlamydomonas reinhardtii] | 0 | cre:CHLREDRAFT_192526 | K03032 |
| TRINITY_DN42890_c0_g1_i9 | 0.0147207 | 5.39293 | XP_002949213.1 | phosphofructokinase family protein [Volvox carteri f. nagariensis] | 1.00E-108 | vcn:VOLCADRAFT_74177 | K00850 |
| TRINITY_DN19956_c0_g3_i1 | 2.22E-16 | 5.4902 | XP_002947115.1 | hypothetical protein VOLCADRAFT_73112 [Volvox carteri f.nagariensis] | 2.00E-84 | vcn:VOLCADRAFT_73112 | K04079 |
| TRINITY_DN44373_c2_g2_i3 | 1.74E-09 | 5.51489 | XP_001689879.1 | peptide methionine sulfoxide reductase [Chlamydomonas reinhardtii] | 2.00E-25 | cre:CHLREDRAFT_189675 | K07304 |
| TRINITY_DN45615_c5_g7_i12 | 0.000552587 | 5.59794 | XP_002958779.1 | actin-binding protein gelsolin [Volvox carteri f. nagariensis] | 1.00E-112 | vcn:VOLCADRAFT_108308 | K05768 |
| TRINITY_DN55737_c1_g1_i1 | 0 | 5.66594 | XP_002947115.1 | hypothetical protein VOLCADRAFT_73112 [Volvox carteri f. nagariensis] | 1.00E-130 | vcn:VOLCADRAFT_73112 | K04079 |
| TRINITY_DN32441_c0_g4_i1 | 0.000101286 | 5.74165 | XP_002949800.1 | hypothetical protein VOLCADRAFT_104457 [Volvox carteri f.nagariensis] | 1.00E-136 | vcn:VOLCADRAFT_104457 | K00645 |
| TRINITY_DN28128_c1_g1_i1 | 2.92E-10 | 5.81859 | XP_001697080.1 | chlorophyll b reductase [Chlamydomonas reinhardtii] | 2.00E-97 | cre:CHLREDRAFT_205572 | K13606 |
| TRINITY_DN46833_c0_g2_i3 | 4.28E-06 | 5.86925 | XP_001698054.1 | cryptochrome photoreceptor [Chlamydomonas reinhardtii] | 0 | cre:CHLREDRAFT_206002 | K02295 |
| TRINITY_DN46382_c5_g1_i4 | 0 | 5.9485 | XP_003057423.1 | ATP-dependent DNA helicase [Micromonas pusilla CCMP1545] | 4.00E-17 | mpp:MICPUCDRAFT_56568 | K03657 |
| TRINITY_DN47251_c1_g1_i6 | 0.00102988 | 6.02847 | XP_005646103.1 | hypothetical protein COCSUDRAFT_17595 [Coccomyxa subellipsoideaC-169] | 1.00E-91 | csl:COCSUDRAFT_17595 | K08740 |
| TRINITY_DN34248_c1_g3_i1 | 1.90E-08 | 6.20503 | XP_002950878.1 | hypothetical protein VOLCADRAFT_117692, partial [Volvox carteri f.nagariensis] | 1.00E-45 | vcn:VOLCADRAFT_117692 | K08850 |
| TRINITY_DN22734_c1_g2_i6 | 5.10E-05 | 6.2356 | XP_002948667.1 | hypothetical protein VOLCADRAFT_89014 [Volvox carteri f.nagariensis] | 4.00E-23 | vcn:VOLCADRAFT_89014 | K13071 |
| TRINITY_DN44373_c2_g2_i1 | 1.72E-12 | 6.29581 | XP_002958724.1 | hypothetical protein VOLCADRAFT_109463 [Volvox carteri f.nagariensis] | 3.00E-84 | vcn:VOLCADRAFT_109463 | K07304 |
| TRINITY_DN34248_c1_g1_i1 | 0 | 6.70183 | XP_002950878.1 | hypothetical protein VOLCADRAFT_117692, partial [Volvox carteri f.nagariensis] | 1.00E-115 | vcn:VOLCADRAFT_117692 | K08850 |
| TRINITY_DN43041_c0_g4_i9 | 0 | 6.7657 | XP_005643732.1 | carboxyl transferase [Coccomyxa subellipsoidea C-169] | 3.00E-47 | csl:COCSUDRAFT_38395 | K01969 |
| TRINITY_DN27356_c0_g2_i1 | 8.55E-15 | 6.99773 | XP_001689942.1 | deoxypusine synthase 1, partial [Chlamydomonas reinhardtii] | 1.00E-158 | cre:CHLREDRAFT_128965 | K00809 |
| TRINITY_DN53605_c2_g1_i1 | 0 | 7.20489 | XP_006476504.1 | PREDICTED: heat shock protein 83-like [Citrus sinensis] | 1.00E-73 | cit:102616579 | K04079 |
| TRINITY_DN47865_c0_g2_i14 | 9.03E-07 | 7.40902 | XP_001698641.1 | ser/thr protein kinase, partial [Chlamydomonas reinhardtii] | 1.00E-106 | cre:CHLREDRAFT_113331 | K14498 |
| TRINITY_DN46110_c0_g4_i1 | 0.0379474 | 7.56533 | XP_002948356.1 | component of cytosolic 80S ribosome and 60S large subunit [Volvoxcarteri f. nagariensis] | 1.00E-24 | vcn:VOLCADRAFT_103828 | K02910 |
| TRINITY_DN39075_c0_g2_i2 | 0.00366231 | 7.579 | XP_005852018.1 | hypothetical protein CHLNCDRAFT_132961 [Chlorella variabilis] | 1.00E-25 | cvr:CHLNCDRAFT_132961 | K10744 |
| TRINITY_DN47883_c1_g1_i2 | 0 | 8.18019 | XP_001691461.1 | dynein heavy chain [Chlamydomonas reinhardtii] | 0 | cre:CHLREDRAFT_206178 | K10408 |
| TRINITY_DN47548_c3_g1_i6 | 0 | 8.3558 | XP_005644118.1 | hypothetical protein COCSUDRAFT_58323 [Coccomyxa subellipsoideaC-169] | 1.00E-60 | csl:COCSUDRAFT_58323 | K08741 |
| TRINITY_DN46063_c0_g2_i4 | 0.0120912 | 8.6286 | XP_002954957.1 | hypothetical protein VOLCADRAFT_106613 [Volvox carteri f.nagariensis] | 5.00E-33 | vcn:VOLCADRAFT_106613 | K12351 |
| TRINITY_DN24514_c2_g1_i4 | 0 | 9.14434 | XP_001693396.1 | predicted protein, partial [Chlamydomonas reinhardtii] | 7.00E-42 | cre:CHLREDRAFT_98954 | K03470 |
| TRINITY_DN47251_c1_g1_i4 | 0.0298426 | 9.18338 | XP_005646103.1 | hypothetical protein COCSUDRAFT_17595 [Coccomyxa subellipsoideaC-169] | 1.00E-89 | csl:COCSUDRAFT_17595 | K08740 |
| TRINITY_DN47840_c0_g1_i18 | 0.0383294 | 9.37272 | XP_001702488.1 | chromosome condensation complex protein [Chlamydomonas reinhardtii] | 8.00E-14 | cre:CHLREDRAFT_186204 | K06677 |
| TRINITY_DN42031_c0_g2_i4 | 0 | 9.41763 | XP_002949842.1 | hypothetical protein VOLCADRAFT_59964 [Volvox carteri f. nagariensis] | 1.00E-144 | vcn:VOLCADRAFT_59964 | K00253 |
| TRINITY_DN43041_c0_g4_i8 | 0 | 9.77542 | XP_011398114.1 | Methylcrotonoyl-CoA carboxylase beta chain, mitochondrial[Auxenochlorella protothecoides] | 0 | apro:F751_6178 | K01969 |
| TRINITY_DN42453_c0_g1_i7 | 0.00358966 | 9.99007 | XP_002959249.1 | hypothetical protein VOLCADRAFT_100659 [Volvox carteri f.nagariensis] | 7.00E-21 | vcn:VOLCADRAFT_100659 | K11438 |
| TRINITY_DN46901_c0_g1_i5 | 0 | 10.1561 | XP_002959097.1 | copper amine oxidase [Volvox carteri f. nagariensis] | 2.00E-68 | vcn:VOLCADRAFT_84774 | K00276 |
| TRINITY_DN44741_c0_g1_i4 | 6.86E-08 | 10.7356 | XP_002948501.1 | hypothetical protein VOLCADRAFT_58370, partial [Volvox carteri f.nagariensis] | 1.00E-79 | vcn:VOLCADRAFT_58370 | K01262 |
| TRINITY_DN45881_c0_g1_i6 | 0 | 10.9503 | XP_001700336.1 | zinc-metallopeptidase-like protein [Chlamydomonas reinhardtii] | 7.00E-62 | cre:CHLREDRAFT_20076 | K13539 |
| TRINITY_DN14047_c0_g1_i1 | 3.94E-13 | 11.1363 | XP_001702571.1 | heavy metal transporting ATPase [Chlamydomonas reinhardtii] | 1.00E-132 | cre:CHLREDRAFT_205938 | K01533 |
| TRINITY_DN34007_c0_g3_i2 | 8.31E-06 | 11.4826 | XP_011397910.1 | Protein arginine N-methyltransferase 7 [Auxenochlorellaprotothecoides] | 3.00E-18 | apro:F751_1901 | K11438 |
| TRINITY_DN46991_c3_g1_i3 | 0.0304729 | 12.2086 | XP_002952814.1 | hypothetical protein VOLCADRAFT_63040, partial [Volvox carteri f.nagariensis] | 2.00E-96 | vcn:VOLCADRAFT_63040 | K17871 |
| TRINITY_DN47892_c11_g1_i1 | 0.0436338 | 12.3537 | XP_001702393.1 | flagellar associated protein, partial [Chlamydomonas reinhardtii] | 1.00E-117 | cre:CHLREDRAFT_194683 | K15426 |
| TRINITY_DN9302_c0_g1_i3 | 0.00561243 | 12.7346 | XP_002953441.1 | hypothetical protein VOLCADRAFT_94253 [Volvox carteri f. nagariensis] | 2.00E-41 | vcn:VOLCADRAFT_94253 | K09651 |
| TRINITY_DN39961_c0_g1_i1 | 0.0181501 | 12.8469 | XP_005644539.1 | hypothetical protein COCSUDRAFT_38028 [Coccomyxa subellipsoideaC-169] | 2.00E-88 | csl:COCSUDRAFT_38028 | K15083 |
| TRINITY_DN47251_c2_g10_i3 | 0 | 12.9163 | XP_005851192.1 | hypothetical protein CHLNCDRAFT_137861 [Chlorella variabilis] | 2.00E-35 | cvr:CHLNCDRAFT_137861 | K08740 |
| TRINITY_DN6016_c0_g1_i1 | 0.00794159 | 13.2133 | XP_007511964.1 | actin [Bathycoccus prasinos] | 1.00E-26 | bpg:Bathy07g04710 | K10355 |
| TRINITY_DN21833_c0_g1_i2 | 0.0256861 | 13.2359 | XP_005849206.1 | hypothetical protein CHLNCDRAFT_143925 [Chlorella variabilis] | 1.00E-14 | cvr:CHLNCDRAFT_143925 | K03537 |
| TRINITY_DN55203_c0_g2_i4 | 0.00228487 | 13.6623 | XP_002950124.1 | NADH-cytochrome b5 reductase [Volvox carteri f. nagariensis] | 1.00E-89 | vcn:VOLCADRAFT_74532 | K00326 |
| TRINITY_DN37148_c0_g2_i2 | 1.08E-06 | 14.3726 | XP_004504869.2 | PREDICTED: 18.2 kDa class I heat shock protein-like [Cicerarietinum] | 1.00E-12 | cam:101490951 | K13993 |
| TRINITY_DN40386_c1_g1_i5 | 0.0243991 | 14.7762 | XP_002954794.1 | hypothetical protein VOLCADRAFT_95623 [Volvox carteri f. nagariensis] | 2.00E-56 | vcn:VOLCADRAFT_95623 | K03667 |
| TRINITY_DN47251_c1_g1_i16 | 1.51E-09 | 15.3574 | XP_005646103.1 | hypothetical protein COCSUDRAFT_17595 [Coccomyxa subellipsoideaC-169] | 1.00E-89 | csl:COCSUDRAFT_17595 | K08740 |
| TRINITY_DN46901_c0_g1_i3 | 5.25E-05 | 16.325 | XP_002959097.1 | copper amine oxidase [Volvox carteri f. nagariensis] | 0 | vcn:VOLCADRAFT_84774 | K00276 |
| TRINITY_DN29774_c0_g1_i2 | 0.032796 | 16.9112 | XP_001698334.1 | molybdenum cofactor biosynthesis protein [Chlamydomonas reinhardtii] | 1.00E-107 | cre:CHLREDRAFT_113296 | K15376 |
| TRINITY_DN42453_c0_g1_i5 | 0.00165268 | 16.9363 | XP_001418581.1 | predicted protein [Ostreococcus lucimarinus CCE9901] | 3.00E-14 | olu:OSTLU_15961 | K11438 |
| TRINITY_DN45333_c0_g5_i5 | 0.00294466 | 17.0472 | XP_005652259.1 | S-adenosyl-L-methionine-dependent methyltransferase [Coccomyxasubellipsoidea C-169] | 7.00E-35 | csl:COCSUDRAFT_11603 | K11438 |
| TRINITY_DN46879_c0_g1_i14 | 6.70E-05 | 17.3217 | XP_001703433.1 | hypothetical protein CHLREDRAFT_188156 [Chlamydomonas reinhardtii] | 3.00E-50 | cre:CHLREDRAFT_188156 | K17263 |
| TRINITY_DN45333_c0_g5_i1 | 0.0029196 | 17.7106 | XP_005652259.1 | S-adenosyl-L-methionine-dependent methyltransferase [Coccomyxasubellipsoidea C-169] | 1.00E-17 | csl:COCSUDRAFT_11603 | K11438 |
| TRINITY_DN39388_c4_g4_i2 | 0 | 17.7918 | XP_002955511.1 | hypothetical protein VOLCADRAFT_83295 [Volvox carteri f. nagariensis] | 2.00E-83 | vcn:VOLCADRAFT_83295 | K15103 |
| TRINITY_DN46901_c0_g1_i2 | 0 | 17.893 | XP_002959097.1 | copper amine oxidase [Volvox carteri f. nagariensis] | 1.00E-158 | vcn:VOLCADRAFT_84774 | K00276 |
| TRINITY_DN52778_c5734_g1_i2 | 1.71E-12 | 18.1411 | XP_001759099.1 | predicted protein [Physcomitrella patens] | 3.00E-36 | ppp:PHYPADRAFT_121199 | K07375 |
| TRINITY_DN45267_c2_g4_i1 | 3.63E-14 | 18.4372 | XP_005647811.1 | Metallophos-domain-containing protein [Coccomyxa subellipsoideaC-169] | 1.00E-79 | csl:COCSUDRAFT_15677 | K18328 |
| TRINITY_DN45852_c0_g1_i3 | 3.77E-15 | 18.4702 | XP_001689583.1 | predicted protein [Chlamydomonas reinhardtii] | 8.00E-52 | cre:CHLREDRAFT_171763 | K11858 |
| TRINITY_DN46499_c7_g3_i8 | 4.81E-06 | 18.481 | XP_005848992.1 | hypothetical protein CHLNCDRAFT_144556 [Chlorella variabilis] | 1.00E-81 | cvr:CHLNCDRAFT_144556 | K08741 |
| TRINITY_DN47234_c0_g1_i3 | 3.72E-08 | 19.0304 | XP_002954657.1 | hypothetical protein VOLCADRAFT_82855 [Volvox carteri f. nagariensis] | 2.00E-14 | vcn:VOLCADRAFT_82855 | K19090 |
| TRINITY_DN26025_c0_g2_i3 | 1.63E-08 | 19.6447 | XP_002949252.1 | hypothetical protein VOLCADRAFT_104190 [Volvox carteri f.nagariensis] | 1.00E-22 | vcn:VOLCADRAFT_104190 | K11877 |
| TRINITY_DN47735_c8_g1_i6 | 0 | 20.1945 | XP_001690583.1 | DEAD/DEAH box helicase-related protein [Chlamydomonas reinhardtii] | 3.00E-13 | cre:CHLREDRAFT_169389 | K13179 |
| TRINITY_DN34248_c1_g4_i2 | 0 | 20.3153 | XP_002950878.1 | hypothetical protein VOLCADRAFT_117692, partial [Volvox carteri f.nagariensis] | 7.00E-47 | vcn:VOLCADRAFT_117692 | K08850 |
| TRINITY_DN46318_c3_g1_i9 | 0.0420051 | 20.3224 | XP_002959026.1 | hypothetical protein VOLCADRAFT_108435 [Volvox carteri f.nagariensis] | 1.00E-57 | vcn:VOLCADRAFT_108435 | K02349 |
| TRINITY_DN46104_c0_g2_i8 | 0.00194703 | 20.3308 | XP_005851493.1 | hypothetical protein CHLNCDRAFT_137877 [Chlorella variabilis] | 4.00E-54 | cvr:CHLNCDRAFT_137877 | K15075 |
| TRINITY_DN45881_c0_g1_i4 | 0 | 21.3294 | XP_001700336.1 | zinc-metallopeptidase-like protein [Chlamydomonas reinhardtii] | 3.00E-74 | cre:CHLREDRAFT_20076 | K13539 |
| TRINITY_DN8768_c0_g2_i2 | 1.10E-09 | 21.5767 | XP_005649320.1 | hypothetical protein COCSUDRAFT_53066 [Coccomyxa subellipsoideaC-169] | 1.00E-126 | csl:COCSUDRAFT_53066 | K01610 |
| TRINITY_DN37349_c2_g3_i4 | 0.0116406 | 21.9076 | XP_001693499.1 | predicted protein [Chlamydomonas reinhardtii] | 4.00E-35 | cre:CHLREDRAFT_182706 | K14401 |
| TRINITY_DN46901_c0_g1_i4 | 1.85E-07 | 22.4279 | XP_002959097.1 | copper amine oxidase [Volvox carteri f. nagariensis] | 1.00E-158 | vcn:VOLCADRAFT_84774 | K00276 |
| TRINITY_DN45279_c0_g2_i2 | 2.39E-07 | 22.4399 | XP_002946905.1 | hypothetical protein VOLCADRAFT_72905 [Volvox carteri f.nagariensis] | 3.00E-47 | vcn:VOLCADRAFT_72905 | K02638 |
| TRINITY_DN42890_c0_g1_i11 | 7.56E-13 | 22.4918 | XP_002949213.1 | phosphofructokinase family protein [Volvox carteri f. nagariensis] | 1.00E-131 | vcn:VOLCADRAFT_74177 | K00850 |
| TRINITY_DN47251_c1_g1_i20 | 0.000796035 | 23.6764 | XP_005646103.1 | hypothetical protein COCSUDRAFT_17595 [Coccomyxa subellipsoideaC-169] | 1.00E-89 | csl:COCSUDRAFT_17595 | K08740 |
| TRINITY_DN42453_c0_g1_i12 | 0 | 24.184 | XP_002959249.1 | hypothetical protein VOLCADRAFT_100659 [Volvox carteri f.nagariensis] | 5.00E-27 | vcn:VOLCADRAFT_100659 | K11438 |
| TRINITY_DN14419_c0_g1_i1 | 0.0493678 | 24.7193 | XP_001693396.1 | predicted protein, partial [Chlamydomonas reinhardtii] | 3.00E-19 | cre:CHLREDRAFT_98954 | K03470 |
| TRINITY_DN45333_c0_g5_i3 | 0 | 25.8715 | XP_003080109.1 | Arginine N-methyltransferase (ISS) [Ostreococcus tauri] | 5.00E-26 | ota:Ot07g00030 | K11438 |
| TRINITY_DN34248_c1_g4_i1 | 0 | 28.2115 | XP_002950878.1 | hypothetical protein VOLCADRAFT_117692, partial [Volvox carteri f.nagariensis] | 8.00E-60 | vcn:VOLCADRAFT_117692 | K08850 |
| TRINITY_DN57916_c0_g3_i1 | 0.00446373 | 30.8629 | XP_005645586.1 | hypothetical protein COCSUDRAFT_30231 [Coccomyxa subellipsoideaC-169] | 1.00E-138 | csl:COCSUDRAFT_30231 | K06901 |
| TRINITY_DN46391_c0_g1_i2 | 0 | 31.9884 | XP_005850059.1 | hypothetical protein CHLNCDRAFT_50614 [Chlorella variabilis] | 9.00E-17 | cvr:CHLNCDRAFT_50614 | K07434 |
| TRINITY_DN47434_c0_g2_i9 | 0.00229802 | 32.1483 | XP_002945657.1 | microtubule-associated protein MAP65 [Volvox carteri f. nagariensis] | 2.00E-67 | vcn:VOLCADRAFT_120144 | K16732 |
| TRINITY_DN41812_c1_g1_i7 | 0.00139123 | 32.2525 | XP_002947919.1 | hypothetical protein VOLCADRAFT_57730 [Volvox carteri f.nagariensis] | 9.00E-34 | vcn:VOLCADRAFT_57730 | K03842 |
| TRINITY_DN42857_c0_g1_i6 | 0.000114429 | 32.7697 | XP_002955556.1 | hypothetical protein VOLCADRAFT_96440 [Volvox carteri f.nagariensis] | 7.00E-39 | vcn:VOLCADRAFT_96440 | K13206 |
| TRINITY_DN47399_c6_g5_i8 | 7.20E-05 | 34.3987 | XP_001689583.1 | predicted protein [Chlamydomonas reinhardtii] | 3.00E-50 | cre:CHLREDRAFT_171763 | K11858 |
| TRINITY_DN9069_c0_g2_i1 | 0 | 34.8739 | XP_002958467.1 | superoxide dismutase [Mn] [Volvox carteri f. nagariensis] | 3.00E-47 | vcn:VOLCADRAFT_108150 | K04564 |
| TRINITY_DN47251_c1_g1_i15 | 2.28E-07 | 35.6364 | XP_005646103.1 | hypothetical protein COCSUDRAFT_17595 [Coccomyxa subellipsoideaC-169] | 2.00E-91 | csl:COCSUDRAFT_17595 | K08740 |
| TRINITY_DN28709_c1_g1_i1 | 0.000969182 | 35.7559 | XP_005649988.1 | WD40 repeat-like protein, partial [Coccomyxa subellipsoidea C-169] | 4.00E-64 | csl:COCSUDRAFT_3880 | K17908 |
| TRINITY_DN47251_c1_g1_i1 | 0 | 37.4837 | XP_005646103.1 | hypothetical protein COCSUDRAFT_17595 [Coccomyxa subellipsoideaC-169] | 2.00E-91 | csl:COCSUDRAFT_17595 | K08740 |
| TRINITY_DN44264_c6_g1_i3 | 0.00875775 | 38.6505 | XP_001689423.1 | 26S proteasome regulatory subunit [Chlamydomonas reinhardtii] | 1.00E-137 | cre:CHLREDRAFT_24132 | K03030 |
| TRINITY_DN22996_c2_g1_i2 | 0.00260691 | 39.1424 | XP_002957409.1 | hypothetical protein VOLCADRAFT_107666 [Volvox carteri f.nagariensis] | 5.00E-68 | vcn:VOLCADRAFT_107666 | K13217 |
| TRINITY_DN45047_c1_g1_i31 | 0.0111187 | 40.2374 | XP_002988885.1 | hypothetical protein SELMODRAFT_427521 [Selaginella moellendorffii] | 2.00E-80 | smo:SELMODRAFT_427521 | K10738 |
| TRINITY_DN50544_c1_g1_i1 | 0.020233 | 40.4146 | XP_001695734.1 | CDF transporter, membrane protein [Chlamydomonas reinhardtii] | 6.00E-47 | cre:CHLREDRAFT_205987 | K14689 |
| TRINITY_DN45177_c13_g1_i1 | 0.00032279 | 40.9366 | XP_001695466.1 | chlorophyll a-b binding protein of LHCII [Chlamydomonasreinhardtii] | 4.00E-29 | cre:CHLREDRAFT_184479 | K08912 |
| TRINITY_DN47857_c0_g4_i7 | 0.0105082 | 41.1083 | XP_002958611.1 | light-harvesting protein of photosystem I [Volvox carteri f.nagariensis] | 2.00E-62 | vcn:VOLCADRAFT_77985 | K08907 |
| TRINITY_DN47399_c6_g5_i18 | 0.000423626 | 41.8737 | XP_001689583.1 | predicted protein [Chlamydomonas reinhardtii] | 4.00E-35 | cre:CHLREDRAFT_171763 | K11858 |
| TRINITY_DN47735_c8_g1_i18 | 0.000625121 | 42.1042 | XP_001690583.1 | DEAD/DEAH box helicase-related protein [Chlamydomonas reinhardtii] | 1.00E-11 | cre:CHLREDRAFT_169389 | K13179 |
| TRINITY_DN46790_c0_g1_i23 | 0.000625703 | 42.3796 | XP_002947904.1 | NimA-related protein kinase 6 [Volvox carteri f. nagariensis] | 1.00E-142 | vcn:VOLCADRAFT_73509 | K08857 |
| TRINITY_DN3615_c2_g1_i1 | 0.00268124 | 44.3954 | XP_002951295.1 | component of cytosolic 80S ribosome and 40S small subunit [Volvoxcarteri f. nagariensis] | 1.00E-44 | vcn:VOLCADRAFT_120952 | K02958 |
| TRINITY_DN47517_c0_g2_i4 | 0.00039334 | 44.8917 | XP_002954979.1 | dynein heavy chain 9 [Volvox carteri f. nagariensis] | 0 | vcn:VOLCADRAFT_65425 | K10408 |
| TRINITY_DN46605_c0_g3_i4 | 3.80E-06 | 44.9934 | XP_010519297.1 | PREDICTED: polyadenylate-binding protein 8-like [Tarenayahassleriana] | 9.00E-20 | thj:104798793 | K13126 |
| TRINITY_DN23058_c6_g1_i2 | 3.41E-06 | 46.0386 | XP_005843025.1 | hypothetical protein CHLNCDRAFT_59390 [Chlorella variabilis] | 1.00E-76 | cvr:CHLNCDRAFT_59390 | K06569 |
| TRINITY_DN45865_c15_g5_i1 | 0.000190086 | 46.5634 | XP_001697913.1 | hypothetical protein CHLREDRAFT_120661, partial [Chlamydomonasreinhardtii] | 1.00E-169 | cre:CHLREDRAFT_120661 | K03977 |
| TRINITY_DN44646_c5_g2_i2 | 5.92E-06 | 46.7185 | XP_005645413.1 | methionyl aminopeptidase-like protein, partial [Coccomyxasubellipsoidea C-169] | 1.00E-22 | csl:COCSUDRAFT_18342 | K01265 |
| TRINITY_DN45082_c0_g3_i2 | 0.00247055 | 47.112 | XP_002952352.1 | dynein heavy chain 5 [Volvox carteri f. nagariensis] | 0 | vcn:VOLCADRAFT_62471 | K10408 |
| TRINITY_DN47616_c0_g2_i12 | 2.68E-05 | 47.3351 | XP_002976541.1 | hypothetical protein SELMODRAFT_105336 [Selaginella moellendorffii] | 2.00E-47 | smo:SELMODRAFT_105336 | K20093 |
| TRINITY_DN45333_c0_g4_i2 | 0.024663 | 47.8644 | XP_005649397.1 | 4-aminobutyrate aminotransferase [Coccomyxa subellipsoidea C-169] | 1.00E-108 | csl:COCSUDRAFT_53099 | K00823 |
| TRINITY_DN60178_c1_g4_i1 | 0.000916367 | 47.9648 | XP_001700902.1 | magnesium chelatase subunit D [Chlamydomonas reinhardtii] | 1.00E-124 | cre:CHLREDRAFT_134594 | K03404 |
| TRINITY_DN38769_c0_g2_i2 | 1.51E-07 | 48.5609 | XP_001700207.1 | cysteinyl-tRNA synthetase, partial [Chlamydomonas reinhardtii] | 1.00E-160 | cre:CHLREDRAFT_153062 | K01883 |
| TRINITY_DN5954_c0_g2_i1 | 7.36E-06 | 48.8211 | XP_001702744.1 | predicted protein [Chlamydomonas reinhardtii] | 7.00E-26 | cre:CHLREDRAFT_186321 | K03123 |
| TRINITY_DN45333_c0_g4_i5 | 8.83E-06 | 49.352 | XP_005649397.1 | 4-aminobutyrate aminotransferase [Coccomyxa subellipsoidea C-169] | 1.00E-108 | csl:COCSUDRAFT_53099 | K00823 |
| TRINITY_DN45273_c1_g3_i1 | 4.47E-06 | 50.666 | XP_001689967.1 | NADP malic enzyme [Chlamydomonas reinhardtii] | 7.00E-77 | cre:CHLREDRAFT_196351 | K00029 |
| TRINITY_DN11590_c1_g1_i1 | 9.81E-07 | 50.7996 | XP_002949928.1 | hypothetical protein VOLCADRAFT_80842 [Volvox carteri f.nagariensis] | 8.00E-65 | vcn:VOLCADRAFT_80842 | K00670 |
| TRINITY_DN33703_c1_g1_i2 | 2.80E-06 | 51.409 | XP_002948498.1 | hypothetical protein VOLCADRAFT_88920 [Volvox carteri f. nagariensis] | 5.00E-30 | vcn:VOLCADRAFT_88920 | K10862 |
| TRINITY_DN47962_c0_g1_i6 | 0.0116755 | 53.195 | XP_002953889.1 | hypothetical protein VOLCADRAFT_118510 [Volvox carteri f.nagariensis] | 3.00E-52 | vcn:VOLCADRAFT_118510 | K17592 |
| TRINITY_DN22493_c2_g5_i1 | 8.68E-08 | 54.2577 | XP_001697667.1 | prefoldin-related KE2-like protein [Chlamydomonas reinhardtii] | 9.00E-12 | cre:CHLREDRAFT_151087 | K09548 |
| TRINITY_DN30615_c0_g1_i1 | 1.15E-09 | 54.4027 | XP_001690530.1 | hypothetical protein CHLREDRAFT_182890 [Chlamydomonas reinhardtii] | 8.00E-51 | cre:CHLREDRAFT_182890 | K19362 |
| TRINITY_DN36849_c0_g2_i2 | 1.71E-10 | 54.5044 | XP_011396503.1 | Chromatin assembly factor 1 subunit B [Auxenochlorellaprotothecoides] | 1.00E-66 | apro:F751_2888 | K10751 |
| TRINITY_DN9359_c0_g1_i1 | 1.54E-10 | 55.21 | XP_001692993.1 | glycine cleavage system, P protein [Chlamydomonas reinhardtii] | 1.00E-55 | cre:CHLREDRAFT_136984 | K00281 |
| TRINITY_DN33491_c2_g2_i1 | 7.31E-11 | 55.5307 | XP_005847220.1 | hypothetical protein CHLNCDRAFT_31176, partial [Chlorella variabilis] | 1.00E-133 | cvr:CHLNCDRAFT_31176 | K17680 |
| TRINITY_DN55579_c0_g4_i1 | 9.23E-09 | 56.3768 | XP_001698141.1 | mitochondrial ribosomal protein L33 [Chlamydomonas reinhardtii] | 2.00E-16 | cre:CHLREDRAFT_151596 | K02913 |
| TRINITY_DN21882_c0_g4_i1 | 2.13E-11 | 58.4548 | XP_001694933.1 | predicted protein [Chlamydomonas reinhardtii] | 1.00E-148 | cre:CHLREDRAFT_137578 | K01889 |
| TRINITY_DN45878_c1_g2_i5 | 0 | 58.6402 | XP_002958086.1 | DEAH-box nuclear pre-mRNA splicing factor [Volvox carteri f.nagariensis] | 4.00E-80 | vcn:VOLCADRAFT_84321 | K12818 |
| TRINITY_DN45333_c0_g4_i32 | 2.11E-10 | 58.9925 | XP_005649397.1 | 4-aminobutyrate aminotransferase [Coccomyxa subellipsoidea C-169] | 3.00E-75 | csl:COCSUDRAFT_53099 | K00823 |
| TRINITY_DN47865_c0_g2_i25 | 1.82E-07 | 60.6579 | XP_001698641.1 | ser/thr protein kinase, partial [Chlamydomonas reinhardtii] | 1.00E-106 | cre:CHLREDRAFT_113331 | K14498 |
| TRINITY_DN39388_c4_g4_i1 | 0 | 61.4628 | XP_002955511.1 | hypothetical protein VOLCADRAFT_83295 [Volvox carteri f. nagariensis] | 1.00E-83 | vcn:VOLCADRAFT_83295 | K15103 |
| TRINITY_DN45333_c0_g4_i16 | 1.81E-12 | 62.4879 | XP_005649397.1 | 4-aminobutyrate aminotransferase [Coccomyxa subellipsoidea C-169] | 1.00E-116 | csl:COCSUDRAFT_53099 | K00823 |
| TRINITY_DN45333_c0_g4_i24 | 1.36E-07 | 63.2313 | XP_005649397.1 | 4-aminobutyrate aminotransferase [Coccomyxa subellipsoidea C-169] | 1.00E-125 | csl:COCSUDRAFT_53099 | K00823 |
| TRINITY_DN22418_c0_g2_i7 | 4.15E-13 | 65.2433 | XP_002953943.1 | hypothetical protein VOLCADRAFT_121243 [Volvox carteri f.nagariensis] | 5.00E-19 | vcn:VOLCADRAFT_121243 | K12418 |
| TRINITY_DN5921_c0_g2_i1 | 1.78E-15 | 66.6081 | XP_001699781.1 | eukaryotic initiation factor [Chlamydomonas reinhardtii] | 1.00E-51 | cre:CHLREDRAFT_141865 | K03236 |
| TRINITY_DN45782_c0_g1_i5 | 2.11E-15 | 69.7202 | XP_002947451.1 | glutathione peroxidase, selenoprotein [Volvox carteri f.nagariensis] | 4.00E-59 | vcn:VOLCADRAFT_127291 | K00432 |
| TRINITY_DN45333_c0_g4_i1 | 3.86E-10 | 69.9313 | XP_005649397.1 | 4-aminobutyrate aminotransferase [Coccomyxa subellipsoidea C-169] | 3.00E-75 | csl:COCSUDRAFT_53099 | K00823 |
| TRINITY_DN58087_c2_g5_i1 | 5.46E-14 | 70.7644 | XP_002952802.1 | hypothetical protein VOLCADRAFT_82002 [Volvox carteri f. nagariensis] | 1.00E-116 | vcn:VOLCADRAFT_82002 | K03650 |
| TRINITY_DN3227_c0_g1_i1 | 4.00E-15 | 73.5538 | XP_001690340.1 | half-size ABC transporter, membrane protein, partial [Chlamydomonasreinhardtii] | 0 | cre:CHLREDRAFT_99736 | K05661 |
| TRINITY_DN52978_c1_g2_i1 | 2.22E-16 | 74.0819 | XP_002951851.1 | hypothetical protein VOLCADRAFT_92444 [Volvox carteri f. nagariensis] | 3.00E-17 | vcn:VOLCADRAFT_92444 | K14798 |
| TRINITY_DN19077_c0_g8_i1 | 3.69E-10 | 74.6542 | XP_005849895.1 | hypothetical protein CHLNCDRAFT_34758 [Chlorella variabilis] | 6.00E-70 | cvr:CHLNCDRAFT_34758 | K15731 |
| TRINITY_DN47735_c8_g1_i10 | 0 | 74.7122 | XP_001690583.1 | DEAD/DEAH box helicase-related protein [Chlamydomonas reinhardtii] | 4.00E-13 | cre:CHLREDRAFT_169389 | K13179 |
| TRINITY_DN46702_c0_g3_i5 | 3.26E-13 | 77.4441 | XP_011396762.1 | Fumarate hydratase 2, chloroplastic [Auxenochlorella protothecoides] | 1.00E-158 | apro:F751_6852 | K01679 |
| TRINITY_DN30053_c4_g2_i1 | 0 | 78.0726 | XP_005845513.1 | hypothetical protein CHLNCDRAFT_58502 [Chlorella variabilis] | 7.00E-27 | cvr:CHLNCDRAFT_58502 | K11291 |
| TRINITY_DN22418_c0_g2_i1 | 0 | 80.2031 | XP_001690117.1 | fatty acid desaturase, partial [Chlamydomonas reinhardtii] | 4.00E-18 | cre:CHLREDRAFT_32523 | K12418 |
| TRINITY_DN47234_c0_g1_i8 | 0 | 81.4238 | XP_001702469.1 | flagellar central pair-associated protein [Chlamydomonasreinhardtii] | 1.00E-17 | cre:CHLREDRAFT_181848 | K19090 |
| TRINITY_DN58646_c0_g1_i3 | 3.33E-16 | 83.0163 | XP_002947426.1 | type XI myosin heavy chain MyoB [Volvox carteri f. nagariensis] | 1.00E-33 | vcn:VOLCADRAFT_57247 | K10357 |
| TRINITY_DN47759_c0_g2_i1 | 0 | 89.9705 | XP_005645275.1 | chloroplast fructose-1,6-bisphosphatase II [Coccomyxasubellipsoidea C-169] | 1.00E-24 | csl:COCSUDRAFT_57295 | K03841 |
| TRINITY_DN29878_c0_g1_i3 | 0 | 91.7101 | XP_011401391.1 | putative helicase [Auxenochlorella protothecoides] | 1.00E-113 | apro:F751_5289 | K10706 |
| TRINITY_DN17200_c0_g2_i2 | 4.44E-16 | 93.8713 | XP_002947426.1 | type XI myosin heavy chain MyoB [Volvox carteri f. nagariensis] | 1.00E-88 | vcn:VOLCADRAFT_57247 | K10357 |
| TRINITY_DN36651_c1_g1_i4 | 0 | 97.1615 | XP_001701378.1 | 3-phosphoinoside dependent protein kinase [Chlamydomonas reinhardtii] | 1.00E-105 | cre:CHLREDRAFT_122463 | K06276 |
| TRINITY_DN22996_c2_g1_i4 | 0 | 98.8371 | XP_002957409.1 | hypothetical protein VOLCADRAFT_107666 [Volvox carteri f.nagariensis] | 4.00E-68 | vcn:VOLCADRAFT_107666 | K13217 |
| TRINITY_DN47233_c0_g3_i3 | 0 | 100.247 | XP_001703170.1 | flagellar inner arm dynein 1 heavy chain alpha [Chlamydomonasreinhardtii] | 0 | cre:CHLREDRAFT_60432 | K10408 |
| TRINITY_DN45805_c1_g1_i3 | 0 | 101.901 | XP_011398446.1 | putative ATP-dependent RNA helicase [Auxenochlorella protothecoides] | 6.00E-95 | apro:F751_0577 | K14780 |
| TRINITY_DN45333_c0_g4_i17 | 0 | 107.628 | XP_005649397.1 | 4-aminobutyrate aminotransferase [Coccomyxa subellipsoidea C-169] | 1.00E-125 | csl:COCSUDRAFT_53099 | K00823 |
| TRINITY_DN42453_c0_g1_i9 | 0 | 108.149 | XP_001418581.1 | predicted protein [Ostreococcus lucimarinus CCE9901] | 4.00E-14 | olu:OSTLU_15961 | K11438 |
| TRINITY_DN45333_c0_g4_i18 | 0 | 108.393 | XP_005649397.1 | 4-aminobutyrate aminotransferase [Coccomyxa subellipsoidea C-169] | 1.00E-120 | csl:COCSUDRAFT_53099 | K00823 |
| TRINITY_DN45333_c0_g4_i28 | 0 | 109.374 | XP_005649397.1 | 4-aminobutyrate aminotransferase [Coccomyxa subellipsoidea C-169] | 2.00E-84 | csl:COCSUDRAFT_53099 | K00823 |
| TRINITY_DN47616_c0_g2_i16 | 0 | 111.609 | XP_002976541.1 | hypothetical protein SELMODRAFT_105336 [Selaginella moellendorffii] | 3.00E-47 | smo:SELMODRAFT_105336 | K20093 |
| TRINITY_DN41227_c3_g1_i7 | 0 | 112.371 | XP_001694888.1 | predicted protein [Chlamydomonas reinhardtii] | 1.00E-70 | cre:CHLREDRAFT_118204 | K00626 |
| TRINITY_DN47822_c6_g1_i1 | 0 | 113.552 | XP_002945611.1 | dynein heavy chain 7 [Volvox carteri f. nagariensis] | 0 | vcn:VOLCADRAFT_78597 | K10408 |
| TRINITY_DN47779_c14_g1_i1 | 0 | 117.681 | XP_002951809.1 | hypothetical protein VOLCADRAFT_81595 [Volvox carteri f. nagariensis] | 0 | vcn:VOLCADRAFT_81595 | K14537 |
| TRINITY_DN45333_c0_g4_i4 | 0 | 119.675 | XP_005649397.1 | 4-aminobutyrate aminotransferase [Coccomyxa subellipsoidea C-169] | 1.00E-120 | csl:COCSUDRAFT_53099 | K00823 |
| TRINITY_DN41734_c3_g5_i1 | 0 | 123.775 | XP_002958730.1 | hypothetical protein VOLCADRAFT_84613 [Volvox carteri f.nagariensis] | 4.00E-43 | vcn:VOLCADRAFT_84613 | K11341 |
| TRINITY_DN42453_c0_g1_i10 | 0 | 125.754 | XP_011402211.1 | Protein arginine N-methyltransferase 7 [Auxenochlorellaprotothecoides] | 2.00E-23 | apro:F751_5514 | K11438 |
| TRINITY_DN52778_c5970_g2_i1 | 0 | 128.27 | XP_001702342.1 | ribosomal protein S7, component of cytosolic 80S ribosome and 40Ssmall subunit [Chlamydomonas reinhardtii] | 8.00E-18 | cre:CHLREDRAFT_179706 | K02993 |
| TRINITY_DN19762_c2_g3_i2 | 0 | 130.268 | XP_005646486.1 | kinase-like protein, partial [Coccomyxa subellipsoidea C-169] | 1.00E-130 | csl:COCSUDRAFT_17062 | K08287 |
| TRINITY_DN38293_c0_g1_i8 | 7.47E-07 | 138.713 | XP_005850038.1 | hypothetical protein CHLNCDRAFT_142029 [Chlorella variabilis] | 1.00E-137 | cvr:CHLNCDRAFT_142029 | K00451 |
| TRINITY_DN45865_c15_g2_i1 | 0 | 143.324 | XP_001697913.1 | hypothetical protein CHLREDRAFT_120661, partial [Chlamydomonasreinhardtii] | 1.00E-169 | cre:CHLREDRAFT_120661 | K03977 |
| TRINITY_DN43168_c0_g3_i2 | 0 | 161.188 | XP_005848554.1 | hypothetical protein CHLNCDRAFT_35207 [Chlorella variabilis] | 1.00E-112 | cvr:CHLNCDRAFT_35207 | K14416 |
| TRINITY_DN10358_c0_g2_i1 | 0 | 162.784 | XP_001697130.1 | mitochondrial processing peptidase alpha subunit [Chlamydomonasreinhardtii] | 1.00E-142 | cre:CHLREDRAFT_206036 | K01412 |
| TRINITY_DN16722_c3_g1_i1 | 0 | 164.484 | XP_001695353.1 | chloropyll a-b binding protein of LHCII type I, chloroplastprecursor [Chlamydomonas reinhardtii] | 3.00E-19 | cre:CHLREDRAFT_184490 | K08912 |
| TRINITY_DN48643_c0_g1_i3 | 0 | 165.658 | XP_005850625.1 | hypothetical protein CHLNCDRAFT_19567 [Chlorella variabilis] | 2.00E-89 | cvr:CHLNCDRAFT_19567 | K17675 |
| TRINITY_DN28478_c0_g1_i1 | 0 | 184.523 | XP_002514955.1 | PREDICTED: NADH dehydrogenase [ubiquinone] flavoprotein 1,mitochondrial [Ricinus communis] | 1.00E-150 | rcu:RCOM_1080980 | K03942 |
| TRINITY_DN43469_c0_g2_i3 | 0 | 188.614 | XP_002958391.1 | hypothetical protein VOLCADRAFT_69374 [Volvox carteri f. nagariensis] | 2.00E-87 | vcn:VOLCADRAFT_69374 | K19787 |
| TRINITY_DN47367_c0_g1_i1 | 0 | 234.983 | XP_005850059.1 | hypothetical protein CHLNCDRAFT_50614 [Chlorella variabilis] | 3.00E-43 | cvr:CHLNCDRAFT_50614 | K07434 |
| TRINITY_DN8748_c0_g2_i1 | 0 | 255.038 | XP_001691372.1 | beta-amylase [Chlamydomonas reinhardtii] | 0 | cre:CHLREDRAFT_183141 | K01177 |
| TRINITY_DN1264_c1_g1_i1 | 0 | 285 | XP_001691107.1 | phosphatidate cytidylyltransferase [Chlamydomonas reinhardtii] | 8.00E-77 | cre:CHLREDRAFT_188754 | K00981 |
| TRINITY_DN43134_c4_g2_i1 | 0 | 373.702 | XP_001701487.1 | sugar phosphate/phosphate translocator-like protein [Chlamydomonasreinhardtii] | 1.00E-124 | cre:CHLREDRAFT_153782 | K15283 |
| TRINITY_DN47826_c0_g1_i6 | 9.44E-07 | 401.764 | XP_001689997.1 | hydin-like protein, partial [Chlamydomonas reinhardtii] | 0 | cre:CHLREDRAFT_116240 | K17570 |
| TRINITY_DN8842_c1_g1_i2 | 0 | 588.315 | XP_002951010.1 | 26S proteasome regulatory complex [Volvox carteri f. nagariensis] | 0 | vcn:VOLCADRAFT_104963 | K03066 |
| TRINITY_DN33094_c0_g1_i2 | 0 | 792.36 | XP_001691291.1 | ZIP family transporter [Chlamydomonas reinhardtii] | 2.00E-69 | cre:CHLREDRAFT_183171 | K14709 |
| TRINITY_DN48035_c0_g2_i1 | 0 | 814.619 | XP_001694585.1 | multicopper ferroxidase [Chlamydomonas reinhardtii] | 0 | cre:CHLREDRAFT_184156 | K14735 |
| TRINITY_DN39278_c0_g1_i1 | 0 | 827.77 | XP_003064258.1 | kinesin-II motor protein, flagellar associated [Micromonas pusillaCCMP1545] | 2.00E-29 | mpp:MICPUCDRAFT_36912 | K10394 |
| TRINITY_DN45628_c0_g1_i9 | 0 | 880.258 | XP_001698065.1 | receptor of activated protein kinase C 1 [Chlamydomonasreinhardtii] | 2.00E-70 | cre:CHLREDRAFT_105734 | K14753 |
| TRINITY_DN47010_c0_g1_i5 | 0 | 11163.3 | XP_002953500.1 | component of cytosolic 80S ribosome and 60S large subunit [Volvoxcarteri f. nagariensis] | 4.00E-60 | vcn:VOLCADRAFT_82216 | K02870 |
| TRINITY_DN46460_c1_g2_i1 | 0 | 0.000981959 | XP_002956915.1 | hypothetical protein VOLCADRAFT_30174, partial [Volvox carteri f.nagariensis] | 0 | vcn:VOLCADRAFT_30174 | K12828 |

Supplementary Data Set 2 Specific metabolite list.

(a) Profiling the amine- and phenol-containing metabolites.

|  | **Input mass** | **Input rt** | **Calibrated RT** | **HMDB No.** | **Name** | **Monoisotopic molecular mass** | **mz_light** | **RT** | **Mass error** | **RT error** | **Fold Change (mutated/wildtype)** | **p-value** |
| --- | --- | --- | --- | --- | --- | --- | --- | --- | --- | --- | --- | --- |
| 1 | 517.1493 | 2.45 | 2.32 | HMDB00133 | Guanosine | 283.0917 | 517.15 | 2.22 | 0.0007 | 0.1 | 0.41 | 5.74E-08 |
| 2 | 293.1053 | 3.14 | 3.04 | HMDB01842 | Guanidine | 59.0483 | 293.1067 | 3 | 0.0014 | 0.04 | 0.28 | 2.32E-05 |
| 3 | 399.1055 | 4 | 3.94 | HMDB02005 | Methionine Sulfoxide | 165.046 | 399.1043 | 3.72 | 0.0012 | 0.22 | 0.58 | 8.06E-07 |
| 4 | 399.1041 | 4.33 | 4.3 | HMDB02005 | Methionine Sulfoxide - Isomer | 165.046 | 399.1043 | 4.2 | 0.0002 | 0.1 | 0.57 | 3.29E-07 |
| 5 | 364.1684 | 6.57 | 7.09 | HMDB02064 | N-Acetylputrescine | 130.1106 | 364.1689 | 7.25 | 0.0005 | 0.16 | 0.62 | 4.59E-05 |
| 6 | 460.1168 | 7.79 | 8.45 | HMDB00296 | Uridine - H2O | 244.0695 | 460.1173 | 8.67 | 0.0005 | 0.22 | 0.28 | 1.45E-06 |
| 7 | 386.0904 | 8.06 | 8.74 | HMDB00292 | Xanthine | 152.0334 | 386.0917 | 8.95 | 0.0013 | 0.21 | 0.21 | 1.50E-06 |
| 8 | 363.1014 | 8.57 | 9.3 | HMDB00148 | L-Glutamic Acid - H2O | 147.0532 | 363.1009 | 9.46 | 0.0005 | 0.16 | 1.59 | 7.18E-07 |
| 9 | 349.122 | 9.4 | 10.21 | HMDB00162 | L-Proline | 115.0633 | 349.1216 | 10.18 | 0.0004 | 0.03 | 1.52 | 7.05E-05 |
| 10 | 306.6361 | 9.75 | 10.61 | HMDB01257 | Spermidine | 145.1579 | 306.6373 | 10.54 | 0.0012 | 0.07 | 0.53 | 1.99E-06 |
| 11 | 426.1178 | 9.96 | 10.84 | HMDB00939 | S-Adenosylhomocysteine | 384.1216 | 426.1191 | 10.52 | 0.0013 | 0.32 | 0.57 | 2.25E-05 |
| 12 | 361.1315 | 9.99 | 10.88 | HMDB03464 | 4-Guanidinobutanoic acid - H2O | 145.0851 | 361.1329 | 11 | 0.0014 | 0.12 | 0.63 | 5.65E-04 |
| 13 | 346.0847 | 10.37 | 11.26 | HMDB00300 | Uracil | 112.0273 | 346.0856 | 11.34 | 0.0009 | 0.08 | 0.36 | 2.86E-09 |
| 14 | 368.0842 | 14.87 | 15.93 | HMDB00676 | L-Homocystine | 268.0551 | 368.0859 | 15.82 | 0.0017 | 0.11 | 0.30 | 4.24E-03 |
| 15 | 300.102 | 15.45 | 16.55 | HMDB00214 | Ornithine | 132.0899 | 300.1033 | 16.58 | 0.0013 | 0.03 | 0.44 | 2.88E-06 |
| 16 | 278.1082 | 19.82 | 21.03 | HMDB01414 | 1_4-diaminobutane | 88.1 | 278.1083 | 21.27 | 0.0001 | 0.24 | 0.59 | 1.60E-04 |
| 17 | 285.1156 | 21.13 | 22.36 | HMDB02322 | Cadaverine | 102.1157 | 285.1162 | 22.39 | 0.0006 | 0.03 | 0.50 | 3.00E-07 |
| 18 | 356.1293 | 24.33 | 25.56 | HMDB29306 | 4-Ethylphenol | 122.0732 | 356.1315 | 25.63 | 0.0022 | 0.07 | 0.64 | 1.47E-04 |

MyCompoundID and METLIN accurate mass search result.

|  | **HMDB ID** | **HMDB Link** | **Common Name** | **m/z (Da)** | **Formula** | **Mass Error (ppm)** | **Fold Change (mutated/wildtype)** | **p-value** |
| --- | --- | --- | --- | --- | --- | --- | --- | --- |
| 1 | HMDB00576 | [HMDB Link](http://www.hmdb.ca/metabolites/HMDB00576) | Monoethyl malonic acid | 132.04226 | C5H8O4 | 3.183819 | 0.43 | 1.25E-08 |
| 1 | HMDB00622 | [HMDB Link](http://www.hmdb.ca/metabolites/HMDB00622) | Ethylmalonic acid | 132.04226 | C5H8O4 | 3.183819 | 0.43 | 1.25E-08 |
| 1 | HMDB00661 | [HMDB Link](http://www.hmdb.ca/metabolites/HMDB00661) | Glutaric acid | 132.04226 | C5H8O4 | 3.183819 | 0.43 | 1.25E-08 |
| 1 | HMDB01844 | [HMDB Link](http://www.hmdb.ca/metabolites/HMDB01844) | Methylsuccinic acid | 132.04226 | C5H8O4 | 3.183819 | 0.43 | 1.25E-08 |
| 1 | HMDB02001 | [HMDB Link](http://www.hmdb.ca/metabolites/HMDB02001) | Dimethylmalonic acid | 132.04226 | C5H8O4 | 3.183819 | 0.43 | 1.25E-08 |
| 1 | HMDB06833 | [HMDB Link](http://www.hmdb.ca/metabolites/HMDB06833) | 2-Acetolactate | 132.04226 | C5H8O4 | 3.183819 | 0.43 | 1.25E-08 |
| 1 | HMDB06855 | [HMDB Link](http://www.hmdb.ca/metabolites/HMDB06855) | (S)-2-Acetolactate | 132.04226 | C5H8O4 | 3.183819 | 0.43 | 1.25E-08 |
| 2 | HMDB00321 | [HMDB Link](http://www.hmdb.ca/metabolites/HMDB00321) | 2-Hydroxyadipic acid | 162.052825 | C6H10O5 | 1.384115 | 2.01 | 6.34E-07 |
| 2 | HMDB00345 | [HMDB Link](http://www.hmdb.ca/metabolites/HMDB00345) | 3-Hydroxyadipic acid | 162.052825 | C6H10O5 | 1.384115 | 2.01 | 6.34E-07 |
| 2 | HMDB00355 | [HMDB Link](http://www.hmdb.ca/metabolites/HMDB00355) | 3-Hydroxymethylglutaric acid | 162.052825 | C6H10O5 | 1.384115 | 2.01 | 6.34E-07 |
| 2 | HMDB00368 | [HMDB Link](http://www.hmdb.ca/metabolites/HMDB00368) | 2(R)-Hydroxyadipic acid | 162.052825 | C6H10O5 | 1.384115 | 2.01 | 6.34E-07 |
| 2 | HMDB00640 | [HMDB Link](http://www.hmdb.ca/metabolites/HMDB00640) | Glucosan | 162.052825 | C6H10O5 | 1.384115 | 2.01 | 6.34E-07 |
| 3 | HMDB00122 | [HMDB Link](http://www.hmdb.ca/metabolites/HMDB00122) | D-Glucose | 180.06339 | C6H12O6 | -0.221589 | 2.35 | 9.40E-06 |
| 3 | HMDB00143 | [HMDB Link](http://www.hmdb.ca/metabolites/HMDB00143) | D-Galactose | 180.06339 | C6H12O6 | -0.221589 | 2.35 | 9.40E-06 |
| 3 | HMDB00169 | [HMDB Link](http://www.hmdb.ca/metabolites/HMDB00169) | D-Mannose | 180.06339 | C6H12O6 | -0.221589 | 2.35 | 9.40E-06 |
| 3 | HMDB00211 | [HMDB Link](http://www.hmdb.ca/metabolites/HMDB00211) | Myoinositol | 180.06339 | C6H12O6 | -0.221589 | 2.35 | 9.40E-06 |
| 3 | HMDB00346 | [HMDB Link](http://www.hmdb.ca/metabolites/HMDB00346) | 3-Deoxyarabinohexonic acid | 180.06339 | C6H12O6 | -0.221589 | 2.35 | 9.40E-06 |
| 3 | HMDB00516 | [HMDB Link](http://www.hmdb.ca/metabolites/HMDB00516) | Beta-D-Glucose | 180.06339 | C6H12O6 | -0.221589 | 2.35 | 9.40E-06 |
| 3 | HMDB00660 | [HMDB Link](http://www.hmdb.ca/metabolites/HMDB00660) | D-Fructose | 180.06339 | C6H12O6 | -0.221589 | 2.35 | 9.40E-06 |
| 3 | HMDB01151 | [HMDB Link](http://www.hmdb.ca/metabolites/HMDB01151) | Allose | 180.06339 | C6H12O6 | -0.221589 | 2.35 | 9.40E-06 |
| 3 | HMDB01266 | [HMDB Link](http://www.hmdb.ca/metabolites/HMDB01266) | L-Sorbose | 180.06339 | C6H12O6 | -0.221589 | 2.35 | 9.40E-06 |
| 3 | HMDB03345 | [HMDB Link](http://www.hmdb.ca/metabolites/HMDB03345) | Alpha-D-Glucose | 180.06339 | C6H12O6 | -0.221589 | 2.35 | 9.40E-06 |
| 3 | HMDB03418 | [HMDB Link](http://www.hmdb.ca/metabolites/HMDB03418) | D-Tagatose | 180.06339 | C6H12O6 | -0.221589 | 2.35 | 9.40E-06 |
| 3 | HMDB03449 | [HMDB Link](http://www.hmdb.ca/metabolites/HMDB03449) | Beta-D-Galactose | 180.06339 | C6H12O6 | -0.221589 | 2.35 | 9.40E-06 |
| 3 | HMDB06088 | [HMDB Link](http://www.hmdb.ca/metabolites/HMDB06088) | Scyllitol | 180.06339 | C6H12O6 | -0.221589 | 2.35 | 9.40E-06 |
| 3 | HMDB12326 | [HMDB Link](http://www.hmdb.ca/metabolites/HMDB12326) | L-Gulose | 180.06339 | C6H12O6 | -0.221589 | 2.35 | 9.40E-06 |
| 4 | HMDB01904 | [HMDB Link](http://www.hmdb.ca/metabolites/HMDB01904) | 3-Nitrotyrosine | 226.058973 | C9H10N2O5 | -2.183063 | 0.28 | 1.45E-06 |
| 5 | HMDB00133 | [HMDB Link](http://www.hmdb.ca/metabolites/HMDB00133) | Guanosine | 283.09167 | C10H13N5O5 | -2.654977 | 0.41 | 2.52E-07 |
| 5 | HMDB03333 | [HMDB Link](http://www.hmdb.ca/metabolites/HMDB03333) | 8-Hydroxy-deoxyguanosine | 283.09167 | C10H13N5O5 | -2.654977 | 0.41 | 2.52E-07 |
| 6 | HMDB00879 | [HMDB Link](http://www.hmdb.ca/metabolites/HMDB00879) | Tetrahydrodeoxycorticosterone | 334.250795 | C21H34O3 | -1.495885 | 0.29 | 1.25E-05 |
| 7 | HMDB00048 | [HMDB Link](http://www.hmdb.ca/metabolites/HMDB00048) | Melibiose | 342.116215 | C12H22O11 | -0.847374 | 2.42 | 1.30E-08 |
| 7 | HMDB00055 | [HMDB Link](http://www.hmdb.ca/metabolites/HMDB00055) | Cellobiose | 342.116215 | C12H22O11 | -0.847374 | 2.42 | 1.30E-08 |
| 7 | HMDB00163 | [HMDB Link](http://www.hmdb.ca/metabolites/HMDB00163) | D-Maltose | 342.116215 | C12H22O11 | -0.847374 | 2.42 | 1.30E-08 |
| 7 | HMDB00186 | [HMDB Link](http://www.hmdb.ca/metabolites/HMDB00186) | Alpha-Lactose | 342.116215 | C12H22O11 | -0.847374 | 2.42 | 1.30E-08 |
| 7 | HMDB00258 | [HMDB Link](http://www.hmdb.ca/metabolites/HMDB00258) | Sucrose | 342.116215 | C12H22O11 | -0.847374 | 2.42 | 1.30E-08 |
| 7 | HMDB00740 | [HMDB Link](http://www.hmdb.ca/metabolites/HMDB00740) | Lactulose | 342.116215 | C12H22O11 | -0.847374 | 2.42 | 1.30E-08 |
| 7 | HMDB00975 | [HMDB Link](http://www.hmdb.ca/metabolites/HMDB00975) | Trehalose | 342.116215 | C12H22O11 | -0.847374 | 2.42 | 1.30E-08 |
| 7 | HMDB02923 | [HMDB Link](http://www.hmdb.ca/metabolites/HMDB02923) | Isomaltose | 342.116215 | C12H22O11 | -0.847374 | 2.42 | 1.30E-08 |
| 7 | HMDB05826 | [HMDB Link](http://www.hmdb.ca/metabolites/HMDB05826) | Galactinol | 342.116215 | C12H22O11 | -0.847374 | 2.42 | 1.30E-08 |
| 7 | HMDB06603 | [HMDB Link](http://www.hmdb.ca/metabolites/HMDB06603) | 3-b-Galactopyranosyl glucose | 342.116215 | C12H22O11 | -0.847374 | 2.42 | 1.30E-08 |
| 7 | HMDB06792 | [HMDB Link](http://www.hmdb.ca/metabolites/HMDB06792) | Epimelibiose | 342.116215 | C12H22O11 | -0.847374 | 2.42 | 1.30E-08 |
| 7 | HMDB11740 | [HMDB Link](http://www.hmdb.ca/metabolites/HMDB11740) | Turanose | 342.116215 | C12H22O11 | -0.847374 | 2.42 | 1.30E-08 |
| 7 | HMDB11742 | [HMDB Link](http://www.hmdb.ca/metabolites/HMDB11742) | Kojibiose | 342.116215 | C12H22O11 | -0.847374 | 2.42 | 1.30E-08 |
| 8 | HMDB01513 | HMDB Link | Dolichyl diphosphate | 344.115381 | C12H26O7P2 | -3.173364 | 2.32 | 1.28E-07 |
| 9 | HMDB11539 | [HMDB Link](http://www.hmdb.ca/metabolites/HMDB11539) | MG(0:0/18:3(6Z,9Z,12Z)/0:0) | 352.26136 | C21H36O4 | -0.794581 | 0.27 | 3.74E-08 |
| 9 | HMDB11540 | [HMDB Link](http://www.hmdb.ca/metabolites/HMDB11540) | MG(0:0/18:3(9Z,12Z,15Z)/0:0) | 352.26136 | C21H36O4 | -0.794581 | 0.27 | 3.74E-08 |
| 9 | HMDB11569 | [HMDB Link](http://www.hmdb.ca/metabolites/HMDB11569) | MG(18:3(6Z,9Z,12Z)/0:0/0:0) | 352.26136 | C21H36O4 | -0.794581 | 0.27 | 3.74E-08 |
| 9 | HMDB11570 | [HMDB Link](http://www.hmdb.ca/metabolites/HMDB11570) | MG(18:3(9Z,12Z,15Z)/0:0/0:0) | 352.26136 | C21H36O4 | -0.794581 | 0.27 | 3.74E-08 |
| 10 | HMDB11538 | [HMDB Link](http://www.hmdb.ca/metabolites/HMDB11538) | MG(0:0/18:2(9Z,12Z)/0:0) | 354.27701 | C21H38O4 | -1.801418 | 0.16 | 2.68E-10 |
| 10 | HMDB11568 | [HMDB Link](http://www.hmdb.ca/metabolites/HMDB11568) | MG(18:2(9Z,12Z)/0:0/0:0) | 354.27701 | C21H38O4 | -1.801418 | 0.16 | 2.68E-10 |
| 11 | HMDB11536 | [HMDB Link](http://www.hmdb.ca/metabolites/HMDB11536) | MG(0:0/18:1(11Z)/0:0) | 356.29266 | C21H40O4 | -4.240914 | 0.10 | 3.48E-09 |
| 11 | HMDB11537 | [HMDB Link](http://www.hmdb.ca/metabolites/HMDB11537) | MG(0:0/18:1(9Z)/0:0) | 356.29266 | C21H40O4 | -4.240914 | 0.10 | 3.48E-09 |
| 11 | HMDB11566 | [HMDB Link](http://www.hmdb.ca/metabolites/HMDB11566) | MG(18:1(11Z)/0:0/0:0) | 356.29266 | C21H40O4 | -4.240914 | 0.10 | 3.48E-09 |
| 11 | HMDB11567 | [HMDB Link](http://www.hmdb.ca/metabolites/HMDB11567) | MG(18:1(9Z)/0:0/0:0) | 356.29266 | C21H40O4 | -4.240914 | 0.10 | 3.48E-09 |
| 12 | HMDB01397 | [HMDB Link](http://www.hmdb.ca/metabolites/HMDB01397) | Guanosine monophosphate | 363.058003 | C10H14N5O8P | -0.327496 | 0.37 | 1.76E-05 |
| 12 | HMDB11670 | [HMDB Link](http://www.hmdb.ca/metabolites/HMDB11670) | 8-Oxo-dGMP | 363.058003 | C10H14N5O8P | -0.327496 | 0.37 | 1.76E-05 |
| 13 | molid63632 | metabo_info.php?molid=63632 | (R)-(+)-2-Pyrrolidone-5-carboxylic acid | 129.0425931 | C5H7NO3 | 3 | 1.59 | 7.18E-07 |
| 13 | molid284 | metabo_info.php?molid=284 | Pyrroline hydroxycarboxylic acid | 129.0425931 | C5H7NO3 | 3 | 1.59 | 7.18E-07 |
| 13 | molid3251 | metabo_info.php?molid=3251 | Pyroglutamic acid | 129.0425931 | C5H7NO3 | 3 | 1.59 | 7.18E-07 |
| 13 | molid6343 | metabo_info.php?molid=6343 | N-Acryloylglycine | 129.042594 | C5H7NO3 | 3 | 1.59 | 7.18E-07 |
| 13 | molid63470 | metabo_info.php?molid=63470 | L-1-Pyrroline-3-hydroxy-5-carboxylate | 129.0425931 | C5H7NO3 | 3 | 1.59 | 7.18E-07 |
| 13 | molid63471 | metabo_info.php?molid=63471 | 4-Oxoproline | 129.0425931 | C5H7NO3 | 3 | 1.59 | 7.18E-07 |
| 13 | molid63483 | metabo_info.php?molid=63483 | 1-Pyrroline-4-hydroxy-2-carboxylate | 129.0425931 | C5H7NO3 | 3 | 1.59 | 7.18E-07 |
| 14 | molid63943 | metabo_info.php?molid=63943 | (S)-4-Amino-5-oxopentanoate | 131.0582432 | C5H9NO3 | 1 | 1.89 | 3.95E-07 |
| 14 | molid22 | metabo_info.php?molid=22 | 3-Hydroxy-L-proline | 131.0582432 | C5H9NO3 | 1 | 1.89 | 3.95E-07 |
| 14 | molid75 | metabo_info.php?molid=75 | 5(d)-Aminolevulinic Acid | 131.0582432 | C5H9NO3 | 1 | 1.89 | 3.95E-07 |
| 14 | molid257 | metabo_info.php?molid=257 | Trans-4-Hydroxy-L-proline | 131.0582432 | C5H9NO3 | 1 | 1.89 | 3.95E-07 |
| 14 | molid295 | metabo_info.php?molid=295 | Glutamic acid g-semialdehyde | 131.0582432 | C5H9NO3 | 1 | 1.89 | 3.95E-07 |
| 14 | molid5733 | metabo_info.php?molid=5733 | N-Acetyl-L-alanine | 131.058244 | C5H9NO3 | 1 | 1.89 | 3.95E-07 |
| 14 | molid5742 | metabo_info.php?molid=5742 | N-Acetylglycine methyl ester | 131.058244 | C5H9NO3 | 1 | 1.89 | 3.95E-07 |
| 14 | molid5749 | metabo_info.php?molid=5749 | Propionylglycine | 131.058244 | C5H9NO3 | 1 | 1.89 | 3.95E-07 |
| 14 | molid35853 | metabo_info.php?molid=35853 | 2-oxo-5-amino-pentanoic acid | 131.0582432 | C5H9NO3 | 1 | 1.89 | 3.95E-07 |
| 14 | molid35854 | metabo_info.php?molid=35854 | 2-amino-4-oxo-pentanoic acid | 131.0582432 | C5H9NO3 | 1 | 1.89 | 3.95E-07 |
| 14 | molid58354 | metabo_info.php?molid=58354 | 4-Hydroxy-L-proline | 131.0582432 | C5H9NO3 | 1 | 1.89 | 3.95E-07 |
| 14 | molid63098 | metabo_info.php?molid=63098 | cis-4-Hydroxy-D-proline | 131.0582432 | C5H9NO3 | 1 | 1.89 | 3.95E-07 |
| 14 | molid63469 | metabo_info.php?molid=63469 | L-Glutamate 5-semialdehyde | 131.0582432 | C5H9NO3 | 1 | 1.89 | 3.95E-07 |
| 14 | molid63572 | metabo_info.php?molid=63572 | N-Acetyl-&beta;-alanine | 131.0582432 | C5H9NO3 | 1 | 1.89 | 3.95E-07 |
| 14 | molid64947 | metabo_info.php?molid=64947 | cis-3-Hydroxy-DL-proline | 131.0582432 | C5H9NO3 | 1 | 1.89 | 3.95E-07 |
| 14 | molid74883 | metabo_info.php?molid=74883 | 5-amino-levulinic acid | 131.0582432 | C5H9NO3 | 1 | 1.89 | 3.95E-07 |
| 14 | molid91648 | metabo_info.php?molid=91648 | 4-Hydroxy-2-pyrrolidinecarboxylic acid | 131.0582432 | C5H9NO3 | 1 | 1.89 | 3.95E-07 |
| 14 | molid44749 | metabo_info.php?molid=44749 | Dimethyl malonate | 132.0422587 | C5H8O4 | 3 | 1.89 | 3.95E-07 |
| 15 | molid5807 | metabo_info.php?molid=5807 | Pteridine | 132.0435961 | C6H4N4 | 3 | 0.38 | 8.99E-08 |
| 16 | molid35997 | metabo_info.php?molid=35997 | 2S-hydroxy-Hexanedioic acid | 162.052801 | C6H10O5 | 4 | 2.45 | 1.78E-08 |
| 16 | molid89928 | metabo_info.php?molid=89928 | (-)-1-Methylpropyl 1-propenyl disulfide | 162.0536918 | C7H14S2 | 3 | 2.45 | 1.78E-08 |
| 17 | molid6428 | metabo_info.php?molid=6428 | DL-Methionine sulfoxide | 165.045966 | C5H11NO3S | 1 | 0.57 | 8.06E-07 |
| 17 | molid63430 | metabo_info.php?molid=63430 | L-Methionine S-oxide | 165.0459639 | C5H11NO3S | 1 | 0.57 | 8.06E-07 |
| 17 | molid92035 | metabo_info.php?molid=92035 | Ethiin | 165.0459639 | C5H11NO3S | 1 | 0.57 | 8.06E-07 |
| 18 | molid72879 | metabo_info.php?molid=72879 | Crimidine | 171.056325 | C7H10ClN3 | 3 | 2.53 | 1.46E-06 |
| 19 | molid1457 | metabo_info.php?molid=1457 | Paraxanthine | 180.0647255 | C7H8N4O2 | 3 | 2.61 | 6.56E-09 |
| 19 | molid1456 | metabo_info.php?molid=1456 | Theobromine | 180.0647255 | C7H8N4O2 | 3 | 2.61 | 6.56E-09 |
| 19 | molid1458 | metabo_info.php?molid=1458 | Theophylline | 180.0647255 | C7H8N4O2 | 3 | 2.61 | 6.56E-09 |
| 20 | molid53568 | metabo_info.php?molid=53568 | (+)-Mayurone | 206.1670653 | C14H22O | 4 | 0.57 | 5.39E-06 |
| 20 | molid43972 | metabo_info.php?molid=43972 | CARYLOPHYLLENE OXIDE | 206.1670653 | C14H22O | 4 | 0.57 | 5.39E-06 |
| 20 | molid44480 | metabo_info.php?molid=44480 | 15-NORCARYOPHYLLEN-3-ONE | 206.1670653 | C14H22O | 4 | 0.57 | 5.39E-06 |
| 20 | molid46472 | metabo_info.php?molid=46472 | 9Z,11E,13-Tetradecatrienal | 206.1670653 | C14H22O | 4 | 0.57 | 5.39E-06 |
| 20 | molid67886 | metabo_info.php?molid=67886 | alpha-Irone | 206.1670653 | C14H22O | 4 | 0.57 | 5.39E-06 |
| 20 | molid69010 | metabo_info.php?molid=69010 | (+)-trans-alpha-Irone | 206.1670653 | C14H22O | 4 | 0.57 | 5.39E-06 |
| 20 | molid69823 | metabo_info.php?molid=69823 | 4-Octylphenol | 206.1670653 | C14H22O | 4 | 0.57 | 5.39E-06 |
| 20 | molid69877 | metabo_info.php?molid=69877 | 4-tert-Octylphenol | 206.1670653 | C14H22O | 4 | 0.57 | 5.39E-06 |
| 20 | molid87908 | metabo_info.php?molid=87908 | delta-Methylionone | 206.1670653 | C14H22O | 4 | 0.57 | 5.39E-06 |
| 20 | molid87909 | metabo_info.php?molid=87909 | 3-Methyl-4-(2,6,6-trimethyl-2-cyclohexen-1-yl)-3-buten-2-one | 206.1670653 | C14H22O | 4 | 0.57 | 5.39E-06 |
| 20 | molid88421 | metabo_info.php?molid=88421 | Methyl-delta-ionone | 206.1670653 | C14H22O | 4 | 0.57 | 5.39E-06 |
| 20 | molid90555 | metabo_info.php?molid=90555 | 1-(2,6,6-Trimethyl-2-cyclohexen-1-yl)-1-penten-3-one | 206.1670653 | C14H22O | 4 | 0.57 | 5.39E-06 |
| 20 | molid91201 | metabo_info.php?molid=91201 | Etaspirene | 206.1670653 | C14H22O | 4 | 0.57 | 5.39E-06 |
| 20 | molid91202 | metabo_info.php?molid=91202 | 10-Isopropyl-2,7-dimethyl-1-oxaspiro[4.5]deca-3,6-diene | 206.1670653 | C14H22O | 4 | 0.57 | 5.39E-06 |
| 20 | molid92875 | metabo_info.php?molid=92875 | 1-(2,6,6-Trimethyl-1-cyclohexen-1-yl)-1-penten-3-one | 206.1670653 | C14H22O | 4 | 0.57 | 5.39E-06 |
| 21 | molid263521 | metabo_info.php?molid=263521 | Cymiazole | 218.0877691 | C12H14N2S | 0 | 1.83 | 2.74E-08 |
| 22 | molid93711 | metabo_info.php?molid=93711 | (2S,3&#39;S)-alpha-Amino-2-carboxy-5-oxo-1-pyrrolidinebutanoic acid | 230.0902716 | C9H14N2O5 | 4 | 0.51 | 1.96E-10 |
| 22 | molid23776 | metabo_info.php?molid=23776 | Pro Asp | 230.090273 | C9H14N2O5 | 4 | 0.51 | 1.96E-10 |
| 22 | molid23849 | metabo_info.php?molid=23849 | Asp Pro | 230.090273 | C9H14N2O5 | 4 | 0.51 | 1.96E-10 |
| 22 | molid45499 | metabo_info.php?molid=45499 | 2-Acetyl-5-tetrahydroxybutyl Imidazole | 230.0902716 | C9H14N2O5 | 4 | 0.51 | 1.96E-10 |
| 22 | molid85675 | metabo_info.php?molid=85675 | Aspartyl-Proline | 230.0902716 | C9H14N2O5 | 4 | 0.51 | 1.96E-10 |
| 22 | molid85911 | metabo_info.php?molid=85911 | Prolyl-Aspartate | 230.0902716 | C9H14N2O5 | 4 | 0.51 | 1.96E-10 |
| 22 | molid87877 | metabo_info.php?molid=87877 | 1-(gamma-Glutamylamino)cyclopropanecarboxylic acid | 230.0902716 | C9H14N2O5 | 4 | 0.51 | 1.96E-10 |
| 23 | molid74304 | metabo_info.php?molid=74304 | (6E,8E,12E,14E)-hexadeca-6,8,12,14-tetraen-10-ynoic acid | 244.1463299 | C16H20O2 | 2 | 0.25 | 2.46E-08 |
| 23 | molid35301 | metabo_info.php?molid=35301 | 6E,8E,12E,14E-Hexadecatetraen-10-ynoic acid | 244.14632 | C16H20O2 | 2 | 0.25 | 2.46E-08 |
| 23 | molid35304 | metabo_info.php?molid=35304 | 6E,8E,12E-Hexadecatrien-10-ynoic acid | 244.14632 | C16H20O2 | 2 | 0.25 | 2.46E-08 |
| 23 | molid43691 | metabo_info.php?molid=43691 | 6-Methoxy-4a-methyl-3,4,4a,9,10,10a-hexahydro-2(1H)-phenanthrenone | 244.1463299 | C16H20O2 | 2 | 0.25 | 2.46E-08 |
| 23 | molid43812 | metabo_info.php?molid=43812 | 14-METHOXY-4,4-BISNOR-8,11,13-PODOCARPATRIEN-3-ONE | 244.1463299 | C16H20O2 | 2 | 0.25 | 2.46E-08 |
| 23 | molid68009 | metabo_info.php?molid=68009 | Arnebinol | 244.1463299 | C16H20O2 | 2 | 0.25 | 2.46E-08 |
| 23 | molid68267 | metabo_info.php?molid=68267 | Geranylbenzoquinone | 244.1463299 | C16H20O2 | 2 | 0.25 | 2.46E-08 |
| 23 | molid74307 | metabo_info.php?molid=74307 | (6E,8E,12E,14Z)-hexadeca-6,8,12,14-tetraen-10-ynoic acid | 244.1463299 | C16H20O2 | 2 | 0.25 | 2.46E-08 |
| 24 | molid72268 | metabo_info.php?molid=72268 | Fludioxonil | 248.0397339 | C12H6F2N2O2 | 0 | 0.28 | 1.81E-06 |
| 24 | molid72787 | metabo_info.php?molid=72787 | Thionazin | 248.0384495 | C8H13N2O3PS | 4 | 0.28 | 1.81E-06 |
| 25 | molid17203 | metabo_info.php?molid=17203 | Ala Gly Lys | 274.164106 | C11H22N4O4 | 4 | 0.59 | 7.81E-07 |
| 25 | molid16140 | metabo_info.php?molid=16140 | Lys Ala Gly | 274.164106 | C11H22N4O4 | 4 | 0.59 | 7.81E-07 |
| 25 | molid16500 | metabo_info.php?molid=16500 | Gly Lys Ala | 274.164106 | C11H22N4O4 | 4 | 0.59 | 7.81E-07 |
| 25 | molid20849 | metabo_info.php?molid=20849 | Gly Ala Lys | 274.164106 | C11H22N4O4 | 4 | 0.59 | 7.81E-07 |
| 25 | molid22435 | metabo_info.php?molid=22435 | Ala Lys Gly | 274.164106 | C11H22N4O4 | 4 | 0.59 | 7.81E-07 |
| 25 | molid22452 | metabo_info.php?molid=22452 | Lys Gly Ala | 274.164106 | C11H22N4O4 | 4 | 0.59 | 7.81E-07 |
| 25 | molid23744 | metabo_info.php?molid=23744 | Gln Lys | 274.164106 | C11H22N4O4 | 4 | 0.59 | 7.81E-07 |
| 25 | molid23754 | metabo_info.php?molid=23754 | Lys Gln | 274.164106 | C11H22N4O4 | 4 | 0.59 | 7.81E-07 |
| 25 | molid85716 | metabo_info.php?molid=85716 | Glutaminyl-Lysine | 274.1641052 | C11H22N4O4 | 4 | 0.59 | 7.81E-07 |
| 25 | molid85851 | metabo_info.php?molid=85851 | Lysyl-Glutamine | 274.1641052 | C11H22N4O4 | 4 | 0.59 | 7.81E-07 |
| 25 | molid85867 | metabo_info.php?molid=85867 | Lysyl-Gamma-glutamate | 274.1641052 | C11H22N4O4 | 4 | 0.59 | 7.81E-07 |
| 25 | molid86046 | metabo_info.php?molid=86046 | Gamma-glutamyl-Lysine | 274.1641052 | C11H22N4O4 | 4 | 0.59 | 7.81E-07 |
| 26 | molid74925 | metabo_info.php?molid=74925 | 8E-Heptadecenedioic acid | 298.2144094 | C17H30O4 | 4 | 0.27 | 5.28E-12 |
| 26 | molid71590 | metabo_info.php?molid=71590 | Plakortic acid | 298.2144094 | C17H30O4 | 4 | 0.27 | 5.28E-12 |
| 27 | molid86570 | metabo_info.php?molid=86570 | Starch acetate | 306.0950822 | C12H18O9 | 3 | 2.03 | 5.35E-06 |
| 28 | molid84966 | metabo_info.php?molid=84966 | (&plusmn;)8-GINGEROL | 322.2144094 | C19H30O4 | 3 | 0.25 | 1.07E-09 |
| 28 | molid44495 | metabo_info.php?molid=44495 | CLOVANEDIOL DIACETATE | 322.2144094 | C19H30O4 | 3 | 0.25 | 1.07E-09 |
| 28 | molid68305 | metabo_info.php?molid=68305 | Rapanone | 322.2144094 | C19H30O4 | 3 | 0.25 | 1.07E-09 |
| 28 | molid70945 | metabo_info.php?molid=70945 | Decylubiquinone | 322.2144094 | C19H30O4 | 3 | 0.25 | 1.07E-09 |
| 28 | molid71735 | metabo_info.php?molid=71735 | (8)-Gingerol | 322.2144094 | C19H30O4 | 3 | 0.25 | 1.07E-09 |
| 29 | molid63413 | metabo_info.php?molid=63413 | 3\'-UMP | 324.0358665 | C9H13N2O9P | 3 | 0.62 | 2.34E-06 |
| 29 | molid678 | metabo_info.php?molid=678 | Arabinoside uridinemonophosphate | 324.035871 | C9H13N2O9P | 3 | 0.62 | 2.34E-06 |
| 29 | molid684 | metabo_info.php?molid=684 | Arabinose Uridinemonophosphate | 324.035871 | C9H13N2O9P | 3 | 0.62 | 2.34E-06 |
| 29 | molid3453 | metabo_info.php?molid=3453 | Uridine monophosphate (UMP) | 324.0358665 | C9H13N2O9P | 3 | 0.62 | 2.34E-06 |
| 29 | molid58032 | metabo_info.php?molid=58032 | Pseudouridine 5\'-phosphate | 324.0358665 | C9H13N2O9P | 3 | 0.62 | 2.34E-06 |
| 29 | molid62394 | metabo_info.php?molid=62394 | Uridine 2\'-phosphate | 324.0358665 | C9H13N2O9P | 3 | 0.62 | 2.34E-06 |
| 30 | molid66635 | metabo_info.php?molid=66635 | Prazepam | 324.1029409 | C19H17ClN2O | 3 | 2.10 | 1.56E-07 |
| 30 | molid95426 | metabo_info.php?molid=95426 | Polixetonium chloride | 324.105392 | C10H28Cl2N2OP2 | 4 | 2.10 | 1.56E-07 |
| 30 | molid66164 | metabo_info.php?molid=66164 | Bis-D-fructose 2\',1:2,1\'-dianhydride | 324.1056469 | C12H20O10 | 4 | 2.10 | 1.56E-07 |
| 30 | molid66178 | metabo_info.php?molid=66178 | D-Fructofuranose 1,2\':2,3\'-dianhydride | 324.1056469 | C12H20O10 | 4 | 2.10 | 1.56E-07 |
| 30 | molid86599 | metabo_info.php?molid=86599 | D-1-Deoxy-erythro-hexo-2,3-diulose | 324.1056469 | C12H20O10 | 4 | 2.10 | 1.56E-07 |
| 31 | molid43724 | metabo_info.php?molid=43724 | AVOCADYNONE ACETATE | 324.2300595 | C19H32O4 | 3 | 0.47 | 2.81E-06 |
| 31 | molid34518 | metabo_info.php?molid=34518 | TOFA | 324.23 | C19H32O4 | 3 | 0.47 | 2.81E-06 |
| 31 | molid70946 | metabo_info.php?molid=70946 | Decylubiquinol | 324.2300595 | C19H32O4 | 3 | 0.47 | 2.81E-06 |
| 31 | molid74463 | metabo_info.php?molid=74463 | methyl-10-hydroperoxy-8E,12Z,15Z-octadecatrienoate | 324.2300595 | C19H32O4 | 3 | 0.47 | 2.81E-06 |
| 31 | molid74464 | metabo_info.php?molid=74464 | methyl 15-hydroperoxy-9Z,12Z,16E-octadecatrienoate | 324.2300595 | C19H32O4 | 3 | 0.47 | 2.81E-06 |
| 32 | molid70630 | metabo_info.php?molid=70630 | 3,3-Difluoro-17-methyl-5alpha-androstan-17beta-ol | 326.2421221 | C20H32F2O | 2 | 0.13 | 1.16E-08 |
| 33 | molid74818 | metabo_info.php?molid=74818 | 11-methoxy-12,13-epoxy-9-octadecenoic acid | 326.2457096 | C19H34O4 | 2 | 0.10 | 5.77E-10 |
| 33 | molid43513 | metabo_info.php?molid=43513 | AVOCADYNE ACETATE | 326.2456964 | C19H34O4 | 2 | 0.10 | 5.77E-10 |
| 33 | molid45935 | metabo_info.php?molid=45935 | Ceriporic acid A | 326.2457096 | C19H34O4 | 2 | 0.10 | 5.77E-10 |
| 33 | molid74813 | metabo_info.php?molid=74813 | 8-methoxy-13-hydroxy-9,11-octadecadienoic acid | 326.2457096 | C19H34O4 | 2 | 0.10 | 5.77E-10 |
| 33 | molid87344 | metabo_info.php?molid=87344 | 1-Acetoxy-2-hydroxy-16-heptadecen-4-one | 326.2457096 | C19H34O4 | 2 | 0.10 | 5.77E-10 |
| 33 | molid87372 | metabo_info.php?molid=87372 | Avocadyne 2-acetate | 326.2457096 | C19H34O4 | 2 | 0.10 | 5.77E-10 |
| 33 | molid87373 | metabo_info.php?molid=87373 | Avocadyne 4-acetate | 326.2457096 | C19H34O4 | 2 | 0.10 | 5.77E-10 |
| 34 | molid24076 | metabo_info.php?molid=24076 | 1-Monopalmitin | 330.2769948 | C19H38O4 | 2 | 0.24 | 2.50E-09 |
| 34 | molid3855 | metabo_info.php?molid=3855 | MG(16:0/0:0/0:0) | 330.27701 | C19H38O4 | 2 | 0.24 | 2.50E-09 |
| 34 | molid62317 | metabo_info.php?molid=62317 | MG(0:0/16:0/0:0) | 330.2770097 | C19H38O4 | 2 | 0.24 | 2.50E-09 |
| 34 | molid75555 | metabo_info.php?molid=75555 | MG(16:0/0:0/0:0)[rac] | 330.2770097 | C19H38O4 | 2 | 0.24 | 2.50E-09 |
| 35 | molid23883 | metabo_info.php?molid=23883 | Arg Tyr | 337.175005 | C15H23N5O4 | 4 | 1.56 | 9.72E-09 |
| 35 | molid23783 | metabo_info.php?molid=23783 | Tyr Arg | 337.175005 | C15H23N5O4 | 4 | 1.56 | 9.72E-09 |
| 35 | molid43911 | metabo_info.php?molid=43911 | d[-Arg-2]KYOTORPHAN | 337.1750042 | C15H23N5O4 | 4 | 1.56 | 9.72E-09 |
| 35 | molid58303 | metabo_info.php?molid=58303 | Kyotorphin | 337.1750042 | C15H23N5O4 | 4 | 1.56 | 9.72E-09 |
| 35 | molid85636 | metabo_info.php?molid=85636 | Arginyl-Tyrosine | 337.1750042 | C15H23N5O4 | 4 | 1.56 | 9.72E-09 |
| 36 | molid34485 | metabo_info.php?molid=34485 | raclopride | 346.085091 | C15H20Cl2N2O3 | 1 | 2.19 | 6.18E-06 |
| 37 | molid2914 | metabo_info.php?molid=2914 | &alpha;-Hydroxytriazolam | 358.038817 | C17H12Cl2N4O | 1 | 0.57 | 2.29E-07 |
| 37 | molid2915 | metabo_info.php?molid=2915 | 4-Hydroxytriazolam | 358.038817 | C17H12Cl2N4O | 1 | 0.57 | 2.29E-07 |
| 38 | molid47756 | metabo_info.php?molid=47756 | 5-Hydroxy-3\',4\'-methylenedioxy-6\'\',6\'\'-dimethylpyrano[2\'\',3\'\':7,8]isoflavone | 364.0946882 | C21H16O6 | 3 | 2.19 | 1.34E-07 |
| 38 | molid47752 | metabo_info.php?molid=47752 | Robustone | 364.0946882 | C21H16O6 | 3 | 2.19 | 1.34E-07 |
| 38 | molid48357 | metabo_info.php?molid=48357 | Gancaonin F | 364.0946882 | C21H16O6 | 3 | 2.19 | 1.34E-07 |
| 38 | molid68451 | metabo_info.php?molid=68451 | Justicidin B | 364.0946882 | C21H16O6 | 3 | 2.19 | 1.34E-07 |
| 38 | molid89160 | metabo_info.php?molid=89160 | Gerberinol | 364.0946882 | C21H16O6 | 3 | 2.19 | 1.34E-07 |
| 39 | molid73484 | metabo_info.php?molid=73484 | Cylindrospermopsin | 415.1161687 | C15H21N5O7S | 4 | 2.52 | 1.34E-08 |
| 40 | molid71340 | metabo_info.php?molid=71340 | AM-toxin I | 445.2212857 | C23H31N3O6 | 1 | 0.36 | 8.39E-07 |
| 41 | molid1778 | metabo_info.php?molid=1778 | 2-Hydroxycisapride | 481.177978 | C23H29ClFN3O5 | 1 | 2.12 | 5.44E-08 |
| 41 | molid1779 | metabo_info.php?molid=1779 | 5-Hydroxycisapride | 481.177978 | C23H29ClFN3O5 | 1 | 2.12 | 5.44E-08 |
| 41 | molid112176 | metabo_info.php?molid=112176 | Cys Cys Arg Thr | 481.1777231 | C16H31N7O6S2 | 0 | 2.12 | 5.44E-08 |
| 41 | molid112214 | metabo_info.php?molid=112214 | Cys Cys Thr Arg | 481.1777231 | C16H31N7O6S2 | 0 | 2.12 | 5.44E-08 |
| 41 | molid117116 | metabo_info.php?molid=117116 | Cys Arg Cys Thr | 481.1777231 | C16H31N7O6S2 | 0 | 2.12 | 5.44E-08 |
| 41 | molid117401 | metabo_info.php?molid=117401 | Cys Arg Thr Cys | 481.1777231 | C16H31N7O6S2 | 0 | 2.12 | 5.44E-08 |
| 41 | molid117914 | metabo_info.php?molid=117914 | Cys Thr Cys Arg | 481.1777231 | C16H31N7O6S2 | 0 | 2.12 | 5.44E-08 |
| 41 | molid118161 | metabo_info.php?molid=118161 | Cys Thr Arg Cys | 481.1777231 | C16H31N7O6S2 | 0 | 2.12 | 5.44E-08 |
| 41 | molid215915 | metabo_info.php?molid=215915 | Arg Cys Cys Thr | 481.1777231 | C16H31N7O6S2 | 0 | 2.12 | 5.44E-08 |
| 41 | molid216200 | metabo_info.php?molid=216200 | Arg Cys Thr Cys | 481.1777231 | C16H31N7O6S2 | 0 | 2.12 | 5.44E-08 |
| 41 | molid221900 | metabo_info.php?molid=221900 | Arg Thr Cys Cys | 481.1777231 | C16H31N7O6S2 | 0 | 2.12 | 5.44E-08 |
| 41 | molid231913 | metabo_info.php?molid=231913 | Thr Cys Cys Arg | 481.1777231 | C16H31N7O6S2 | 0 | 2.12 | 5.44E-08 |
| 41 | molid232160 | metabo_info.php?molid=232160 | Thr Cys Arg Cys | 481.1777231 | C16H31N7O6S2 | 0 | 2.12 | 5.44E-08 |
| 41 | molid237100 | metabo_info.php?molid=237100 | Thr Arg Cys Cys | 481.1777231 | C16H31N7O6S2 | 0 | 2.12 | 5.44E-08 |
| 42 | molid122857 | metabo_info.php?molid=122857 | Asp Lys Lys Val | 488.2958477 | C21H40N6O7 | 1 | 0.38 | 2.88E-09 |
| 42 | molid123028 | metabo_info.php?molid=123028 | Asp Lys Val Lys | 488.2958477 | C21H40N6O7 | 1 | 0.38 | 2.88E-09 |
| 42 | molid126448 | metabo_info.php?molid=126448 | Asp Val Lys Lys | 488.2958477 | C21H40N6O7 | 1 | 0.38 | 2.88E-09 |
| 42 | molid162955 | metabo_info.php?molid=162955 | Ile Lys Gln Thr | 488.2958477 | C21H40N6O7 | 1 | 0.38 | 2.88E-09 |
| 42 | molid163012 | metabo_info.php?molid=163012 | Ile Lys Thr Gln | 488.2958477 | C21H40N6O7 | 1 | 0.38 | 2.88E-09 |
| 42 | molid164855 | metabo_info.php?molid=164855 | Ile Gln Lys Thr | 488.2958477 | C21H40N6O7 | 1 | 0.38 | 2.88E-09 |
| 42 | molid165007 | metabo_info.php?molid=165007 | Ile Gln Thr Lys | 488.2958477 | C21H40N6O7 | 1 | 0.38 | 2.88E-09 |
| 42 | molid166052 | metabo_info.php?molid=166052 | Ile Thr Lys Gln | 488.2958477 | C21H40N6O7 | 1 | 0.38 | 2.88E-09 |
| 42 | molid166147 | metabo_info.php?molid=166147 | Ile Thr Gln Lys | 488.2958477 | C21H40N6O7 | 1 | 0.38 | 2.88E-09 |
| 42 | molid168456 | metabo_info.php?molid=168456 | Lys Asp Lys Val | 488.2958477 | C21H40N6O7 | 1 | 0.38 | 2.88E-09 |
| 42 | molid168627 | metabo_info.php?molid=168627 | Lys Asp Val Lys | 488.2958477 | C21H40N6O7 | 1 | 0.38 | 2.88E-09 |
| 42 | molid170555 | metabo_info.php?molid=170555 | Lys Ile Gln Thr | 488.2958477 | C21H40N6O7 | 1 | 0.38 | 2.88E-09 |
| 42 | molid170612 | metabo_info.php?molid=170612 | Lys Ile Thr Gln | 488.2958477 | C21H40N6O7 | 1 | 0.38 | 2.88E-09 |
| 42 | molid170736 | metabo_info.php?molid=170736 | Lys Lys Asp Val | 488.2958477 | C21H40N6O7 | 1 | 0.38 | 2.88E-09 |
| 42 | molid171021 | metabo_info.php?molid=171021 | Lys Lys Val Asp | 488.2958477 | C21H40N6O7 | 1 | 0.38 | 2.88E-09 |
| 42 | molid171355 | metabo_info.php?molid=171355 | Lys Leu Gln Thr | 488.2958477 | C21H40N6O7 | 1 | 0.38 | 2.88E-09 |
| 42 | molid171412 | metabo_info.php?molid=171412 | Lys Leu Thr Gln | 488.2958477 | C21H40N6O7 | 1 | 0.38 | 2.88E-09 |
| 42 | molid172835 | metabo_info.php?molid=172835 | Lys Gln Ile Thr | 488.2958477 | C21H40N6O7 | 1 | 0.38 | 2.88E-09 |
| 42 | molid172875 | metabo_info.php?molid=172875 | Lys Gln Leu Thr | 488.2958477 | C21H40N6O7 | 1 | 0.38 | 2.88E-09 |
| 42 | molid173006 | metabo_info.php?molid=173006 | Lys Gln Thr Ile | 488.2958477 | C21H40N6O7 | 1 | 0.38 | 2.88E-09 |
| 42 | molid173008 | metabo_info.php?molid=173008 | Lys Gln Thr Leu | 488.2958477 | C21H40N6O7 | 1 | 0.38 | 2.88E-09 |
| 42 | molid174032 | metabo_info.php?molid=174032 | Lys Thr Ile Gln | 488.2958477 | C21H40N6O7 | 1 | 0.38 | 2.88E-09 |
| 42 | molid174072 | metabo_info.php?molid=174072 | Lys Thr Leu Gln | 488.2958477 | C21H40N6O7 | 1 | 0.38 | 2.88E-09 |
| 42 | molid174146 | metabo_info.php?molid=174146 | Lys Thr Gln Ile | 488.2958477 | C21H40N6O7 | 1 | 0.38 | 2.88E-09 |
| 42 | molid174148 | metabo_info.php?molid=174148 | Lys Thr Gln Leu | 488.2958477 | C21H40N6O7 | 1 | 0.38 | 2.88E-09 |
| 42 | molid174327 | metabo_info.php?molid=174327 | Lys Val Asp Lys | 488.2958477 | C21H40N6O7 | 1 | 0.38 | 2.88E-09 |
| 42 | molid174441 | metabo_info.php?molid=174441 | Lys Val Lys Asp | 488.2958477 | C21H40N6O7 | 1 | 0.38 | 2.88E-09 |
| 42 | molid178955 | metabo_info.php?molid=178955 | Leu Lys Gln Thr | 488.2958477 | C21H40N6O7 | 1 | 0.38 | 2.88E-09 |
| 42 | molid179012 | metabo_info.php?molid=179012 | Leu Lys Thr Gln | 488.2958477 | C21H40N6O7 | 1 | 0.38 | 2.88E-09 |
| 42 | molid180855 | metabo_info.php?molid=180855 | Leu Gln Lys Thr | 488.2958477 | C21H40N6O7 | 1 | 0.38 | 2.88E-09 |
| 42 | molid181007 | metabo_info.php?molid=181007 | Leu Gln Thr Lys | 488.2958477 | C21H40N6O7 | 1 | 0.38 | 2.88E-09 |
| 42 | molid182052 | metabo_info.php?molid=182052 | Leu Thr Lys Gln | 488.2958477 | C21H40N6O7 | 1 | 0.38 | 2.88E-09 |
| 42 | molid182147 | metabo_info.php?molid=182147 | Leu Thr Gln Lys | 488.2958477 | C21H40N6O7 | 1 | 0.38 | 2.88E-09 |
| 42 | molid210455 | metabo_info.php?molid=210455 | Gln Ile Lys Thr | 488.2958477 | C21H40N6O7 | 1 | 0.38 | 2.88E-09 |
| 42 | molid210607 | metabo_info.php?molid=210607 | Gln Ile Thr Lys | 488.2958477 | C21H40N6O7 | 1 | 0.38 | 2.88E-09 |
| 42 | molid210835 | metabo_info.php?molid=210835 | Gln Lys Ile Thr | 488.2958477 | C21H40N6O7 | 1 | 0.38 | 2.88E-09 |
| 42 | molid210875 | metabo_info.php?molid=210875 | Gln Lys Leu Thr | 488.2958477 | C21H40N6O7 | 1 | 0.38 | 2.88E-09 |
| 42 | molid211006 | metabo_info.php?molid=211006 | Gln Lys Thr Ile | 488.2958477 | C21H40N6O7 | 1 | 0.38 | 2.88E-09 |
| 42 | molid211008 | metabo_info.php?molid=211008 | Gln Lys Thr Leu | 488.2958477 | C21H40N6O7 | 1 | 0.38 | 2.88E-09 |
| 42 | molid211255 | metabo_info.php?molid=211255 | Gln Leu Lys Thr | 488.2958477 | C21H40N6O7 | 1 | 0.38 | 2.88E-09 |
| 42 | molid211407 | metabo_info.php?molid=211407 | Gln Leu Thr Lys | 488.2958477 | C21H40N6O7 | 1 | 0.38 | 2.88E-09 |
| 42 | molid214027 | metabo_info.php?molid=214027 | Gln Thr Ile Lys | 488.2958477 | C21H40N6O7 | 1 | 0.38 | 2.88E-09 |
| 42 | molid214046 | metabo_info.php?molid=214046 | Gln Thr Lys Ile | 488.2958477 | C21H40N6O7 | 1 | 0.38 | 2.88E-09 |
| 42 | molid214048 | metabo_info.php?molid=214048 | Gln Thr Lys Leu | 488.2958477 | C21H40N6O7 | 1 | 0.38 | 2.88E-09 |
| 42 | molid214067 | metabo_info.php?molid=214067 | Gln Thr Leu Lys | 488.2958477 | C21H40N6O7 | 1 | 0.38 | 2.88E-09 |
| 42 | molid234452 | metabo_info.php?molid=234452 | Thr Ile Lys Gln | 488.2958477 | C21H40N6O7 | 1 | 0.38 | 2.88E-09 |
| 42 | molid234547 | metabo_info.php?molid=234547 | Thr Ile Gln Lys | 488.2958477 | C21H40N6O7 | 1 | 0.38 | 2.88E-09 |
| 42 | molid234832 | metabo_info.php?molid=234832 | Thr Lys Ile Gln | 488.2958477 | C21H40N6O7 | 1 | 0.38 | 2.88E-09 |
| 42 | molid234872 | metabo_info.php?molid=234872 | Thr Lys Leu Gln | 488.2958477 | C21H40N6O7 | 1 | 0.38 | 2.88E-09 |
| 42 | molid234946 | metabo_info.php?molid=234946 | Thr Lys Gln Ile | 488.2958477 | C21H40N6O7 | 1 | 0.38 | 2.88E-09 |
| 42 | molid234948 | metabo_info.php?molid=234948 | Thr Lys Gln Leu | 488.2958477 | C21H40N6O7 | 1 | 0.38 | 2.88E-09 |
| 42 | molid235252 | metabo_info.php?molid=235252 | Thr Leu Lys Gln | 488.2958477 | C21H40N6O7 | 1 | 0.38 | 2.88E-09 |
| 42 | molid235347 | metabo_info.php?molid=235347 | Thr Leu Gln Lys | 488.2958477 | C21H40N6O7 | 1 | 0.38 | 2.88E-09 |
| 42 | molid236827 | metabo_info.php?molid=236827 | Thr Gln Ile Lys | 488.2958477 | C21H40N6O7 | 1 | 0.38 | 2.88E-09 |
| 42 | molid236846 | metabo_info.php?molid=236846 | Thr Gln Lys Ile | 488.2958477 | C21H40N6O7 | 1 | 0.38 | 2.88E-09 |
| 42 | molid236848 | metabo_info.php?molid=236848 | Thr Gln Lys Leu | 488.2958477 | C21H40N6O7 | 1 | 0.38 | 2.88E-09 |
| 42 | molid236867 | metabo_info.php?molid=236867 | Thr Gln Leu Lys | 488.2958477 | C21H40N6O7 | 1 | 0.38 | 2.88E-09 |
| 42 | molid240447 | metabo_info.php?molid=240447 | Val Asp Lys Lys | 488.2958477 | C21H40N6O7 | 1 | 0.38 | 2.88E-09 |
| 42 | molid242727 | metabo_info.php?molid=242727 | Val Lys Asp Lys | 488.2958477 | C21H40N6O7 | 1 | 0.38 | 2.88E-09 |
| 42 | molid242841 | metabo_info.php?molid=242841 | Val Lys Lys Asp | 488.2958477 | C21H40N6O7 | 1 | 0.38 | 2.88E-09 |
| 43 | molid45462 | metabo_info.php?molid=45462 | BIX01294 | 490.3056245 | C28H38N6O2 | 4 | 0.36 | 1.26E-07 |
| 43 | molid82349 | metabo_info.php?molid=82349 | PA(22:2(13Z,16Z)/0:0) | 490.3059404 | C25H47O7P | 4 | 0.36 | 1.26E-07 |
| 44 | molid192538 | metabo_info.php?molid=192538 | Asn Asp Pro Tyr | 507.1965276 | C22H29N5O9 | 3 | 0.65 | 2.45E-07 |
| 44 | molid16919 | metabo_info.php?molid=16919 | Tyr Tyr Tyr | 507.200552 | C27H29N3O7 | 3 | 0.65 | 2.45E-07 |
| 44 | molid112748 | metabo_info.php?molid=112748 | Cys Glu Glu Lys | 507.1998984 | C19H33N5O9S | 2 | 0.65 | 2.45E-07 |
| 44 | molid112843 | metabo_info.php?molid=112843 | Cys Glu Lys Glu | 507.1998984 | C19H33N5O9S | 2 | 0.65 | 2.45E-07 |
| 44 | molid114743 | metabo_info.php?molid=114743 | Cys Lys Glu Glu | 507.1998984 | C19H33N5O9S | 2 | 0.65 | 2.45E-07 |
| 44 | molid120450 | metabo_info.php?molid=120450 | Asp Asp Lys Met | 507.1998984 | C19H33N5O9S | 2 | 0.65 | 2.45E-07 |
| 44 | molid120488 | metabo_info.php?molid=120488 | Asp Asp Met Lys | 507.1998984 | C19H33N5O9S | 2 | 0.65 | 2.45E-07 |
| 44 | molid122730 | metabo_info.php?molid=122730 | Asp Lys Asp Met | 507.1998984 | C19H33N5O9S | 2 | 0.65 | 2.45E-07 |
| 44 | molid122882 | metabo_info.php?molid=122882 | Asp Lys Met Asp | 507.1998984 | C19H33N5O9S | 2 | 0.65 | 2.45E-07 |
| 44 | molid123528 | metabo_info.php?molid=123528 | Asp Met Asp Lys | 507.1998984 | C19H33N5O9S | 2 | 0.65 | 2.45E-07 |
| 44 | molid123642 | metabo_info.php?molid=123642 | Asp Met Lys Asp | 507.1998984 | C19H33N5O9S | 2 | 0.65 | 2.45E-07 |
| 44 | molid124139 | metabo_info.php?molid=124139 | Asp Asn Pro Tyr | 507.1965276 | C22H29N5O9 | 3 | 0.65 | 2.45E-07 |
| 44 | molid124272 | metabo_info.php?molid=124272 | Asp Asn Tyr Pro | 507.1965276 | C22H29N5O9 | 3 | 0.65 | 2.45E-07 |
| 44 | molid124519 | metabo_info.php?molid=124519 | Asp Pro Asn Tyr | 507.1965276 | C22H29N5O9 | 3 | 0.65 | 2.45E-07 |
| 44 | molid124671 | metabo_info.php?molid=124671 | Asp Pro Tyr Asn | 507.1965276 | C22H29N5O9 | 3 | 0.65 | 2.45E-07 |
| 44 | molid125818 | metabo_info.php?molid=125818 | Asp Ser Thr Trp | 507.1965276 | C22H29N5O9 | 3 | 0.65 | 2.45E-07 |
| 44 | molid125856 | metabo_info.php?molid=125856 | Asp Ser Trp Thr | 507.1965276 | C22H29N5O9 | 3 | 0.65 | 2.45E-07 |
| 44 | molid126198 | metabo_info.php?molid=126198 | Asp Thr Ser Trp | 507.1965276 | C22H29N5O9 | 3 | 0.65 | 2.45E-07 |
| 44 | molid126255 | metabo_info.php?molid=126255 | Asp Thr Trp Ser | 507.1965276 | C22H29N5O9 | 3 | 0.65 | 2.45E-07 |
| 44 | molid126996 | metabo_info.php?molid=126996 | Asp Trp Ser Thr | 507.1965276 | C22H29N5O9 | 3 | 0.65 | 2.45E-07 |
| 44 | molid127015 | metabo_info.php?molid=127015 | Asp Trp Thr Ser | 507.1965276 | C22H29N5O9 | 3 | 0.65 | 2.45E-07 |
| 44 | molid127312 | metabo_info.php?molid=127312 | Asp Tyr Asn Pro | 507.1965276 | C22H29N5O9 | 3 | 0.65 | 2.45E-07 |
| 44 | molid127331 | metabo_info.php?molid=127331 | Asp Tyr Pro Asn | 507.1965276 | C22H29N5O9 | 3 | 0.65 | 2.45E-07 |
| 44 | molid127948 | metabo_info.php?molid=127948 | Glu Cys Glu Lys | 507.1998984 | C19H33N5O9S | 2 | 0.65 | 2.45E-07 |
| 44 | molid128043 | metabo_info.php?molid=128043 | Glu Cys Lys Glu | 507.1998984 | C19H33N5O9S | 2 | 0.65 | 2.45E-07 |
| 44 | molid128708 | metabo_info.php?molid=128708 | Glu Glu Cys Lys | 507.1998984 | C19H33N5O9S | 2 | 0.65 | 2.45E-07 |
| 44 | molid128841 | metabo_info.php?molid=128841 | Glu Glu Lys Cys | 507.1998984 | C19H33N5O9S | 2 | 0.65 | 2.45E-07 |
| 44 | molid130703 | metabo_info.php?molid=130703 | Glu Lys Cys Glu | 507.1998984 | C19H33N5O9S | 2 | 0.65 | 2.45E-07 |
| 44 | molid130741 | metabo_info.php?molid=130741 | Glu Lys Glu Cys | 507.1998984 | C19H33N5O9S | 2 | 0.65 | 2.45E-07 |
| 44 | molid131756 | metabo_info.php?molid=131756 | Glu Met Gln Thr | 507.1998984 | C19H33N5O9S | 2 | 0.65 | 2.45E-07 |
| 44 | molid131813 | metabo_info.php?molid=131813 | Glu Met Thr Gln | 507.1998984 | C19H33N5O9S | 2 | 0.65 | 2.45E-07 |
| 44 | molid132896 | metabo_info.php?molid=132896 | Glu Gln Met Thr | 507.1998984 | C19H33N5O9S | 2 | 0.65 | 2.45E-07 |
| 44 | molid133010 | metabo_info.php?molid=133010 | Glu Gln Thr Met | 507.1998984 | C19H33N5O9S | 2 | 0.65 | 2.45E-07 |
| 44 | molid133798 | metabo_info.php?molid=133798 | Glu Ser Ser Trp | 507.1965276 | C22H29N5O9 | 3 | 0.65 | 2.45E-07 |
| 44 | molid133855 | metabo_info.php?molid=133855 | Glu Ser Trp Ser | 507.1965276 | C22H29N5O9 | 3 | 0.65 | 2.45E-07 |
| 44 | molid134093 | metabo_info.php?molid=134093 | Glu Thr Met Gln | 507.1998984 | C19H33N5O9S | 2 | 0.65 | 2.45E-07 |
| 44 | molid134150 | metabo_info.php?molid=134150 | Glu Thr Gln Met | 507.1998984 | C19H33N5O9S | 2 | 0.65 | 2.45E-07 |
| 44 | molid134995 | metabo_info.php?molid=134995 | Glu Trp Ser Ser | 507.1965276 | C22H29N5O9 | 3 | 0.65 | 2.45E-07 |
| 44 | molid167942 | metabo_info.php?molid=167942 | Lys Cys Glu Glu | 507.1998984 | C19H33N5O9S | 2 | 0.65 | 2.45E-07 |
| 44 | molid168329 | metabo_info.php?molid=168329 | Lys Asp Asp Met | 507.1998984 | C19H33N5O9S | 2 | 0.65 | 2.45E-07 |
| 44 | molid168481 | metabo_info.php?molid=168481 | Lys Asp Met Asp | 507.1998984 | C19H33N5O9S | 2 | 0.65 | 2.45E-07 |
| 44 | molid168702 | metabo_info.php?molid=168702 | Lys Glu Cys Glu | 507.1998984 | C19H33N5O9S | 2 | 0.65 | 2.45E-07 |
| 44 | molid168740 | metabo_info.php?molid=168740 | Lys Glu Glu Cys | 507.1998984 | C19H33N5O9S | 2 | 0.65 | 2.45E-07 |
| 44 | molid171521 | metabo_info.php?molid=171521 | Lys Met Asp Asp | 507.1998984 | C19H33N5O9S | 2 | 0.65 | 2.45E-07 |
| 44 | molid184327 | metabo_info.php?molid=184327 | Met Asp Asp Lys | 507.1998984 | C19H33N5O9S | 2 | 0.65 | 2.45E-07 |
| 44 | molid184441 | metabo_info.php?molid=184441 | Met Asp Lys Asp | 507.1998984 | C19H33N5O9S | 2 | 0.65 | 2.45E-07 |
| 44 | molid184955 | metabo_info.php?molid=184955 | Met Glu Gln Thr | 507.1998984 | C19H33N5O9S | 2 | 0.65 | 2.45E-07 |
| 44 | molid185012 | metabo_info.php?molid=185012 | Met Glu Thr Gln | 507.1998984 | C19H33N5O9S | 2 | 0.65 | 2.45E-07 |
| 44 | molid186721 | metabo_info.php?molid=186721 | Met Lys Asp Asp | 507.1998984 | C19H33N5O9S | 2 | 0.65 | 2.45E-07 |
| 44 | molid188755 | metabo_info.php?molid=188755 | Met Gln Glu Thr | 507.1998984 | C19H33N5O9S | 2 | 0.65 | 2.45E-07 |
| 44 | molid189002 | metabo_info.php?molid=189002 | Met Gln Thr Glu | 507.1998984 | C19H33N5O9S | 2 | 0.65 | 2.45E-07 |
| 44 | molid189952 | metabo_info.php?molid=189952 | Met Thr Glu Gln | 507.1998984 | C19H33N5O9S | 2 | 0.65 | 2.45E-07 |
| 44 | molid190142 | metabo_info.php?molid=190142 | Met Thr Gln Glu | 507.1998984 | C19H33N5O9S | 2 | 0.65 | 2.45E-07 |
| 44 | molid192671 | metabo_info.php?molid=192671 | Asn Asp Tyr Pro | 507.1965276 | C22H29N5O9 | 3 | 0.65 | 2.45E-07 |
| 44 | molid196338 | metabo_info.php?molid=196338 | Asn Pro Asp Tyr | 507.1965276 | C22H29N5O9 | 3 | 0.65 | 2.45E-07 |
| 44 | molid196661 | metabo_info.php?molid=196661 | Asn Pro Tyr Asp | 507.1965276 | C22H29N5O9 | 3 | 0.65 | 2.45E-07 |
| 44 | molid199131 | metabo_info.php?molid=199131 | Asn Tyr Asp Pro | 507.1965276 | C22H29N5O9 | 3 | 0.65 | 2.45E-07 |
| 44 | molid199321 | metabo_info.php?molid=199321 | Asn Tyr Pro Asp | 507.1965276 | C22H29N5O9 | 3 | 0.65 | 2.45E-07 |
| 44 | molid200518 | metabo_info.php?molid=200518 | Pro Asp Asn Tyr | 507.1965276 | C22H29N5O9 | 3 | 0.65 | 2.45E-07 |
| 44 | molid200670 | metabo_info.php?molid=200670 | Pro Asp Tyr Asn | 507.1965276 | C22H29N5O9 | 3 | 0.65 | 2.45E-07 |
| 44 | molid203938 | metabo_info.php?molid=203938 | Pro Asn Asp Tyr | 507.1965276 | C22H29N5O9 | 3 | 0.65 | 2.45E-07 |
| 44 | molid204261 | metabo_info.php?molid=204261 | Pro Asn Tyr Asp | 507.1965276 | C22H29N5O9 | 3 | 0.65 | 2.45E-07 |
| 44 | molid207130 | metabo_info.php?molid=207130 | Pro Tyr Asp Asn | 507.1965276 | C22H29N5O9 | 3 | 0.65 | 2.45E-07 |
| 44 | molid207301 | metabo_info.php?molid=207301 | Pro Tyr Asn Asp | 507.1965276 | C22H29N5O9 | 3 | 0.65 | 2.45E-07 |
| 44 | molid208895 | metabo_info.php?molid=208895 | Gln Glu Met Thr | 507.1998984 | C19H33N5O9S | 2 | 0.65 | 2.45E-07 |
| 44 | molid209009 | metabo_info.php?molid=209009 | Gln Glu Thr Met | 507.1998984 | C19H33N5O9S | 2 | 0.65 | 2.45E-07 |
| 44 | molid211555 | metabo_info.php?molid=211555 | Gln Met Glu Thr | 507.1998984 | C19H33N5O9S | 2 | 0.65 | 2.45E-07 |
| 44 | molid211802 | metabo_info.php?molid=211802 | Gln Met Thr Glu | 507.1998984 | C19H33N5O9S | 2 | 0.65 | 2.45E-07 |
| 44 | molid213949 | metabo_info.php?molid=213949 | Gln Thr Glu Met | 507.1998984 | C19H33N5O9S | 2 | 0.65 | 2.45E-07 |
| 44 | molid214082 | metabo_info.php?molid=214082 | Gln Thr Met Glu | 507.1998984 | C19H33N5O9S | 2 | 0.65 | 2.45E-07 |
| 44 | molid224617 | metabo_info.php?molid=224617 | Ser Asp Thr Trp | 507.1965276 | C22H29N5O9 | 3 | 0.65 | 2.45E-07 |
| 44 | molid224655 | metabo_info.php?molid=224655 | Ser Asp Trp Thr | 507.1965276 | C22H29N5O9 | 3 | 0.65 | 2.45E-07 |
| 44 | molid224997 | metabo_info.php?molid=224997 | Ser Glu Ser Trp | 507.1965276 | C22H29N5O9 | 3 | 0.65 | 2.45E-07 |
| 44 | molid225054 | metabo_info.php?molid=225054 | Ser Glu Trp Ser | 507.1965276 | C22H29N5O9 | 3 | 0.65 | 2.45E-07 |
| 44 | molid229557 | metabo_info.php?molid=229557 | Ser Ser Glu Trp | 507.1965276 | C22H29N5O9 | 3 | 0.65 | 2.45E-07 |
| 44 | molid229842 | metabo_info.php?molid=229842 | Ser Ser Trp Glu | 507.1965276 | C22H29N5O9 | 3 | 0.65 | 2.45E-07 |
| 44 | molid229937 | metabo_info.php?molid=229937 | Ser Thr Asp Trp | 507.1965276 | C22H29N5O9 | 3 | 0.65 | 2.45E-07 |
| 44 | molid230241 | metabo_info.php?molid=230241 | Ser Thr Trp Asp | 507.1965276 | C22H29N5O9 | 3 | 0.65 | 2.45E-07 |
| 44 | molid230735 | metabo_info.php?molid=230735 | Ser Trp Asp Thr | 507.1965276 | C22H29N5O9 | 3 | 0.65 | 2.45E-07 |
| 44 | molid230754 | metabo_info.php?molid=230754 | Ser Trp Glu Ser | 507.1965276 | C22H29N5O9 | 3 | 0.65 | 2.45E-07 |
| 44 | molid230982 | metabo_info.php?molid=230982 | Ser Trp Ser Glu | 507.1965276 | C22H29N5O9 | 3 | 0.65 | 2.45E-07 |
| 44 | molid231001 | metabo_info.php?molid=231001 | Ser Trp Thr Asp | 507.1965276 | C22H29N5O9 | 3 | 0.65 | 2.45E-07 |
| 44 | molid232597 | metabo_info.php?molid=232597 | Thr Asp Ser Trp | 507.1965276 | C22H29N5O9 | 3 | 0.65 | 2.45E-07 |
| 44 | molid232654 | metabo_info.php?molid=232654 | Thr Asp Trp Ser | 507.1965276 | C22H29N5O9 | 3 | 0.65 | 2.45E-07 |
| 44 | molid232892 | metabo_info.php?molid=232892 | Thr Glu Met Gln | 507.1998984 | C19H33N5O9S | 2 | 0.65 | 2.45E-07 |
| 44 | molid232949 | metabo_info.php?molid=232949 | Thr Glu Gln Met | 507.1998984 | C19H33N5O9S | 2 | 0.65 | 2.45E-07 |
| 44 | molid235552 | metabo_info.php?molid=235552 | Thr Met Glu Gln | 507.1998984 | C19H33N5O9S | 2 | 0.65 | 2.45E-07 |
| 44 | molid235742 | metabo_info.php?molid=235742 | Thr Met Gln Glu | 507.1998984 | C19H33N5O9S | 2 | 0.65 | 2.45E-07 |
| 44 | molid236749 | metabo_info.php?molid=236749 | Thr Gln Glu Met | 507.1998984 | C19H33N5O9S | 2 | 0.65 | 2.45E-07 |
| 44 | molid236882 | metabo_info.php?molid=236882 | Thr Gln Met Glu | 507.1998984 | C19H33N5O9S | 2 | 0.65 | 2.45E-07 |
| 44 | molid237537 | metabo_info.php?molid=237537 | Thr Ser Asp Trp | 507.1965276 | C22H29N5O9 | 3 | 0.65 | 2.45E-07 |
| 44 | molid237841 | metabo_info.php?molid=237841 | Thr Ser Trp Asp | 507.1965276 | C22H29N5O9 | 3 | 0.65 | 2.45E-07 |
| 44 | molid238734 | metabo_info.php?molid=238734 | Thr Trp Asp Ser | 507.1965276 | C22H29N5O9 | 3 | 0.65 | 2.45E-07 |
| 44 | molid238981 | metabo_info.php?molid=238981 | Thr Trp Ser Asp | 507.1965276 | C22H29N5O9 | 3 | 0.65 | 2.45E-07 |
| 44 | molid248595 | metabo_info.php?molid=248595 | Trp Asp Ser Thr | 507.1965276 | C22H29N5O9 | 3 | 0.65 | 2.45E-07 |
| 44 | molid248614 | metabo_info.php?molid=248614 | Trp Asp Thr Ser | 507.1965276 | C22H29N5O9 | 3 | 0.65 | 2.45E-07 |
| 44 | molid248994 | metabo_info.php?molid=248994 | Trp Glu Ser Ser | 507.1965276 | C22H29N5O9 | 3 | 0.65 | 2.45E-07 |
| 44 | molid253535 | metabo_info.php?molid=253535 | Trp Ser Asp Thr | 507.1965276 | C22H29N5O9 | 3 | 0.65 | 2.45E-07 |
| 44 | molid253554 | metabo_info.php?molid=253554 | Trp Ser Glu Ser | 507.1965276 | C22H29N5O9 | 3 | 0.65 | 2.45E-07 |
| 44 | molid253782 | metabo_info.php?molid=253782 | Trp Ser Ser Glu | 507.1965276 | C22H29N5O9 | 3 | 0.65 | 2.45E-07 |
| 44 | molid253801 | metabo_info.php?molid=253801 | Trp Ser Thr Asp | 507.1965276 | C22H29N5O9 | 3 | 0.65 | 2.45E-07 |
| 44 | molid253934 | metabo_info.php?molid=253934 | Trp Thr Asp Ser | 507.1965276 | C22H29N5O9 | 3 | 0.65 | 2.45E-07 |
| 44 | molid254181 | metabo_info.php?molid=254181 | Trp Thr Ser Asp | 507.1965276 | C22H29N5O9 | 3 | 0.65 | 2.45E-07 |
| 44 | molid256511 | metabo_info.php?molid=256511 | Tyr Asp Asn Pro | 507.1965276 | C22H29N5O9 | 3 | 0.65 | 2.45E-07 |
| 44 | molid256530 | metabo_info.php?molid=256530 | Tyr Asp Pro Asn | 507.1965276 | C22H29N5O9 | 3 | 0.65 | 2.45E-07 |
| 44 | molid259931 | metabo_info.php?molid=259931 | Tyr Asn Asp Pro | 507.1965276 | C22H29N5O9 | 3 | 0.65 | 2.45E-07 |
| 44 | molid260121 | metabo_info.php?molid=260121 | Tyr Asn Pro Asp | 507.1965276 | C22H29N5O9 | 3 | 0.65 | 2.45E-07 |
| 44 | molid260330 | metabo_info.php?molid=260330 | Tyr Pro Asp Asn | 507.1965276 | C22H29N5O9 | 3 | 0.65 | 2.45E-07 |
| 44 | molid260501 | metabo_info.php?molid=260501 | Tyr Pro Asn Asp | 507.1965276 | C22H29N5O9 | 3 | 0.65 | 2.45E-07 |
| 45 | molid84028 | metabo_info.php?molid=84028 | 27-nor-24S-methylcholestan-3beta,4beta,5alpha,6alpha,7beta,8beta,14alpha,15alpha,24-nonol | 516.3298331 | C27H48O9 | 2 | 0.31 | 1.91E-05 |
| 45 | molid130448 | metabo_info.php?molid=130448 | Glu Ile Lys Lys | 516.3271478 | C23H44N6O7 | 3 | 0.31 | 1.91E-05 |
| 45 | molid130828 | metabo_info.php?molid=130828 | Glu Lys Ile Lys | 516.3271478 | C23H44N6O7 | 3 | 0.31 | 1.91E-05 |
| 45 | molid130847 | metabo_info.php?molid=130847 | Glu Lys Lys Ile | 516.3271478 | C23H44N6O7 | 3 | 0.31 | 1.91E-05 |
| 45 | molid130849 | metabo_info.php?molid=130849 | Glu Lys Lys Leu | 516.3271478 | C23H44N6O7 | 3 | 0.31 | 1.91E-05 |
| 45 | molid130868 | metabo_info.php?molid=130868 | Glu Lys Leu Lys | 516.3271478 | C23H44N6O7 | 3 | 0.31 | 1.91E-05 |
| 45 | molid131248 | metabo_info.php?molid=131248 | Glu Leu Lys Lys | 516.3271478 | C23H44N6O7 | 3 | 0.31 | 1.91E-05 |
| 45 | molid160847 | metabo_info.php?molid=160847 | Ile Glu Lys Lys | 516.3271478 | C23H44N6O7 | 3 | 0.31 | 1.91E-05 |
| 45 | molid162747 | metabo_info.php?molid=162747 | Ile Lys Glu Lys | 516.3271478 | C23H44N6O7 | 3 | 0.31 | 1.91E-05 |
| 45 | molid162842 | metabo_info.php?molid=162842 | Ile Lys Lys Glu | 516.3271478 | C23H44N6O7 | 3 | 0.31 | 1.91E-05 |
| 45 | molid168827 | metabo_info.php?molid=168827 | Lys Glu Ile Lys | 516.3271478 | C23H44N6O7 | 3 | 0.31 | 1.91E-05 |
| 45 | molid168846 | metabo_info.php?molid=168846 | Lys Glu Lys Ile | 516.3271478 | C23H44N6O7 | 3 | 0.31 | 1.91E-05 |
| 45 | molid168848 | metabo_info.php?molid=168848 | Lys Glu Lys Leu | 516.3271478 | C23H44N6O7 | 3 | 0.31 | 1.91E-05 |
| 45 | molid168867 | metabo_info.php?molid=168867 | Lys Glu Leu Lys | 516.3271478 | C23H44N6O7 | 3 | 0.31 | 1.91E-05 |
| 45 | molid170347 | metabo_info.php?molid=170347 | Lys Ile Glu Lys | 516.3271478 | C23H44N6O7 | 3 | 0.31 | 1.91E-05 |
| 45 | molid170442 | metabo_info.php?molid=170442 | Lys Ile Lys Glu | 516.3271478 | C23H44N6O7 | 3 | 0.31 | 1.91E-05 |
| 45 | molid170746 | metabo_info.php?molid=170746 | Lys Lys Glu Ile | 516.3271478 | C23H44N6O7 | 3 | 0.31 | 1.91E-05 |
| 45 | molid170748 | metabo_info.php?molid=170748 | Lys Lys Glu Leu | 516.3271478 | C23H44N6O7 | 3 | 0.31 | 1.91E-05 |
| 45 | molid170822 | metabo_info.php?molid=170822 | Lys Lys Ile Glu | 516.3271478 | C23H44N6O7 | 3 | 0.31 | 1.91E-05 |
| 45 | molid170862 | metabo_info.php?molid=170862 | Lys Lys Leu Glu | 516.3271478 | C23H44N6O7 | 3 | 0.31 | 1.91E-05 |
| 45 | molid171147 | metabo_info.php?molid=171147 | Lys Leu Glu Lys | 516.3271478 | C23H44N6O7 | 3 | 0.31 | 1.91E-05 |
| 45 | molid171242 | metabo_info.php?molid=171242 | Lys Leu Lys Glu | 516.3271478 | C23H44N6O7 | 3 | 0.31 | 1.91E-05 |
| 45 | molid176847 | metabo_info.php?molid=176847 | Leu Glu Lys Lys | 516.3271478 | C23H44N6O7 | 3 | 0.31 | 1.91E-05 |
| 45 | molid178747 | metabo_info.php?molid=178747 | Leu Lys Glu Lys | 516.3271478 | C23H44N6O7 | 3 | 0.31 | 1.91E-05 |
| 45 | molid178842 | metabo_info.php?molid=178842 | Leu Lys Lys Glu | 516.3271478 | C23H44N6O7 | 3 | 0.31 | 1.91E-05 |
| 46 | molid65419 | metabo_info.php?molid=65419 | 6-Desmethoxy hormothamnione triacetate | 526.1475117 | C27H26O11 | 4 | 2.05 | 3.56E-05 |

(b) Profiling the Carboxylic Acids.

| **#** | **Input mass** | **Input rt** | **Calibrated RT** | **HMDB No.** | **Name** | **Monoisotopic molecular mass** | **mz_light** | **RT** | **Mass error** | **RT error** | **Fold Change (mutated/wildtype)** | **p-value** |
| --- | --- | --- | --- | --- | --- | --- | --- | --- | --- | --- | --- | --- |
| 1 | 360.2520424 | 30.91 | 32.14 | HMDB00529 | 5-Dodecenoic acid | 198.162 | 360.2533 | 32.54 | 0.0013 | 0.40 | 2.78 | 1.75E-05 |
| 2 | 442.3303471 | 34.72 | 37.32 | HMDB00673 | Linoleic acid | 280.2402 | 442.3316 | 37.65 | 0.0013 | 0.33 | 6.66 | 2.91E-07 |
| 3 | 444.3461873 | 36.60 | 40.25 | HMDB00207 | Oleic acid | 282.2559 | 444.3472 | 40.67 | 0.0010 | 0.42 | 12.68 | 1.95E-07 |
| 4 | 460.3762238 | 40.98 | 49.17 | HMDB00772 | Nonadecanoic acid | 298.2872 | 460.3785 | 48.88 | 0.0023 | 0.29 | 1.86 | 4.09E-02 |
| 5 | 468.3452465 | 35.16 | 38.68 | HMDB02925 | Eicosatrienoic acid | 306.2559 | 468.3472 | 38.38 | 0.0020 | 0.30 | 3.28 | 2.61E-06 |

MyCompoundID and METLIN accurate mass search result.

| **#** | **inputmass** | **mass** | **mass error (ppm)** | **mz_light** | **name** | **formula** | **kegg** | **Fold Change (mutated/wildtype)** | **p-value** |
| --- | --- | --- | --- | --- | --- | --- | --- | --- | --- |
| 1 | 291.0940 | 291.0954 | 4 | 453.185342 | 2,7-Anhydro-alpha-N-acetylneuraminic acid | C11H17NO8 | C04521 | 0.54 | 9.51E-03 |
| 1 | 291.0940 | 291.0954 | 4 | 453.185342 | 2-Deoxy-2,3-dehydro-N-acetylneuraminic acid | C11H17NO8 | C04580 | 0.54 | 9.51E-03 |
| 2 | 291.0936 | 291.0954 | 6 | 453.1849552 | 2,7-Anhydro-alpha-N-acetylneuraminic acid | C11H17NO8 | C04521 | 0.64 | 4.38E-02 |
| 2 | 291.0936 | 291.0954 | 6 | 453.1849552 | 2-Deoxy-2,3-dehydro-N-acetylneuraminic acid | C11H17NO8 | C04580 | 0.64 | 4.38E-02 |
| 3 | 172.1089 | 172.1099 | 5 | 334.2002694 | n-heptanoyl acetic acid | C9H16O3 |  | 0.15 | 1.27E-06 |
| 3 | 172.1089 | 172.1099 | 5 | 334.2002694 | 3-caproyl propionic acid | C9H16O3 |  | 0.15 | 1.27E-06 |
| 3 | 172.1089 | 172.1099 | 5 | 334.2002694 | 4-n-valeryl butyric acid | C9H16O3 |  | 0.15 | 1.27E-06 |
| 3 | 172.1089 | 172.1099 | 5 | 334.2002694 | n-butyrl n-valeric acid | C9H16O3 |  | 0.15 | 1.27E-06 |
| 3 | 172.1089 | 172.1099 | 5 | 334.2002694 | 6-propionyl n-caproic acid | C9H16O3 |  | 0.15 | 1.27E-06 |
| 3 | 172.1089 | 172.1099 | 5 | 334.2002694 | 7-methyl-3-oxooctanoic acid | C9H16O3 |  | 0.15 | 1.27E-06 |
| 3 | 172.1089 | 172.1099 | 5 | 334.2002694 | 9-hydroxy-5Z-nonenoic acid | C9H16O3 |  | 0.15 | 1.27E-06 |
| 3 | 172.1089 | 172.1099 | 5 | 334.2002694 | 7-methyl-4-oxo-octanoic acid | C9H16O3 |  | 0.15 | 1.27E-06 |
| 3 | 172.1089 | 172.1099 | 5 | 334.2002694 | 7-methyl-6-oxo-octanoic acid | C9H16O3 |  | 0.15 | 1.27E-06 |
| 3 | 172.1089 | 172.1099 | 5 | 334.2002694 | 3-oxo-nonanoic acid | C9H16O3 |  | 0.15 | 1.27E-06 |
| 3 | 172.1089 | 172.1099 | 5 | 334.2002694 | 8-oxo-nonanoic acid | C9H16O3 |  | 0.15 | 1.27E-06 |
| 3 | 172.1089 | 172.1099 | 5 | 334.2002694 | 9-oxo-nonanoic acid | C9H16O3 |  | 0.15 | 1.27E-06 |
| 4 | 184.1087 | 184.1099 | 6 | 346.2000339 | (3R)-3-isopropenyl-6-oxoheptanoic acid | C10H16O3 | C11405 | 2.66 | 1.74E-03 |
| 4 | 184.1087 | 184.1099 | 6 | 346.2000339 | (3S)-3-isopropenyl-6-oxoheptanoic acid | C10H16O3 | C11419 | 2.66 | 1.74E-03 |
| 4 | 184.1087 | 184.1099 | 6 | 346.2000339 | 9-oxo-2E-decenoic acid | C10H16O3 |  | 2.66 | 1.74E-03 |
| 4 | 184.1087 | 184.1099 | 6 | 346.2000339 | 9-oxo-2Z-decenoic acid | C10H16O3 |  | 2.66 | 1.74E-03 |
| 4 | 184.1087 | 184.1099 | 6 | 346.2000339 | 5-oxo-7E-decenoic acid | C10H16O3 |  | 2.66 | 1.74E-03 |
| 4 | 184.1087 | 184.1099 | 6 | 346.2000339 | (1&#39;R)-Nepetalic acid | C10H16O3 |  | 2.66 | 1.74E-03 |
| 4 | 184.1087 | 184.1099 | 6 | 346.2000339 | (S)-Oleuropeic acid | C10H16O3 |  | 2.66 | 1.74E-03 |
| 4 | 184.1087 | 184.1099 | 6 | 346.2000339 | (E)-10-Oxo-8-decenoic acid | C10H16O3 |  | 2.66 | 1.74E-03 |
| 5 | 223.0834 | 223.0845 | 4 | 385.17477 | Methyl o-methoxyhippuric acid | C11H13NO4 |  | 2.24 | 2.49E-04 |
| 6 | 251.0783 | 251.0794 | 4 | 574.253679 | N-Phenylacetylaspartic acid | C12H13NO5 |  | 1.60 | 4.11E-04 |
| 7 | 290.1841 | 290.1882 | 14 | 452.275408 | 8-hydroxy-13Z-octadecene-9,11-diynoic acid | C18H26O3 |  | 9.24 | 6.63E-07 |
| 7 | 290.1841 | 290.1882 | 14 | 452.275408 | 8-hydroxy-17-octadecene-9,11-diynoic acid | C18H26O3 |  | 9.24 | 6.63E-07 |
| 7 | 290.1841 | 290.1882 | 14 | 452.275408 | 8-hydroxy-17-octadecene-10,12-diynoic acid | C18H26O3 |  | 9.24 | 6.63E-07 |
| 7 | 290.1841 | 290.1882 | 14 | 452.275408 | 4-oxo-9Z,11Z,13E,15E-octadecatetraenoic acid | C18H26O3 |  | 9.24 | 6.63E-07 |
| 7 | 290.1841 | 290.1882 | 14 | 452.275408 | 8-oxo-9,11-octadecadiynoic acid | C18H26O3 |  | 9.24 | 6.63E-07 |
| 7 | 290.1841 | 290.1882 | 14 | 452.275408 | (9Z,11E,13E,15Z)-4-Oxo-9,11,13,15-octadecatetraenoic acid | C18H26O3 |  | 9.24 | 6.63E-07 |
| 8 | 268.2024 | 268.2038 | 5 | 430.2937882 | Methoprene acid | C16H28O3 |  | 9.29 | 8.35E-08 |
| 8 | 268.2024 | 268.2038 | 5 | 430.2937882 | (1R,2R)-3-oxo-2-pentyl-cyclopentanehexanoic acid | C16H28O3 |  | 9.29 | 8.35E-08 |
| 8 | 268.2024 | 268.2038 | 5 | 430.2937882 | (1S,2S)-3-oxo-2-pentyl-cyclopentanehexanoic acid | C16H28O3 |  | 9.29 | 8.35E-08 |
| 9 | 316.2600 | 316.2613 | 4 | 478.3513613 | 2,3-dihydroxy stearic acid | C18H36O4 |  | 26.76 | 7.28E-07 |
| 9 | 316.2600 | 316.2613 | 4 | 478.3513613 | 5,6-dihydroxy stearic acid | C18H36O4 |  | 26.76 | 7.28E-07 |
| 9 | 316.2600 | 316.2613 | 4 | 478.3513613 | 6,7-dihydroxy stearic acid | C18H36O4 |  | 26.76 | 7.28E-07 |
| 9 | 316.2600 | 316.2613 | 4 | 478.3513613 | 7,8-dihydroxy stearic acid | C18H36O4 |  | 26.76 | 7.28E-07 |
| 9 | 316.2600 | 316.2613 | 4 | 478.3513613 | 8,9-dihydroxy stearic acid | C18H36O4 |  | 26.76 | 7.28E-07 |
| 9 | 316.2600 | 316.2613 | 4 | 478.3513613 | 9,12-dihydroxy stearic acid | C18H36O4 |  | 26.76 | 7.28E-07 |
| 9 | 316.2600 | 316.2613 | 4 | 478.3513613 | 10,11-dihydroxy stearic acid | C18H36O4 |  | 26.76 | 7.28E-07 |
| 9 | 316.2600 | 316.2613 | 4 | 478.3513613 | 11,12-dihydroxy stearic acid | C18H36O4 |  | 26.76 | 7.28E-07 |
| 9 | 316.2600 | 316.2613 | 4 | 478.3513613 | 12,13-dihydroxy stearic acid | C18H36O4 |  | 26.76 | 7.28E-07 |
| 9 | 316.2600 | 316.2613 | 4 | 478.3513613 | 9R,10S-dihydroxy-stearic acid | C18H36O4 |  | 26.76 | 7.28E-07 |
| 9 | 316.2600 | 316.2613 | 4 | 478.3513613 | 9S,10R-dihydroxy-stearic acid | C18H36O4 |  | 26.76 | 7.28E-07 |
| 9 | 316.2600 | 316.2614 | 4 | 478.3513613 | 9,10-dihydroxy stearic acid | C18H36O4 | C19622 | 26.76 | 7.28E-07 |
| 9 | 316.2600 | 316.2614 | 4 | 478.3513613 | (9S,10S)-9,10-dihydroxyoctadecanoic acid | C18H36O4 | C15988 | 26.76 | 7.28E-07 |
| 9 | 316.2600 | 316.2614 | 4 | 478.3513613 | 15,16-dihydroxy-octadecanoic acid | C18H36O4 |  | 26.76 | 7.28E-07 |
| 9 | 316.2600 | 316.2614 | 4 | 478.3513613 | 4,14-dihydroxy-octadecanoic acid | C18H36O4 |  | 26.76 | 7.28E-07 |
| 9 | 316.2600 | 316.2614 | 4 | 478.3513613 | 9,14-dihydroxy-octadecanoic acid | C18H36O4 |  | 26.76 | 7.28E-07 |
| 10 | 316.1998 | 316.2038 | 12 | 478.2911255 | 19-Hydroky-all-trans-retinoic acid | C20H28O3 |  | 2.05 | 6.52E-04 |
| 10 | 316.1998 | 316.2038 | 12 | 478.2911255 | 19-Hydroxy-13-cis-retinoic acid | C20H28O3 |  | 2.05 | 6.52E-04 |
| 10 | 316.1998 | 316.2038 | 12 | 478.2911255 | TOTAROL-19-CARBOXYLIC ACID | C20H28O3 |  | 2.05 | 6.52E-04 |
| 10 | 316.1998 | 316.2038 | 12 | 478.2911255 | 5,6-Epoxyretinoic acid | C20H28O3 | C16680 | 2.05 | 6.52E-04 |
| 10 | 316.1998 | 316.2038 | 12 | 478.2911255 | all-trans-4-hydroxyretinoic acid | C20H28O3 | C16677 | 2.05 | 6.52E-04 |
| 10 | 316.1998 | 316.2038 | 12 | 478.2911255 | all-trans-18-Hydroxyretinoic acid | C20H28O3 | C16679 | 2.05 | 6.52E-04 |
| 10 | 316.1998 | 316.2038 | 12 | 478.2911255 | Pisiferic acid | C20H28O3 | C09163 | 2.05 | 6.52E-04 |
| 10 | 316.1998 | 316.2038 | 12 | 478.2911255 | ent-17-Oxo-15-kauren-19-oic acid | C20H28O3 |  | 2.05 | 6.52E-04 |
| 10 | 316.1998 | 316.2038 | 12 | 478.2911255 | ent-7-Oxo-8(14),15-pimaradien-19-oic acid | C20H28O3 |  | 2.05 | 6.52E-04 |
| 10 | 316.1998 | 316.2038 | 12 | 478.2911255 | ent-15-Oxo-16-kauren-19-oic acid | C20H28O3 |  | 2.05 | 6.52E-04 |
| 10 | 316.1998 | 316.2038 | 12 | 478.2911255 | 7-Oxo-8,15-isopimaradien-18-oic acid | C20H28O3 |  | 2.05 | 6.52E-04 |
| 10 | 316.1998 | 316.2038 | 12 | 478.2911255 | ent-15,16-Epoxy-1(10),13(16),14-halimatrien-19-oic acid | C20H28O3 |  | 2.05 | 6.52E-04 |
| 11 | 252.2074 | 252.2089 | 6 | 414.2986983 | 9,12-hexadecadienoic acid | C16H28O2 |  | 11.92 | 8.13E-07 |
| 11 | 252.2074 | 252.2089 | 6 | 414.2986983 | 10Z,12E-hexadecadienoic acid | C16H28O2 |  | 11.92 | 8.13E-07 |
| 11 | 252.2074 | 252.2089 | 6 | 414.2986983 | 2E,4E-hexadecadienoic acid | C16H28O2 |  | 11.92 | 8.13E-07 |
| 11 | 252.2074 | 252.2089 | 6 | 414.2986983 | 2E,4Z-hexadecadienoic acid | C16H28O2 |  | 11.92 | 8.13E-07 |
| 11 | 252.2074 | 252.2089 | 6 | 414.2986983 | 3Z,9Z-hexadecadienoic acid | C16H28O2 |  | 11.92 | 8.13E-07 |
| 11 | 252.2074 | 252.2089 | 6 | 414.2986983 | 6,9-hexadecadienoic acid | C16H28O2 |  | 11.92 | 8.13E-07 |
| 11 | 252.2074 | 252.2089 | 6 | 414.2986983 | 6Z,9Z-hexadecadienoic acid | C16H28O2 |  | 11.92 | 8.13E-07 |
| 11 | 252.2074 | 252.2089 | 6 | 414.2986983 | 8Z,10Z-hexadecadienoic acid | C16H28O2 |  | 11.92 | 8.13E-07 |
| 11 | 252.2074 | 252.2089 | 6 | 414.2986983 | 9Z,12Z-hexadecadienoic acid | C16H28O2 |  | 11.92 | 8.13E-07 |
| 11 | 252.2074 | 252.2089 | 6 | 414.2986983 | 7-hexadecynoic acid | C16H28O2 |  | 11.92 | 8.13E-07 |
| 11 | 252.2074 | 252.2089 | 6 | 414.2986983 | Hydnocarpic acid | C16H28O2 |  | 11.92 | 8.13E-07 |
| 11 | 252.2074 | 252.2089 | 6 | 414.2986983 | 7,10-hexadecadienoic acid | C16H28O2 |  | 11.92 | 8.13E-07 |
| 11 | 252.2074 | 252.2089 | 6 | 414.2986983 | 7Z,10Z-hexadecadienoic acid | C16H28O2 |  | 11.92 | 8.13E-07 |
| 11 | 252.2074 | 252.2089 | 6 | 414.2986983 | 5Z,9Z-hexadecadienoic acid | C16H28O2 |  | 11.92 | 8.13E-07 |
| 11 | 252.2074 | 252.2089 | 6 | 414.2986983 | (R)-Hydnocarpic acid | C16H28O2 | C16795 | 11.92 | 8.13E-07 |
| 11 | 252.2074 | 252.2089 | 6 | 414.2986983 | 6,9-hexadecadienoic acid | C16H28O2 |  | 11.92 | 8.13E-07 |
| 11 | 252.2074 | 252.2089 | 6 | 414.2986983 | 6Z,9Z-hexadecadienoic acid | C16H28O2 |  | 11.92 | 8.13E-07 |
| 11 | 252.2074 | 252.2089 | 6 | 414.2986983 | Palmitolinoleic acid | C16H28O2 |  | 11.92 | 8.13E-07 |
| 11 | 252.2074 | 252.2089 | 6 | 414.2986983 | 2-Hexadecynoic acid | C16H28O2 |  | 11.92 | 8.13E-07 |
| 11 | 252.2074 | 252.2089 | 6 | 414.2986983 | 4-Hexadecynoic acid | C16H28O2 |  | 11.92 | 8.13E-07 |
| 11 | 252.2074 | 252.2089 | 6 | 414.2986983 | 7-Hexadecynoic acid; Palmitolic acid | C16H28O2 |  | 11.92 | 8.13E-07 |
| 11 | 252.2074 | 252.2089 | 6 | 414.2986983 | 10-Hexadecynoic acid | C16H28O2 |  | 11.92 | 8.13E-07 |
| 12 | 276.2067 | 276.2089 | 7 | 438.2980676 | 3E,9Z,12Z,15Z-octadecatetraenoic acid | C18H28O2 |  | 2.11 | 3.79E-04 |
| 12 | 276.2067 | 276.2089 | 7 | 438.2980676 | Moroctic acid | C18H28O2 |  | 2.11 | 3.79E-04 |
| 12 | 276.2067 | 276.2089 | 7 | 438.2980676 | 6,9,12,15-octadecatetraenoic acid | C18H28O2 |  | 2.11 | 3.79E-04 |
| 12 | 276.2067 | 276.2089 | 7 | 438.2980676 | &alpha;-parinaric acid | C18H28O2 |  | 2.11 | 3.79E-04 |
| 12 | 276.2067 | 276.2089 | 7 | 438.2980676 | 9,12,15,17-octadecatetraenoic acid | C18H28O2 |  | 2.11 | 3.79E-04 |
| 12 | 276.2067 | 276.2089 | 7 | 438.2980676 | 5,8,11,14-octadecatetraenoic acid | C18H28O2 |  | 2.11 | 3.79E-04 |
| 12 | 276.2067 | 276.2089 | 7 | 438.2980676 | 5,9,12,15-octadecatetraenoic acid | C18H28O2 |  | 2.11 | 3.79E-04 |
| 12 | 276.2067 | 276.2089 | 7 | 438.2980676 | 5Z,8Z,11Z,14Z-octadecatetraenoic acid | C18H28O2 |  | 2.11 | 3.79E-04 |
| 12 | 276.2067 | 276.2089 | 7 | 438.2980676 | 9Z,11Z,13E,15E-octadecatetraenoic acid | C18H28O2 |  | 2.11 | 3.79E-04 |
| 12 | 276.2067 | 276.2089 | 7 | 438.2980676 | 11E,13E-octadecadien-9-ynoic acid | C18H28O2 |  | 2.11 | 3.79E-04 |
| 12 | 276.2067 | 276.2089 | 7 | 438.2980676 | 9,12-Octadecadiynoic Acid | C18H28O2 |  | 2.11 | 3.79E-04 |
| 12 | 276.2067 | 276.2089 | 7 | 438.2980676 | 9Z,12Z-Octadecadien-6-ynoic acid | C18H28O2 |  | 2.11 | 3.79E-04 |
| 12 | 276.2067 | 276.2089 | 7 | 438.2980676 | 9Z,14Z-Octadecadien-12-ynoic acid | C18H28O2 |  | 2.11 | 3.79E-04 |
| 12 | 276.2067 | 276.2089 | 7 | 438.2980676 | trans-2,trans-4,trans-6,cis-11-octadecatetraenoic acid | C18H28O2 |  | 2.11 | 3.79E-04 |
| 12 | 276.2067 | 276.2089 | 7 | 438.2980676 | Stearidonic Acid | C18H28O2 | C16300 | 2.11 | 3.79E-04 |
| 12 | 276.2067 | 276.2089 | 7 | 438.2980676 | cis-Parinaric Acid | C18H28O2 |  | 2.11 | 3.79E-04 |
| 12 | 276.2067 | 276.2089 | 7 | 438.2980676 | trans-3, cis-9, cis-12, cis-15-octadecatetraenoic acid; C18:4n-3,6,9,15 | C18H28O2 |  | 2.11 | 3.79E-04 |
| 12 | 276.2067 | 276.2089 | 7 | 438.2980676 | beta-parinaric acid | C18H28O2 |  | 2.11 | 3.79E-04 |
| 12 | 276.2067 | 276.2089 | 7 | 438.2980676 | 5,8,11,14-octadecatetraenoic acid | C18H28O2 |  | 2.11 | 3.79E-04 |
| 12 | 276.2067 | 276.2089 | 7 | 438.2980676 | 5Z,8Z,11Z,14Z-octadecatetraenoic acid | C18H28O2 |  | 2.11 | 3.79E-04 |
| 12 | 276.2067 | 276.2089 | 7 | 438.2980676 | Acetylenic acids; 11,13-Octadecadien-9-ynoic acid, (E,E)- | C18H28O2 |  | 2.11 | 3.79E-04 |
| 12 | 276.2067 | 276.2089 | 7 | 438.2980676 | 2,5-Octadecadiynoic acid | C18H28O2 |  | 2.11 | 3.79E-04 |
| 12 | 276.2067 | 276.2089 | 7 | 438.2980676 | 2,6-Octadecadiynoic acid | C18H28O2 |  | 2.11 | 3.79E-04 |
| 12 | 276.2067 | 276.2089 | 7 | 438.2980676 | 2,7-Octadecadiynoic acid | C18H28O2 |  | 2.11 | 3.79E-04 |
| 12 | 276.2067 | 276.2089 | 7 | 438.2980676 | 3,6-Octadecadiynoic acid | C18H28O2 |  | 2.11 | 3.79E-04 |
| 12 | 276.2067 | 276.2089 | 7 | 438.2980676 | 3,7-Octadecadiynoic acid | C18H28O2 |  | 2.11 | 3.79E-04 |
| 12 | 276.2067 | 276.2089 | 7 | 438.2980676 | 3,8-Octadecadiynoic acid | C18H28O2 |  | 2.11 | 3.79E-04 |
| 12 | 276.2067 | 276.2089 | 7 | 438.2980676 | 4,6-Octadecadiynoic acid | C18H28O2 |  | 2.11 | 3.79E-04 |
| 12 | 276.2067 | 276.2089 | 7 | 438.2980676 | 4,7-Octadecadiynoic acid | C18H28O2 |  | 2.11 | 3.79E-04 |
| 12 | 276.2067 | 276.2089 | 7 | 438.2980676 | 4,8-Octadecadiynoic acid | C18H28O2 |  | 2.11 | 3.79E-04 |
| 12 | 276.2067 | 276.2089 | 7 | 438.2980676 | 4,9-Octadecadiynoic acid | C18H28O2 |  | 2.11 | 3.79E-04 |
| 12 | 276.2067 | 276.2089 | 7 | 438.2980676 | 5,7-Octadecadiynoic acid | C18H28O2 |  | 2.11 | 3.79E-04 |
| 12 | 276.2067 | 276.2089 | 7 | 438.2980676 | 5,8-Octadecadiynoic acid | C18H28O2 |  | 2.11 | 3.79E-04 |
| 12 | 276.2067 | 276.2089 | 7 | 438.2980676 | 5,9-Octadecadiynoic acid | C18H28O2 |  | 2.11 | 3.79E-04 |
| 12 | 276.2067 | 276.2089 | 7 | 438.2980676 | 5,10-Octadecadiynoic acid | C18H28O2 |  | 2.11 | 3.79E-04 |
| 12 | 276.2067 | 276.2089 | 7 | 438.2980676 | 5,12-Octadecadiynoic acid | C18H28O2 |  | 2.11 | 3.79E-04 |
| 12 | 276.2067 | 276.2089 | 7 | 438.2980676 | 6,8-Octadecadiynoic acid | C18H28O2 |  | 2.11 | 3.79E-04 |
| 12 | 276.2067 | 276.2089 | 7 | 438.2980676 | 6,9-Octadecadiynoic acid | C18H28O2 |  | 2.11 | 3.79E-04 |
| 12 | 276.2067 | 276.2089 | 7 | 438.2980676 | 6,10-Octadecadiynoic acid | C18H28O2 |  | 2.11 | 3.79E-04 |
| 12 | 276.2067 | 276.2089 | 7 | 438.2980676 | 6,11-Octadecadiynoic acid | C18H28O2 |  | 2.11 | 3.79E-04 |
| 12 | 276.2067 | 276.2089 | 7 | 438.2980676 | 6,12-Octadecadiynoic acid | C18H28O2 |  | 2.11 | 3.79E-04 |
| 12 | 276.2067 | 276.2089 | 7 | 438.2980676 | 7,9-Octadecadiynoic acid | C18H28O2 |  | 2.11 | 3.79E-04 |
| 12 | 276.2067 | 276.2089 | 7 | 438.2980676 | 7,10-Octadecadiynoic acid | C18H28O2 |  | 2.11 | 3.79E-04 |
| 12 | 276.2067 | 276.2089 | 7 | 438.2980676 | 7,11-Octadecadiynoic acid | C18H28O2 |  | 2.11 | 3.79E-04 |
| 12 | 276.2067 | 276.2089 | 7 | 438.2980676 | 7,12-Octadecadiynoic acid | C18H28O2 |  | 2.11 | 3.79E-04 |
| 12 | 276.2067 | 276.2089 | 7 | 438.2980676 | 8,10-Octadecadiynoic acid | C18H28O2 |  | 2.11 | 3.79E-04 |
| 12 | 276.2067 | 276.2089 | 7 | 438.2980676 | 8,11-Octadecadiynoic acid | C18H28O2 |  | 2.11 | 3.79E-04 |
| 12 | 276.2067 | 276.2089 | 7 | 438.2980676 | 8,12-Octadecadiynoic acid | C18H28O2 |  | 2.11 | 3.79E-04 |
| 12 | 276.2067 | 276.2089 | 7 | 438.2980676 | 9,11-Octadecadiynoic acid | C18H28O2 |  | 2.11 | 3.79E-04 |
| 12 | 276.2067 | 276.2089 | 7 | 438.2980676 | 9,13-Octadecadiynoic acid | C18H28O2 |  | 2.11 | 3.79E-04 |
| 12 | 276.2067 | 276.2089 | 7 | 438.2980676 | 10,12-Octadecadiynoic acid | C18H28O2 |  | 2.11 | 3.79E-04 |
| 12 | 276.2067 | 276.2089 | 7 | 438.2980676 | 10,13-Octadecadiynoic acid | C18H28O2 |  | 2.11 | 3.79E-04 |
| 12 | 276.2067 | 276.2089 | 7 | 438.2980676 | 10,14-Octadecadiynoic acid | C18H28O2 |  | 2.11 | 3.79E-04 |
| 12 | 276.2067 | 276.2089 | 7 | 438.2980676 | 11,14-Octadecadiynoic acid | C18H28O2 |  | 2.11 | 3.79E-04 |
| 12 | 276.2067 | 276.2089 | 7 | 438.2980676 | 11,15-Octadecadiynoic acid | C18H28O2 |  | 2.11 | 3.79E-04 |
| 12 | 276.2067 | 276.2089 | 7 | 438.2980676 | 12,14-Octadecadiynoic acid | C18H28O2 |  | 2.11 | 3.79E-04 |
| 12 | 276.2067 | 276.2089 | 7 | 438.2980676 | 12,15-Octadecadiynoic acid | C18H28O2 |  | 2.11 | 3.79E-04 |
| 12 | 276.2067 | 276.2089 | 7 | 438.2980676 | 12,16-Octadecadiynoic acid | C18H28O2 |  | 2.11 | 3.79E-04 |
| 12 | 276.2067 | 276.2089 | 7 | 438.2980676 | 13,16-Octadecadiynoic acid | C18H28O2 |  | 2.11 | 3.79E-04 |
| 12 | 276.2067 | 276.2089 | 7 | 438.2980676 | 13,17-Octadecadiynoic acid | C18H28O2 |  | 2.11 | 3.79E-04 |
| 12 | 276.2067 | 276.2089 | 7 | 438.2980676 | 14,17-Octadecadiynoic acid | C18H28O2 |  | 2.11 | 3.79E-04 |
| 12 | 276.2067 | 276.2089 | 7 | 438.2980676 | (9Z,12Z)-octadeca-9,12-dien-6-ynoic acid | C18H28O2 |  | 2.11 | 3.79E-04 |
| 12 | 276.2067 | 276.2089 | 7 | 438.2980676 | (9Z,14Z)-octadeca-9,14-dien-6-ynoic acid | C18H28O2 |  | 2.11 | 3.79E-04 |
| 12 | 276.2067 | 276.2089 | 7 | 438.2980676 | 6-[3]-ladderane-hexanoic acid | C18H28O2 |  | 2.11 | 3.79E-04 |
| 12 | 276.2067 | 276.2089 | 7 | 438.2980676 | 4,8,12,15-Octadecatetraenoic acid | C18H28O2 |  | 2.11 | 3.79E-04 |
| 13 | 270.2176 | 270.2195 | 6 | 432.3089897 | Ambrettolic acid | C16H30O3 |  | 12.16 | 5.96E-07 |
| 13 | 270.2176 | 270.2195 | 6 | 432.3089897 | (+)-12-hydroxy-9Z-hexadecenoic acid | C16H30O3 |  | 12.16 | 5.96E-07 |
| 13 | 270.2176 | 270.2195 | 6 | 432.3089897 | 16-hydroxy-5-hexadecenoic acid | C16H30O3 |  | 12.16 | 5.96E-07 |
| 13 | 270.2176 | 270.2195 | 6 | 432.3089897 | 16-hydroxy-6-hexadecenoic acid | C16H30O3 |  | 12.16 | 5.96E-07 |
| 13 | 270.2176 | 270.2195 | 6 | 432.3089897 | 2-keto palmitic acid | C16H30O3 |  | 12.16 | 5.96E-07 |
| 13 | 270.2176 | 270.2195 | 6 | 432.3089897 | 3-keto palmitic acid | C16H30O3 |  | 12.16 | 5.96E-07 |
| 13 | 270.2176 | 270.2195 | 6 | 432.3089897 | 4-keto palmitic acid | C16H30O3 |  | 12.16 | 5.96E-07 |
| 13 | 270.2176 | 270.2195 | 6 | 432.3089897 | 5-keto palmitic acid | C16H30O3 |  | 12.16 | 5.96E-07 |
| 13 | 270.2176 | 270.2195 | 6 | 432.3089897 | 7-keto palmitic acid | C16H30O3 |  | 12.16 | 5.96E-07 |
| 13 | 270.2176 | 270.2195 | 6 | 432.3089897 | 8-keto palmitic acid | C16H30O3 |  | 12.16 | 5.96E-07 |
| 13 | 270.2176 | 270.2195 | 6 | 432.3089897 | 9-keto palmitic acid | C16H30O3 |  | 12.16 | 5.96E-07 |
| 13 | 270.2176 | 270.2195 | 6 | 432.3089897 | 10-keto palmitic acid | C16H30O3 |  | 12.16 | 5.96E-07 |
| 13 | 270.2176 | 270.2195 | 6 | 432.3089897 | 11-keto palmitic acid | C16H30O3 |  | 12.16 | 5.96E-07 |
| 13 | 270.2176 | 270.2195 | 6 | 432.3089897 | 16-hydroxy-9E-hexadecenoic acid | C16H30O3 |  | 12.16 | 5.96E-07 |
| 13 | 270.2176 | 270.2195 | 6 | 432.3089897 | 16-hydroxy-9Z-hexadecenoic acid | C16H30O3 |  | 12.16 | 5.96E-07 |
| 13 | 270.2176 | 270.2195 | 6 | 432.3089897 | 9-Hexadecenoic acid, 12-hydroxy-, (Z)-(+)- | C16H30O3 |  | 12.16 | 5.96E-07 |
| 13 | 270.2176 | 270.2195 | 6 | 432.3089897 | 5-Hexadecenoic acid, 16-hydroxy-; Delta5-Isoambrettolic acid | C16H30O3 |  | 12.16 | 5.96E-07 |
| 13 | 270.2176 | 270.2195 | 6 | 432.3089897 | 6-Hexadecenoic acid, 16-hydroxy-; Delta6-Isoambrettolic acid | C16H30O3 |  | 12.16 | 5.96E-07 |
| 13 | 270.2176 | 270.2195 | 6 | 432.3089897 | 10-oxo-14-methyl-pentadecanoic acid | C16H30O3 |  | 12.16 | 5.96E-07 |
| 13 | 270.2176 | 270.2195 | 6 | 432.3089897 | 2-methyl-4-oxo-pentadecanoic acid | C16H30O3 |  | 12.16 | 5.96E-07 |
| 13 | 270.2176 | 270.2195 | 6 | 432.3089897 | 15-oxo-hexadecanoic acid | C16H30O3 |  | 12.16 | 5.96E-07 |
| 14 | 292.2004 | 292.2038 | 11 | 454.2917181 | alpha-licanic acid | C18H28O3 | C08319 | 3.60 | 4.54E-07 |
| 14 | 292.2004 | 292.2038 | 11 | 454.2917181 | 12,13S-epoxy-9Z,11,15Z-octadecatrienoic acid | C18H28O3 | C04672 | 3.60 | 4.54E-07 |
| 14 | 292.2004 | 292.2038 | 11 | 454.2917181 | (9R,13R)-12-oxo-phytodienoic acid | C18H28O3 | C13816 | 3.60 | 4.54E-07 |
| 14 | 292.2004 | 292.2038 | 11 | 454.2917181 | 13-epi-12-oxo Phytodienoic Acid | C18H28O3 |  | 3.60 | 4.54E-07 |
| 14 | 292.2004 | 292.2038 | 11 | 454.2917181 | Colnelenic acid | C18H28O3 | C16320 | 3.60 | 4.54E-07 |
| 14 | 292.2004 | 292.2038 | 11 | 454.2917181 | Etherolenic acid | C18H28O3 | C16319 | 3.60 | 4.54E-07 |
| 14 | 292.2004 | 292.2038 | 11 | 454.2917181 | (-)-8-hydroxy-11E,17-octadecadien-9-ynoic acid | C18H28O3 |  | 3.60 | 4.54E-07 |
| 14 | 292.2004 | 292.2038 | 11 | 454.2917181 | 9-hydroxy-10E,14Z-octadecadien-12-ynoic acid | C18H28O3 |  | 3.60 | 4.54E-07 |
| 14 | 292.2004 | 292.2038 | 11 | 454.2917181 | 8-hydroxy-9,11-octadecadiynoic acid | C18H28O3 |  | 3.60 | 4.54E-07 |
| 14 | 292.2004 | 292.2038 | 11 | 454.2917181 | 8-hydroxy-10,12-octadecadiynoic acid | C18H28O3 |  | 3.60 | 4.54E-07 |
| 14 | 292.2004 | 292.2038 | 11 | 454.2917181 | 13-keto-9Z,11E,15Z-octadecatrienoic acid | C18H28O3 |  | 3.60 | 4.54E-07 |
| 15 | 276.2066 | 276.2089 | 8 | 438.2979779 | 3E,9Z,12Z,15Z-octadecatetraenoic acid | C18H28O2 |  | 2.19 | 1.30E-04 |
| 15 | 276.2066 | 276.2089 | 8 | 438.2979779 | Moroctic acid | C18H28O2 |  | 2.19 | 1.30E-04 |
| 15 | 276.2066 | 276.2089 | 8 | 438.2979779 | 6,9,12,15-octadecatetraenoic acid | C18H28O2 |  | 2.19 | 1.30E-04 |
| 15 | 276.2066 | 276.2089 | 8 | 438.2979779 | &alpha;-parinaric acid | C18H28O2 |  | 2.19 | 1.30E-04 |
| 15 | 276.2066 | 276.2089 | 8 | 438.2979779 | 9,12,15,17-octadecatetraenoic acid | C18H28O2 |  | 2.19 | 1.30E-04 |
| 15 | 276.2066 | 276.2089 | 8 | 438.2979779 | 5,8,11,14-octadecatetraenoic acid | C18H28O2 |  | 2.19 | 1.30E-04 |
| 15 | 276.2066 | 276.2089 | 8 | 438.2979779 | 5,9,12,15-octadecatetraenoic acid | C18H28O2 |  | 2.19 | 1.30E-04 |
| 15 | 276.2066 | 276.2089 | 8 | 438.2979779 | 5Z,8Z,11Z,14Z-octadecatetraenoic acid | C18H28O2 |  | 2.19 | 1.30E-04 |
| 15 | 276.2066 | 276.2089 | 8 | 438.2979779 | 9Z,11Z,13E,15E-octadecatetraenoic acid | C18H28O2 |  | 2.19 | 1.30E-04 |
| 15 | 276.2066 | 276.2089 | 8 | 438.2979779 | 11E,13E-octadecadien-9-ynoic acid | C18H28O2 |  | 2.19 | 1.30E-04 |
| 15 | 276.2066 | 276.2089 | 8 | 438.2979779 | 9,12-Octadecadiynoic Acid | C18H28O2 |  | 2.19 | 1.30E-04 |
| 15 | 276.2066 | 276.2089 | 8 | 438.2979779 | 9Z,12Z-Octadecadien-6-ynoic acid | C18H28O2 |  | 2.19 | 1.30E-04 |
| 15 | 276.2066 | 276.2089 | 8 | 438.2979779 | 9Z,14Z-Octadecadien-12-ynoic acid | C18H28O2 |  | 2.19 | 1.30E-04 |
| 15 | 276.2066 | 276.2089 | 8 | 438.2979779 | trans-2,trans-4,trans-6,cis-11-octadecatetraenoic acid | C18H28O2 |  | 2.19 | 1.30E-04 |
| 15 | 276.2066 | 276.2089 | 8 | 438.2979779 | Stearidonic Acid | C18H28O2 | C16300 | 2.19 | 1.30E-04 |
| 15 | 276.2066 | 276.2089 | 8 | 438.2979779 | cis-Parinaric Acid | C18H28O2 |  | 2.19 | 1.30E-04 |
| 15 | 276.2066 | 276.2089 | 8 | 438.2979779 | trans-3, cis-9, cis-12, cis-15-octadecatetraenoic acid; C18:4n-3,6,9,15 | C18H28O2 |  | 2.19 | 1.30E-04 |
| 15 | 276.2066 | 276.2089 | 8 | 438.2979779 | beta-parinaric acid | C18H28O2 |  | 2.19 | 1.30E-04 |
| 15 | 276.2066 | 276.2089 | 8 | 438.2979779 | 5,8,11,14-octadecatetraenoic acid | C18H28O2 |  | 2.19 | 1.30E-04 |
| 15 | 276.2066 | 276.2089 | 8 | 438.2979779 | 5Z,8Z,11Z,14Z-octadecatetraenoic acid | C18H28O2 |  | 2.19 | 1.30E-04 |
| 15 | 276.2066 | 276.2089 | 8 | 438.2979779 | Acetylenic acids; 11,13-Octadecadien-9-ynoic acid, (E,E)- | C18H28O2 |  | 2.19 | 1.30E-04 |
| 15 | 276.2066 | 276.2089 | 8 | 438.2979779 | 2,5-Octadecadiynoic acid | C18H28O2 |  | 2.19 | 1.30E-04 |
| 15 | 276.2066 | 276.2089 | 8 | 438.2979779 | 2,6-Octadecadiynoic acid | C18H28O2 |  | 2.19 | 1.30E-04 |
| 15 | 276.2066 | 276.2089 | 8 | 438.2979779 | 2,7-Octadecadiynoic acid | C18H28O2 |  | 2.19 | 1.30E-04 |
| 15 | 276.2066 | 276.2089 | 8 | 438.2979779 | 3,6-Octadecadiynoic acid | C18H28O2 |  | 2.19 | 1.30E-04 |
| 15 | 276.2066 | 276.2089 | 8 | 438.2979779 | 3,7-Octadecadiynoic acid | C18H28O2 |  | 2.19 | 1.30E-04 |
| 15 | 276.2066 | 276.2089 | 8 | 438.2979779 | 3,8-Octadecadiynoic acid | C18H28O2 |  | 2.19 | 1.30E-04 |
| 15 | 276.2066 | 276.2089 | 8 | 438.2979779 | 4,6-Octadecadiynoic acid | C18H28O2 |  | 2.19 | 1.30E-04 |
| 15 | 276.2066 | 276.2089 | 8 | 438.2979779 | 4,7-Octadecadiynoic acid | C18H28O2 |  | 2.19 | 1.30E-04 |
| 15 | 276.2066 | 276.2089 | 8 | 438.2979779 | 4,8-Octadecadiynoic acid | C18H28O2 |  | 2.19 | 1.30E-04 |
| 15 | 276.2066 | 276.2089 | 8 | 438.2979779 | 4,9-Octadecadiynoic acid | C18H28O2 |  | 2.19 | 1.30E-04 |
| 15 | 276.2066 | 276.2089 | 8 | 438.2979779 | 5,7-Octadecadiynoic acid | C18H28O2 |  | 2.19 | 1.30E-04 |
| 15 | 276.2066 | 276.2089 | 8 | 438.2979779 | 5,8-Octadecadiynoic acid | C18H28O2 |  | 2.19 | 1.30E-04 |
| 15 | 276.2066 | 276.2089 | 8 | 438.2979779 | 5,9-Octadecadiynoic acid | C18H28O2 |  | 2.19 | 1.30E-04 |
| 15 | 276.2066 | 276.2089 | 8 | 438.2979779 | 5,10-Octadecadiynoic acid | C18H28O2 |  | 2.19 | 1.30E-04 |
| 15 | 276.2066 | 276.2089 | 8 | 438.2979779 | 5,12-Octadecadiynoic acid | C18H28O2 |  | 2.19 | 1.30E-04 |
| 15 | 276.2066 | 276.2089 | 8 | 438.2979779 | 6,8-Octadecadiynoic acid | C18H28O2 |  | 2.19 | 1.30E-04 |
| 15 | 276.2066 | 276.2089 | 8 | 438.2979779 | 6,9-Octadecadiynoic acid | C18H28O2 |  | 2.19 | 1.30E-04 |
| 15 | 276.2066 | 276.2089 | 8 | 438.2979779 | 6,10-Octadecadiynoic acid | C18H28O2 |  | 2.19 | 1.30E-04 |
| 15 | 276.2066 | 276.2089 | 8 | 438.2979779 | 6,11-Octadecadiynoic acid | C18H28O2 |  | 2.19 | 1.30E-04 |
| 15 | 276.2066 | 276.2089 | 8 | 438.2979779 | 6,12-Octadecadiynoic acid | C18H28O2 |  | 2.19 | 1.30E-04 |
| 15 | 276.2066 | 276.2089 | 8 | 438.2979779 | 7,9-Octadecadiynoic acid | C18H28O2 |  | 2.19 | 1.30E-04 |
| 15 | 276.2066 | 276.2089 | 8 | 438.2979779 | 7,10-Octadecadiynoic acid | C18H28O2 |  | 2.19 | 1.30E-04 |
| 15 | 276.2066 | 276.2089 | 8 | 438.2979779 | 7,11-Octadecadiynoic acid | C18H28O2 |  | 2.19 | 1.30E-04 |
| 15 | 276.2066 | 276.2089 | 8 | 438.2979779 | 7,12-Octadecadiynoic acid | C18H28O2 |  | 2.19 | 1.30E-04 |
| 15 | 276.2066 | 276.2089 | 8 | 438.2979779 | 8,10-Octadecadiynoic acid | C18H28O2 |  | 2.19 | 1.30E-04 |
| 15 | 276.2066 | 276.2089 | 8 | 438.2979779 | 8,11-Octadecadiynoic acid | C18H28O2 |  | 2.19 | 1.30E-04 |
| 15 | 276.2066 | 276.2089 | 8 | 438.2979779 | 8,12-Octadecadiynoic acid | C18H28O2 |  | 2.19 | 1.30E-04 |
| 15 | 276.2066 | 276.2089 | 8 | 438.2979779 | 9,11-Octadecadiynoic acid | C18H28O2 |  | 2.19 | 1.30E-04 |
| 15 | 276.2066 | 276.2089 | 8 | 438.2979779 | 9,13-Octadecadiynoic acid | C18H28O2 |  | 2.19 | 1.30E-04 |
| 15 | 276.2066 | 276.2089 | 8 | 438.2979779 | 10,12-Octadecadiynoic acid | C18H28O2 |  | 2.19 | 1.30E-04 |
| 15 | 276.2066 | 276.2089 | 8 | 438.2979779 | 10,13-Octadecadiynoic acid | C18H28O2 |  | 2.19 | 1.30E-04 |
| 15 | 276.2066 | 276.2089 | 8 | 438.2979779 | 10,14-Octadecadiynoic acid | C18H28O2 |  | 2.19 | 1.30E-04 |
| 15 | 276.2066 | 276.2089 | 8 | 438.2979779 | 11,14-Octadecadiynoic acid | C18H28O2 |  | 2.19 | 1.30E-04 |
| 15 | 276.2066 | 276.2089 | 8 | 438.2979779 | 11,15-Octadecadiynoic acid | C18H28O2 |  | 2.19 | 1.30E-04 |
| 15 | 276.2066 | 276.2089 | 8 | 438.2979779 | 12,14-Octadecadiynoic acid | C18H28O2 |  | 2.19 | 1.30E-04 |
| 15 | 276.2066 | 276.2089 | 8 | 438.2979779 | 12,15-Octadecadiynoic acid | C18H28O2 |  | 2.19 | 1.30E-04 |
| 15 | 276.2066 | 276.2089 | 8 | 438.2979779 | 12,16-Octadecadiynoic acid | C18H28O2 |  | 2.19 | 1.30E-04 |
| 15 | 276.2066 | 276.2089 | 8 | 438.2979779 | 13,16-Octadecadiynoic acid | C18H28O2 |  | 2.19 | 1.30E-04 |
| 15 | 276.2066 | 276.2089 | 8 | 438.2979779 | 13,17-Octadecadiynoic acid | C18H28O2 |  | 2.19 | 1.30E-04 |
| 15 | 276.2066 | 276.2089 | 8 | 438.2979779 | 14,17-Octadecadiynoic acid | C18H28O2 |  | 2.19 | 1.30E-04 |
| 15 | 276.2066 | 276.2089 | 8 | 438.2979779 | (9Z,12Z)-octadeca-9,12-dien-6-ynoic acid | C18H28O2 |  | 2.19 | 1.30E-04 |
| 15 | 276.2066 | 276.2089 | 8 | 438.2979779 | (9Z,14Z)-octadeca-9,14-dien-6-ynoic acid | C18H28O2 |  | 2.19 | 1.30E-04 |
| 15 | 276.2066 | 276.2089 | 8 | 438.2979779 | 6-[3]-ladderane-hexanoic acid | C18H28O2 |  | 2.19 | 1.30E-04 |
| 15 | 276.2066 | 276.2089 | 8 | 438.2979779 | 4,8,12,15-Octadecatetraenoic acid | C18H28O2 |  | 2.19 | 1.30E-04 |
| 16 | 316.1997 | 316.2038 | 13 | 478.2910016 | 19-Hydroky-all-trans-retinoic acid | C20H28O3 |  | 2.24 | 4.92E-05 |
| 16 | 316.1997 | 316.2038 | 13 | 478.2910016 | 19-Hydroxy-13-cis-retinoic acid | C20H28O3 |  | 2.24 | 4.92E-05 |
| 16 | 316.1997 | 316.2038 | 13 | 478.2910016 | TOTAROL-19-CARBOXYLIC ACID | C20H28O3 |  | 2.24 | 4.92E-05 |
| 16 | 316.1997 | 316.2038 | 13 | 478.2910016 | 5,6-Epoxyretinoic acid | C20H28O3 | C16680 | 2.24 | 4.92E-05 |
| 16 | 316.1997 | 316.2038 | 13 | 478.2910016 | all-trans-4-hydroxyretinoic acid | C20H28O3 | C16677 | 2.24 | 4.92E-05 |
| 16 | 316.1997 | 316.2038 | 13 | 478.2910016 | all-trans-18-Hydroxyretinoic acid | C20H28O3 | C16679 | 2.24 | 4.92E-05 |
| 16 | 316.1997 | 316.2038 | 13 | 478.2910016 | Pisiferic acid | C20H28O3 | C09163 | 2.24 | 4.92E-05 |
| 16 | 316.1997 | 316.2038 | 13 | 478.2910016 | ent-17-Oxo-15-kauren-19-oic acid | C20H28O3 |  | 2.24 | 4.92E-05 |
| 16 | 316.1997 | 316.2038 | 13 | 478.2910016 | ent-7-Oxo-8(14),15-pimaradien-19-oic acid | C20H28O3 |  | 2.24 | 4.92E-05 |
| 16 | 316.1997 | 316.2038 | 13 | 478.2910016 | ent-15-Oxo-16-kauren-19-oic acid | C20H28O3 |  | 2.24 | 4.92E-05 |
| 16 | 316.1997 | 316.2038 | 13 | 478.2910016 | 7-Oxo-8,15-isopimaradien-18-oic acid | C20H28O3 |  | 2.24 | 4.92E-05 |
| 16 | 316.1997 | 316.2038 | 13 | 478.2910016 | ent-15,16-Epoxy-1(10),13(16),14-halimatrien-19-oic acid | C20H28O3 |  | 2.24 | 4.92E-05 |
| 17 | 242.1869 | 242.1882 | 5 | 404.2782333 | 4-keto myristic acid | C14H26O3 |  | 6.00 | 6.01E-06 |
| 17 | 242.1869 | 242.1882 | 5 | 404.2782333 | 10-keto myristic acid | C14H26O3 |  | 6.00 | 6.01E-06 |
| 17 | 242.1869 | 242.1882 | 5 | 404.2782333 | 13-keto myristic acid | C14H26O3 |  | 6.00 | 6.01E-06 |
| 17 | 242.1869 | 242.1882 | 5 | 404.2782333 | 7-Oxotetradecanoic acid | C14H26O3 |  | 6.00 | 6.01E-06 |
| 17 | 242.1869 | 242.1882 | 5 | 404.2782333 | 14-hydroxy-12-tetradecenoic acid | C14H26O3 |  | 6.00 | 6.01E-06 |
| 17 | 242.1869 | 242.1882 | 5 | 404.2782333 | 14-hydroxy-12Z-tetradecenoic acid | C14H26O3 |  | 6.00 | 6.01E-06 |
| 17 | 242.1869 | 242.1882 | 5 | 404.2782333 | 12-hydroxy-13-tetradecenoic acid | C14H26O3 |  | 6.00 | 6.01E-06 |
| 17 | 242.1869 | 242.1882 | 5 | 404.2782333 | 14-hydroxy-5Z-tetradecenoic acid | C14H26O3 |  | 6.00 | 6.01E-06 |
| 17 | 242.1869 | 242.1882 | 5 | 404.2782333 | 12-methyl-10-oxo-tridecanoic acid | C14H26O3 |  | 6.00 | 6.01E-06 |
| 17 | 242.1869 | 242.1882 | 5 | 404.2782333 | 2-oxo-tetradecanoic acid | C14H26O3 |  | 6.00 | 6.01E-06 |
| 17 | 242.1869 | 242.1882 | 5 | 404.2782333 | 3-oxo-tetradecanoic acid | C14H26O3 |  | 6.00 | 6.01E-06 |
| 17 | 242.1869 | 242.1882 | 5 | 404.2782333 | 6-oxo-tetradecanoic acid | C14H26O3 |  | 6.00 | 6.01E-06 |
| 18 | 268.2027 | 268.2038 | 4 | 430.2940095 | Methoprene acid | C16H28O3 |  | 4.54 | 3.20E-06 |
| 18 | 268.2027 | 268.2038 | 4 | 430.2940095 | (1R,2R)-3-oxo-2-pentyl-cyclopentanehexanoic acid | C16H28O3 |  | 4.54 | 3.20E-06 |
| 18 | 268.2027 | 268.2038 | 4 | 430.2940095 | (1S,2S)-3-oxo-2-pentyl-cyclopentanehexanoic acid | C16H28O3 |  | 4.54 | 3.20E-06 |
| 19 | 278.2228 | 278.2246 | 6 | 440.3141742 | 3E,9Z,12Z-octadecatrienoic acid | C18H30O2 |  | 3.56 | 9.33E-06 |
| 19 | 278.2228 | 278.2246 | 6 | 440.3141742 | 6,10,14-octadecatrienoic acid | C18H30O2 |  | 3.56 | 9.33E-06 |
| 19 | 278.2228 | 278.2246 | 6 | 440.3141742 | 8Z,10E,12Z-octadecatrienoic acid | C18H30O2 |  | 3.56 | 9.33E-06 |
| 19 | 278.2228 | 278.2246 | 6 | 440.3141742 | &alpha;-calendic acid | C18H30O2 |  | 3.56 | 9.33E-06 |
| 19 | 278.2228 | 278.2246 | 6 | 440.3141742 | &beta;-calendic acid | C18H30O2 |  | 3.56 | 9.33E-06 |
| 19 | 278.2228 | 278.2246 | 6 | 440.3141742 | &beta;-eleostearic acid | C18H30O2 |  | 3.56 | 9.33E-06 |
| 19 | 278.2228 | 278.2246 | 6 | 440.3141742 | 9,12,14-octadecatrienoic acid | C18H30O2 |  | 3.56 | 9.33E-06 |
| 19 | 278.2228 | 278.2246 | 6 | 440.3141742 | 10,12,15-octadecatrienoic acid | C18H30O2 |  | 3.56 | 9.33E-06 |
| 19 | 278.2228 | 278.2246 | 6 | 440.3141742 | 2E,9Z,12Z-octadecatrienoic acid | C18H30O2 |  | 3.56 | 9.33E-06 |
| 19 | 278.2228 | 278.2246 | 6 | 440.3141742 | octadeca-5S,6,16E-trienoic acid | C18H30O2 |  | 3.56 | 9.33E-06 |
| 19 | 278.2228 | 278.2246 | 6 | 440.3141742 | 5,8,11-octadecatrienoic acid | C18H30O2 |  | 3.56 | 9.33E-06 |
| 19 | 278.2228 | 278.2246 | 6 | 440.3141742 | 5,9,12-octadecatrienoic acid | C18H30O2 |  | 3.56 | 9.33E-06 |
| 19 | 278.2228 | 278.2246 | 6 | 440.3141742 | 5Z,9Z,12E-octadecatrienoic acid | C18H30O2 |  | 3.56 | 9.33E-06 |
| 19 | 278.2228 | 278.2246 | 6 | 440.3141742 | Pinolenic Acid | C18H30O2 |  | 3.56 | 9.33E-06 |
| 19 | 278.2228 | 278.2246 | 6 | 440.3141742 | 7E,9Z,12Z-octadecatrienoic acid | C18H30O2 |  | 3.56 | 9.33E-06 |
| 19 | 278.2228 | 278.2246 | 6 | 440.3141742 | 7Z,9Z,12Z-octadecatrienoic acid | C18H30O2 |  | 3.56 | 9.33E-06 |
| 19 | 278.2228 | 278.2246 | 6 | 440.3141742 | 9E,11Z,13Z-octadecatrienoic acid | C18H30O2 |  | 3.56 | 9.33E-06 |
| 19 | 278.2228 | 278.2246 | 6 | 440.3141742 | 9E,12E,15Z-octadecatrienoic acid | C18H30O2 |  | 3.56 | 9.33E-06 |
| 19 | 278.2228 | 278.2246 | 6 | 440.3141742 | 9E,12Z,15E-octadecatrienoic acid | C18H30O2 |  | 3.56 | 9.33E-06 |
| 19 | 278.2228 | 278.2246 | 6 | 440.3141742 | 9E,12Z,15Z-octadecatrienoic acid | C18H30O2 |  | 3.56 | 9.33E-06 |
| 19 | 278.2228 | 278.2246 | 6 | 440.3141742 | 9Z,12E,15E-octadecatrienoic acid | C18H30O2 |  | 3.56 | 9.33E-06 |
| 19 | 278.2228 | 278.2246 | 6 | 440.3141742 | 9Z,12E,15Z-octadecatrienoic acid | C18H30O2 |  | 3.56 | 9.33E-06 |
| 19 | 278.2228 | 278.2246 | 6 | 440.3141742 | 9Z,12Z,15E-octadecatrienoic acid | C18H30O2 |  | 3.56 | 9.33E-06 |
| 19 | 278.2228 | 278.2246 | 6 | 440.3141742 | 11E-octadecen-9-ynoic acid | C18H30O2 |  | 3.56 | 9.33E-06 |
| 19 | 278.2228 | 278.2246 | 6 | 440.3141742 | 11Z-octadecen-9-ynoic acid | C18H30O2 |  | 3.56 | 9.33E-06 |
| 19 | 278.2228 | 278.2246 | 6 | 440.3141742 | 17-octadecen-9-ynoic acid | C18H30O2 |  | 3.56 | 9.33E-06 |
| 19 | 278.2228 | 278.2246 | 6 | 440.3141742 | 9E-Octadecen-12-ynoic acid | C18H30O2 |  | 3.56 | 9.33E-06 |
| 19 | 278.2228 | 278.2246 | 6 | 440.3141742 | &alpha;-Linolenic Acid | C18H30O2 | C06427 | 3.56 | 9.33E-06 |
| 19 | 278.2228 | 278.2246 | 6 | 440.3141742 | &gamma;-Linolenic Acid | C18H30O2 | C06426 | 3.56 | 9.33E-06 |
| 19 | 278.2228 | 278.2246 | 6 | 440.3141742 | Punicic acid | C18H30O2 | C08364 | 3.56 | 9.33E-06 |
| 19 | 278.2228 | 278.2246 | 6 | 440.3141742 | Elaidolinoleic acid | C18H30O2 | C06427 | 3.56 | 9.33E-06 |
| 19 | 278.2228 | 278.2246 | 6 | 440.3141742 | 9Z-Octadecen-12-ynoic acid | C18H30O2 | C07289 | 3.56 | 9.33E-06 |
| 19 | 278.2228 | 278.2246 | 6 | 440.3141742 | octadeca-9Z,11E,14Z-trienoic acid | C18H30O2 |  | 3.56 | 9.33E-06 |
| 19 | 278.2228 | 278.2246 | 6 | 440.3141742 | octadeca-11E,13E,15Z-trienoic acid | C18H30O2 |  | 3.56 | 9.33E-06 |
| 19 | 278.2228 | 278.2246 | 6 | 440.3141742 | octadeca-9Z,11E,15Z-trienoic acid | C18H30O2 |  | 3.56 | 9.33E-06 |
| 19 | 278.2228 | 278.2246 | 6 | 440.3141742 | Columbinic acid | C18H30O2 |  | 3.56 | 9.33E-06 |
| 19 | 278.2228 | 278.2246 | 6 | 440.3141742 | Gorlic acid | C18H30O2 |  | 3.56 | 9.33E-06 |
| 19 | 278.2228 | 278.2246 | 6 | 440.3141742 | 16-methyl-6Z,9Z,12Z-heptadecatrienoic acid | C18H30O2 |  | 3.56 | 9.33E-06 |
| 19 | 278.2228 | 278.2246 | 6 | 440.3141742 | trans-3, cis-9, cis-12-octadecatrienoic acid; C18:3n-6,9,15 | C18H30O2 |  | 3.56 | 9.33E-06 |
| 19 | 278.2228 | 278.2246 | 6 | 440.3141742 | cis-8, trans-10, cis-12-octadecatrienoic acid; C18:3n-6,8,10 | C18H30O2 |  | 3.56 | 9.33E-06 |
| 19 | 278.2228 | 278.2246 | 6 | 440.3141742 | 5,9,12-octadecatrienoic acid | C18H30O2 |  | 3.56 | 9.33E-06 |
| 19 | 278.2228 | 278.2246 | 6 | 440.3141742 | 5Z,9Z,12E-octadecatrienoic acid | C18H30O2 |  | 3.56 | 9.33E-06 |
| 19 | 278.2228 | 278.2246 | 6 | 440.3141742 | 7E,9Z,12Z-octadecatrienoic acid | C18H30O2 |  | 3.56 | 9.33E-06 |
| 19 | 278.2228 | 278.2246 | 6 | 440.3141742 | 7Z,9Z,12Z-octadecatrienoic acid | C18H30O2 |  | 3.56 | 9.33E-06 |
| 19 | 278.2228 | 278.2246 | 6 | 440.3141742 | 9E,12E,15Z-octadecatrienoic acid | C18H30O2 |  | 3.56 | 9.33E-06 |
| 19 | 278.2228 | 278.2246 | 6 | 440.3141742 | 9E,12Z,15E-octadecatrienoic acid | C18H30O2 |  | 3.56 | 9.33E-06 |
| 19 | 278.2228 | 278.2246 | 6 | 440.3141742 | 9E,12Z,15Z-octadecatrienoic acid | C18H30O2 |  | 3.56 | 9.33E-06 |
| 19 | 278.2228 | 278.2246 | 6 | 440.3141742 | 9Z,12E,15E-octadecatrienoic acid | C18H30O2 |  | 3.56 | 9.33E-06 |
| 19 | 278.2228 | 278.2246 | 6 | 440.3141742 | 9Z,12E,15Z-octadecatrienoic acid | C18H30O2 |  | 3.56 | 9.33E-06 |
| 19 | 278.2228 | 278.2246 | 6 | 440.3141742 | 9Z,12Z,15E-octadecatrienoic acid | C18H30O2 |  | 3.56 | 9.33E-06 |
| 19 | 278.2228 | 278.2246 | 6 | 440.3141742 | Acetylenic acids; 11-Octadecen-9-ynoic acid, (E)-; Ximenynic acid; Santalbic acid; trans-11-Octadecen-9-ynoic acid; Ximeninic acid | C18H30O2 |  | 3.56 | 9.33E-06 |
| 19 | 278.2228 | 278.2246 | 6 | 440.3141742 | Acetylenic acids; 11-Octadecen-9-ynoic acid, (Z)-; cis-11-Octadecen-9-ynoic acid | C18H30O2 |  | 3.56 | 9.33E-06 |
| 19 | 278.2228 | 278.2246 | 6 | 440.3141742 | Acetylenic acids; 17-Octadecen-9-ynoic acid | C18H30O2 |  | 3.56 | 9.33E-06 |
| 19 | 278.2228 | 278.2246 | 6 | 440.3141742 | (E)-octadec-9-en-12-ynoic acid | C18H30O2 |  | 3.56 | 9.33E-06 |
| 19 | 278.2228 | 278.2246 | 6 | 440.3141742 | Crepenynic acid | C18H30O2 |  | 3.56 | 9.33E-06 |
| 19 | 278.2228 | 278.2246 | 6 | 440.3141742 | (R)-lamenallenic acid | C18H30O2 |  | 3.56 | 9.33E-06 |
| 19 | 278.2228 | 278.2246 | 6 | 440.3141742 | (S)-lamenallenic acid | C18H30O2 |  | 3.56 | 9.33E-06 |
| 19 | 278.2228 | 278.2246 | 6 | 440.3141742 | Catalpic acid | C18H30O2 |  | 3.56 | 9.33E-06 |
| 19 | 278.2228 | 278.2246 | 6 | 440.3141742 | 10,12,14-octadecatrienoic acid | C18H30O2 |  | 3.56 | 9.33E-06 |
| 19 | 278.2228 | 278.2246 | 6 | 440.3141742 | Pseudoeleostearic acid | C18H30O2 |  | 3.56 | 9.33E-06 |
| 20 | 334.2102 | 334.2144 | 12 | 496.3014918 | 20-carboxy Arachidonic Acid | C20H30O4 |  | 4.96 | 2.96E-04 |
| 20 | 334.2102 | 334.2144 | 12 | 496.3014918 | 5,12-dihydroxy-6,8,10,14,17-eicosapentaenoic acid | C20H30O4 |  | 4.96 | 2.96E-04 |
| 20 | 334.2102 | 334.2144 | 12 | 496.3014918 | (-)-Cassaic acid | C20H30O4 |  | 4.96 | 2.96E-04 |
| 20 | 334.2102 | 334.2144 | 12 | 496.3014918 | (-)-8,16-Dihydroxy-19-serrulatanoic acid | C20H30O4 |  | 4.96 | 2.96E-04 |
| 20 | 334.2102 | 334.2144 | 12 | 496.3014918 | 6&beta;,7&beta;-Dihydroxykaurenoic acid | C20H30O4 | C11876 | 4.96 | 2.96E-04 |
| 20 | 334.2102 | 334.2144 | 12 | 496.3014918 | 17-Oxogrindelic acid | C20H30O4 | C09150 | 4.96 | 2.96E-04 |
| 20 | 334.2102 | 334.2144 | 12 | 496.3014918 | 3beta,13-Dihydroxy-16-(hydroxymethylene)-13,17-seco-5alpha-androstan-17-oic acid, delta-lactone | C20H30O4 | C15159 | 4.96 | 2.96E-04 |
| 20 | 334.2102 | 334.2144 | 12 | 496.3014918 | 5Z,8Z,11Z,14Z-Eicosatetraenedioic acid | C20H30O4 |  | 4.96 | 2.96E-04 |
| 20 | 334.2102 | 334.2144 | 12 | 496.3014918 | 8alpha-8-Hydroxy-12-oxo-13-abieten-18-oic acid | C20H30O4 |  | 4.96 | 2.96E-04 |
| 20 | 334.2102 | 334.2144 | 12 | 496.3014918 | (ent-16betaOH)-16,17-Dihydroxy-9(11)-kauren-19-oic acid | C20H30O4 |  | 4.96 | 2.96E-04 |
| 20 | 334.2102 | 334.2144 | 12 | 496.3014918 | Dehydropinifolic acid | C20H30O4 |  | 4.96 | 2.96E-04 |
| 21 | 318.2159 | 318.2195 | 11 | 480.3072089 | 12-oxo-5Z,8Z,10E,14Z-eicosatetraenoic acid | C20H30O3 |  | 4.16 | 7.78E-06 |
| 21 | 318.2159 | 318.2195 | 11 | 480.3072089 | 8-oxo-5E,9Z,11Z,14Z-eicosatetraenoic acid | C20H30O3 |  | 4.16 | 7.78E-06 |
| 21 | 318.2159 | 318.2195 | 11 | 480.3072089 | 9-oxo-5E,7Z,11Z,14Z-eicosatetraenoic acid | C20H30O3 |  | 4.16 | 7.78E-06 |
| 21 | 318.2159 | 318.2195 | 11 | 480.3072089 | 11-oxo-5E,8Z,12Z,14Z-Eicosatetraenoic acid | C20H30O3 |  | 4.16 | 7.78E-06 |
| 21 | 318.2159 | 318.2195 | 11 | 480.3072089 | 5-Ketoeicosatetraenoic acid | C20H30O3 |  | 4.16 | 7.78E-06 |
| 21 | 318.2159 | 318.2195 | 11 | 480.3072089 | (+)-7beta-Hydroxy-15-beyeren-19-oic acid | C20H30O3 |  | 4.16 | 7.78E-06 |
| 21 | 318.2159 | 318.2195 | 11 | 480.3072089 | ent-7alpha-hydroxykaur-16-en-19-oic acid | C20H30O3 |  | 4.16 | 7.78E-06 |
| 21 | 318.2159 | 318.2195 | 11 | 480.3072089 | ent-7&alpha;-Hydroxykaur-16-en-19-oic acid | C20H30O3 | C11875 | 4.16 | 7.78E-06 |
| 21 | 318.2159 | 318.2195 | 11 | 480.3072089 | Grandifloric acid | C20H30O3 | C17956 | 4.16 | 7.78E-06 |
| 21 | 318.2159 | 318.2195 | 11 | 480.3072089 | 9-hydroxy-2Z,5E,7Z,11Z,14Z-Eicosapentaenoic acid | C20H30O3 |  | 4.16 | 7.78E-06 |
| 21 | 318.2159 | 318.2195 | 11 | 480.3072089 | 5,9,11,14-Eicosatetraenoic acid, 8-oxo-, (E,Z,Z,Z)- | C20H30O3 |  | 4.16 | 7.78E-06 |
| 21 | 318.2159 | 318.2195 | 11 | 480.3072089 | 5,7,11,14-Eicosatetraenoic acid, 9-oxo-, (E,Z,Z,Z)- | C20H30O3 |  | 4.16 | 7.78E-06 |
| 21 | 318.2159 | 318.2195 | 11 | 480.3072089 | 5,8,12,14-Eicosatetraenoic acid, 11-oxo-, (E,Z,Z,Z)- | C20H30O3 |  | 4.16 | 7.78E-06 |
| 21 | 318.2159 | 318.2195 | 11 | 480.3072089 | (ent-7alpha)-7-Hydroxy-8(14),15-pimaradien-19-oic acid | C20H30O3 |  | 4.16 | 7.78E-06 |
| 21 | 318.2159 | 318.2195 | 11 | 480.3072089 | ent-17-Hydroxy-15-kauren-19-oic acid | C20H30O3 |  | 4.16 | 7.78E-06 |
| 21 | 318.2159 | 318.2195 | 11 | 480.3072089 | (ent-15beta)-15-Hydroxy-19-trachylobanoic acid | C20H30O3 |  | 4.16 | 7.78E-06 |
| 21 | 318.2159 | 318.2195 | 11 | 480.3072089 | 2-Hydroxy-6-(8-tridecenyl)benzoic acid | C20H30O3 |  | 4.16 | 7.78E-06 |
| 21 | 318.2159 | 318.2195 | 11 | 480.3072089 | 12alpha-12-Hydroxy-7,13-abietadien-18-oic acid | C20H30O3 |  | 4.16 | 7.78E-06 |
| 22 | 198.1607 | 198.1620 | 6 | 360.2520424 | 2-lauroleic acid | C12H22O2 |  | 2.78 | 1.75E-05 |
| 22 | 198.1607 | 198.1620 | 6 | 360.2520424 | Linderic acid | C12H22O2 |  | 2.78 | 1.75E-05 |
| 22 | 198.1607 | 198.1620 | 6 | 360.2520424 | 6-lauroleic acid | C12H22O2 |  | 2.78 | 1.75E-05 |
| 22 | 198.1607 | 198.1620 | 6 | 360.2520424 | 7-lauroleic acid | C12H22O2 |  | 2.78 | 1.75E-05 |
| 22 | 198.1607 | 198.1620 | 6 | 360.2520424 | 9-lauroleic acid | C12H22O2 |  | 2.78 | 1.75E-05 |
| 22 | 198.1607 | 198.1620 | 6 | 360.2520424 | 10-Lauroleic acid | C12H22O2 |  | 2.78 | 1.75E-05 |
| 22 | 198.1607 | 198.1620 | 6 | 360.2520424 | 11-lauroleic acid | C12H22O2 |  | 2.78 | 1.75E-05 |
| 22 | 198.1607 | 198.1620 | 6 | 360.2520424 | 10Z-dodecenoic acid | C12H22O2 |  | 2.78 | 1.75E-05 |
| 22 | 198.1607 | 198.1620 | 6 | 360.2520424 | 2Z-dodecenoic acid | C12H22O2 |  | 2.78 | 1.75E-05 |
| 22 | 198.1607 | 198.1620 | 6 | 360.2520424 | cis-5-dodecenoic acid | C12H22O2 |  | 2.78 | 1.75E-05 |
| 22 | 198.1607 | 198.1620 | 6 | 360.2520424 | 6Z-dodecenoic acid | C12H22O2 |  | 2.78 | 1.75E-05 |
| 22 | 198.1607 | 198.1620 | 6 | 360.2520424 | 7Z-dodecenoic acid | C12H22O2 |  | 2.78 | 1.75E-05 |
| 22 | 198.1607 | 198.1620 | 6 | 360.2520424 | 9Z-dodecenoic acid | C12H22O2 |  | 2.78 | 1.75E-05 |
| 22 | 198.1607 | 198.1620 | 6 | 360.2520424 | cis-dodec-3-enoic acid | C12H22O2 |  | 2.78 | 1.75E-05 |
| 22 | 198.1607 | 198.1620 | 6 | 360.2520424 | trans-dodec-3-enoic acid | C12H22O2 |  | 2.78 | 1.75E-05 |
| 22 | 198.1607 | 198.1620 | 6 | 360.2520424 | 2E-Lauroleic acid | C12H22O2 |  | 2.78 | 1.75E-05 |
| 22 | 198.1607 | 198.1620 | 6 | 360.2520424 | 5-methyl-2-undecenoic acid | C12H22O2 |  | 2.78 | 1.75E-05 |
| 23 | 294.2159 | 294.2195 | 12 | 456.3072888 | alpha-kamlolenic acid | C18H30O3 |  | 2.14 | 2.22E-05 |
| 23 | 294.2159 | 294.2195 | 12 | 456.3072888 | beta-kamlolenic acid | C18H30O3 |  | 2.14 | 2.22E-05 |
| 23 | 294.2159 | 294.2195 | 12 | 456.3072888 | 2-hydroxy-9Z,12Z,15Z-octadecatrienoic acid | C18H30O3 |  | 2.14 | 2.22E-05 |
| 23 | 294.2159 | 294.2195 | 12 | 456.3072888 | 9-oxo-10,12-octadecadienoic acid | C18H30O3 |  | 2.14 | 2.22E-05 |
| 23 | 294.2159 | 294.2195 | 12 | 456.3072888 | (9E,11E)-13-oxooctadeca-9,11-dienoic acid | C18H30O3 |  | 2.14 | 2.22E-05 |
| 23 | 294.2159 | 294.2195 | 12 | 456.3072888 | (9R,13R)-10-oxo-11-phytoenoic acid | C18H30O3 |  | 2.14 | 2.22E-05 |
| 23 | 294.2159 | 294.2195 | 12 | 456.3072888 | (9S,13S)-10-oxo-11-phytoenoic acid | C18H30O3 |  | 2.14 | 2.22E-05 |
| 23 | 294.2159 | 294.2195 | 12 | 456.3072888 | (9R,13R)-15,16-dihydro-12-oxo-10-phytoenoic acid | C18H30O3 |  | 2.14 | 2.22E-05 |
| 23 | 294.2159 | 294.2195 | 12 | 456.3072888 | (9S,13S)-15,16-dihydro-12-oxo-10-phytoenoic acid | C18H30O3 |  | 2.14 | 2.22E-05 |
| 23 | 294.2159 | 294.2195 | 12 | 456.3072888 | (9R,13R)-10,11-dihydro-12-oxo-15-phytoenoic acid | C18H30O3 |  | 2.14 | 2.22E-05 |
| 23 | 294.2159 | 294.2195 | 12 | 456.3072888 | (9S,13S)-10,11-dihydro-12-oxo-15-phytoenoic acid | C18H30O3 | C04780 | 2.14 | 2.22E-05 |
| 23 | 294.2159 | 294.2195 | 12 | 456.3072888 | Colneleic acid | C18H30O3 | C19827 | 2.14 | 2.22E-05 |
| 23 | 294.2159 | 294.2195 | 12 | 456.3072888 | 17-hydroxy-linolenic acid | C18H30O3 |  | 2.14 | 2.22E-05 |
| 23 | 294.2159 | 294.2195 | 12 | 456.3072888 | 17-Hydroxylinolenic acid | C18H30O3 | C16346 | 2.14 | 2.22E-05 |
| 23 | 294.2159 | 294.2195 | 12 | 456.3072888 | 8-hydroxy-11Z-octadecen-9-ynoic acid | C18H30O3 |  | 2.14 | 2.22E-05 |
| 23 | 294.2159 | 294.2195 | 12 | 456.3072888 | 9-hydroxy-10E-octadecen-12-ynoic acid | C18H30O3 |  | 2.14 | 2.22E-05 |
| 23 | 294.2159 | 294.2195 | 12 | 456.3072888 | 12-oxo-9-octadecynoic acid | C18H30O3 |  | 2.14 | 2.22E-05 |
| 23 | 294.2159 | 294.2195 | 12 | 456.3072888 | 8-(5-hexyl-furan-2-yl)-octanoic acid | C18H30O3 |  | 2.14 | 2.22E-05 |
| 23 | 294.2159 | 294.2195 | 12 | 456.3072888 | 9S,10-epoxy-10,12Z-octadecadienoic acid | C18H30O3 |  | 2.14 | 2.22E-05 |
| 23 | 294.2159 | 294.2195 | 12 | 456.3072888 | 2R-hydroxy-9Z,12Z,15Z-octadecatrienoic acid | C18H30O3 |  | 2.14 | 2.22E-05 |
| 23 | 294.2159 | 294.2195 | 12 | 456.3072888 | 15,16-Epoxy-9,12-octadecadienoic acid | C18H30O3 |  | 2.14 | 2.22E-05 |
| 23 | 294.2159 | 294.2195 | 12 | 456.3072888 | 12,13-Epoxy-9,15-octadecadienoic acid | C18H30O3 |  | 2.14 | 2.22E-05 |
| 23 | 294.2159 | 294.2195 | 12 | 456.3072888 | 2-Hydroxylinolenic acid | C18H30O3 |  | 2.14 | 2.22E-05 |
| 23 | 294.2159 | 294.2195 | 12 | 456.3072888 | (9Z,12Z,14E)-16-Hydroxy-9,12,14-octadecatrienoic acid | C18H30O3 |  | 2.14 | 2.22E-05 |
| 23 | 294.2159 | 294.2195 | 12 | 456.3072888 | 3,4-Dimethyl-5-pentyl-2-furanheptanoic acid | C18H30O3 |  | 2.14 | 2.22E-05 |
| 24 | 298.2477 | 298.2508 | 10 | 460.339038 | 9-hydroxy-12Z-octadecenoic acid | C18H34O3 |  | 5.03 | 1.52E-05 |
| 24 | 298.2477 | 298.2508 | 10 | 460.339038 | Ricinoleic acid | C18H34O3 |  | 5.03 | 1.52E-05 |
| 24 | 298.2477 | 298.2508 | 10 | 460.339038 | Ricinelaidic acid | C18H34O3 |  | 5.03 | 1.52E-05 |
| 24 | 298.2477 | 298.2508 | 10 | 460.339038 | 12-hydroxy-10E-octadecenoic acid | C18H34O3 |  | 5.03 | 1.52E-05 |
| 24 | 298.2477 | 298.2508 | 10 | 460.339038 | 9-hydroxy-10Z-octadecenoic acid | C18H34O3 |  | 5.03 | 1.52E-05 |
| 24 | 298.2477 | 298.2508 | 10 | 460.339038 | 9-hydroxy-12-octadecenoic acid | C18H34O3 |  | 5.03 | 1.52E-05 |
| 24 | 298.2477 | 298.2508 | 10 | 460.339038 | 17-hydroxy-9Z-octadecenoic acid | C18H34O3 |  | 5.03 | 1.52E-05 |
| 24 | 298.2477 | 298.2508 | 10 | 460.339038 | 5-hydroxy-2-octadecenoic acid | C18H34O3 |  | 5.03 | 1.52E-05 |
| 24 | 298.2477 | 298.2508 | 10 | 460.339038 | 8-hydroxy-9-octadecenoic acid | C18H34O3 |  | 5.03 | 1.52E-05 |
| 24 | 298.2477 | 298.2508 | 10 | 460.339038 | 8R-hydroxy-9Z-octadecenoic acid | C18H34O3 |  | 5.03 | 1.52E-05 |
| 24 | 298.2477 | 298.2508 | 10 | 460.339038 | 9R-hydroxy-10E-octadecenoic acid | C18H34O3 |  | 5.03 | 1.52E-05 |
| 24 | 298.2477 | 298.2508 | 10 | 460.339038 | 9-hydroxy-10E-octadecenoic acid | C18H34O3 |  | 5.03 | 1.52E-05 |
| 24 | 298.2477 | 298.2508 | 10 | 460.339038 | 9R-hydroxy-12E-octadecenoic acid | C18H34O3 |  | 5.03 | 1.52E-05 |
| 24 | 298.2477 | 298.2508 | 10 | 460.339038 | 9R-hydroxy-12Z-octadecenoic acid | C18H34O3 |  | 5.03 | 1.52E-05 |
| 24 | 298.2477 | 298.2508 | 10 | 460.339038 | 10-hydroxy-8-octadecenoic acid | C18H34O3 |  | 5.03 | 1.52E-05 |
| 24 | 298.2477 | 298.2508 | 10 | 460.339038 | 10R-hydroxy-8E-octadecenoic acid | C18H34O3 |  | 5.03 | 1.52E-05 |
| 24 | 298.2477 | 298.2508 | 10 | 460.339038 | 11-hydroxy-9-octadecenoic acid | C18H34O3 |  | 5.03 | 1.52E-05 |
| 24 | 298.2477 | 298.2508 | 10 | 460.339038 | 12R-hydroxy-9E-octadecenoic acid | C18H34O3 |  | 5.03 | 1.52E-05 |
| 24 | 298.2477 | 298.2508 | 10 | 460.339038 | 12S-hydroxy-9E-octadecenoic acid | C18H34O3 |  | 5.03 | 1.52E-05 |
| 24 | 298.2477 | 298.2508 | 10 | 460.339038 | 12S-hydroxy-9Z-octadecenoic acid | C18H34O3 |  | 5.03 | 1.52E-05 |
| 24 | 298.2477 | 298.2508 | 10 | 460.339038 | 3-keto stearic acid | C18H34O3 |  | 5.03 | 1.52E-05 |
| 24 | 298.2477 | 298.2508 | 10 | 460.339038 | 4-keto stearic acid | C18H34O3 |  | 5.03 | 1.52E-05 |
| 24 | 298.2477 | 298.2508 | 10 | 460.339038 | 5-keto stearic acid | C18H34O3 |  | 5.03 | 1.52E-05 |
| 24 | 298.2477 | 298.2508 | 10 | 460.339038 | 6-keto stearic acid | C18H34O3 |  | 5.03 | 1.52E-05 |
| 24 | 298.2477 | 298.2508 | 10 | 460.339038 | 7-keto-stearic acid | C18H34O3 |  | 5.03 | 1.52E-05 |
| 24 | 298.2477 | 298.2508 | 10 | 460.339038 | 9-keto stearic acid | C18H34O3 |  | 5.03 | 1.52E-05 |
| 24 | 298.2477 | 298.2508 | 10 | 460.339038 | 10-keto stearic acid | C18H34O3 |  | 5.03 | 1.52E-05 |
| 24 | 298.2477 | 298.2508 | 10 | 460.339038 | 9R,10S-epoxy-stearic acid | C18H34O3 |  | 5.03 | 1.52E-05 |
| 24 | 298.2477 | 298.2508 | 10 | 460.339038 | 18-hydroxy-9Z-octadecenoic acid | C18H34O3 | C19616 | 5.03 | 1.52E-05 |
| 24 | 298.2477 | 298.2508 | 10 | 460.339038 | 12R-hydroxy-9Z-octadecenoic acid | C18H34O3 | C08365 | 5.03 | 1.52E-05 |
| 24 | 298.2477 | 298.2508 | 10 | 460.339038 | cis-9,10-Epoxystearic acid | C18H34O3 | C19418 | 5.03 | 1.52E-05 |
| 24 | 298.2477 | 298.2508 | 10 | 460.339038 | 3S,7,11-Trimethyl-6,10-dodecadienoic acid | C18H34O3 |  | 5.03 | 1.52E-05 |
| 24 | 298.2477 | 298.2508 | 10 | 460.339038 | 2R-hydroxy-oleic acid | C18H34O3 |  | 5.03 | 1.52E-05 |
| 24 | 298.2477 | 298.2508 | 10 | 460.339038 | Isoricinoleic Acid | C18H34O3 |  | 5.03 | 1.52E-05 |
| 24 | 298.2477 | 298.2508 | 10 | 460.339038 | 16-methyl-10-oxo-heptadecanoic acid | C18H34O3 |  | 5.03 | 1.52E-05 |
| 24 | 298.2477 | 298.2508 | 10 | 460.339038 | 2-methyl-4-oxo-heptadecanoic acid | C18H34O3 |  | 5.03 | 1.52E-05 |
| 24 | 298.2477 | 298.2508 | 10 | 460.339038 | 11-oxo-octadecanoic acid | C18H34O3 |  | 5.03 | 1.52E-05 |
| 24 | 298.2477 | 298.2508 | 10 | 460.339038 | 12-oxo-octadecanoic acid | C18H34O3 |  | 5.03 | 1.52E-05 |
| 24 | 298.2477 | 298.2508 | 10 | 460.339038 | 13-oxo-octadecanoic acid | C18H34O3 |  | 5.03 | 1.52E-05 |
| 24 | 298.2477 | 298.2508 | 10 | 460.339038 | 14-oxo-octadecanoic acid | C18H34O3 |  | 5.03 | 1.52E-05 |
| 24 | 298.2477 | 298.2508 | 10 | 460.339038 | 15-oxo-octadecanoic acid | C18H34O3 |  | 5.03 | 1.52E-05 |
| 24 | 298.2477 | 298.2508 | 10 | 460.339038 | 16-oxo-octadecanoic acid | C18H34O3 |  | 5.03 | 1.52E-05 |
| 24 | 298.2477 | 298.2508 | 10 | 460.339038 | 17-oxo-octadecanoic acid | C18H34O3 |  | 5.03 | 1.52E-05 |
| 24 | 298.2477 | 298.2508 | 10 | 460.339038 | 2-oxo-octadecanoic acid | C18H34O3 |  | 5.03 | 1.52E-05 |
| 24 | 298.2477 | 298.2508 | 10 | 460.339038 | 8-oxo-octadecanoic acid | C18H34O3 |  | 5.03 | 1.52E-05 |
| 24 | 298.2477 | 298.2508 | 10 | 460.339038 | 6R,7S-epoxy-octadecanoic acid | C18H34O3 |  | 5.03 | 1.52E-05 |
| 24 | 298.2477 | 298.2508 | 10 | 460.339038 | 5-Hexyltetrahydro-2-furanoctanoic acid | C18H34O3 |  | 5.03 | 1.52E-05 |
| 24 | 298.2477 | 298.2508 | 10 | 460.339038 | 8Z-decen-4,6-diynoic acid | C18H34O3 |  | 5.03 | 1.52E-05 |
| 25 | 248.1769 | 248.1776 | 2 | 410.2682175 | 4,7,10,13-hexadecatetraenoic acid | C16H24O2 |  | 6.18 | 6.60E-07 |
| 25 | 248.1769 | 248.1776 | 2 | 410.2682175 | 4,7,11,14-hexadecatetraenoic acid | C16H24O2 |  | 6.18 | 6.60E-07 |
| 25 | 248.1769 | 248.1776 | 2 | 410.2682175 | 4,8,12,16-hexadecatetraenoic acid | C16H24O2 |  | 6.18 | 6.60E-07 |
| 25 | 248.1769 | 248.1776 | 2 | 410.2682175 | 6,9,12,15-hexadecatetraenoic acid | C16H24O2 |  | 6.18 | 6.60E-07 |
| 25 | 248.1769 | 248.1776 | 2 | 410.2682175 | 2E,6Z,8Z,12E-hexadecatetraenoic acid | C16H24O2 |  | 6.18 | 6.60E-07 |
| 25 | 248.1769 | 248.1776 | 2 | 410.2682175 | 2E,6Z,8Z,12Z-hexadecatetraenoic acid | C16H24O2 |  | 6.18 | 6.60E-07 |
| 25 | 248.1769 | 248.1776 | 2 | 410.2682175 | 2Z,6Z,8Z,12E-hexadecatetraenoic acid | C16H24O2 |  | 6.18 | 6.60E-07 |
| 25 | 248.1769 | 248.1776 | 2 | 410.2682175 | 2Z,6Z,8Z,12Z-hexadecatetraenoic acid | C16H24O2 |  | 6.18 | 6.60E-07 |
| 25 | 248.1769 | 248.1776 | 2 | 410.2682175 | 4Z,7Z,10Z,13Z-hexadecatetraenoic acid | C16H24O2 |  | 6.18 | 6.60E-07 |
| 25 | 248.1769 | 248.1776 | 3 | 410.2682175 | 4-[3]-ladderane-butanoic acid | C16H24O2 |  | 6.18 | 6.60E-07 |
| 25 | 248.1769 | 248.1776 | 3 | 410.2682175 | 2E,6Z,8Z,12E-hexadecatetraenoic acid | C16H24O2 |  | 6.18 | 6.60E-07 |
| 25 | 248.1769 | 248.1776 | 3 | 410.2682175 | 2E,6Z,8Z,12Z-hexadecatetraenoic acid | C16H24O2 |  | 6.18 | 6.60E-07 |
| 25 | 248.1769 | 248.1776 | 3 | 410.2682175 | 2Z,6Z,8Z,12E-hexadecatetraenoic acid | C16H24O2 |  | 6.18 | 6.60E-07 |
| 25 | 248.1769 | 248.1776 | 3 | 410.2682175 | 2Z,6Z,8Z,12Z-hexadecatetraenoic acid | C16H24O2 |  | 6.18 | 6.60E-07 |
| 25 | 248.1769 | 248.1776 | 3 | 410.2682175 | 3,9-Hexadecadiynoic acid | C16H24O2 |  | 6.18 | 6.60E-07 |
| 25 | 248.1769 | 248.1776 | 3 | 410.2682175 | 7,10-Hexadecadiynoic acid | C16H24O2 |  | 6.18 | 6.60E-07 |
| 25 | 248.1769 | 248.1776 | 3 | 410.2682175 | 8,10-Hexadecadiynoic acid | C16H24O2 |  | 6.18 | 6.60E-07 |
| 26 | 280.2372 | 280.2402 | 10 | 442.328577 | 5Z,12Z-otadecadienoic acid | C18H32O2 |  | 5.18 | 4.71E-06 |
| 26 | 280.2372 | 280.2402 | 10 | 442.328577 | 5Z,12E-otadecadienoic acid | C18H32O2 |  | 5.18 | 4.71E-06 |
| 26 | 280.2372 | 280.2402 | 10 | 442.328577 | 5E,12Z-otadecadienoic acid | C18H32O2 |  | 5.18 | 4.71E-06 |
| 26 | 280.2372 | 280.2402 | 10 | 442.328577 | 5E,12E-otadecadienoic acid | C18H32O2 |  | 5.18 | 4.71E-06 |
| 26 | 280.2372 | 280.2402 | 10 | 442.328577 | 6, 8-octadecadienoic acid | C18H32O2 |  | 5.18 | 4.71E-06 |
| 26 | 280.2372 | 280.2402 | 10 | 442.328577 | 8E,10E-octadecadienoic acid | C18H32O2 |  | 5.18 | 4.71E-06 |
| 26 | 280.2372 | 280.2402 | 10 | 442.328577 | 8Z,11Z-octadecadienoic acid | C18H32O2 |  | 5.18 | 4.71E-06 |
| 26 | 280.2372 | 280.2402 | 10 | 442.328577 | 9Z,11Z-octadecadienoic acid | C18H32O2 |  | 5.18 | 4.71E-06 |
| 26 | 280.2372 | 280.2402 | 10 | 442.328577 | 9(E),11(E)-Conjugated Linoleic Acid | C18H32O2 |  | 5.18 | 4.71E-06 |
| 26 | 280.2372 | 280.2402 | 10 | 442.328577 | 9Z,12E-octadecadienoic acid | C18H32O2 |  | 5.18 | 4.71E-06 |
| 26 | 280.2372 | 280.2402 | 10 | 442.328577 | 9E,12Z-octadecadienoic acid | C18H32O2 |  | 5.18 | 4.71E-06 |
| 26 | 280.2372 | 280.2402 | 10 | 442.328577 | 10Z,12Z-octadecadienoic acid | C18H32O2 |  | 5.18 | 4.71E-06 |
| 26 | 280.2372 | 280.2402 | 10 | 442.328577 | 10E,12Z-Octadecadienoic acid | C18H32O2 |  | 5.18 | 4.71E-06 |
| 26 | 280.2372 | 280.2402 | 10 | 442.328577 | 10E,12E-octadecadienoic acid | C18H32O2 |  | 5.18 | 4.71E-06 |
| 26 | 280.2372 | 280.2402 | 10 | 442.328577 | 10Z,13Z-octadecadienoic acid | C18H32O2 |  | 5.18 | 4.71E-06 |
| 26 | 280.2372 | 280.2402 | 10 | 442.328577 | 11E,14Z-octadecadienoic acid | C18H32O2 |  | 5.18 | 4.71E-06 |
| 26 | 280.2372 | 280.2402 | 10 | 442.328577 | 10E,14E-octadecadienoic acid | C18H32O2 |  | 5.18 | 4.71E-06 |
| 26 | 280.2372 | 280.2402 | 10 | 442.328577 | 10Z,14Z-octadecadienoic acid | C18H32O2 |  | 5.18 | 4.71E-06 |
| 26 | 280.2372 | 280.2402 | 10 | 442.328577 | 11Z,14Z-octadecadienoic acid | C18H32O2 |  | 5.18 | 4.71E-06 |
| 26 | 280.2372 | 280.2402 | 10 | 442.328577 | 11Z,15Z-octadecadienoic acid | C18H32O2 |  | 5.18 | 4.71E-06 |
| 26 | 280.2372 | 280.2402 | 10 | 442.328577 | 12E,16E-octadecadienoic acid | C18H32O2 |  | 5.18 | 4.71E-06 |
| 26 | 280.2372 | 280.2402 | 10 | 442.328577 | 12Z,15Z-octadecadienoic acid | C18H32O2 |  | 5.18 | 4.71E-06 |
| 26 | 280.2372 | 280.2402 | 10 | 442.328577 | 13E,17-octadecadienoic acid | C18H32O2 |  | 5.18 | 4.71E-06 |
| 26 | 280.2372 | 280.2402 | 10 | 442.328577 | 13Z,16Z-octadecadienoic acid | C18H32O2 |  | 5.18 | 4.71E-06 |
| 26 | 280.2372 | 280.2402 | 10 | 442.328577 | 14Z,17-octadecadienoic acid | C18H32O2 |  | 5.18 | 4.71E-06 |
| 26 | 280.2372 | 280.2402 | 10 | 442.328577 | 2,4-octadecadienoic acid | C18H32O2 |  | 5.18 | 4.71E-06 |
| 26 | 280.2372 | 280.2402 | 10 | 442.328577 | 2E,6E-octadecadienoic acid | C18H32O2 |  | 5.18 | 4.71E-06 |
| 26 | 280.2372 | 280.2402 | 10 | 442.328577 | 2Z,5Z-octadecadienoic acid | C18H32O2 |  | 5.18 | 4.71E-06 |
| 26 | 280.2372 | 280.2402 | 10 | 442.328577 | 2Z,6Z-octadecadienoic acid | C18H32O2 |  | 5.18 | 4.71E-06 |
| 26 | 280.2372 | 280.2402 | 10 | 442.328577 | 3E,7E-octadecadienoic acid | C18H32O2 |  | 5.18 | 4.71E-06 |
| 26 | 280.2372 | 280.2402 | 10 | 442.328577 | 3Z,12Z-octadecadienoic acid | C18H32O2 |  | 5.18 | 4.71E-06 |
| 26 | 280.2372 | 280.2402 | 10 | 442.328577 | 3Z,6Z-octadecadienoic acid | C18H32O2 |  | 5.18 | 4.71E-06 |
| 26 | 280.2372 | 280.2402 | 10 | 442.328577 | 3Z,7Z-octadecadienoic acid | C18H32O2 |  | 5.18 | 4.71E-06 |
| 26 | 280.2372 | 280.2402 | 10 | 442.328577 | 4,9-octadecadienoic acid | C18H32O2 |  | 5.18 | 4.71E-06 |
| 26 | 280.2372 | 280.2402 | 10 | 442.328577 | 4E,8E-octadecadienoic acid | C18H32O2 |  | 5.18 | 4.71E-06 |
| 26 | 280.2372 | 280.2402 | 10 | 442.328577 | 4Z,7Z-octadecadienoic acid | C18H32O2 |  | 5.18 | 4.71E-06 |
| 26 | 280.2372 | 280.2402 | 10 | 442.328577 | 4Z,8Z-octadecadienoic acid | C18H32O2 |  | 5.18 | 4.71E-06 |
| 26 | 280.2372 | 280.2402 | 10 | 442.328577 | 5,10-octadecadienoic acid | C18H32O2 |  | 5.18 | 4.71E-06 |
| 26 | 280.2372 | 280.2402 | 10 | 442.328577 | 5,11-octadecadienoic acid | C18H32O2 |  | 5.18 | 4.71E-06 |
| 26 | 280.2372 | 280.2402 | 10 | 442.328577 | 5,6-octadecadienoic acid | C18H32O2 |  | 5.18 | 4.71E-06 |
| 26 | 280.2372 | 280.2402 | 10 | 442.328577 | 5E,9Z-octadecadienoic acid | C18H32O2 |  | 5.18 | 4.71E-06 |
| 26 | 280.2372 | 280.2402 | 10 | 442.328577 | 5Z,11Z-octadecadienoic acid | C18H32O2 |  | 5.18 | 4.71E-06 |
| 26 | 280.2372 | 280.2402 | 10 | 442.328577 | 5Z,8Z-octadecadienoic acid | C18H32O2 |  | 5.18 | 4.71E-06 |
| 26 | 280.2372 | 280.2402 | 10 | 442.328577 | 5Z,9E-octadecadienoic acid | C18H32O2 |  | 5.18 | 4.71E-06 |
| 26 | 280.2372 | 280.2402 | 10 | 442.328577 | 5Z,9Z-octadecadienoic acid | C18H32O2 |  | 5.18 | 4.71E-06 |
| 26 | 280.2372 | 280.2402 | 10 | 442.328577 | 6,11-octadecadienoic acid | C18H32O2 |  | 5.18 | 4.71E-06 |
| 26 | 280.2372 | 280.2402 | 10 | 442.328577 | 6E,10E-octadecadienoic acid | C18H32O2 |  | 5.18 | 4.71E-06 |
| 26 | 280.2372 | 280.2402 | 10 | 442.328577 | 6E,11Z-octadecadienoic acid | C18H32O2 |  | 5.18 | 4.71E-06 |
| 26 | 280.2372 | 280.2402 | 10 | 442.328577 | 6E,12E-octadecadienoic acid | C18H32O2 |  | 5.18 | 4.71E-06 |
| 26 | 280.2372 | 280.2402 | 10 | 442.328577 | 6E,9E-octadecadienoic acid | C18H32O2 |  | 5.18 | 4.71E-06 |
| 26 | 280.2372 | 280.2402 | 10 | 442.328577 | 6Z,11Z-octadecadienoic acid | C18H32O2 |  | 5.18 | 4.71E-06 |
| 26 | 280.2372 | 280.2402 | 10 | 442.328577 | 6Z,9Z-octadecadienoic acid | C18H32O2 |  | 5.18 | 4.71E-06 |
| 26 | 280.2372 | 280.2402 | 10 | 442.328577 | 7E,12E-octadecadienoic acid | C18H32O2 |  | 5.18 | 4.71E-06 |
| 26 | 280.2372 | 280.2402 | 10 | 442.328577 | 7Z,10Z-octadecadienoic acid | C18H32O2 |  | 5.18 | 4.71E-06 |
| 26 | 280.2372 | 280.2402 | 10 | 442.328577 | 7Z,11Z-octadecadienoic acid | C18H32O2 |  | 5.18 | 4.71E-06 |
| 26 | 280.2372 | 280.2402 | 10 | 442.328577 | 8,11-octadecadienoic acid | C18H32O2 |  | 5.18 | 4.71E-06 |
| 26 | 280.2372 | 280.2402 | 10 | 442.328577 | 8,12-octadecadienoic acid | C18H32O2 |  | 5.18 | 4.71E-06 |
| 26 | 280.2372 | 280.2402 | 10 | 442.328577 | 9,13-octadecadienoic acid | C18H32O2 |  | 5.18 | 4.71E-06 |
| 26 | 280.2372 | 280.2402 | 10 | 442.328577 | 6-octadecynoic acid | C18H32O2 |  | 5.18 | 4.71E-06 |
| 26 | 280.2372 | 280.2402 | 10 | 442.328577 | 7-octadecynoic acid | C18H32O2 |  | 5.18 | 4.71E-06 |
| 26 | 280.2372 | 280.2402 | 10 | 442.328577 | 12-octadecynoic acid | C18H32O2 |  | 5.18 | 4.71E-06 |
| 26 | 280.2372 | 280.2402 | 10 | 442.328577 | 17-Octadecynoic Acid | C18H32O2 |  | 5.18 | 4.71E-06 |
| 26 | 280.2372 | 280.2402 | 10 | 442.328577 | Linoleic acid | C18H32O2 | C01595 | 5.18 | 4.71E-06 |
| 26 | 280.2372 | 280.2402 | 10 | 442.328577 | 9(Z),11(E)-Conjugated Linoleic Acid | C18H32O2 | C04056 | 5.18 | 4.71E-06 |
| 26 | 280.2372 | 280.2402 | 10 | 442.328577 | Stearolic acid | C18H32O2 | C08459 | 5.18 | 4.71E-06 |
| 26 | 280.2372 | 280.2402 | 10 | 442.328577 | Malvalic acid | C18H32O2 | C08321 | 5.18 | 4.71E-06 |
| 26 | 280.2372 | 280.2402 | 10 | 442.328577 | 7-trans,9-cis-octadecadienoic acid | C18H32O2 |  | 5.18 | 4.71E-06 |
| 26 | 280.2372 | 280.2402 | 10 | 442.328577 | Linoelaidic Acid | C18H32O2 |  | 5.18 | 4.71E-06 |
| 26 | 280.2372 | 280.2402 | 10 | 442.328577 | Chaulmoogric acid | C18H32O2 | C08282 | 5.18 | 4.71E-06 |
| 26 | 280.2372 | 280.2402 | 10 | 442.328577 | 16-methyl-6Z,9Z-heptadecadienoic acid | C18H32O2 |  | 5.18 | 4.71E-06 |
| 26 | 280.2372 | 280.2402 | 10 | 442.328577 | 16-methyl-9Z,12Z-heptadecadienoic acid | C18H32O2 |  | 5.18 | 4.71E-06 |
| 26 | 280.2372 | 280.2402 | 10 | 442.328577 | cis-5, cis-12-octadecadienoic acid; C18:2n-6,13 | C18H32O2 |  | 5.18 | 4.71E-06 |
| 26 | 280.2372 | 280.2402 | 10 | 442.328577 | cis-5, trans-12-octadecadienoic acid; C18:2n-6,13 | C18H32O2 |  | 5.18 | 4.71E-06 |
| 26 | 280.2372 | 280.2402 | 10 | 442.328577 | trans-5, cis12-octadecadienoic acid; C18:2n-6,13 | C18H32O2 |  | 5.18 | 4.71E-06 |
| 26 | 280.2372 | 280.2402 | 10 | 442.328577 | trans-5, trans-12-octadecadienoic acid; C18:2n-6,13 | C18H32O2 |  | 5.18 | 4.71E-06 |
| 26 | 280.2372 | 280.2402 | 10 | 442.328577 | trans-8, trans-10-octadecadienoic acid; C18:2n-8,10 | C18H32O2 |  | 5.18 | 4.71E-06 |
| 26 | 280.2372 | 280.2402 | 10 | 442.328577 | cis-8, cis-11-octadecadienoic acid; C18:2n-7,10 | C18H32O2 |  | 5.18 | 4.71E-06 |
| 26 | 280.2372 | 280.2402 | 10 | 442.328577 | cis-9, cis-11-octadecadienoic acid; C18:2n-7,9 | C18H32O2 |  | 5.18 | 4.71E-06 |
| 26 | 280.2372 | 280.2402 | 10 | 442.328577 | trans-9, trans-11-octadecadienoic acid; C18:2n-7,9 | C18H32O2 |  | 5.18 | 4.71E-06 |
| 26 | 280.2372 | 280.2402 | 10 | 442.328577 | cis-9, trans-12-octadecadienoic acid; C18:2n-6,9 | C18H32O2 |  | 5.18 | 4.71E-06 |
| 26 | 280.2372 | 280.2402 | 10 | 442.328577 | trans-9, cis-12-octadecadienoic acid; C18:2n-6,9 | C18H32O2 |  | 5.18 | 4.71E-06 |
| 26 | 280.2372 | 280.2402 | 10 | 442.328577 | cis-10, cis-12-octadecadienoic acid; C18:2n-6,8 | C18H32O2 |  | 5.18 | 4.71E-06 |
| 26 | 280.2372 | 280.2402 | 10 | 442.328577 | trans-10, cis-12-octadecadienoic acid; C18:2n-6,8 | C18H32O2 |  | 5.18 | 4.71E-06 |
| 26 | 280.2372 | 280.2402 | 10 | 442.328577 | trans-10, trans-12-octadecadienoic acid; C18:2n-6,8 | C18H32O2 |  | 5.18 | 4.71E-06 |
| 26 | 280.2372 | 280.2402 | 10 | 442.328577 | cis-10, cis-13-octadecadienoic acid; C18:2n-5,8 | C18H32O2 |  | 5.18 | 4.71E-06 |
| 26 | 280.2372 | 280.2402 | 10 | 442.328577 | 10E,14E-octadecadienoic acid | C18H32O2 |  | 5.18 | 4.71E-06 |
| 26 | 280.2372 | 280.2402 | 10 | 442.328577 | 10Z,14Z-octadecadienoic acid | C18H32O2 |  | 5.18 | 4.71E-06 |
| 26 | 280.2372 | 280.2402 | 10 | 442.328577 | 2E,6E-octadecadienoic acid | C18H32O2 |  | 5.18 | 4.71E-06 |
| 26 | 280.2372 | 280.2402 | 10 | 442.328577 | 2Z,6Z-octadecadienoic acid | C18H32O2 |  | 5.18 | 4.71E-06 |
| 26 | 280.2372 | 280.2402 | 10 | 442.328577 | 3E,7E-octadecadienoic acid | C18H32O2 |  | 5.18 | 4.71E-06 |
| 26 | 280.2372 | 280.2402 | 10 | 442.328577 | 3Z,7Z-octadecadienoic acid | C18H32O2 |  | 5.18 | 4.71E-06 |
| 26 | 280.2372 | 280.2402 | 10 | 442.328577 | 4E,8E-octadecadienoic acid | C18H32O2 |  | 5.18 | 4.71E-06 |
| 26 | 280.2372 | 280.2402 | 10 | 442.328577 | 4Z,8Z-octadecadienoic acid | C18H32O2 |  | 5.18 | 4.71E-06 |
| 26 | 280.2372 | 280.2402 | 10 | 442.328577 | 5,11-octadecadienoic acid | C18H32O2 |  | 5.18 | 4.71E-06 |
| 26 | 280.2372 | 280.2402 | 10 | 442.328577 | 5E,9Z-octadecadienoic acid | C18H32O2 |  | 5.18 | 4.71E-06 |
| 26 | 280.2372 | 280.2402 | 10 | 442.328577 | 5Z,11Z-octadecadienoic acid | C18H32O2 |  | 5.18 | 4.71E-06 |
| 26 | 280.2372 | 280.2402 | 10 | 442.328577 | Sebaleic acid | C18H32O2 |  | 5.18 | 4.71E-06 |
| 26 | 280.2372 | 280.2402 | 10 | 442.328577 | 5Z,9E-octadecadienoic acid | C18H32O2 |  | 5.18 | 4.71E-06 |
| 26 | 280.2372 | 280.2402 | 10 | 442.328577 | Taxoleic acid | C18H32O2 |  | 5.18 | 4.71E-06 |
| 26 | 280.2372 | 280.2402 | 10 | 442.328577 | 6,11-octadecadienoic acid | C18H32O2 |  | 5.18 | 4.71E-06 |
| 26 | 280.2372 | 280.2402 | 10 | 442.328577 | 6E,11Z-octadecadienoic acid | C18H32O2 |  | 5.18 | 4.71E-06 |
| 26 | 280.2372 | 280.2402 | 10 | 442.328577 | 6Z,11Z-octadecadienoic acid | C18H32O2 |  | 5.18 | 4.71E-06 |
| 26 | 280.2372 | 280.2402 | 10 | 442.328577 | cis,cis-6,9-octadecadienoic acid; C18:2n-9,12 | C18H32O2 |  | 5.18 | 4.71E-06 |
| 26 | 280.2372 | 280.2402 | 10 | 442.328577 | 6-Octadecynoic acid; Tariric acid; 6,7-Stearolic acid; 6-Stearolic acid | C18H32O2 |  | 5.18 | 4.71E-06 |
| 26 | 280.2372 | 280.2402 | 10 | 442.328577 | 8-Octadecynoic acid | C18H32O2 |  | 5.18 | 4.71E-06 |
| 26 | 280.2372 | 280.2402 | 10 | 442.328577 | 2-Octadecynoic acid | C18H32O2 |  | 5.18 | 4.71E-06 |
| 26 | 280.2372 | 280.2402 | 10 | 442.328577 | 3-Octadecynoic acid | C18H32O2 |  | 5.18 | 4.71E-06 |
| 26 | 280.2372 | 280.2402 | 10 | 442.328577 | 4-Octadecynoic acid | C18H32O2 |  | 5.18 | 4.71E-06 |
| 26 | 280.2372 | 280.2402 | 10 | 442.328577 | 5-Octadecynoic acid | C18H32O2 |  | 5.18 | 4.71E-06 |
| 26 | 280.2372 | 280.2402 | 10 | 442.328577 | 10-Octadecynoic acid | C18H32O2 |  | 5.18 | 4.71E-06 |
| 26 | 280.2372 | 280.2402 | 10 | 442.328577 | 11-Octadecynoic acid | C18H32O2 |  | 5.18 | 4.71E-06 |
| 26 | 280.2372 | 280.2402 | 10 | 442.328577 | 12-Octadecynoic acid; 12-Stearolic acid | C18H32O2 |  | 5.18 | 4.71E-06 |
| 26 | 280.2372 | 280.2402 | 10 | 442.328577 | 13-Octadecynoic acid | C18H32O2 |  | 5.18 | 4.71E-06 |
| 26 | 280.2372 | 280.2402 | 10 | 442.328577 | 14-Octadecynoic acid | C18H32O2 |  | 5.18 | 4.71E-06 |
| 26 | 280.2372 | 280.2402 | 10 | 442.328577 | 15-Octadecynoic acid | C18H32O2 |  | 5.18 | 4.71E-06 |
| 26 | 280.2372 | 280.2402 | 10 | 442.328577 | 16-Octadecynoic acid | C18H32O2 |  | 5.18 | 4.71E-06 |
| 26 | 280.2372 | 280.2402 | 10 | 442.328577 | (R)-laballenic acid | C18H32O2 |  | 5.18 | 4.71E-06 |
| 26 | 280.2372 | 280.2402 | 10 | 442.328577 | (S)-laballenic acid | C18H32O2 |  | 5.18 | 4.71E-06 |
| 26 | 280.2372 | 280.2402 | 10 | 442.328577 | Mangiferic acid | C18H32O2 |  | 5.18 | 4.71E-06 |
| 27 | 270.2176 | 270.2195 | 6 | 432.3089728 | Ambrettolic acid | C16H30O3 |  | 2.10 | 9.36E-06 |
| 27 | 270.2176 | 270.2195 | 6 | 432.3089728 | (+)-12-hydroxy-9Z-hexadecenoic acid | C16H30O3 |  | 2.10 | 9.36E-06 |
| 27 | 270.2176 | 270.2195 | 6 | 432.3089728 | 16-hydroxy-5-hexadecenoic acid | C16H30O3 |  | 2.10 | 9.36E-06 |
| 27 | 270.2176 | 270.2195 | 6 | 432.3089728 | 16-hydroxy-6-hexadecenoic acid | C16H30O3 |  | 2.10 | 9.36E-06 |
| 27 | 270.2176 | 270.2195 | 6 | 432.3089728 | 2-keto palmitic acid | C16H30O3 |  | 2.10 | 9.36E-06 |
| 27 | 270.2176 | 270.2195 | 6 | 432.3089728 | 3-keto palmitic acid | C16H30O3 |  | 2.10 | 9.36E-06 |
| 27 | 270.2176 | 270.2195 | 6 | 432.3089728 | 4-keto palmitic acid | C16H30O3 |  | 2.10 | 9.36E-06 |
| 27 | 270.2176 | 270.2195 | 6 | 432.3089728 | 5-keto palmitic acid | C16H30O3 |  | 2.10 | 9.36E-06 |
| 27 | 270.2176 | 270.2195 | 6 | 432.3089728 | 7-keto palmitic acid | C16H30O3 |  | 2.10 | 9.36E-06 |
| 27 | 270.2176 | 270.2195 | 6 | 432.3089728 | 8-keto palmitic acid | C16H30O3 |  | 2.10 | 9.36E-06 |
| 27 | 270.2176 | 270.2195 | 6 | 432.3089728 | 9-keto palmitic acid | C16H30O3 |  | 2.10 | 9.36E-06 |
| 27 | 270.2176 | 270.2195 | 6 | 432.3089728 | 10-keto palmitic acid | C16H30O3 |  | 2.10 | 9.36E-06 |
| 27 | 270.2176 | 270.2195 | 6 | 432.3089728 | 11-keto palmitic acid | C16H30O3 |  | 2.10 | 9.36E-06 |
| 27 | 270.2176 | 270.2195 | 6 | 432.3089728 | 16-hydroxy-9E-hexadecenoic acid | C16H30O3 |  | 2.10 | 9.36E-06 |
| 27 | 270.2176 | 270.2195 | 6 | 432.3089728 | 16-hydroxy-9Z-hexadecenoic acid | C16H30O3 |  | 2.10 | 9.36E-06 |
| 27 | 270.2176 | 270.2195 | 6 | 432.3089728 | 9-Hexadecenoic acid, 12-hydroxy-, (Z)-(+)- | C16H30O3 |  | 2.10 | 9.36E-06 |
| 27 | 270.2176 | 270.2195 | 6 | 432.3089728 | 5-Hexadecenoic acid, 16-hydroxy-; Delta5-Isoambrettolic acid | C16H30O3 |  | 2.10 | 9.36E-06 |
| 27 | 270.2176 | 270.2195 | 6 | 432.3089728 | 6-Hexadecenoic acid, 16-hydroxy-; Delta6-Isoambrettolic acid | C16H30O3 |  | 2.10 | 9.36E-06 |
| 27 | 270.2176 | 270.2195 | 6 | 432.3089728 | 10-oxo-14-methyl-pentadecanoic acid | C16H30O3 |  | 2.10 | 9.36E-06 |
| 27 | 270.2176 | 270.2195 | 6 | 432.3089728 | 2-methyl-4-oxo-pentadecanoic acid | C16H30O3 |  | 2.10 | 9.36E-06 |
| 27 | 270.2176 | 270.2195 | 6 | 432.3089728 | 15-oxo-hexadecanoic acid | C16H30O3 |  | 2.10 | 9.36E-06 |
| 28 | 224.1767 | 224.1776 | 4 | 386.2680293 | Alepric acid | C14H24O2 |  | 3.87 | 7.31E-09 |
| 28 | 224.1767 | 224.1776 | 4 | 386.2680293 | 10Z,12E-tetradecadienoic acid | C14H24O2 |  | 3.87 | 7.31E-09 |
| 28 | 224.1767 | 224.1776 | 4 | 386.2680293 | 2E,4E-tetradecadienoic acid | C14H24O2 |  | 3.87 | 7.31E-09 |
| 28 | 224.1767 | 224.1776 | 4 | 386.2680293 | 3,4-tetradecadienoic acid | C14H24O2 |  | 3.87 | 7.31E-09 |
| 28 | 224.1767 | 224.1776 | 4 | 386.2680293 | 3Z,5E-tetradecadienoic acid | C14H24O2 |  | 3.87 | 7.31E-09 |
| 28 | 224.1767 | 224.1776 | 4 | 386.2680293 | 3Z,5Z-tetradecadienoic acid | C14H24O2 |  | 3.87 | 7.31E-09 |
| 28 | 224.1767 | 224.1776 | 4 | 386.2680293 | 5Z,8Z-tetradecadienoic acid | C14H24O2 |  | 3.87 | 7.31E-09 |
| 28 | 224.1767 | 224.1776 | 4 | 386.2680293 | Myristic Acid Alkyne | C14H24O2 |  | 3.87 | 7.31E-09 |
| 28 | 224.1767 | 224.1776 | 4 | 386.2680293 | 5,8-Tetradecadienoic acid | C14H24O2 |  | 3.87 | 7.31E-09 |
| 28 | 224.1767 | 224.1776 | 4 | 386.2680293 | 3E,5Z-Tetradecadienoic acid | C14H24O2 |  | 3.87 | 7.31E-09 |
| 28 | 224.1767 | 224.1776 | 4 | 386.2680293 | 3Z,5E-tetradecadienoic acid | C14H24O2 |  | 3.87 | 7.31E-09 |
| 28 | 224.1767 | 224.1776 | 4 | 386.2680293 | 3Z,5Z-tetradecadienoic acid | C14H24O2 |  | 3.87 | 7.31E-09 |
| 28 | 224.1767 | 224.1776 | 4 | 386.2680293 | 5,8-tetradecadienoic acid | C14H24O2 |  | 3.87 | 7.31E-09 |
| 28 | 224.1767 | 224.1776 | 4 | 386.2680293 | 5Z,8Z-tetradecadienoic acid | C14H24O2 |  | 3.87 | 7.31E-09 |
| 28 | 224.1767 | 224.1776 | 4 | 386.2680293 | 3-Tetradecynoic acid | C14H24O2 |  | 3.87 | 7.31E-09 |
| 28 | 224.1767 | 224.1776 | 4 | 386.2680293 | 4-Tetradecynoic acid | C14H24O2 |  | 3.87 | 7.31E-09 |
| 28 | 224.1767 | 224.1776 | 4 | 386.2680293 | 5-Tetradecynoic acid | C14H24O2 |  | 3.87 | 7.31E-09 |
| 28 | 224.1767 | 224.1776 | 4 | 386.2680293 | 6-Tetradecynoic acid | C14H24O2 |  | 3.87 | 7.31E-09 |
| 28 | 224.1767 | 224.1776 | 4 | 386.2680293 | 7-Tetradecynoic acid | C14H24O2 |  | 3.87 | 7.31E-09 |
| 28 | 224.1767 | 224.1776 | 4 | 386.2680293 | 8-Tetradecynoic acid | C14H24O2 |  | 3.87 | 7.31E-09 |
| 28 | 224.1767 | 224.1776 | 4 | 386.2680293 | 9-Tetradecynoic acid | C14H24O2 |  | 3.87 | 7.31E-09 |
| 28 | 224.1767 | 224.1776 | 4 | 386.2680293 | 10-Tetradecynoic acid | C14H24O2 |  | 3.87 | 7.31E-09 |
| 28 | 224.1767 | 224.1776 | 4 | 386.2680293 | 11-Tetradecynoic acid | C14H24O2 |  | 3.87 | 7.31E-09 |
| 28 | 224.1767 | 224.1776 | 4 | 386.2680293 | 12-Tetradecynoic acid | C14H24O2 |  | 3.87 | 7.31E-09 |
| 28 | 224.1767 | 224.1776 | 4 | 386.2680293 | 13-Tetradecynoic acid | C14H24O2 |  | 3.87 | 7.31E-09 |
| 28 | 224.1767 | 224.1776 | 4 | 386.2680293 | Goshuyic acid | C14H24O2 |  | 3.87 | 7.31E-09 |
| 29 | 296.2339 | 296.2351 | 4 | 458.3252012 | Dimorphecolic acid | C18H32O3 |  | 4.28 | 9.53E-06 |
| 29 | 296.2339 | 296.2351 | 4 | 458.3252012 | Densipolic acid | C18H32O3 |  | 4.28 | 9.53E-06 |
| 29 | 296.2339 | 296.2351 | 4 | 458.3252012 | alpha-artemisic acid | C18H32O3 |  | 4.28 | 9.53E-06 |
| 29 | 296.2339 | 296.2351 | 4 | 458.3252012 | 6-hydroxy-9Z,12Z-octadecadienoic acid | C18H32O3 |  | 4.28 | 9.53E-06 |
| 29 | 296.2339 | 296.2351 | 4 | 458.3252012 | 9S-hydroxy-10E,12E-octadecadienoic acid | C18H32O3 |  | 4.28 | 9.53E-06 |
| 29 | 296.2339 | 296.2351 | 4 | 458.3252012 | 12R-hydroxy-9Z,15Z-octadecadienoic acid | C18H32O3 |  | 4.28 | 9.53E-06 |
| 29 | 296.2339 | 296.2351 | 4 | 458.3252012 | 13R-hydroxy-9E,11Z-octadecadienoic acid | C18H32O3 |  | 4.28 | 9.53E-06 |
| 29 | 296.2339 | 296.2351 | 4 | 458.3252012 | 13S-hydroxy-9E,11Z-octadecadienoic acid | C18H32O3 |  | 4.28 | 9.53E-06 |
| 29 | 296.2339 | 296.2351 | 4 | 458.3252012 | 12-oxo-10E-octadecenoic acid | C18H32O3 |  | 4.28 | 9.53E-06 |
| 29 | 296.2339 | 296.2351 | 4 | 458.3252012 | 12-oxo-10Z-octadecenoic acid | C18H32O3 |  | 4.28 | 9.53E-06 |
| 29 | 296.2339 | 296.2351 | 4 | 458.3252012 | 12-oxo-9E-octadecenoic acid | C18H32O3 |  | 4.28 | 9.53E-06 |
| 29 | 296.2339 | 296.2351 | 4 | 458.3252012 | 12-oxo-9Z-octadecenoic acid | C18H32O3 |  | 4.28 | 9.53E-06 |
| 29 | 296.2339 | 296.2351 | 4 | 458.3252012 | 12,13-epoxy-9-octadecenoic acid | C18H32O3 |  | 4.28 | 9.53E-06 |
| 29 | 296.2339 | 296.2351 | 4 | 458.3252012 | 9,10-epoxy-12-octadecenoic acid | C18H32O3 |  | 4.28 | 9.53E-06 |
| 29 | 296.2339 | 296.2351 | 4 | 458.3252012 | 12R,13S-epoxy-9Z-octadecenoic acid | C18H32O3 | C08368 | 4.28 | 9.53E-06 |
| 29 | 296.2339 | 296.2351 | 4 | 458.3252012 | 7-Methoxy-9-methyl-hexadeca-4E,8E-dienoic acid | C18H32O3 |  | 4.28 | 9.53E-06 |
| 29 | 296.2339 | 296.2351 | 4 | 458.3252012 | Avenoleic acid | C18H32O3 |  | 4.28 | 9.53E-06 |
| 29 | 296.2339 | 296.2351 | 4 | 458.3252012 | 10-keto-12Z-octadecenoic acid | C18H32O3 |  | 4.28 | 9.53E-06 |
| 29 | 296.2339 | 296.2351 | 4 | 458.3252012 | 2R-hydroxy-linoleic acid | C18H32O3 |  | 4.28 | 9.53E-06 |
| 29 | 296.2339 | 296.2351 | 4 | 458.3252012 | beta-Dimorphecolic acid | C18H32O3 |  | 4.28 | 9.53E-06 |
| 29 | 296.2339 | 296.2351 | 4 | 458.3252012 | 12-hydroxy-9-octadecynoic acid | C18H32O3 |  | 4.28 | 9.53E-06 |
| 29 | 296.2339 | 296.2351 | 4 | 458.3252012 | 12S-hydroxy-9-octadecynoic acid | C18H32O3 |  | 4.28 | 9.53E-06 |
| 29 | 296.2339 | 296.2351 | 4 | 458.3252012 | 12-hydroxy-10-octadecynoic acid | C18H32O3 |  | 4.28 | 9.53E-06 |
| 29 | 296.2339 | 296.2351 | 4 | 458.3252012 | (1R,2R)-3-oxo-2-pentyl-cyclopentaneoctanoic acid | C18H32O3 |  | 4.28 | 9.53E-06 |
| 29 | 296.2339 | 296.2351 | 4 | 458.3252012 | (1S,2S)-3-oxo-2-pentyl-cyclopentaneoctanoic acid | C18H32O3 |  | 4.28 | 9.53E-06 |
| 29 | 296.2339 | 296.2351 | 4 | 458.3252012 | (Z)-13-Oxo-9-octadecenoic acid | C18H32O3 |  | 4.28 | 9.53E-06 |
| 29 | 296.2339 | 296.2351 | 4 | 458.3252012 | 12-Hydroxy-8,10-octadecadienoic acid | C18H32O3 |  | 4.28 | 9.53E-06 |
| 30 | 250.1923 | 250.1933 | 4 | 412.2836013 | 4,7,10-hexadecatrienoic acid | C16H26O2 |  | 5.64 | 9.33E-07 |
| 30 | 250.1923 | 250.1933 | 4 | 412.2836013 | 5E,8E,11E-hexadecatrienoic acid | C16H26O2 |  | 5.64 | 9.33E-07 |
| 30 | 250.1923 | 250.1933 | 4 | 412.2836013 | 6,9,12-hexadecatrienoic acid | C16H26O2 |  | 5.64 | 9.33E-07 |
| 30 | 250.1923 | 250.1933 | 4 | 412.2836013 | Hiragonic acid | C16H26O2 |  | 5.64 | 9.33E-07 |
| 30 | 250.1923 | 250.1933 | 4 | 412.2836013 | 7,10,13-hexadecatrienoic acid | C16H26O2 |  | 5.64 | 9.33E-07 |
| 30 | 250.1923 | 250.1933 | 4 | 412.2836013 | 9,12,15-hexadecatrienoic acid | C16H26O2 |  | 5.64 | 9.33E-07 |
| 30 | 250.1923 | 250.1933 | 4 | 412.2836013 | 7,10,13-hexadecatrienoic acid / Roughanic acid | C16H26O2 |  | 5.64 | 9.33E-07 |
| 30 | 250.1923 | 250.1933 | 4 | 412.2836013 | trans-5, trans-8, trans-11-hexadecatrienoic acid; C16:3n-5,8,11 | C16H26O2 |  | 5.64 | 9.33E-07 |
| 30 | 250.1923 | 250.1933 | 4 | 412.2836013 | 7Z,10Z,13Z-hexadecatrienoic acid | C16H26O2 |  | 5.64 | 9.33E-07 |
| 31 | 250.1917 | 250.1933 | 6 | 412.2830893 | 4,7,10-hexadecatrienoic acid | C16H26O2 |  | 6.65 | 3.81E-07 |
| 31 | 250.1917 | 250.1933 | 6 | 412.2830893 | 5E,8E,11E-hexadecatrienoic acid | C16H26O2 |  | 6.65 | 3.81E-07 |
| 31 | 250.1917 | 250.1933 | 6 | 412.2830893 | 6,9,12-hexadecatrienoic acid | C16H26O2 |  | 6.65 | 3.81E-07 |
| 31 | 250.1917 | 250.1933 | 6 | 412.2830893 | Hiragonic acid | C16H26O2 |  | 6.65 | 3.81E-07 |
| 31 | 250.1917 | 250.1933 | 6 | 412.2830893 | 7,10,13-hexadecatrienoic acid | C16H26O2 |  | 6.65 | 3.81E-07 |
| 31 | 250.1917 | 250.1933 | 6 | 412.2830893 | 9,12,15-hexadecatrienoic acid | C16H26O2 |  | 6.65 | 3.81E-07 |
| 31 | 250.1917 | 250.1933 | 6 | 412.2830893 | 7,10,13-hexadecatrienoic acid / Roughanic acid | C16H26O2 |  | 6.65 | 3.81E-07 |
| 31 | 250.1917 | 250.1933 | 6 | 412.2830893 | trans-5, trans-8, trans-11-hexadecatrienoic acid; C16:3n-5,8,11 | C16H26O2 |  | 6.65 | 3.81E-07 |
| 31 | 250.1917 | 250.1933 | 6 | 412.2830893 | 7Z,10Z,13Z-hexadecatrienoic acid | C16H26O2 |  | 6.65 | 3.81E-07 |
| 32 | 226.1930 | 226.1933 | 1 | 388.2843131 | 2-tetradecenoic acid | C14H26O2 |  | 34.03 | 9.61E-07 |
| 32 | 226.1930 | 226.1933 | 1 | 388.2843131 | Tsuzuic acid | C14H26O2 |  | 34.03 | 9.61E-07 |
| 32 | 226.1930 | 226.1933 | 1 | 388.2843131 | Physeteric acid | C14H26O2 |  | 34.03 | 9.61E-07 |
| 32 | 226.1930 | 226.1933 | 1 | 388.2843131 | 8Z-tetradecenoic acid | C14H26O2 |  | 34.03 | 9.61E-07 |
| 32 | 226.1930 | 226.1933 | 1 | 388.2843131 | 3E-tetradecenoic acid | C14H26O2 |  | 34.03 | 9.61E-07 |
| 32 | 226.1930 | 226.1933 | 1 | 388.2843131 | 4Z-tetradecenoic acid | C14H26O2 |  | 34.03 | 9.61E-07 |
| 32 | 226.1930 | 226.1933 | 1 | 388.2843131 | 5Z-tetradecenoic acid | C14H26O2 |  | 34.03 | 9.61E-07 |
| 32 | 226.1930 | 226.1933 | 1 | 388.2843131 | 7Z-tetradecenoic acid | C14H26O2 |  | 34.03 | 9.61E-07 |
| 32 | 226.1930 | 226.1933 | 1 | 388.2843131 | 9E-tetradecenoic acid | C14H26O2 |  | 34.03 | 9.61E-07 |
| 32 | 226.1930 | 226.1933 | 1 | 388.2843131 | 5-methyl-2E-tridecenoic acid | C14H26O2 |  | 34.03 | 9.61E-07 |
| 32 | 226.1930 | 226.1933 | 1 | 388.2843131 | cis-&Delta;2-11-methyl-Dodecenoic Acid | C14H26O2 |  | 34.03 | 9.61E-07 |
| 32 | 226.1930 | 226.1933 | 1 | 388.2843131 | Myristoleic acid | C14H26O2 | C08322 | 34.03 | 9.61E-07 |
| 32 | 226.1930 | 226.1933 | 1 | 388.2843131 | cis-tetradec-11-enoic acid | C14H26O2 |  | 34.03 | 9.61E-07 |
| 32 | 226.1930 | 226.1933 | 1 | 388.2843131 | trans-tetradec-11-enoic acid | C14H26O2 |  | 34.03 | 9.61E-07 |
| 32 | 226.1930 | 226.1933 | 1 | 388.2843131 | 2,4-dimethyl-2E-dodecenoic acid | C14H26O2 |  | 34.03 | 9.61E-07 |
| 32 | 226.1930 | 226.1933 | 1 | 388.2843131 | cis-8-tetradecenoic acid; C14:1n-6 | C14H26O2 |  | 34.03 | 9.61E-07 |
| 32 | 226.1930 | 226.1933 | 1 | 388.2843131 | 5E-Tetradecenoic acid | C14H26O2 |  | 34.03 | 9.61E-07 |
| 33 | 252.2076 | 252.2089 | 5 | 414.2989056 | 9,12-hexadecadienoic acid | C16H28O2 |  | 8.83 | 3.44E-06 |
| 33 | 252.2076 | 252.2089 | 5 | 414.2989056 | 10Z,12E-hexadecadienoic acid | C16H28O2 |  | 8.83 | 3.44E-06 |
| 33 | 252.2076 | 252.2089 | 5 | 414.2989056 | 2E,4E-hexadecadienoic acid | C16H28O2 |  | 8.83 | 3.44E-06 |
| 33 | 252.2076 | 252.2089 | 5 | 414.2989056 | 2E,4Z-hexadecadienoic acid | C16H28O2 |  | 8.83 | 3.44E-06 |
| 33 | 252.2076 | 252.2089 | 5 | 414.2989056 | 3Z,9Z-hexadecadienoic acid | C16H28O2 |  | 8.83 | 3.44E-06 |
| 33 | 252.2076 | 252.2089 | 5 | 414.2989056 | 6,9-hexadecadienoic acid | C16H28O2 |  | 8.83 | 3.44E-06 |
| 33 | 252.2076 | 252.2089 | 5 | 414.2989056 | 6Z,9Z-hexadecadienoic acid | C16H28O2 |  | 8.83 | 3.44E-06 |
| 33 | 252.2076 | 252.2089 | 5 | 414.2989056 | 8Z,10Z-hexadecadienoic acid | C16H28O2 |  | 8.83 | 3.44E-06 |
| 33 | 252.2076 | 252.2089 | 5 | 414.2989056 | 9Z,12Z-hexadecadienoic acid | C16H28O2 |  | 8.83 | 3.44E-06 |
| 33 | 252.2076 | 252.2089 | 5 | 414.2989056 | 7-hexadecynoic acid | C16H28O2 |  | 8.83 | 3.44E-06 |
| 33 | 252.2076 | 252.2089 | 5 | 414.2989056 | Hydnocarpic acid | C16H28O2 |  | 8.83 | 3.44E-06 |
| 33 | 252.2076 | 252.2089 | 5 | 414.2989056 | 7,10-hexadecadienoic acid | C16H28O2 |  | 8.83 | 3.44E-06 |
| 33 | 252.2076 | 252.2089 | 5 | 414.2989056 | 7Z,10Z-hexadecadienoic acid | C16H28O2 |  | 8.83 | 3.44E-06 |
| 33 | 252.2076 | 252.2089 | 5 | 414.2989056 | 5Z,9Z-hexadecadienoic acid | C16H28O2 |  | 8.83 | 3.44E-06 |
| 33 | 252.2076 | 252.2089 | 5 | 414.2989056 | (R)-Hydnocarpic acid | C16H28O2 | C16795 | 8.83 | 3.44E-06 |
| 33 | 252.2076 | 252.2089 | 5 | 414.2989056 | 6,9-hexadecadienoic acid | C16H28O2 |  | 8.83 | 3.44E-06 |
| 33 | 252.2076 | 252.2089 | 5 | 414.2989056 | 6Z,9Z-hexadecadienoic acid | C16H28O2 |  | 8.83 | 3.44E-06 |
| 33 | 252.2076 | 252.2089 | 5 | 414.2989056 | Palmitolinoleic acid | C16H28O2 |  | 8.83 | 3.44E-06 |
| 33 | 252.2076 | 252.2089 | 5 | 414.2989056 | 2-Hexadecynoic acid | C16H28O2 |  | 8.83 | 3.44E-06 |
| 33 | 252.2076 | 252.2089 | 5 | 414.2989056 | 4-Hexadecynoic acid | C16H28O2 |  | 8.83 | 3.44E-06 |
| 33 | 252.2076 | 252.2089 | 5 | 414.2989056 | 7-Hexadecynoic acid; Palmitolic acid | C16H28O2 |  | 8.83 | 3.44E-06 |
| 33 | 252.2076 | 252.2089 | 5 | 414.2989056 | 10-Hexadecynoic acid | C16H28O2 |  | 8.83 | 3.44E-06 |
| 34 | 266.2225 | 266.2246 | 7 | 428.3138537 | 7-Heptadecynoic acid | C17H30O2 |  | 2.51 | 7.47E-06 |
| 34 | 266.2225 | 266.2246 | 7 | 428.3138537 | 8-Heptadecynoic acid | C17H30O2 |  | 2.51 | 7.47E-06 |
| 34 | 266.2225 | 266.2246 | 7 | 428.3138537 | 9-Heptadecynoic acid | C17H30O2 |  | 2.51 | 7.47E-06 |
| 34 | 266.2225 | 266.2246 | 7 | 428.3138537 | 12-Heptadecynoic acid | C17H30O2 |  | 2.51 | 7.47E-06 |
| 34 | 266.2225 | 266.2246 | 7 | 428.3138537 | 16-Heptadecynoic acid | C17H30O2 |  | 2.51 | 7.47E-06 |
| 35 | 254.2232 | 254.2246 | 5 | 416.3145406 | Gaidic acid | C16H30O2 |  | 3.51 | 1.56E-06 |
| 35 | 254.2232 | 254.2246 | 5 | 416.3145406 | 7-palmitoleic acid | C16H30O2 |  | 3.51 | 1.56E-06 |
| 35 | 254.2232 | 254.2246 | 5 | 416.3145406 | cis-10-palmitoleic acid | C16H30O2 |  | 3.51 | 1.56E-06 |
| 35 | 254.2232 | 254.2246 | 5 | 416.3145406 | 10-hexadecenoic acid | C16H30O2 |  | 3.51 | 1.56E-06 |
| 35 | 254.2232 | 254.2246 | 5 | 416.3145406 | 11-hexadecenoic acid | C16H30O2 |  | 3.51 | 1.56E-06 |
| 35 | 254.2232 | 254.2246 | 5 | 416.3145406 | 11Z-hexadecenoic acid | C16H30O2 |  | 3.51 | 1.56E-06 |
| 35 | 254.2232 | 254.2246 | 5 | 416.3145406 | 13-hexadecenoic acid | C16H30O2 |  | 3.51 | 1.56E-06 |
| 35 | 254.2232 | 254.2246 | 5 | 416.3145406 | 13Z-hexadecenoic acid | C16H30O2 |  | 3.51 | 1.56E-06 |
| 35 | 254.2232 | 254.2246 | 5 | 416.3145406 | 3E-hexadecenoic acid | C16H30O2 |  | 3.51 | 1.56E-06 |
| 35 | 254.2232 | 254.2246 | 5 | 416.3145406 | 6Z-hexadecenoic acid | C16H30O2 |  | 3.51 | 1.56E-06 |
| 35 | 254.2232 | 254.2246 | 5 | 416.3145406 | cis-7-Hexadecenoic Acid | C16H30O2 |  | 3.51 | 1.56E-06 |
| 35 | 254.2232 | 254.2246 | 5 | 416.3145406 | cis-9-palmitoleic acid | C16H30O2 | C08362 | 3.51 | 1.56E-06 |
| 35 | 254.2232 | 254.2246 | 5 | 416.3145406 | trans-9-palmitoleic acid | C16H30O2 | C08362 | 3.51 | 1.56E-06 |
| 35 | 254.2232 | 254.2246 | 5 | 416.3145406 | Hypogeic acid | C16H30O2 |  | 3.51 | 1.56E-06 |
| 35 | 254.2232 | 254.2246 | 5 | 416.3145406 | 2,4-dimethyl-2E-tetradecenoic acid | C16H30O2 |  | 3.51 | 1.56E-06 |
| 35 | 254.2232 | 254.2246 | 5 | 416.3145406 | 14-methyl-4-pentadecenoic acid | C16H30O2 |  | 3.51 | 1.56E-06 |
| 35 | 254.2232 | 254.2246 | 5 | 416.3145406 | 2-hexyl-2-decenoic acid | C16H30O2 |  | 3.51 | 1.56E-06 |
| 35 | 254.2232 | 254.2246 | 5 | 416.3145406 | 6-isopentyl-9-methyl-5-decenoic acid | C16H30O2 |  | 3.51 | 1.56E-06 |
| 35 | 254.2232 | 254.2246 | 5 | 416.3145406 | cis-Palmitvaccenic acid | C16H30O2 |  | 3.51 | 1.56E-06 |
| 35 | 254.2232 | 254.2246 | 5 | 416.3145406 | 13-hexadecenoic acid | C16H30O2 |  | 3.51 | 1.56E-06 |
| 35 | 254.2232 | 254.2246 | 5 | 416.3145406 | 13Z-hexadecenoic acid | C16H30O2 |  | 3.51 | 1.56E-06 |
| 35 | 254.2232 | 254.2246 | 5 | 416.3145406 | 3E-Hexadecenoic acid | C16H30O2 |  | 3.51 | 1.56E-06 |
| 35 | 254.2232 | 254.2246 | 5 | 416.3145406 | Sapienic acid | C16H30O2 |  | 3.51 | 1.56E-06 |
| 35 | 254.2232 | 254.2246 | 5 | 416.3145406 | hexadec-7Z-enoic acid | C16H30O2 |  | 3.51 | 1.56E-06 |
| 35 | 254.2232 | 254.2246 | 5 | 416.3145406 | (Z)-5-Hexadecenoic acid | C16H30O2 |  | 3.51 | 1.56E-06 |
| 35 | 254.2232 | 254.2246 | 5 | 416.3145406 | (E)-3-Hexadecenoic acid | C16H30O2 |  | 3.51 | 1.56E-06 |
| 35 | 254.2232 | 254.2246 | 5 | 416.3145406 | (Z)-14-Methyl-6-pentadecenoic acid | C16H30O2 |  | 3.51 | 1.56E-06 |
| 35 | 254.2232 | 254.2246 | 5 | 416.3145406 | &Delta;2-trans-Hexadecenoic Acid | C16H30O2 |  | 3.51 | 1.56E-06 |
| 35 | 254.2232 | 254.2246 | 5 | 416.3145406 | &Delta;2-cis-Hexadecenoic Acid | C16H30O2 |  | 3.51 | 1.56E-06 |
| 36 | 338.2436 | 338.2457 | 6 | 500.334903 | 5,6-dihydroxy-8,11,14-eicosatrienoic acid | C20H34O4 |  | 4.22 | 3.71E-07 |
| 36 | 338.2436 | 338.2457 | 6 | 500.334903 | 8,9-dihydroxy-5,11,14-eicosatrienoic acid | C20H34O4 |  | 4.22 | 3.71E-07 |
| 36 | 338.2436 | 338.2457 | 6 | 500.334903 | 8,11,14-Eicosatrienoic acid, 5,6-dihydroxy-; 5,6-Dihydroxy-8,11,14-eicosatrienoic acid | C20H34O4 |  | 4.22 | 3.71E-07 |
| 36 | 338.2436 | 338.2457 | 6 | 500.334903 | 5,11,14-Eicosatrienoic acid, 8,9-dihydroxy-; 8,9-Dihydroxy-5,11,14-eicosatrienoic acid | C20H34O4 |  | 4.22 | 3.71E-07 |
| 36 | 338.2436 | 338.2457 | 6 | 500.334903 | 8R,9S-cis-epoxy-10S-hydroxy-eicosa-11Z,14Z-dienoic acid. | C20H34O4 |  | 4.22 | 3.71E-07 |
| 37 | 280.2390 | 280.2402 | 4 | 442.3303471 | 5Z,12Z-otadecadienoic acid | C18H32O2 |  | 6.66 | 2.91E-07 |
| 37 | 280.2390 | 280.2402 | 4 | 442.3303471 | 5Z,12E-otadecadienoic acid | C18H32O2 |  | 6.66 | 2.91E-07 |
| 37 | 280.2390 | 280.2402 | 4 | 442.3303471 | 5E,12Z-otadecadienoic acid | C18H32O2 |  | 6.66 | 2.91E-07 |
| 37 | 280.2390 | 280.2402 | 4 | 442.3303471 | 5E,12E-otadecadienoic acid | C18H32O2 |  | 6.66 | 2.91E-07 |
| 37 | 280.2390 | 280.2402 | 4 | 442.3303471 | 6, 8-octadecadienoic acid | C18H32O2 |  | 6.66 | 2.91E-07 |
| 37 | 280.2390 | 280.2402 | 4 | 442.3303471 | 8E,10E-octadecadienoic acid | C18H32O2 |  | 6.66 | 2.91E-07 |
| 37 | 280.2390 | 280.2402 | 4 | 442.3303471 | 8Z,11Z-octadecadienoic acid | C18H32O2 |  | 6.66 | 2.91E-07 |
| 37 | 280.2390 | 280.2402 | 4 | 442.3303471 | 9Z,11Z-octadecadienoic acid | C18H32O2 |  | 6.66 | 2.91E-07 |
| 37 | 280.2390 | 280.2402 | 4 | 442.3303471 | 9(E),11(E)-Conjugated Linoleic Acid | C18H32O2 |  | 6.66 | 2.91E-07 |
| 37 | 280.2390 | 280.2402 | 4 | 442.3303471 | 9Z,12E-octadecadienoic acid | C18H32O2 |  | 6.66 | 2.91E-07 |
| 37 | 280.2390 | 280.2402 | 4 | 442.3303471 | 9E,12Z-octadecadienoic acid | C18H32O2 |  | 6.66 | 2.91E-07 |
| 37 | 280.2390 | 280.2402 | 4 | 442.3303471 | 10Z,12Z-octadecadienoic acid | C18H32O2 |  | 6.66 | 2.91E-07 |
| 37 | 280.2390 | 280.2402 | 4 | 442.3303471 | 10E,12Z-Octadecadienoic acid | C18H32O2 |  | 6.66 | 2.91E-07 |
| 37 | 280.2390 | 280.2402 | 4 | 442.3303471 | 10E,12E-octadecadienoic acid | C18H32O2 |  | 6.66 | 2.91E-07 |
| 37 | 280.2390 | 280.2402 | 4 | 442.3303471 | 10Z,13Z-octadecadienoic acid | C18H32O2 |  | 6.66 | 2.91E-07 |
| 37 | 280.2390 | 280.2402 | 4 | 442.3303471 | 11E,14Z-octadecadienoic acid | C18H32O2 |  | 6.66 | 2.91E-07 |
| 37 | 280.2390 | 280.2402 | 4 | 442.3303471 | 10E,14E-octadecadienoic acid | C18H32O2 |  | 6.66 | 2.91E-07 |
| 37 | 280.2390 | 280.2402 | 4 | 442.3303471 | 10Z,14Z-octadecadienoic acid | C18H32O2 |  | 6.66 | 2.91E-07 |
| 37 | 280.2390 | 280.2402 | 4 | 442.3303471 | 11Z,14Z-octadecadienoic acid | C18H32O2 |  | 6.66 | 2.91E-07 |
| 37 | 280.2390 | 280.2402 | 4 | 442.3303471 | 11Z,15Z-octadecadienoic acid | C18H32O2 |  | 6.66 | 2.91E-07 |
| 37 | 280.2390 | 280.2402 | 4 | 442.3303471 | 12E,16E-octadecadienoic acid | C18H32O2 |  | 6.66 | 2.91E-07 |
| 37 | 280.2390 | 280.2402 | 4 | 442.3303471 | 12Z,15Z-octadecadienoic acid | C18H32O2 |  | 6.66 | 2.91E-07 |
| 37 | 280.2390 | 280.2402 | 4 | 442.3303471 | 13E,17-octadecadienoic acid | C18H32O2 |  | 6.66 | 2.91E-07 |
| 37 | 280.2390 | 280.2402 | 4 | 442.3303471 | 13Z,16Z-octadecadienoic acid | C18H32O2 |  | 6.66 | 2.91E-07 |
| 37 | 280.2390 | 280.2402 | 4 | 442.3303471 | 14Z,17-octadecadienoic acid | C18H32O2 |  | 6.66 | 2.91E-07 |
| 37 | 280.2390 | 280.2402 | 4 | 442.3303471 | 2,4-octadecadienoic acid | C18H32O2 |  | 6.66 | 2.91E-07 |
| 37 | 280.2390 | 280.2402 | 4 | 442.3303471 | 2E,6E-octadecadienoic acid | C18H32O2 |  | 6.66 | 2.91E-07 |
| 37 | 280.2390 | 280.2402 | 4 | 442.3303471 | 2Z,5Z-octadecadienoic acid | C18H32O2 |  | 6.66 | 2.91E-07 |
| 37 | 280.2390 | 280.2402 | 4 | 442.3303471 | 2Z,6Z-octadecadienoic acid | C18H32O2 |  | 6.66 | 2.91E-07 |
| 37 | 280.2390 | 280.2402 | 4 | 442.3303471 | 3E,7E-octadecadienoic acid | C18H32O2 |  | 6.66 | 2.91E-07 |
| 37 | 280.2390 | 280.2402 | 4 | 442.3303471 | 3Z,12Z-octadecadienoic acid | C18H32O2 |  | 6.66 | 2.91E-07 |
| 37 | 280.2390 | 280.2402 | 4 | 442.3303471 | 3Z,6Z-octadecadienoic acid | C18H32O2 |  | 6.66 | 2.91E-07 |
| 37 | 280.2390 | 280.2402 | 4 | 442.3303471 | 3Z,7Z-octadecadienoic acid | C18H32O2 |  | 6.66 | 2.91E-07 |
| 37 | 280.2390 | 280.2402 | 4 | 442.3303471 | 4,9-octadecadienoic acid | C18H32O2 |  | 6.66 | 2.91E-07 |
| 37 | 280.2390 | 280.2402 | 4 | 442.3303471 | 4E,8E-octadecadienoic acid | C18H32O2 |  | 6.66 | 2.91E-07 |
| 37 | 280.2390 | 280.2402 | 4 | 442.3303471 | 4Z,7Z-octadecadienoic acid | C18H32O2 |  | 6.66 | 2.91E-07 |
| 37 | 280.2390 | 280.2402 | 4 | 442.3303471 | 4Z,8Z-octadecadienoic acid | C18H32O2 |  | 6.66 | 2.91E-07 |
| 37 | 280.2390 | 280.2402 | 4 | 442.3303471 | 5,10-octadecadienoic acid | C18H32O2 |  | 6.66 | 2.91E-07 |
| 37 | 280.2390 | 280.2402 | 4 | 442.3303471 | 5,11-octadecadienoic acid | C18H32O2 |  | 6.66 | 2.91E-07 |
| 37 | 280.2390 | 280.2402 | 4 | 442.3303471 | 5,6-octadecadienoic acid | C18H32O2 |  | 6.66 | 2.91E-07 |
| 37 | 280.2390 | 280.2402 | 4 | 442.3303471 | 5E,9Z-octadecadienoic acid | C18H32O2 |  | 6.66 | 2.91E-07 |
| 37 | 280.2390 | 280.2402 | 4 | 442.3303471 | 5Z,11Z-octadecadienoic acid | C18H32O2 |  | 6.66 | 2.91E-07 |
| 37 | 280.2390 | 280.2402 | 4 | 442.3303471 | 5Z,8Z-octadecadienoic acid | C18H32O2 |  | 6.66 | 2.91E-07 |
| 37 | 280.2390 | 280.2402 | 4 | 442.3303471 | 5Z,9E-octadecadienoic acid | C18H32O2 |  | 6.66 | 2.91E-07 |
| 37 | 280.2390 | 280.2402 | 4 | 442.3303471 | 5Z,9Z-octadecadienoic acid | C18H32O2 |  | 6.66 | 2.91E-07 |
| 37 | 280.2390 | 280.2402 | 4 | 442.3303471 | 6,11-octadecadienoic acid | C18H32O2 |  | 6.66 | 2.91E-07 |
| 37 | 280.2390 | 280.2402 | 4 | 442.3303471 | 6E,10E-octadecadienoic acid | C18H32O2 |  | 6.66 | 2.91E-07 |
| 37 | 280.2390 | 280.2402 | 4 | 442.3303471 | 6E,11Z-octadecadienoic acid | C18H32O2 |  | 6.66 | 2.91E-07 |
| 37 | 280.2390 | 280.2402 | 4 | 442.3303471 | 6E,12E-octadecadienoic acid | C18H32O2 |  | 6.66 | 2.91E-07 |
| 37 | 280.2390 | 280.2402 | 4 | 442.3303471 | 6E,9E-octadecadienoic acid | C18H32O2 |  | 6.66 | 2.91E-07 |
| 37 | 280.2390 | 280.2402 | 4 | 442.3303471 | 6Z,11Z-octadecadienoic acid | C18H32O2 |  | 6.66 | 2.91E-07 |
| 37 | 280.2390 | 280.2402 | 4 | 442.3303471 | 6Z,9Z-octadecadienoic acid | C18H32O2 |  | 6.66 | 2.91E-07 |
| 37 | 280.2390 | 280.2402 | 4 | 442.3303471 | 7E,12E-octadecadienoic acid | C18H32O2 |  | 6.66 | 2.91E-07 |
| 37 | 280.2390 | 280.2402 | 4 | 442.3303471 | 7Z,10Z-octadecadienoic acid | C18H32O2 |  | 6.66 | 2.91E-07 |
| 37 | 280.2390 | 280.2402 | 4 | 442.3303471 | 7Z,11Z-octadecadienoic acid | C18H32O2 |  | 6.66 | 2.91E-07 |
| 37 | 280.2390 | 280.2402 | 4 | 442.3303471 | 8,11-octadecadienoic acid | C18H32O2 |  | 6.66 | 2.91E-07 |
| 37 | 280.2390 | 280.2402 | 4 | 442.3303471 | 8,12-octadecadienoic acid | C18H32O2 |  | 6.66 | 2.91E-07 |
| 37 | 280.2390 | 280.2402 | 4 | 442.3303471 | 9,13-octadecadienoic acid | C18H32O2 |  | 6.66 | 2.91E-07 |
| 37 | 280.2390 | 280.2402 | 4 | 442.3303471 | 6-octadecynoic acid | C18H32O2 |  | 6.66 | 2.91E-07 |
| 37 | 280.2390 | 280.2402 | 4 | 442.3303471 | 7-octadecynoic acid | C18H32O2 |  | 6.66 | 2.91E-07 |
| 37 | 280.2390 | 280.2402 | 4 | 442.3303471 | 12-octadecynoic acid | C18H32O2 |  | 6.66 | 2.91E-07 |
| 37 | 280.2390 | 280.2402 | 4 | 442.3303471 | 17-Octadecynoic Acid | C18H32O2 |  | 6.66 | 2.91E-07 |
| 37 | 280.2390 | 280.2402 | 4 | 442.3303471 | Linoleic acid | C18H32O2 | C01595 | 6.66 | 2.91E-07 |
| 37 | 280.2390 | 280.2402 | 4 | 442.3303471 | 9(Z),11(E)-Conjugated Linoleic Acid | C18H32O2 | C04056 | 6.66 | 2.91E-07 |
| 37 | 280.2390 | 280.2402 | 4 | 442.3303471 | Stearolic acid | C18H32O2 | C08459 | 6.66 | 2.91E-07 |
| 37 | 280.2390 | 280.2402 | 4 | 442.3303471 | Malvalic acid | C18H32O2 | C08321 | 6.66 | 2.91E-07 |
| 37 | 280.2390 | 280.2402 | 4 | 442.3303471 | 7-trans,9-cis-octadecadienoic acid | C18H32O2 |  | 6.66 | 2.91E-07 |
| 37 | 280.2390 | 280.2402 | 4 | 442.3303471 | Linoelaidic Acid | C18H32O2 |  | 6.66 | 2.91E-07 |
| 37 | 280.2390 | 280.2402 | 4 | 442.3303471 | Chaulmoogric acid | C18H32O2 | C08282 | 6.66 | 2.91E-07 |
| 37 | 280.2390 | 280.2402 | 4 | 442.3303471 | 16-methyl-6Z,9Z-heptadecadienoic acid | C18H32O2 |  | 6.66 | 2.91E-07 |
| 37 | 280.2390 | 280.2402 | 4 | 442.3303471 | 16-methyl-9Z,12Z-heptadecadienoic acid | C18H32O2 |  | 6.66 | 2.91E-07 |
| 37 | 280.2390 | 280.2402 | 4 | 442.3303471 | cis-5, cis-12-octadecadienoic acid; C18:2n-6,13 | C18H32O2 |  | 6.66 | 2.91E-07 |
| 37 | 280.2390 | 280.2402 | 4 | 442.3303471 | cis-5, trans-12-octadecadienoic acid; C18:2n-6,13 | C18H32O2 |  | 6.66 | 2.91E-07 |
| 37 | 280.2390 | 280.2402 | 4 | 442.3303471 | trans-5, cis12-octadecadienoic acid; C18:2n-6,13 | C18H32O2 |  | 6.66 | 2.91E-07 |
| 37 | 280.2390 | 280.2402 | 4 | 442.3303471 | trans-5, trans-12-octadecadienoic acid; C18:2n-6,13 | C18H32O2 |  | 6.66 | 2.91E-07 |
| 37 | 280.2390 | 280.2402 | 4 | 442.3303471 | trans-8, trans-10-octadecadienoic acid; C18:2n-8,10 | C18H32O2 |  | 6.66 | 2.91E-07 |
| 37 | 280.2390 | 280.2402 | 4 | 442.3303471 | cis-8, cis-11-octadecadienoic acid; C18:2n-7,10 | C18H32O2 |  | 6.66 | 2.91E-07 |
| 37 | 280.2390 | 280.2402 | 4 | 442.3303471 | cis-9, cis-11-octadecadienoic acid; C18:2n-7,9 | C18H32O2 |  | 6.66 | 2.91E-07 |
| 37 | 280.2390 | 280.2402 | 4 | 442.3303471 | trans-9, trans-11-octadecadienoic acid; C18:2n-7,9 | C18H32O2 |  | 6.66 | 2.91E-07 |
| 37 | 280.2390 | 280.2402 | 4 | 442.3303471 | cis-9, trans-12-octadecadienoic acid; C18:2n-6,9 | C18H32O2 |  | 6.66 | 2.91E-07 |
| 37 | 280.2390 | 280.2402 | 4 | 442.3303471 | trans-9, cis-12-octadecadienoic acid; C18:2n-6,9 | C18H32O2 |  | 6.66 | 2.91E-07 |
| 37 | 280.2390 | 280.2402 | 4 | 442.3303471 | cis-10, cis-12-octadecadienoic acid; C18:2n-6,8 | C18H32O2 |  | 6.66 | 2.91E-07 |
| 37 | 280.2390 | 280.2402 | 4 | 442.3303471 | trans-10, cis-12-octadecadienoic acid; C18:2n-6,8 | C18H32O2 |  | 6.66 | 2.91E-07 |
| 37 | 280.2390 | 280.2402 | 4 | 442.3303471 | trans-10, trans-12-octadecadienoic acid; C18:2n-6,8 | C18H32O2 |  | 6.66 | 2.91E-07 |
| 37 | 280.2390 | 280.2402 | 4 | 442.3303471 | cis-10, cis-13-octadecadienoic acid; C18:2n-5,8 | C18H32O2 |  | 6.66 | 2.91E-07 |
| 37 | 280.2390 | 280.2402 | 4 | 442.3303471 | 10E,14E-octadecadienoic acid | C18H32O2 |  | 6.66 | 2.91E-07 |
| 37 | 280.2390 | 280.2402 | 4 | 442.3303471 | 10Z,14Z-octadecadienoic acid | C18H32O2 |  | 6.66 | 2.91E-07 |
| 37 | 280.2390 | 280.2402 | 4 | 442.3303471 | 2E,6E-octadecadienoic acid | C18H32O2 |  | 6.66 | 2.91E-07 |
| 37 | 280.2390 | 280.2402 | 4 | 442.3303471 | 2Z,6Z-octadecadienoic acid | C18H32O2 |  | 6.66 | 2.91E-07 |
| 37 | 280.2390 | 280.2402 | 4 | 442.3303471 | 3E,7E-octadecadienoic acid | C18H32O2 |  | 6.66 | 2.91E-07 |
| 37 | 280.2390 | 280.2402 | 4 | 442.3303471 | 3Z,7Z-octadecadienoic acid | C18H32O2 |  | 6.66 | 2.91E-07 |
| 37 | 280.2390 | 280.2402 | 4 | 442.3303471 | 4E,8E-octadecadienoic acid | C18H32O2 |  | 6.66 | 2.91E-07 |
| 37 | 280.2390 | 280.2402 | 4 | 442.3303471 | 4Z,8Z-octadecadienoic acid | C18H32O2 |  | 6.66 | 2.91E-07 |
| 37 | 280.2390 | 280.2402 | 4 | 442.3303471 | 5,11-octadecadienoic acid | C18H32O2 |  | 6.66 | 2.91E-07 |
| 37 | 280.2390 | 280.2402 | 4 | 442.3303471 | 5E,9Z-octadecadienoic acid | C18H32O2 |  | 6.66 | 2.91E-07 |
| 37 | 280.2390 | 280.2402 | 4 | 442.3303471 | 5Z,11Z-octadecadienoic acid | C18H32O2 |  | 6.66 | 2.91E-07 |
| 37 | 280.2390 | 280.2402 | 4 | 442.3303471 | Sebaleic acid | C18H32O2 |  | 6.66 | 2.91E-07 |
| 37 | 280.2390 | 280.2402 | 4 | 442.3303471 | 5Z,9E-octadecadienoic acid | C18H32O2 |  | 6.66 | 2.91E-07 |
| 37 | 280.2390 | 280.2402 | 4 | 442.3303471 | Taxoleic acid | C18H32O2 |  | 6.66 | 2.91E-07 |
| 37 | 280.2390 | 280.2402 | 4 | 442.3303471 | 6,11-octadecadienoic acid | C18H32O2 |  | 6.66 | 2.91E-07 |
| 37 | 280.2390 | 280.2402 | 4 | 442.3303471 | 6E,11Z-octadecadienoic acid | C18H32O2 |  | 6.66 | 2.91E-07 |
| 37 | 280.2390 | 280.2402 | 4 | 442.3303471 | 6Z,11Z-octadecadienoic acid | C18H32O2 |  | 6.66 | 2.91E-07 |
| 37 | 280.2390 | 280.2402 | 4 | 442.3303471 | cis,cis-6,9-octadecadienoic acid; C18:2n-9,12 | C18H32O2 |  | 6.66 | 2.91E-07 |
| 37 | 280.2390 | 280.2402 | 4 | 442.3303471 | 6-Octadecynoic acid; Tariric acid; 6,7-Stearolic acid; 6-Stearolic acid | C18H32O2 |  | 6.66 | 2.91E-07 |
| 37 | 280.2390 | 280.2402 | 4 | 442.3303471 | 8-Octadecynoic acid | C18H32O2 |  | 6.66 | 2.91E-07 |
| 37 | 280.2390 | 280.2402 | 4 | 442.3303471 | 2-Octadecynoic acid | C18H32O2 |  | 6.66 | 2.91E-07 |
| 37 | 280.2390 | 280.2402 | 4 | 442.3303471 | 3-Octadecynoic acid | C18H32O2 |  | 6.66 | 2.91E-07 |
| 37 | 280.2390 | 280.2402 | 4 | 442.3303471 | 4-Octadecynoic acid | C18H32O2 |  | 6.66 | 2.91E-07 |
| 37 | 280.2390 | 280.2402 | 4 | 442.3303471 | 5-Octadecynoic acid | C18H32O2 |  | 6.66 | 2.91E-07 |
| 37 | 280.2390 | 280.2402 | 4 | 442.3303471 | 10-Octadecynoic acid | C18H32O2 |  | 6.66 | 2.91E-07 |
| 37 | 280.2390 | 280.2402 | 4 | 442.3303471 | 11-Octadecynoic acid | C18H32O2 |  | 6.66 | 2.91E-07 |
| 37 | 280.2390 | 280.2402 | 4 | 442.3303471 | 12-Octadecynoic acid; 12-Stearolic acid | C18H32O2 |  | 6.66 | 2.91E-07 |
| 37 | 280.2390 | 280.2402 | 4 | 442.3303471 | 13-Octadecynoic acid | C18H32O2 |  | 6.66 | 2.91E-07 |
| 37 | 280.2390 | 280.2402 | 4 | 442.3303471 | 14-Octadecynoic acid | C18H32O2 |  | 6.66 | 2.91E-07 |
| 37 | 280.2390 | 280.2402 | 4 | 442.3303471 | 15-Octadecynoic acid | C18H32O2 |  | 6.66 | 2.91E-07 |
| 37 | 280.2390 | 280.2402 | 4 | 442.3303471 | 16-Octadecynoic acid | C18H32O2 |  | 6.66 | 2.91E-07 |
| 37 | 280.2390 | 280.2402 | 4 | 442.3303471 | (R)-laballenic acid | C18H32O2 |  | 6.66 | 2.91E-07 |
| 37 | 280.2390 | 280.2402 | 4 | 442.3303471 | (S)-laballenic acid | C18H32O2 |  | 6.66 | 2.91E-07 |
| 37 | 280.2390 | 280.2402 | 4 | 442.3303471 | Mangiferic acid | C18H32O2 |  | 6.66 | 2.91E-07 |
| 38 | 254.2225 | 254.2246 | 7 | 416.3138822 | Gaidic acid | C16H30O2 |  | 2.33 | 1.54E-04 |
| 38 | 254.2225 | 254.2246 | 7 | 416.3138822 | 7-palmitoleic acid | C16H30O2 |  | 2.33 | 1.54E-04 |
| 38 | 254.2225 | 254.2246 | 7 | 416.3138822 | cis-10-palmitoleic acid | C16H30O2 |  | 2.33 | 1.54E-04 |
| 38 | 254.2225 | 254.2246 | 7 | 416.3138822 | 10-hexadecenoic acid | C16H30O2 |  | 2.33 | 1.54E-04 |
| 38 | 254.2225 | 254.2246 | 7 | 416.3138822 | 11-hexadecenoic acid | C16H30O2 |  | 2.33 | 1.54E-04 |
| 38 | 254.2225 | 254.2246 | 7 | 416.3138822 | 11Z-hexadecenoic acid | C16H30O2 |  | 2.33 | 1.54E-04 |
| 38 | 254.2225 | 254.2246 | 7 | 416.3138822 | 13-hexadecenoic acid | C16H30O2 |  | 2.33 | 1.54E-04 |
| 38 | 254.2225 | 254.2246 | 7 | 416.3138822 | 13Z-hexadecenoic acid | C16H30O2 |  | 2.33 | 1.54E-04 |
| 38 | 254.2225 | 254.2246 | 7 | 416.3138822 | 3E-hexadecenoic acid | C16H30O2 |  | 2.33 | 1.54E-04 |
| 38 | 254.2225 | 254.2246 | 7 | 416.3138822 | 6Z-hexadecenoic acid | C16H30O2 |  | 2.33 | 1.54E-04 |
| 38 | 254.2225 | 254.2246 | 8 | 416.3138822 | cis-7-Hexadecenoic Acid | C16H30O2 |  | 2.33 | 1.54E-04 |
| 38 | 254.2225 | 254.2246 | 8 | 416.3138822 | cis-9-palmitoleic acid | C16H30O2 | C08362 | 2.33 | 1.54E-04 |
| 38 | 254.2225 | 254.2246 | 8 | 416.3138822 | trans-9-palmitoleic acid | C16H30O2 | C08362 | 2.33 | 1.54E-04 |
| 38 | 254.2225 | 254.2246 | 8 | 416.3138822 | Hypogeic acid | C16H30O2 |  | 2.33 | 1.54E-04 |
| 38 | 254.2225 | 254.2246 | 8 | 416.3138822 | 2,4-dimethyl-2E-tetradecenoic acid | C16H30O2 |  | 2.33 | 1.54E-04 |
| 38 | 254.2225 | 254.2246 | 8 | 416.3138822 | 14-methyl-4-pentadecenoic acid | C16H30O2 |  | 2.33 | 1.54E-04 |
| 38 | 254.2225 | 254.2246 | 8 | 416.3138822 | 2-hexyl-2-decenoic acid | C16H30O2 |  | 2.33 | 1.54E-04 |
| 38 | 254.2225 | 254.2246 | 8 | 416.3138822 | 6-isopentyl-9-methyl-5-decenoic acid | C16H30O2 |  | 2.33 | 1.54E-04 |
| 38 | 254.2225 | 254.2246 | 8 | 416.3138822 | cis-Palmitvaccenic acid | C16H30O2 |  | 2.33 | 1.54E-04 |
| 38 | 254.2225 | 254.2246 | 8 | 416.3138822 | 13-hexadecenoic acid | C16H30O2 |  | 2.33 | 1.54E-04 |
| 38 | 254.2225 | 254.2246 | 8 | 416.3138822 | 13Z-hexadecenoic acid | C16H30O2 |  | 2.33 | 1.54E-04 |
| 38 | 254.2225 | 254.2246 | 8 | 416.3138822 | 3E-Hexadecenoic acid | C16H30O2 |  | 2.33 | 1.54E-04 |
| 38 | 254.2225 | 254.2246 | 8 | 416.3138822 | Sapienic acid | C16H30O2 |  | 2.33 | 1.54E-04 |
| 38 | 254.2225 | 254.2246 | 8 | 416.3138822 | hexadec-7Z-enoic acid | C16H30O2 |  | 2.33 | 1.54E-04 |
| 38 | 254.2225 | 254.2246 | 8 | 416.3138822 | (Z)-5-Hexadecenoic acid | C16H30O2 |  | 2.33 | 1.54E-04 |
| 38 | 254.2225 | 254.2246 | 8 | 416.3138822 | (E)-3-Hexadecenoic acid | C16H30O2 |  | 2.33 | 1.54E-04 |
| 38 | 254.2225 | 254.2246 | 8 | 416.3138822 | (Z)-14-Methyl-6-pentadecenoic acid | C16H30O2 |  | 2.33 | 1.54E-04 |
| 38 | 254.2225 | 254.2246 | 8 | 416.3138822 | &Delta;2-trans-Hexadecenoic Acid | C16H30O2 |  | 2.33 | 1.54E-04 |
| 38 | 254.2225 | 254.2246 | 8 | 416.3138822 | &Delta;2-cis-Hexadecenoic Acid | C16H30O2 |  | 2.33 | 1.54E-04 |
| 39 | 306.2539 | 306.2559 | 6 | 468.3452465 | 5,8,11-eicosatrienoic acid | C20H34O2 |  | 3.28 | 2.61E-06 |
| 39 | 306.2539 | 306.2559 | 6 | 468.3452465 | 11,14,17-eicosatrienoic acid | C20H34O2 |  | 3.28 | 2.61E-06 |
| 39 | 306.2539 | 306.2559 | 6 | 468.3452465 | Podocarpric acid | C20H34O2 |  | 3.28 | 2.61E-06 |
| 39 | 306.2539 | 306.2559 | 6 | 468.3452465 | 2E,4E,8Z-eicosatrienoic acid | C20H34O2 |  | 3.28 | 2.61E-06 |
| 39 | 306.2539 | 306.2559 | 6 | 468.3452465 | 5(Z),11(Z),14(Z)-Eicosatrienoic acid | C20H34O2 |  | 3.28 | 2.61E-06 |
| 39 | 306.2539 | 306.2559 | 6 | 468.3452465 | 5(Z),8(Z),11(Z)-Eicosatrienoic Acid | C20H34O2 |  | 3.28 | 2.61E-06 |
| 39 | 306.2539 | 306.2559 | 6 | 468.3452465 | 7,10,13-Eicosatrienoic acid | C20H34O2 |  | 3.28 | 2.61E-06 |
| 39 | 306.2539 | 306.2559 | 6 | 468.3452465 | 7,11,14-Eicosatrienoic acid | C20H34O2 |  | 3.28 | 2.61E-06 |
| 39 | 306.2539 | 306.2559 | 6 | 468.3452465 | 7Z,10Z,13Z-eicosatrienoic acid | C20H34O2 |  | 3.28 | 2.61E-06 |
| 39 | 306.2539 | 306.2559 | 6 | 468.3452465 | 7Z,11Z,14E-eicosatrienoic acid | C20H34O2 |  | 3.28 | 2.61E-06 |
| 39 | 306.2539 | 306.2559 | 6 | 468.3452465 | 7Z,11Z,14Z-eicosatrienoic acid | C20H34O2 |  | 3.28 | 2.61E-06 |
| 39 | 306.2539 | 306.2559 | 6 | 468.3452465 | 8Z,12E,14Z-eicosatrienoic acid | C20H34O2 |  | 3.28 | 2.61E-06 |
| 39 | 306.2539 | 306.2559 | 6 | 468.3452465 | Eicosatrienoic acid | C20H34O2 |  | 3.28 | 2.61E-06 |
| 39 | 306.2539 | 306.2559 | 6 | 468.3452465 | Linolenic Acid ethyl ester | C20H34O2 |  | 3.28 | 2.61E-06 |
| 39 | 306.2539 | 306.2559 | 6 | 468.3452465 | 9(Z),11(E),13(E)-Octadecatrienoic Acid ethyl ester | C20H34O2 |  | 3.28 | 2.61E-06 |
| 39 | 306.2539 | 306.2559 | 6 | 468.3452465 | Dihomo-&gamma;-Linolenic Acid | C20H34O2 | C03242 | 3.28 | 2.61E-06 |
| 39 | 306.2539 | 306.2559 | 6 | 468.3452465 | 11(Z),14(Z),17(Z)-Eicosatrienoic Acid | C20H34O2 | C16522 | 3.28 | 2.61E-06 |
| 39 | 306.2539 | 306.2559 | 6 | 468.3452465 | Pinolenic Acid ethyl ester | C20H34O2 |  | 3.28 | 2.61E-06 |
| 39 | 306.2539 | 306.2559 | 6 | 468.3452465 | 5(Z),8(Z),14(Z)-Eicosatrienoic Acid | C20H34O2 |  | 3.28 | 2.61E-06 |
| 39 | 306.2539 | 306.2559 | 6 | 468.3452465 | (5Z,9E,14Z)-icosa-5,9,14-trienoic acid | C20H34O2 |  | 3.28 | 2.61E-06 |
| 39 | 306.2539 | 306.2559 | 6 | 468.3452465 | Oncobic acid | C20H34O2 |  | 3.28 | 2.61E-06 |
| 39 | 306.2539 | 306.2559 | 6 | 468.3452465 | 18-methyl-8Z,11Z,14Z-nonadecatrienoic acid | C20H34O2 |  | 3.28 | 2.61E-06 |
| 39 | 306.2539 | 306.2559 | 6 | 468.3452465 | 5, 8, 11-icosatrienoic acid; C20:3n-9,12,15 | C20H34O2 |  | 3.28 | 2.61E-06 |
| 39 | 306.2539 | 306.2559 | 6 | 468.3452465 | 11, 14, 17-icosatrienoic acid; C20:3n-3,6,9 | C20H34O2 |  | 3.28 | 2.61E-06 |
| 39 | 306.2539 | 306.2559 | 6 | 468.3452465 | Sciadonic acid | C20H34O2 |  | 3.28 | 2.61E-06 |
| 39 | 306.2539 | 306.2559 | 6 | 468.3452465 | 7,10,13-Eicosatrienoic acid | C20H34O2 |  | 3.28 | 2.61E-06 |
| 39 | 306.2539 | 306.2559 | 6 | 468.3452465 | 7,11,14-Eicosatrienoic acid | C20H34O2 |  | 3.28 | 2.61E-06 |
| 39 | 306.2539 | 306.2559 | 6 | 468.3452465 | 7Z,10Z,13Z-eicosatrienoic acid | C20H34O2 |  | 3.28 | 2.61E-06 |
| 39 | 306.2539 | 306.2559 | 6 | 468.3452465 | 7Z,11Z,14E-eicosatrienoic acid | C20H34O2 |  | 3.28 | 2.61E-06 |
| 39 | 306.2539 | 306.2559 | 6 | 468.3452465 | 7Z,11Z,14Z-eicosatrienoic acid | C20H34O2 |  | 3.28 | 2.61E-06 |
| 39 | 306.2539 | 306.2559 | 6 | 468.3452465 | eicosa-5Z,8Z,14Z-trienoic acid | C20H34O2 |  | 3.28 | 2.61E-06 |
| 39 | 306.2539 | 306.2559 | 6 | 468.3452465 | &gamma;-Linolenic Acid ethyl ester | C20H34O2 |  | 3.28 | 2.61E-06 |
| 40 | 268.2391 | 268.2402 | 4 | 430.3303976 | 10-methyl-9-hexadecenoic acid | C17H32O2 |  | 4.78 | 5.29E-05 |
| 40 | 268.2391 | 268.2402 | 4 | 430.3303976 | 14R-methyl-8Z-hexadecenoic acid | C17H32O2 |  | 4.78 | 5.29E-05 |
| 40 | 268.2391 | 268.2402 | 4 | 430.3303976 | 14S-methyl-8Z-hexadecenoic acid | C17H32O2 |  | 4.78 | 5.29E-05 |
| 40 | 268.2391 | 268.2402 | 4 | 430.3303976 | 2-heptadecylenic acid | C17H32O2 |  | 4.78 | 5.29E-05 |
| 40 | 268.2391 | 268.2402 | 4 | 430.3303976 | 10E-heptadecenoic acid | C17H32O2 |  | 4.78 | 5.29E-05 |
| 40 | 268.2391 | 268.2402 | 4 | 430.3303976 | 10Z-heptadecenoic acid | C17H32O2 |  | 4.78 | 5.29E-05 |
| 40 | 268.2391 | 268.2402 | 4 | 430.3303976 | 16-heptadecenoic acid | C17H32O2 |  | 4.78 | 5.29E-05 |
| 40 | 268.2391 | 268.2402 | 4 | 430.3303976 | 2Z-heptadecenoic acid | C17H32O2 |  | 4.78 | 5.29E-05 |
| 40 | 268.2391 | 268.2402 | 4 | 430.3303976 | 7Z-heptadecenoic acid | C17H32O2 |  | 4.78 | 5.29E-05 |
| 40 | 268.2391 | 268.2402 | 4 | 430.3303976 | 8E-heptadecenoic acid | C17H32O2 |  | 4.78 | 5.29E-05 |
| 40 | 268.2391 | 268.2402 | 4 | 430.3303976 | 8Z-heptadecenoic acid | C17H32O2 |  | 4.78 | 5.29E-05 |
| 40 | 268.2391 | 268.2402 | 4 | 430.3303976 | 3-heptadecenoic acid | C17H32O2 |  | 4.78 | 5.29E-05 |
| 40 | 268.2391 | 268.2402 | 4 | 430.3303976 | 7-heptadecenoic acid | C17H32O2 |  | 4.78 | 5.29E-05 |
| 40 | 268.2391 | 268.2402 | 4 | 430.3303976 | cis-7-Hexadecenoic Acid methyl ester | C17H32O2 |  | 4.78 | 5.29E-05 |
| 40 | 268.2391 | 268.2402 | 4 | 430.3303976 | 9-heptadecylenic acid | C17H32O2 | C16536 | 4.78 | 5.29E-05 |
| 40 | 268.2391 | 268.2402 | 4 | 430.3303976 | 9E-heptadecenoic acid | C17H32O2 | C16536 | 4.78 | 5.29E-05 |
| 40 | 268.2391 | 268.2402 | 4 | 430.3303976 | 11-Cyclohexylundecanoic acid | C17H32O2 | C12100 | 4.78 | 5.29E-05 |
| 40 | 268.2391 | 268.2402 | 4 | 430.3303976 | 7-methyl-6E-hexadecenoic acid | C17H32O2 |  | 4.78 | 5.29E-05 |
| 40 | 268.2391 | 268.2402 | 4 | 430.3303976 | 14-methyl-8E-hexadecenoic acid | C17H32O2 |  | 4.78 | 5.29E-05 |
| 40 | 268.2391 | 268.2402 | 4 | 430.3303976 | 9-Hexadecenoic acid, 10-methyl- | C17H32O2 |  | 4.78 | 5.29E-05 |
| 40 | 268.2391 | 268.2402 | 4 | 430.3303976 | 8-Hexadecenoic acid, 14-methyl-, [R-(Z)]- | C17H32O2 |  | 4.78 | 5.29E-05 |
| 40 | 268.2391 | 268.2402 | 4 | 430.3303976 | 8-Hexadecenoic acid, 14-methyl-, [S-(Z)]- | C17H32O2 |  | 4.78 | 5.29E-05 |
| 40 | 268.2391 | 268.2402 | 4 | 430.3303976 | 10E-heptadecenoic acid | C17H32O2 |  | 4.78 | 5.29E-05 |
| 40 | 268.2391 | 268.2402 | 4 | 430.3303976 | 10Z-heptadecenoic acid | C17H32O2 |  | 4.78 | 5.29E-05 |
| 40 | 268.2391 | 268.2402 | 4 | 430.3303976 | 8E-heptadecenoic acid | C17H32O2 |  | 4.78 | 5.29E-05 |
| 40 | 268.2391 | 268.2402 | 4 | 430.3303976 | 8Z-heptadecenoic acid | C17H32O2 |  | 4.78 | 5.29E-05 |
| 40 | 268.2391 | 268.2402 | 4 | 430.3303976 | 3-Heptadecenoic acid; C17:1n-14 | C17H32O2 |  | 4.78 | 5.29E-05 |
| 40 | 268.2391 | 268.2402 | 4 | 430.3303976 | 7-Heptadecenoic acid; C17:1n-10 | C17H32O2 |  | 4.78 | 5.29E-05 |
| 40 | 268.2391 | 268.2402 | 4 | 430.3303976 | 9,10-methanohexadecanoic acid | C17H32O2 |  | 4.78 | 5.29E-05 |
| 41 | 300.2630 | 300.2664 | 11 | 462.3543284 | DL-2-hydroxy stearic acid | C18H36O3 |  | 2.32 | 2.63E-06 |
| 41 | 300.2630 | 300.2664 | 11 | 462.3543284 | DL-3-hydroxy stearic acid | C18H36O3 |  | 2.32 | 2.63E-06 |
| 41 | 300.2630 | 300.2664 | 11 | 462.3543284 | DL-4-hydroxy stearic acid | C18H36O3 |  | 2.32 | 2.63E-06 |
| 41 | 300.2630 | 300.2664 | 11 | 462.3543284 | DL-5-hydroxy stearic acid | C18H36O3 |  | 2.32 | 2.63E-06 |
| 41 | 300.2630 | 300.2664 | 11 | 462.3543284 | DL-6-hydroxy stearic acid | C18H36O3 |  | 2.32 | 2.63E-06 |
| 41 | 300.2630 | 300.2664 | 11 | 462.3543284 | DL-7-hydroxy stearic acid | C18H36O3 |  | 2.32 | 2.63E-06 |
| 41 | 300.2630 | 300.2664 | 11 | 462.3543284 | DL-8-hydroxy stearic acid | C18H36O3 |  | 2.32 | 2.63E-06 |
| 41 | 300.2630 | 300.2664 | 11 | 462.3543284 | DL-9-hydroxy stearic acid | C18H36O3 |  | 2.32 | 2.63E-06 |
| 41 | 300.2630 | 300.2664 | 11 | 462.3543284 | DL-10-hydroxy stearic acid | C18H36O3 |  | 2.32 | 2.63E-06 |
| 41 | 300.2630 | 300.2664 | 11 | 462.3543284 | DL-11-hydroxy stearic acid | C18H36O3 |  | 2.32 | 2.63E-06 |
| 41 | 300.2630 | 300.2664 | 11 | 462.3543284 | DL-12-hydroxy stearic acid | C18H36O3 |  | 2.32 | 2.63E-06 |
| 41 | 300.2630 | 300.2664 | 11 | 462.3543284 | DL-13-hydroxy stearic acid | C18H36O3 |  | 2.32 | 2.63E-06 |
| 41 | 300.2630 | 300.2664 | 11 | 462.3543284 | 14-hydroxy stearic acid | C18H36O3 |  | 2.32 | 2.63E-06 |
| 41 | 300.2630 | 300.2664 | 11 | 462.3543284 | 15-hydroxy stearic acid | C18H36O3 |  | 2.32 | 2.63E-06 |
| 41 | 300.2630 | 300.2664 | 11 | 462.3543284 | 16-hydroxy stearic acid | C18H36O3 |  | 2.32 | 2.63E-06 |
| 41 | 300.2630 | 300.2664 | 11 | 462.3543284 | 17-hydroxy stearic acid | C18H36O3 |  | 2.32 | 2.63E-06 |
| 41 | 300.2630 | 300.2664 | 11 | 462.3543284 | 18-hydroxy stearic acid | C18H36O3 |  | 2.32 | 2.63E-06 |
| 41 | 300.2630 | 300.2664 | 11 | 462.3543284 | 2S-hydroxy-octadecanoic acid | C18H36O3 | C03045 | 2.32 | 2.63E-06 |
| 41 | 300.2630 | 300.2664 | 11 | 462.3543284 | 2R-hydroxy-stearic acid | C18H36O3 | C03042 | 2.32 | 2.63E-06 |
| 41 | 300.2630 | 300.2664 | 11 | 462.3543284 | (R)-10-hydroxystearic acid | C18H36O3 | C03195 | 2.32 | 2.63E-06 |
| 41 | 300.2630 | 300.2664 | 11 | 462.3543284 | 3-hydroxy-16-methyl-heptadecanoic acid | C18H36O3 |  | 2.32 | 2.63E-06 |
| 41 | 300.2630 | 300.2664 | 11 | 462.3543284 | 3R-hydroxy-octadecanoic acid | C18H36O3 |  | 2.32 | 2.63E-06 |
| 41 | 300.2630 | 300.2664 | 11 | 462.3543284 | 9R-hydroxy-octadecanoic acid | C18H36O3 |  | 2.32 | 2.63E-06 |
| 41 | 300.2630 | 300.2664 | 11 | 462.3543284 | 9S-hydroxy-octadecanoic acid | C18H36O3 |  | 2.32 | 2.63E-06 |
| 41 | 300.2630 | 300.2664 | 11 | 462.3543284 | 12R-hydroxy-octadecanoic acid | C18H36O3 |  | 2.32 | 2.63E-06 |
| 41 | 300.2630 | 300.2664 | 11 | 462.3543284 | 13R-hydroxy-octadecanoic acid | C18H36O3 |  | 2.32 | 2.63E-06 |
| 41 | 300.2630 | 300.2664 | 11 | 462.3543284 | 9-methoxy-heptadecanoic acid | C18H36O3 |  | 2.32 | 2.63E-06 |
| 42 | 340.2600 | 340.2614 | 3 | 502.3513525 | Thromboxanoic acid skeleton | C20H36O4 |  | 4.85 | 4.40E-06 |
| 43 | 282.2548 | 282.2559 | 3 | 444.3461873 | 2Z-octadecenoic acid | C18H34O2 |  | 12.68 | 1.95E-07 |
| 43 | 282.2548 | 282.2559 | 3 | 444.3461873 | trans-2-oleic acid | C18H34O2 |  | 12.68 | 1.95E-07 |
| 43 | 282.2548 | 282.2559 | 3 | 444.3461873 | 3-octadecylenic acid | C18H34O2 |  | 12.68 | 1.95E-07 |
| 43 | 282.2548 | 282.2559 | 3 | 444.3461873 | 4-octadecylenic acid | C18H34O2 |  | 12.68 | 1.95E-07 |
| 43 | 282.2548 | 282.2559 | 3 | 444.3461873 | 5-octadecylenic acid | C18H34O2 |  | 12.68 | 1.95E-07 |
| 43 | 282.2548 | 282.2559 | 3 | 444.3461873 | Petroselaidic acid | C18H34O2 |  | 12.68 | 1.95E-07 |
| 43 | 282.2548 | 282.2559 | 3 | 444.3461873 | 7Z-octadecenoic acid | C18H34O2 |  | 12.68 | 1.95E-07 |
| 43 | 282.2548 | 282.2559 | 3 | 444.3461873 | 7E-octadecenoic acid | C18H34O2 |  | 12.68 | 1.95E-07 |
| 43 | 282.2548 | 282.2559 | 3 | 444.3461873 | cis-8-oleic acid | C18H34O2 |  | 12.68 | 1.95E-07 |
| 43 | 282.2548 | 282.2559 | 3 | 444.3461873 | trans-8-elaidic acid | C18H34O2 |  | 12.68 | 1.95E-07 |
| 43 | 282.2548 | 282.2559 | 3 | 444.3461873 | cis-10-oleic acid | C18H34O2 |  | 12.68 | 1.95E-07 |
| 43 | 282.2548 | 282.2559 | 3 | 444.3461873 | 10E-octadecenoic acid | C18H34O2 |  | 12.68 | 1.95E-07 |
| 43 | 282.2548 | 282.2559 | 3 | 444.3461873 | cis-12-oleic acid | C18H34O2 |  | 12.68 | 1.95E-07 |
| 43 | 282.2548 | 282.2559 | 3 | 444.3461873 | trans-12-elaidic acid | C18H34O2 |  | 12.68 | 1.95E-07 |
| 43 | 282.2548 | 282.2559 | 3 | 444.3461873 | 15E-octadecenoic acid | C18H34O2 |  | 12.68 | 1.95E-07 |
| 43 | 282.2548 | 282.2559 | 3 | 444.3461873 | 16E-octadecenoic acid | C18H34O2 |  | 12.68 | 1.95E-07 |
| 43 | 282.2548 | 282.2559 | 3 | 444.3461873 | 13Z-octadecenoic acid | C18H34O2 |  | 12.68 | 1.95E-07 |
| 43 | 282.2548 | 282.2559 | 3 | 444.3461873 | 15Z-octadecenoic acid | C18H34O2 |  | 12.68 | 1.95E-07 |
| 43 | 282.2548 | 282.2559 | 3 | 444.3461873 | 16Z-octadecenoic acid | C18H34O2 |  | 12.68 | 1.95E-07 |
| 43 | 282.2548 | 282.2559 | 3 | 444.3461873 | 17-octadecenoic acid | C18H34O2 |  | 12.68 | 1.95E-07 |
| 43 | 282.2548 | 282.2559 | 3 | 444.3461873 | 3Z-octadecenoic acid | C18H34O2 |  | 12.68 | 1.95E-07 |
| 43 | 282.2548 | 282.2559 | 3 | 444.3461873 | 4Z-octadecenoic acid | C18H34O2 |  | 12.68 | 1.95E-07 |
| 43 | 282.2548 | 282.2559 | 3 | 444.3461873 | 5Z-octadecenoic acid | C18H34O2 |  | 12.68 | 1.95E-07 |
| 43 | 282.2548 | 282.2559 | 3 | 444.3461873 | Palmitoleic Acid ethyl ester | C18H34O2 |  | 12.68 | 1.95E-07 |
| 43 | 282.2548 | 282.2559 | 3 | 444.3461873 | Oleic Acid | C18H34O2 | C00712 | 12.68 | 1.95E-07 |
| 43 | 282.2548 | 282.2559 | 3 | 444.3461873 | Elaidic Acid | C18H34O2 | C01712 | 12.68 | 1.95E-07 |
| 43 | 282.2548 | 282.2559 | 3 | 444.3461873 | Vaccenic acid | C18H34O2 | C08367 | 12.68 | 1.95E-07 |
| 43 | 282.2548 | 282.2559 | 3 | 444.3461873 | cis-vaccenic acid | C18H34O2 | C08367 | 12.68 | 1.95E-07 |
| 43 | 282.2548 | 282.2559 | 3 | 444.3461873 | Petroselinic acid | C18H34O2 | C08363 | 12.68 | 1.95E-07 |
| 43 | 282.2548 | 282.2559 | 3 | 444.3461873 | 11-Cycloheptylundecanoic acid | C18H34O2 | C12103 | 12.68 | 1.95E-07 |
| 43 | 282.2548 | 282.2559 | 3 | 444.3461873 | 2-methyl-16-heptadecenoic acid | C18H34O2 |  | 12.68 | 1.95E-07 |
| 43 | 282.2548 | 282.2559 | 3 | 444.3461873 | cis-2-octadecenoic acid; C18:1n-16 | C18H34O2 |  | 12.68 | 1.95E-07 |
| 43 | 282.2548 | 282.2559 | 3 | 444.3461873 | cis-7-octadecenoic acid; C18:1n-11 | C18H34O2 |  | 12.68 | 1.95E-07 |
| 43 | 282.2548 | 282.2559 | 3 | 444.3461873 | trans-7-octadecenoic acid; C18:1n-11 | C18H34O2 |  | 12.68 | 1.95E-07 |
| 43 | 282.2548 | 282.2559 | 3 | 444.3461873 | trans-10-octadecenoic acid; C18:1n-8 | C18H34O2 |  | 12.68 | 1.95E-07 |
| 43 | 282.2548 | 282.2559 | 3 | 444.3461873 | trans-15-octadecenoic acid; C18:1n-3 | C18H34O2 |  | 12.68 | 1.95E-07 |
| 43 | 282.2548 | 282.2559 | 3 | 444.3461873 | trans-16-octadecenoic acid; C18:1n-2 | C18H34O2 |  | 12.68 | 1.95E-07 |
| 44 | 326.2803 | 326.2821 | 5 | 488.3715985 | Lesquerolic acid | C20H38O3 |  | 2.22 | 3.20E-06 |
| 44 | 326.2803 | 326.2821 | 5 | 488.3715985 | 14R-hydroxy-11E-eicosenoic acid | C20H38O3 |  | 2.22 | 3.20E-06 |
| 44 | 326.2803 | 326.2821 | 5 | 488.3715985 | 14R-hydroxy-11Z-eicosenoic acid | C20H38O3 |  | 2.22 | 3.20E-06 |
| 44 | 326.2803 | 326.2821 | 5 | 488.3715985 | 2-oxophytanic acid | C20H38O3 | C02117 | 2.22 | 3.20E-06 |
| 44 | 326.2803 | 326.2821 | 5 | 488.3715985 | 11-Eicosenoic acid, 14-hydroxy-, [R-(E)]- | C20H38O3 |  | 2.22 | 3.20E-06 |
| 44 | 326.2803 | 326.2821 | 5 | 488.3715985 | 11-Eicosenoic acid, 14-hydroxy-, [R-(Z)]-; 11-Eicosenoic acid, 14-hydroxy-, (Z)-D-(+)-; Lesquerolic acid | C20H38O3 |  | 2.22 | 3.20E-06 |
| 44 | 326.2803 | 326.2821 | 5 | 488.3715985 | 19-oxo-eicosanoic acid | C20H38O3 |  | 2.22 | 3.20E-06 |
| 44 | 326.2803 | 326.2821 | 5 | 488.3715985 | 2-oxo-eicosanoic acid | C20H38O3 |  | 2.22 | 3.20E-06 |
| 44 | 326.2803 | 326.2821 | 5 | 488.3715985 | 3-oxo-eicosanoic acid | C20H38O3 |  | 2.22 | 3.20E-06 |
| 45 | 308.2689 | 308.2715 | 8 | 470.3602106 | Eicosa-5Z,8Z-dienoic acid (20:2, n-12) | C20H36O2 |  | 2.95 | 7.78E-07 |
| 45 | 308.2689 | 308.2715 | 8 | 470.3602106 | 11Z,15Z-eicosadienoic acid | C20H36O2 |  | 2.95 | 7.78E-07 |
| 45 | 308.2689 | 308.2715 | 8 | 470.3602106 | 5,11-Eicosadienoic acid | C20H36O2 |  | 2.95 | 7.78E-07 |
| 45 | 308.2689 | 308.2715 | 8 | 470.3602106 | 7,13-Eicosadienoic acid | C20H36O2 |  | 2.95 | 7.78E-07 |
| 45 | 308.2689 | 308.2715 | 8 | 470.3602106 | 8,11-Eicosadienoic acid | C20H36O2 |  | 2.95 | 7.78E-07 |
| 45 | 308.2689 | 308.2715 | 8 | 470.3602106 | 8Z,11Z-eicosadienoic acid | C20H36O2 |  | 2.95 | 7.78E-07 |
| 45 | 308.2689 | 308.2715 | 8 | 470.3602106 | Linoleic Acid ethyl ester | C20H36O2 |  | 2.95 | 7.78E-07 |
| 45 | 308.2689 | 308.2715 | 8 | 470.3602106 | 11,14-trans-Eicosadienoic acid | C20H36O2 |  | 2.95 | 7.78E-07 |
| 45 | 308.2689 | 308.2715 | 8 | 470.3602106 | 11(Z),14(Z)-Eicosadienoic Acid | C20H36O2 | C16525 | 2.95 | 7.78E-07 |
| 45 | 308.2689 | 308.2715 | 8 | 470.3602106 | 8(Z),14(Z)-Eicosadienoic Acid | C20H36O2 |  | 2.95 | 7.78E-07 |
| 45 | 308.2689 | 308.2715 | 8 | 470.3602106 | 11, 14-icosadienoic acid; C20:2n-6,9 | C20H36O2 |  | 2.95 | 7.78E-07 |
| 45 | 308.2689 | 308.2715 | 8 | 470.3602106 | 8,11-Eicosadienoic acid | C20H36O2 |  | 2.95 | 7.78E-07 |
| 45 | 308.2689 | 308.2715 | 8 | 470.3602106 | 8Z,11Z-eicosadienoic acid | C20H36O2 |  | 2.95 | 7.78E-07 |
| 45 | 308.2689 | 308.2715 | 8 | 470.3602106 | 5(Z),14(Z)-Eicosadienoic Acid | C20H36O2 |  | 2.95 | 7.78E-07 |
| 46 | 298.2829 | 298.2872 | 14 | 460.3741903 | (+)-16-methyl stearic acid | C19H38O2 |  | 5.01 | 1.52E-02 |
| 46 | 298.2829 | 298.2872 | 14 | 460.3741903 | 4,14-dimethyl-heptadecanoic acid | C19H38O2 |  | 5.01 | 1.52E-02 |
| 46 | 298.2829 | 298.2872 | 14 | 460.3741903 | 2-methyl-octadecanoic acid | C19H38O2 |  | 5.01 | 1.52E-02 |
| 46 | 298.2829 | 298.2872 | 14 | 460.3741903 | 17-methyl-octadecanoic acid | C19H38O2 |  | 5.01 | 1.52E-02 |
| 46 | 298.2829 | 298.2872 | 14 | 460.3741903 | Pristanic acid | C19H38O2 |  | 5.01 | 1.52E-02 |
| 46 | 298.2829 | 298.2872 | 14 | 460.3741903 | Nonadecanoic acid | C19H38O2 | C16535 | 5.01 | 1.52E-02 |
| 46 | 298.2829 | 298.2872 | 14 | 460.3741903 | Tuberculostearic acid | C19H38O2 | C16794 | 5.01 | 1.52E-02 |
| 46 | 298.2829 | 298.2872 | 14 | 460.3741903 | 15-methyl-octadecanoic acid | C19H38O2 |  | 5.01 | 1.52E-02 |
| 46 | 298.2829 | 298.2872 | 14 | 460.3741903 | 5-methyl-octadecanoic acid | C19H38O2 |  | 5.01 | 1.52E-02 |
| 46 | 298.2829 | 298.2872 | 14 | 460.3741903 | 11-methyl-octadecanoic acid | C19H38O2 |  | 5.01 | 1.52E-02 |
| 46 | 298.2829 | 298.2872 | 14 | 460.3741903 | 12-methyl-octadecanoic acid | C19H38O2 |  | 5.01 | 1.52E-02 |
| 46 | 298.2829 | 298.2872 | 14 | 460.3741903 | 13-methyl-octadecanoic acid | C19H38O2 |  | 5.01 | 1.52E-02 |
| 46 | 298.2829 | 298.2872 | 14 | 460.3741903 | 14-methyl-octadecanoic acid | C19H38O2 |  | 5.01 | 1.52E-02 |
| 46 | 298.2829 | 298.2872 | 14 | 460.3741903 | 3-methyl-octadecanoic acid | C19H38O2 |  | 5.01 | 1.52E-02 |
| 46 | 298.2829 | 298.2872 | 14 | 460.3741903 | 4-methyl-octadecanoic acid | C19H38O2 |  | 5.01 | 1.52E-02 |
| 46 | 298.2829 | 298.2872 | 14 | 460.3741903 | 5-methyl-octadecanoic acid | C19H38O2 |  | 5.01 | 1.52E-02 |
| 46 | 298.2829 | 298.2872 | 14 | 460.3741903 | 7-methyl-octadecanoic acid | C19H38O2 |  | 5.01 | 1.52E-02 |
| 46 | 298.2829 | 298.2872 | 14 | 460.3741903 | 8-methyl-octadecanoic acid | C19H38O2 |  | 5.01 | 1.52E-02 |
| 46 | 298.2829 | 298.2872 | 14 | 460.3741903 | 9-methyl-octadecanoic acid | C19H38O2 |  | 5.01 | 1.52E-02 |
| 47 | 296.2689 | 296.2715 | 8 | 458.3601993 | 10-methylene-octadecanoic acid | C19H36O2 |  | 3.37 | 3.47E-09 |
| 47 | 296.2689 | 296.2715 | 8 | 458.3601993 | 2-nonadecenoic acid | C19H36O2 |  | 3.37 | 3.47E-09 |
| 47 | 296.2689 | 296.2715 | 8 | 458.3601993 | 10E-nonadecenoic acid | C19H36O2 |  | 3.37 | 3.47E-09 |
| 47 | 296.2689 | 296.2715 | 8 | 458.3601993 | 10Z-nonadecenoic acid | C19H36O2 |  | 3.37 | 3.47E-09 |
| 47 | 296.2689 | 296.2715 | 8 | 458.3601993 | 7E-nonadecenoic acid | C19H36O2 |  | 3.37 | 3.47E-09 |
| 47 | 296.2689 | 296.2715 | 8 | 458.3601993 | 7Z-nonadecenoic acid | C19H36O2 |  | 3.37 | 3.47E-09 |
| 47 | 296.2689 | 296.2715 | 9 | 458.3601993 | 17-methyl-6Z-octadecenoic acid | C19H36O2 | C13787 | 3.37 | 3.47E-09 |
| 47 | 296.2689 | 296.2715 | 9 | 458.3601993 | 11R,12S-methylene-octadecanoic acid | C19H36O2 | C13838 | 3.37 | 3.47E-09 |
| 47 | 296.2689 | 296.2715 | 9 | 458.3601993 | cis-12-Octadecenoic Acid methyl ester | C19H36O2 |  | 3.37 | 3.47E-09 |
| 47 | 296.2689 | 296.2715 | 9 | 458.3601993 | Phytomonic Acid | C19H36O2 |  | 3.37 | 3.47E-09 |
| 47 | 296.2689 | 296.2715 | 9 | 458.3601993 | 13-Cyclohexyltridecanoic acid | C19H36O2 |  | 3.37 | 3.47E-09 |
| 47 | 296.2689 | 296.2715 | 9 | 458.3601993 | Dihydrosterculic acid | C19H36O2 |  | 3.37 | 3.47E-09 |
| 47 | 296.2689 | 296.2715 | 9 | 458.3601993 | 4-heptyl-2-methyl-2-undecenoic acid | C19H36O2 |  | 3.37 | 3.47E-09 |
| 47 | 296.2689 | 296.2715 | 9 | 458.3601993 | 2,5-dimethyl-2-heptadecenoic acid | C19H36O2 |  | 3.37 | 3.47E-09 |
| 47 | 296.2689 | 296.2715 | 9 | 458.3601993 | Octadecanoic acid, 10-methylene-; 10-Undecenoic acid, 10-octyl-; 10-Methylenestearic acid | C19H36O2 |  | 3.37 | 3.47E-09 |
| 47 | 296.2689 | 296.2715 | 9 | 458.3601993 | 10E-nonadecenoic acid | C19H36O2 |  | 3.37 | 3.47E-09 |
| 47 | 296.2689 | 296.2715 | 9 | 458.3601993 | 10Z-nonadecenoic acid | C19H36O2 |  | 3.37 | 3.47E-09 |
| 47 | 296.2689 | 296.2715 | 9 | 458.3601993 | 7E-nonadecenoic acid | C19H36O2 |  | 3.37 | 3.47E-09 |
| 47 | 296.2689 | 296.2715 | 9 | 458.3601993 | 7Z-nonadecenoic acid | C19H36O2 |  | 3.37 | 3.47E-09 |
| 47 | 296.2689 | 296.2715 | 9 | 458.3601993 | Lactobacillic acid | C19H36O2 |  | 3.37 | 3.47E-09 |
| 47 | 296.2689 | 296.2715 | 9 | 458.3601993 | (E)-9-Octadecenoic acid methyl ester | C19H36O2 |  | 3.37 | 3.47E-09 |
| 48 | 310.2864 | 310.2872 | 2 | 472.3777613 | Phytenoic Acid | C20H38O2 |  | 4.43 | 2.77E-05 |
| 48 | 310.2864 | 310.2872 | 2 | 472.3777613 | 17Z-octadecenoic acid | C20H38O2 |  | 4.43 | 2.77E-05 |
| 48 | 310.2864 | 310.2872 | 2 | 472.3777613 | cis-gadoleic acid | C20H38O2 |  | 4.43 | 2.77E-05 |
| 48 | 310.2864 | 310.2872 | 2 | 472.3777613 | trans-gondoic acid | C20H38O2 |  | 4.43 | 2.77E-05 |
| 48 | 310.2864 | 310.2872 | 2 | 472.3777613 | 14(Z)-Eicosenoic Acid | C20H38O2 |  | 4.43 | 2.77E-05 |
| 48 | 310.2864 | 310.2872 | 2 | 472.3777613 | 13-eicosenoic acid | C20H38O2 |  | 4.43 | 2.77E-05 |
[truncated: 13,866 more chars]
